# Getting a molecular grip on the half-lives of iminothioindoxyl photoswitches†

**DOI:** 10.1039/d4sc01457j

**Published:** 2024-07-26

**Authors:** Melody E. Boëtius, Mark W. H. Hoorens, Martin Ošťadnický, Adèle D. Laurent, Mariangela di Donato, Aldo C. A. van Wingaarden, Michiel F. Hilbers, Ben L. Feringa, Wybren Jan Buma, Miroslav Medveď, Wiktor Szymanski

**Affiliations:** a Department of Radiology, Medical Imaging Center, University Medical Center Groningen Hanzeplein 1 9713GZ Groningen The Netherlands w.c.szymanski@rug.nl; b Center for Systems Chemistry, Stratingh Institute for Chemistry, University of Groningen Nijenborgh 7 Groningen The Netherlands; c Department of Medicinal Chemistry, Photopharmacology and Imaging, Groningen Research Institute of Pharmacy, University of Groningen A. Deusinglaan 1 9713 AV Groningen The Netherlands; d Faculty of Natural Sciences, Comenius University Ilkovičova 6 SK-842 15 Bratislava Slovak Republic; e Nantes Université, CNRS CEISAM UMR 6230 F-44000 Nantes France; f LENS, European Laboratory for Non-Linear Spectroscopy 50019 Sesto Fiorentino FI Italy; g CNR-ICCOM via Madonna del Piano 10 50019 Sesto Fiorentino (FI) Italy; h Van't Hoff Institute for Molecular Sciences, University of Amsterdam Science Park 904 1098 XH Amsterdam The Netherlands; i Institute for Molecules and Materials, FELIX Laboratory, Radboud University Toernooiveld 7c 6525 ED Nijmegen The Netherlands W.J.Buma@uva.nl; j Faculty of Natural Sciences, Department of Chemistry, Matej Bel University Tajovského 40 SK-97400 Banská Bystrica Slovak Republic miroslav.medved@upol.cz; k Regional Centre of Advanced Technologies and Materials, Czech Advanced Technology and Research Institute (CATRIN), Palacky University Olomouc Křížkovského 511/8 77900 Olomouc Czech Republic

## Abstract

Visible-light-operated photoswitches are of growing interest in reversibly controlling molecular processes, enabling for example the precise spatiotemporal focusing of drug activity and manipulating the properties of materials. Therefore, many research efforts have been spent on seeking control over the (photo)physical properties of photoswitches, in particular the absorption maxima and the half-life. For photopharmacological applications, photoswitches should ideally be operated by visible light in at least one direction, and feature a metastable isomer with a half-life of 0.1–10 seconds. Here we present our efforts towards the engineering of the half-life of iminothioindoxyl (ITI) photoswitches, a recently discovered class of visible-light-responsive photochromes, whose applicability was hitherto limited by half-lives in the low millisecond range. Through the synthesis and characterization of a library of ITI photoswitches, we discovered variants with a substantially increased thermal stability, reaching half-lives of up to 0.2 seconds. Based on spectroscopic and computational analyses, we demonstrate how different substituent positions on the ITI molecule can be used to tune its photophysical properties independently to fit the desired application. Additionally, the unique reactivity of the ITI derivative that featured a perfluoro-aromatic ring and had the most long-lived metastable state was shown to be useful for labeling of nucleophilic functional groups. The present research thus paves the way for using ITI photoswitches in photopharmacology and chemical biology.

## Abstract 2

Molecular engineering of an ITI photoswitch, supported by DFT calculations and advanced spectroscopy, allows the half-life of its unstable state to be boosted to the levels needed for biological applications.

## Graphical abstract

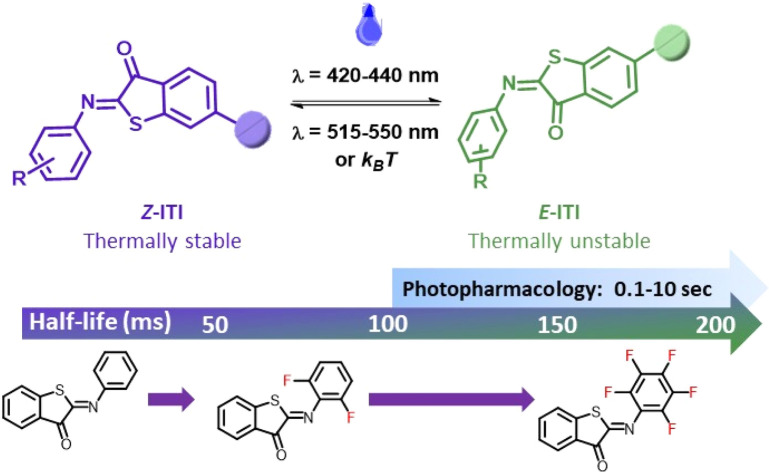

## Introduction

Recent years have witnessed a surge in the development of molecular photoswitches^1–3^ that have found applications in, *e.g.*, materials science, optical information storage, chemical biology, and photopharmacology.^4–8^ Their increasing popularity stems from their capability to enable reversible control over a system of interest and the precision with which they can be used to manipulate (bio)chemical processes using light.^1,9,10^ A key advantage in such applications is that light offers high spatiotemporal resolution and bio-orthogonality.^3,9–12^

Though most photoswitches require UV light for their photoisomerization in at least one direction,^2,3,13–15^ the use of UV light is often harmful and has limited their applications *in vivo*^2,16^ and in materials sciences.^16–18^ Because of its high energy, UV light not only damages living cells, but is also non-selectively absorbed by surrounding matter, which restricts its ability to penetrate tissues.^10,16^ As a result, there has been a growing interest in visible-light operable photoswitches, since the use of visible light is more convenient and not associated with adverse effects.^1,2,14,19^ With this in mind, considerable efforts have been spent on bathochromically shifting the absorption spectra of photoswitches for *in vivo* applications,^1,2,19^ since light penetration generally increases in (human) tissues with increasing wavelength.^9^ This has been achieved for example by incorporating *para*-electron donating groups (EDGs) and/or extending the π-conjugation in azobenzenes,^19^ azonium ions,^20,21^ azo-BF_2_ photoswitches,^22^ and indigoid photoswitches.^14,23–26^ Other approaches relied on protonation;^20,21,27,28^ creating push–pull substituted systems^1,3,11,29^ or enforcing (co)planarization *e.g.* in hydrazones^30^ or in azobenzenes to create diazocines.^31–35^ Other methods yet include introducing substituents in the *ortho*-positions of azobenzenes and azonium ions^20,21,36^ or *N*-functionalization of indigoid (and related) photoswitches.^23,37,38^

Another key feature of photoswitches, which defines their fit for a given application, is the thermal stability of their metastable isomer.^11,30,39–41^ The so-called T-type photoswitches, in particular, only require light irradiation for switching in one direction, namely for conversion to the metastable isomer. This isomer then thermally reverts to the stable isomer, thereby avoiding the need for a second wavelength. The half-life of this process is crucial and can range from nanoseconds to years.^3,41^ The required half-life of the metastable form depends on the specific application for which the photoswitch is to be used.^3,42^ In super-resolution imaging, for example, half-lives of nano- to microseconds are needed for fast data acquisition.^43,44^ In biomedical applications (photopharmacology), two different scenarios can be envisioned.^3^ On the one hand, half-lives of several hours may be needed.^3^ In those cases, a photocontrolled drug that shows higher potency in its metastable state is activated before being administered to the patient and switches back to its inactive form when excreted, making it useful for preventing *e.g.* the development of antibiotic resistance.^3,45^ Photoswitches that could be used in these scenarios include azobenzenes, hemithioindigos (HTIs), and spiropyrans.^3^ On the other hand, for applications in which the drug is locally activated in the human body, an attractive approach is to use photoswitches that feature faster thermal isomerization from the active to the inactive form. Such molecules – especially when visible light can be used for their activation – allow for precise confinement of drug activity solely in the irradiated spot.^12^ If the half-life of the metastable isomer is too short, it will not accumulate enough to achieve the desired therapeutic effect. However, if the half-life is too long, it would not result in the desired local therapeutic effect due to diffusion and the blood flow. For such applications, it has been suggested that half-lives of 0.1–10 seconds are ideal.^46^ Photoswitches that are suitable for these applications include azonium ions and azobenzenes with a push–pull system.^3,11,20,21,41,46^ However, the introduction of such extended molecules into drugs results in a large structural change. Moreover, some azobenzenes have been proven to be unstable under reducing conditions in cells.^33,46,47^

Recently, we have reported a family of small, visible light-responsive photoswitches known as iminothioindoxyls (ITIs). With their short half-lives of up to 20 milliseconds at room temperature, they hold promise for applications that require a rapid responses,^13,48^ such as photoactuators in optical lenses.^7,42^ Although their small size as well as solubility and stability in water^13,47^ are potentially useful for photopharmacology, their half-lives are too short and need improvement.^13,27^ However, tailoring the half-life of molecular photoswitches without compromising other photophysical properties is often challenging.^3,11^

**Scheme 1:**
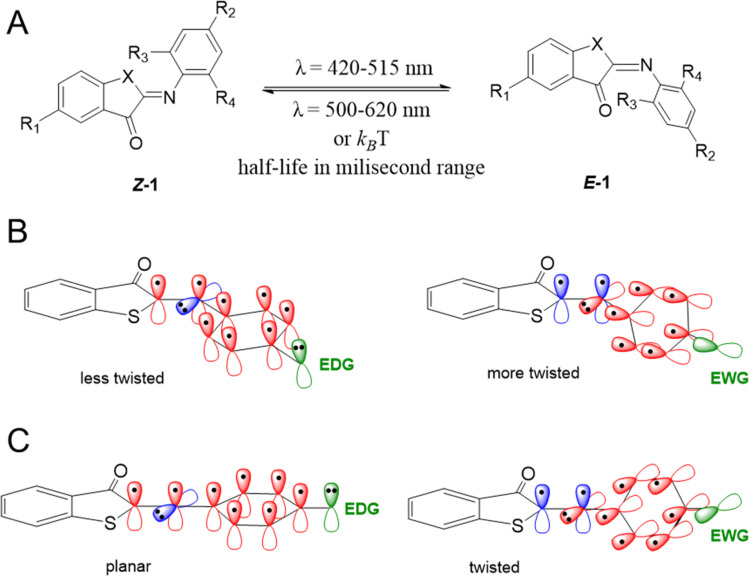
(A) Structures of the two isomers of iminothioindoxyl (ITI) with varying substituents on the thioindoxyl and phenyl moieties. Switching to the *E*-isomer occurs under irradiation at 420–515 nm and back-switching under irradiation at 500–620 nm. (B) The influence of electron-donating groups (EDGs) (left) and electron-withdrawing groups (EWGs) (right) in the R_2_ position on the twisting of the *Z*-isomer structure. (C) The influence of EDGs (left) and EWGs (right) in the R_2_ position on the structure of the transition state. Valence p orbitals of atoms in the central part of molecule and the phenyl moiety entering the π-conjugation, atomic orbitals of EDG/EWG, and orbitals not participating in the conjugation with the phenyl ring are drawn in red, green, and blue, respectively.

Here we describe the design, synthesis, and spectroscopic and computational analyses of ITI photoswitches, for which the half-life can be controlled through molecular engineering by over two orders of magnitude. Inspired by previously reported ITIs,^13,42^ and structurally similar azobenzene^16,19^ and HTI^15,49^ photoswitches, several positions on the parent ITI molecule were identified (Scheme 1A), through which spectrochemical features and the half-life of the metastable isomer could be independently modulated. Furthermore, we investigated the influence of these substitution patterns on the half-life of ITIs through a combination of theoretical calculations and spectroscopy, with the goal of increasing the half-life to at least 0.1 seconds for photopharmacological applications. These studies revealed the structure–photophysical properties relationships in the ITI molecule and enabled the engineering of the desired half-life, which could be achieved through manipulating the electron density on the phenyl aromatic ring.

## Results & discussion

This work was initiated by a systematic evaluation of the influence of the substituents (R_1_–R_4_ and X, Scheme 1A) on the photophysical properties of the switch, including the position of absorption maxima for both isomers, the thermal half-life of the metastable *E* isomer and the forward switching quantum yield. All molecules were synthesized through a reaction of the indoxyl precursors with substituted nitrosobenzenes, either in benzene with piperidine as a catalyst at 80 °C, or under newly developed, more environmentally friendly conditions (KOH in ethanol, RT). All the synthetic schemes, experimental procedures, and analytical data are reported in the ESI.†

To understand how a substituent in a specific position can affect the photophysical properties of ITIs, it is worth recalling some observations revealed in our pilot study of *para*-phenyl substituted ITIs.^14^ Firstly, both *Z* and *E* forms of ITIs are twisted (Scheme 1B), and the twisting angle (being systematically larger for the *E* isomer due to repulsion between the carbonyl oxygen and the hydrogen atom in the R_3_ position) can be tuned depending on the nature of a substituent.^50^ The electron-withdrawing groups (EWGs) bring about a larger twist, while electron-donating groups (EDGs) cause the reverse, which leads to a small auxochromic shift of the absorption maxima for EWGs and bathochromic shift for EDGs. Secondly, the nature of a substituent also significantly affects the thermal back relaxation pathway. Whereas the transition state (TS) structure of EWG derivatives adopts a perpendicular arrangement similar to that of the parent (unsubstituted) ITI, in the case of EDG derivatives the TS is planar (Scheme 1C). The different geometry of the TS strongly modifies the expected correlation between the activation energy and the Hammett constants of substituents. The preference of the EDG derivatives for less twisted *Z*, *E*, and TS structures can be explained by an intricate interplay between the valence orbitals of the central nitrogen atom and those of the phenyl moiety. In the *Z* and *E* forms, the nitrogen is sp^2^-hybridized. Whereas in quasi-planar structures (Scheme 1B, left) the π-orbitals on the phenyl ring conjugate with the C=N double bond (negative mesomeric effect, −M), in strongly twisted structures (Scheme 1B, right) the phenyl's π-orbitals mainly interact with the nitrogen lone pair (positive mesomeric effect, +M). An EWG in the *para*-position favors pairing with the lone pair, thereby leading to a more twisted structure. In contrast, EDGs, by increasing the electron density on the phenyl ring, bring about partial planarization of the structures. Such planarization enables better π-electron delocalization over the whole molecule. These effects are even more pronounced for the transitions state (Scheme 1C), where the nitrogen is sp-hybridized. In this case, the lone pair can be either in-plane (in the case of EDG) or can conjugate with the p orbitals of the phenyl ring (EWG). Since there is no steric hindrance of the thioindoxyl and phenyl moieties (in the case of unsubstituted *ortho* positions on the phenyl ring), the introduction of an EDG leads to a full planarization of the structure.

### Sulfur substitution (X, Scheme 1A)

The first modification point explored in the ITI molecule is at the sulfur atom. This position was previously substituted with the bulkier N–Ac to obtain phenylimino indolinone (PIO) photoswitches,^42^ which not only displayed negative photochromism and an inverted stability compared to ITI, but also had a much lower half-life of several tens to hundreds of μs.^42^ To further explore the effects of substitution on this position (X in Table 1), we substituted sulfur for selenium. This substitution resulted in a slight red shift of the absorption maxima of both *Z* and *E* forms, and a higher quantum yield (*Φ*, Table 1). This red shift was also observed in rhodanine-based dyes in which an oxygen was substituted for the larger and less electronegative sulfur.^51^ In ITIs, however, this substitution led to a much shorter half-life than the parent ITI. Density Functional Theory (DFT) calculations revealed that the TS structure preserves the perpendicular arrangement (Fig. S106†), since the presence of the slightly less electronegative and larger selenium atom in the thioindoxyl moiety does not improve the π-electron delocalization from the phenyl moiety through the C=N double bond. This is also reflected in the structure of the *E*-isomer, which is notably more twisted for 1b compared to 1a (*θ*_CNCC_ = 93.4 *versus* 70.4°, Fig. S106†), indicating its lower stability and presumably being the reason for the decreased activation barrier.

**Table 1:** Photophysical properties of the ITI photoswitches substituted in the X position^a^

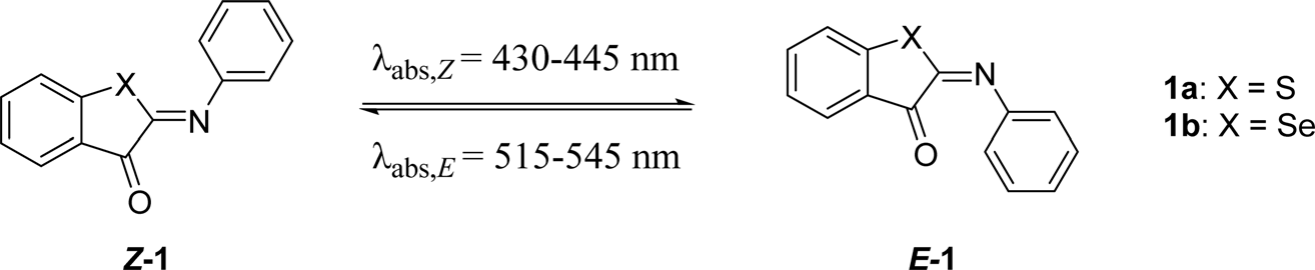								
	X	*λ*_max,*Z*_ (nm)	*λ*_max,*E*_ (nm)	*Φ*_*Z*–*E*_ (%)	*ε*_*Z*_ (M^−1^ cm^−1^)	Δ*G*°^#^ (kcal mol^−1^) calc.	Δ*G*°^#^ (kcal mol^−1^) exp.	*t*_1/2_ (ms)
1a	S	429	**515**, 552	6.2	4300	13.3 (t)	15.0	18.5
1b	Se	446	546	16.2	1800	12.8 (t)	13.8	2.3

a Position of the absorption band (*λ*_max_), extinction coefficients for the *Z*- and *E*-isomers and quantum yields of forward switching of ITIs 1a and 1b in MeOH. Where possible, experimental *λ*_max,*E*_ values are obtained from transient absorption (TA) spectra where two absorption maxima are identified; the most intense absorption is highlighted in bold. Theoretical activation barriers for thermal relaxation were obtained at the M06-2X/6-31+G(d)//6-311++G(2df, 2p) level. The nature of TS is specified in parentheses; t/p stands for twisted/planar structure. Experimental activation energies were calculated with the Eyring equation (with *κ* = 1). Thermal relaxation rates of ITIs 1a and 1b were recorded in MeOH. Experimental half-lives were obtained from ns TA spectroscopy. Data for 1a have been reported earlier and are reported for comparison.^13^

### Thioindoxyl moiety substitutions (R_1_, Scheme 1A)

The second group of ITIs that was explored involved derivatives with a substituent on the thioindoxyl moiety, in *para* position to the sulfur atom. For HTIs, Kink *et al.*^49^ have discovered that substitutions at this position can be used to tune the absorption maximum, generally without affecting the half-lives. Due to the structural resemblance between ITIs and HTIs, similar substituent effects were anticipated here. Indeed, introducing EDG substituents at this position gave a bathochromic shift, which increased with the ED strength of a substituent in 1e–g (Table 2), while the EWG substitution in *Z*-1c led to a slight blue shift. DFT calculations revealed that the shifts mainly result (i) from the destabilization and stabilization of the π-HOMO in EDG and EWG substituted ITIs, respectively, and (ii) from the slightly stronger n–π* character of the transition in 1c (Scheme 1B and Table S1†). With EWGs, the HOMO spreads over the entire molecule (*i.e.* it is stabilized), since the phenyl group (ED) can efficiently delocalize electrons to the thioindoxyl moiety (Fig. S113†). This delocalization is hampered in *E*-isomers due to a larger twisting (Fig. S114†), which results in a large band separation between the isomers of 1c (Table 2). Having observed a significant red shift for the EDG methoxy substituent (1f), we set out to see if the incorporation of an even stronger ED dimethylamino substituent (1g) would result in an ITI photoswitch with improved properties. However, theoretical calculations suggested that with R_1_ = NMe_2_ the thioindoxyl moiety becomes electron-donating, through which the HOMO of *Z*-1g remains localized on the thioindoxyl moiety, similar to its *E*-isomer (Fig. S113 and S114†). This not only results in a narrow band separation due to the overlap of the S_0_ → S_1_ bands of both the *Z*- and *E*-isomers, but also red shifts the absorption maximum of the *Z*-isomer beyond that of the *E*-isomer (Fig. S115†). Although TD-DFT calculations predict a better band separation for the S_0_ → S_2_ transition (Table S1†), transient absorption spectroscopy did not indicate the occurrence of substantial isomerization, regardless of whether it was excited within the S_1_ or S_2_ state (compare Fig. S85a and d†), indicating that 1g does not properly switch. Weaker EDGs (1e, 1f), on the other hand, did not seem to influence the half-life – similar to HTIs^49^ – while substitution with EWGs resulted in slightly lower half-lives (1c, 1d). If one avoids the substitution with strong EDGs, the R_1_ position appears to be useful as a reactivity handle for introducing functional groups for, *e.g.*, coupling to drugs or proteins without severely affecting the photophysical properties of the assembly.

**Table 2:** Photophysical properties of the ITI photoswitches substituted in the R_1_ position^a^

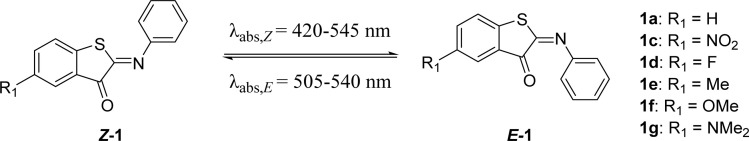									
	R_2_	Hammett (*R*)	*λ*_max,*Z*_ (nm)	*λ*_max,*E*_ (nm)	*Φ*_*Z*–*E*_ (%)	*ε*_*Z*_ (M^−1^ cm^−1^)	Δ*G*°^#^ (kcal mol^−1^) calc.	Δ*G*°^#^ (kcal mol^−1^) exp.	*t*_1/2_ (ms)
1a	H	0	429	515	6.2	4300	13.3 (t)	15.0	18.5
1c	NO_2_	0.78	419	527	10.9	2900	12.7 (t)	14.7	10.3
1d	F	0.06	435	537	6.8	4600	13.1 (t)	14.9	14.6
1e	Me	−0.17	437	536	6.5	4200	13.6 (t)	15.0	18.0
1f	OMe	−0.27	460	540	3.9	1400	14.0 (t)	15.1	20.0
1g	NMe_2_	−0.83	545	504 (calc)	N.A.	1000	13.6 (t)	N.A.	N.A.

a Position of the absorption band (*λ*_max_), extinction coefficients for the *Z*- and *E*-isomers and quantum yields of forward switching of ITIs 1c–g in MeOH. Experimental *λ*_max,*E*_ values are obtained from TA. Theoretical activation barriers for thermal relaxation were derived at the M06-2X/6-31+G(d)//6-311++G(2df, 2p) level. The nature of TS is specified in the parentheses; t/p stands for twisted/planar structure. Experimental activation energies were calculated with the Eyring equation (with *κ* = 1). Thermal relaxation rates of ITIs 1c–g were recorded in MeOH. Experimental half-lives were obtained from ns TA spectroscopy. Data for 1a have been reported earlier and are reported for comparison.^13^

### Substitutions on the aromatic imine: *para*-position (R_2_, Scheme 1)

Next, we screened the influence of the *para*-position in the aromatic imine moiety. Given that ITIs are a hybrid of HTI and azobenzene photoswitches,^13^ substituent effects comparable to those observed in azobenzenes were expected at first for ITIs.^16,49,52^ In azobenzenes, substitution at this position allows for tuning of the absorption maximum. However, it is worth noticing that in azobenzenes the introduction of strong EDGs also affects the half-life of their metastable *Z-*isomer. Substitution at the *para*-position of ITIs has previously been shown to influence the absorption maximum. Whereas modifications by substituents with an increasingly negative Hammett constant (*i.e.*, EDGs) resulted in a larger red shift for both the *Z*- and *E*-isomers, a blue shift was observed for *E*-isomers with substituents characterized by positive Hammett constants (Table 3),^13^ similar to azobenzenes^16,52^ and HTIs.^49^ Herein, this library of *para*-substituted ITIs has been further expanded with two compounds (1i and 1k in Table 3).

**Table 3:** Photophysical properties of the ITI photoswitches substituted in the R_2_ position^a^

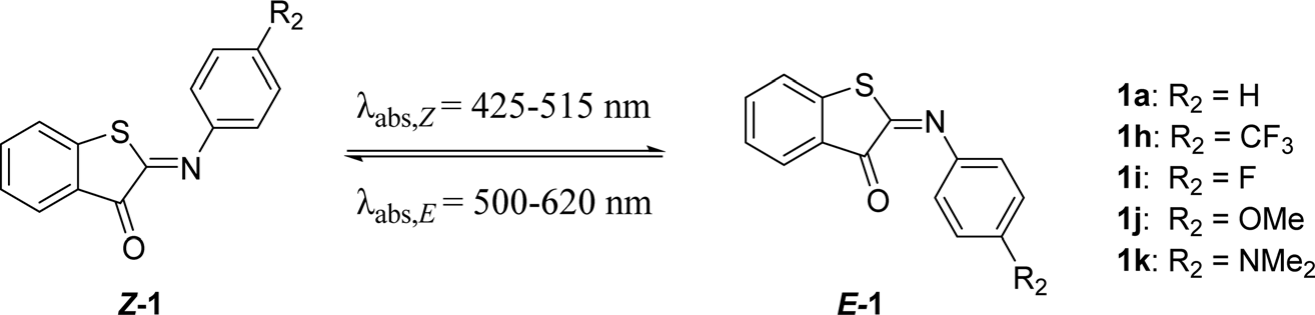									
	R_2_	Hammett (*R*)	*λ*_max,*Z*_ (nm)	*λ*_max,*E*_ (nm)	*Φ*_*Z*–*E*_ (%)	*ε*_*Z*_ (M^−1^ cm^−1^)	Δ*G*°^#^ (kcal mol^−1^) calc.	Δ*G*°^#^ (kcal mol^−1^) exp.	*t*_1/2_ (ms)
1a	H	0	429	515	6.2	4300	13.3 (t)	15.0	18.5
1h	CF_3_	0.54	424	500	4.9	2100	13.1 (t)	14.5	7.5
1i	F	0.06	425	**512**, 542	0.4	4000	13.8 (p)	15.2	22.9
1j	OMe	−0.27	448	516, **553**	4.5	11 000	13.2 (p)	14.3	5.3
1k	NMe_2_	−0.83	516	620	6.2 × 10^−3^	27 000	10.3 (p)	13.8	2.0

a Position of the absorption band (*λ*_max_), extinction coefficients for the *Z*- and *E*-isomers and quantum yields of forward switching of ITIs 1h–k in MeOH. Experimental *λ*_max,*E*_ values are obtained from TA spectra where two absorption maxima are identified; the most intense absorption is highlighted in bold. Theoretical activation barriers for thermal relaxation were derived at the M06-2X/6-31+G(d)//6-311++G(2df, 2p) level. The nature of TS is specified in the parentheses; t/p stands for twisted/planar structure. Experimental activation energies were calculated with the Eyring equation (with *κ* = 1). Thermal relaxation rates of ITIs 1h–k were recorded in MeOH. Experimental half-lives were obtained from ns TA spectroscopy. Data for 1a, 1i, and 1j have been reported earlier and are reported for comparison.^13^

As expected, 1k displayed the most red shifted absorption maximum mainly due to the significant destabilization of the π-HOMO (Fig. S117 and S118†) in the least twisted *Z* and *E* structures (Fig. S107†). In addition, the S_0_ → S_1_ excitation in 1k has strong charge transfer character (see HOMO, LUMO and EDD plots in Fig. S116–S118† and dipole moment values in Table S17†) analogous to that observed for *para*-amine derivatives of HTIs,^53^ which further stabilizes the S_1_ state in polar solvents such as methanol. On the other hand, 1k also exhibited the shortest half-life compared to previously reported *para*-substituted ITIs, together with a very low forward switching quantum yield (1a, 1h, 1j).^13^ This combination of red shifting and shortened half-lives is also observed for HTIs,^53^ azobenzenes and other heteroaryl azoswitches.^11,14,15,30^ However, no clear correlation between the Hammett parameter and the half-life was found due to different thermal relaxation pathways for EDG and EWG derivatives as explained above. Based on these results, it appears that this position is more useful for tuning the absorption maximum than tuning the thermal stability of the *E*-isomer.

### Substitutions on the aromatic imine-moiety: *ortho*-position (R_3_, R_4_, Scheme 1A)

Finally, we investigated the structure–photophysical properties relationships for *ortho*-positions on the aromatic imine moiety. Substituents at the *ortho*-position in azobenzenes tend to influence the stability of the metastable isomer, and thereby the half-life.^11,16,54^ Several *ortho*-substituted ITIs (1l–w) were synthesized to evaluate whether the same holds true for ITIs (Table 2). The absorption maxima of these *ortho*-substituted *Z*-isomers correspond to the S_0_ → S_1_ transition and lie between 420–440 nm (Table 2), with a slight red shift observed for mono-substituted alkyl and methoxy derivatives (1l, 1m, 1o, 1p). Importantly, the bathochromic shift brought by substitution with EDGs was also observed in the case of *para*-substituted ITIs and can be rationalized in similar terms, that is, that EDGs favor delocalization of the increased electron density on the phenyl ring, resulting in a less twisted structure (Scheme 1B, Fig. S108†) with extended π-conjugation and thus a smaller HOMO(π)–LUMO(π*) gap (Fig. S121 and S122†). However, a double EDG substitution in *Z*-1n brings about a slight blue shift due to the steric hindrance of the methyl group with the sulfur atom, forcing the structure to be more twisted (−90.7°, Fig. S108†) and thus hampering the π-conjugation (Fig. S121†).

The thermal stability of metastable isomers can be effectively tuned by substitutions in *ortho*-position(s) (Table 4). Similar to *para*-substituted ITIs, most of the substitutions resulted in shorter half-lives, irrespective of the electronic properties of the substituents (*cf.*, compounds 1m, 1n, 1o, 1p, 1r, 1w). Strikingly, a very strong positive influence on the half-life was observed for *ortho*-fluorine substitutions. While the mono-substitution (1u) increased the half-life about two times compared to the parent ITI (1a), the desired half-life of at least 0.1 seconds was nearly reached when this small and σ-electron-withdrawing atom was substituted in both *ortho*-positions (1v). The same effect was observed in *ortho*-fluorinated azobenzenes, and was attributed to the lowering of the n-orbital energy of the N=N-bond.^16^ Importantly, no clear correlation was found when comparing the thermal stability and absorption maximum of different *ortho*-substituted ITIs. This indicates that the *ortho*-position can be used to independently modulate the thermal stability to fit a desired ITI application.

**Table 4:** Photophysical properties of the ITI photoswitches substituted in the R_3_ and R_4_ position^a^

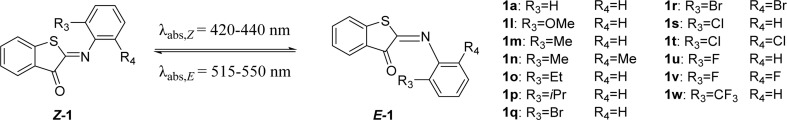									
	R_3_	R_4_	*λ*_max,*Z*_ (nm)	*λ*_max,*E*_ (nm)	*Φ*_*Z*–*E*_ (%)	*ε*_*Z*_ (M^−1^ cm^−1^)	Δ*G*°^#^ (kcal mol^−1^) calc.	Δ*G*°^#^ (kcal mol^−1^) exp.	*t*_1/2_ (ms)
1a	H	H	429	515	6.2	4300	13.3 (t)	15.0	18.5
1l	OMe	H	438	546	6.1	3500	13.3 (p)	14.9	15.4
1m	Me	H	432	551	12.2	1600	13.2 (p)	14.1	3.7
1n	Me	Me	417	549	2.1	2500	13.4 (d)	13.2	0.8
1o	Et	H	433	543	6.2	3000	13.8 (p)	14.0	3.1
1p	iPr	H	432	542	5.7	3000	13.4 (d)	14.0	3.0
1q	Br	H	425	520	4.6	2100	13.7 (t)	14.7	9.8
1r	Br	Br	423	523	3.6	1500	13.4 (t)	14.4	5.9
1s	Cl	H	426	520	8.4	1400	13.6 (t)	14.7	10.5
1t	Cl	Cl	421	521	2.6	2700	14.2 (t)	14.5	7.6
1u	F	H	428	517	7.5	2000	13.8 (p)	15.4	36.5
1v	F	F	421	515	4.8	2100	14.8 (d)	15.9	83.3
1w	CF_3_	H	425	519	7.9	1400	13.1 (t)	14.2	4.2

a Position of the absorption band (*λ*_max_), extinction coefficients for the *Z*- and *E*-isomers and quantum yields of forward switching of ITIs 1l–w in MeOH. Experimental *λ*_max,*E*_ values are obtained from TA. Theoretical activation barriers for thermal relaxation were derived at the M06-2X/6-31+G(d)//6-311++G(2df, 2p) level. The nature of TS is specified in the parentheses; t/d/p stands for twisted/distorted/planar structure. Experimental activation energies were calculated with the Eyring equation (with *κ* = 1). Thermal relaxation rates of ITIs 1l–w were recorded in MeOH. Experimental half-lives were obtained from ns TA spectroscopy. Data for 1a have been reported earlier and are reported for comparison.^13^

In order to rationalize these observations and elucidate the effect of *ortho*-substituents on the half-life, we performed DFT calculations at the M06-2X/6-31+G(d)//6-311++G(2df, 2p) level for the TS and the *E*-isomer. In general, the calculated standard Gibbs activation energies (Δ*G*°^#^, *T* = 298.15 K) of the thermal back-isomerization fairly well reproduce the experimental data (Table 4). The first important outcome of the DFT analysis is that the TSs can again be grouped into two subclasses according to the dihedral angle (*θ*) describing the mutual twisting of thioindoxyl and phenyl moieties. As in the case of *para*-substituted ITIs, the EWG substitutions (–CF_3_, –Cl, –Br) lead to strongly twisted structures (*t*-TS) facilitating electron donation from the lone pair of the nitrogen to the phenyl aromatic ring. The increased stabilization of the n-orbital by its +M effect in the presence of an EWG compared to the parent ITI explains the shortening of the half-life in this subclass. The bulkiness of a substituent does not play a major role here, although it can contribute to the destabilization of the *E*-form in the case of bi-substitution, inducing a slight decrease of half-life (*cf.*1q*vs.*1r and 1s*vs.*1t). On the other hand, EDGs bring about the planarization of the TS structure (1l, 1m, 1o) because of the aforementioned redistribution of electron density on the phenyl ring (Scheme 1C, Fig. S108†). In the case of the *i*Pr group (1p), the planar structure is only slightly less stable than the distorted one (Table S9†). Although the planarization is accompanied by the destabilization of the n-orbital and the π-HOMO, it is compensated by the stabilization of lower π-orbitals as shown *e.g.* for 1m (Table S16†), also resulting in a decrease of the half-life (compared to 1a).

**Fig. 1:**
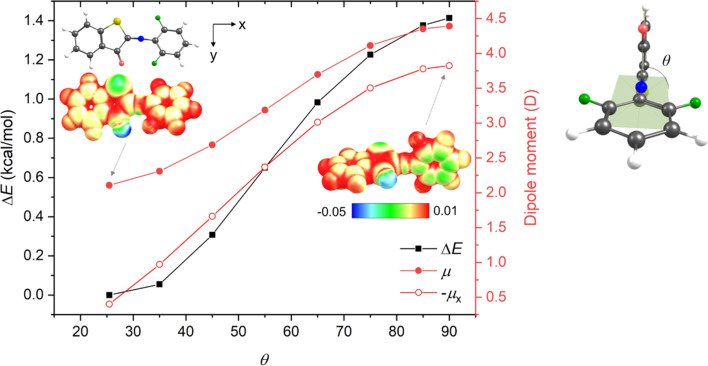
Relative electronic energy (Δ*E*, kcal mol^−1^), total dipole moment (*μ*, Debye) and negative longitudinal component of dipole moment (−*μ*_*x*_, Debye) of 1v as a function of the *θ* dihedral angle (defined on the right) obtained by unrelaxed scan starting from the optimized structure (*θ* = 25.5°) at the M06-2X/6-311++G(2df, 2p) level. Electrostatic potential surfaces (insets) for *θ* = 25.5° and 90° illustrate the larger accumulation of the negative charge (greenish area) in the phenyl moiety in the twisted structure.

Interestingly, this is not the case for fluorine substitutions, which – despite acting as EWGs – exhibit planar (or only slightly distorted in the case of 1v) TSs but give rise to longer thermal half-lives. To understand the uniqueness of the fluorinated compounds, we analyzed the key structural parameters and electronic features of the *E*-isomers and TSs of *ortho*-methyl, *ortho*-fluoro and di-*ortho*-fluoro derivatives (1m, 1u, and 1v) (Table S16†). This analysis revealed that (i) the C=N/N–C bond lengths increase/decrease in the order 1m, 1u, and 1v, indicating that the conjugation is enhanced by fluorine substituents in the *E*-form as well as in the TS; (ii) the increase of n-orbital energy is the dominant factor accompanying the formation of the TS; however, it does not explain by itself the differences between the systems, and one needs in fact to sum the orbital energies of at least six higher occupied MOs (HOMO – HOMO-5) (Fig. S123†) to qualitatively account for the trend of electronic activation energies; and (iii) thermal enthalpic and entropic contributions also partly contribute to the smaller activation barrier of 1m (Tables S4.9 and S4.12†). We thus conclude that disentangling the role of fluorine is not straightforward. It seems that the determining factor is the strong –I effect of fluorine atom, which – due to its high electronegativity – leads to the accumulation of negative charge on the phenyl moiety through σ-bonds. Such charge partitioning (polarization) is energetically unfavorable and induces the planarization of the TS structure (Fig. 1). This helps to reach a more uniform electron distribution within the molecule owing to π-electron delocalization, yet the TS remains less stable compared to the parent ITI. In the di-*ortho*-fluoro derivative (1v), the repulsion between one of the fluorine atoms and the carbonyl oxygen atom does not allow for a perfect planarization of the TS structure, which results in a less effective charge redistribution and thus an even higher activation energy. In this way fluorine behaves differently from other EWGs (exhibiting −M) where the decreased electron density in the phenyl π-delocalized system is (partly) recovered from the nitrogen lone pair in the twisted arrangement.

### Optimization of the half-life through the engineering of the complete aromatic imine ring

Based on the promising results for 1v, another series of fluorinated ITIs was designed to further increase the half-life (Table 5). In particular, we expected that increasing the number of fluorine atoms would improve the half-lives by enhancing the polarization of the ITI molecule, as also predicted by DFT calculations (Table 5).

**Table 5:** Photophysical properties of the ITI photoswitches with a fluorine or chlorine-substituted aromatic ring^a^

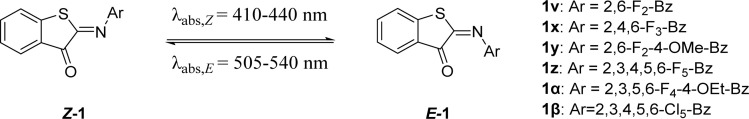							
	*λ*_max,*Z*_ (nm)	*λ*_max,*E*_ (nm)	*Φ*_*Z*–*E*_ (%)	*ε*_*Z*_ (M^−1^ cm^−1^)	Δ*G*°^#^ (kcal mol^−1^) calc.	Δ*G*°^#^ (kcal mol^−1^) exp.	*t*_1/2_ (ms)
1v	421	515	4.8	2100	14.8	15.9	83.3
1x	424	517	4.6	2400	14.5	15.8	70.1
1y	438	541	0.047	4000	13.2	15.2	26.5
1z	423	510	1.1	2000	15.4	16.3	157.6
1α	425	523	5.7	1800	14.6	16.0	104.8
1β	410	505	7.6	600	14.9	14.4	6.3

a Shifts of *λ*_max_, quantum yields, extinction coefficients for the *Z*- and *E*-isomers of ITIs 1v-β in MeOH. Experimental *λ*_max,*E*_ values are obtained from TA. Theoretical activation barriers for thermal relaxation were derived at the M06-2X/6-31+G(d)//6-311++G(2df, 2p) level. Experimental activation energies were calculated with the Eyring equation (with *κ* = 1). Thermal relaxation rates of ITIs 1v-β were recorded in MeOH. Experimental half-lives were obtained from ns TA spectroscopy.

Much to our delight, this strategy indeed led to an increase in the thermal stability of the *E*-isomer (Table 5), with the most promising perfluorinated ITI (1z) featuring a half-life of 0.16 seconds. Compound 1y, on the other hand, exhibited slightly red shifted absorption for both *Z*- and *E*-isomers owing to destabilization of the HOMO (*e.g.*, compared to 1x, Fig. S124 and S125†) but also had a much lower half-life due to the partial compensation of the –I effect of fluorines by the +M effect of the methoxy group. This was also observed for azobenzenes, in which *para*-EDGs counteract the stabilizing effect of fluorine atoms.^16^ It was also discovered that the aromatic phenyl ring of 1z was so electron deficient that it underwent a nucleophilic aromatic substitution (S_N_Ar) reaction with the solvent under basic conditions to form 1α, a derivative with valuable photophysical properties as well (Table 5). This required us to use a different synthetic route for the synthesis of 1z (ESI† page 64), which avoided the use of nucleophilic solvents. While the perfluorinated ITI (1z) gave promising results, its perchlorinated counterpart (1β) yielded a lower half-life than expected. This shorter half-life of 1β is caused by the smaller –I effect of chlorine compared to fluorine. Unlike 1z, the TS structure of 1β attains perpendicular arrangement, and the *Z* and *E* structures are more twisted due to a larger repulsion of chlorines with the oxygen and sulfur atoms (Fig. S110†), which also brings about a blue shift of their absorption maxima (Table 5).

### Reactivity of perfluoro-ITI

**Fig. 2:**
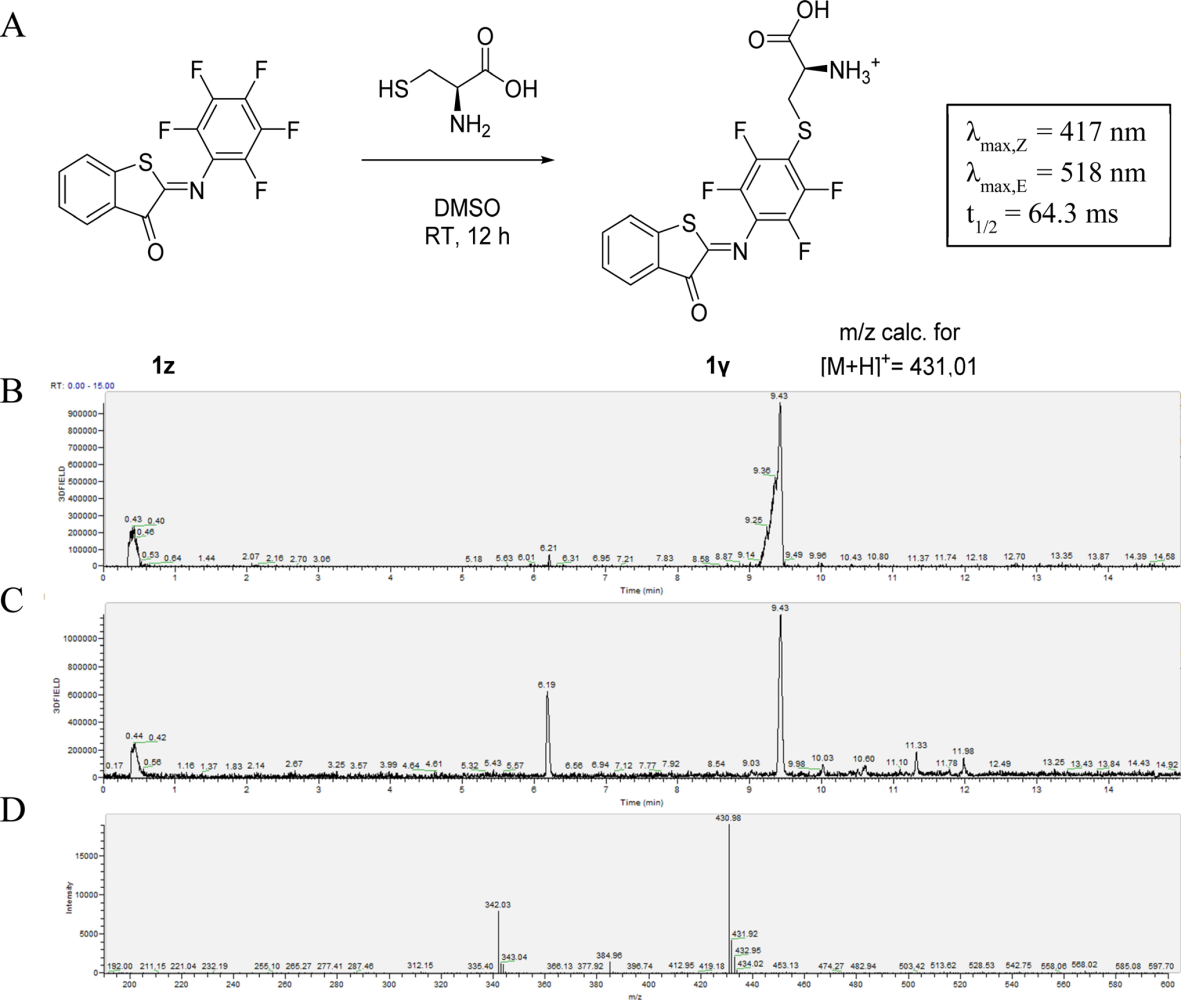
(A) Nucleophilic aromatic substitution of 1x with l-cysteine to form 1γ. LC-trace of the reaction to 1γ after 2 hours (B) and 8 hours (C). (D) Mass-spectrum (positive mode) of the peak corresponding to 6.20 minutes. NB. The depicted structure of the product is one of the possible regioisomers resulting from the substitution of different fluorine atoms.

The susceptibility of 1z to S_N_Ar reaction inspired us to take advantage of its reactivity for coupling to a nucleophilic thiol group, which is present for example in peptides and proteins and is often the target for the incorporation of molecular photoswitches to control biological activity.^9,10,55,56^ The possibility of using 1z to label biological thiols was explored by reacting it with l-cysteine. We observed the clean formation of the adduct after 8 hours of reaction. MS analysis confirmed the formation of the S_N_Ar product 1γ instead of the product of a competing addition to the C=N-bond that is responsible for photo-isomerization (Fig. 2).^13^ This was also confirmed by the observation that 1γ still shows photoswitching in solution. Moreover, this reaction with l-cysteine also slightly increased the solubility of 1z in aqueous media at micromolar concentrations. However, when 1z was added to a solution of 5 mM glutathione in phosphate buffer, a decrease in the absorption maximum was observed. This likely indicates reduction of the C=N-bond, as is also observed for electron-poor azobenzenes.^47^

### Stability of 1z and 1β

One of the possible side-reactions of halogen-substituted HTIs is the irreversible intramolecular cyclization upon *Z* → *E* photoisomerization.^15,57^ To determine whether the same applied for ITIs, the stability of 1z and 1β was measured at 20 °C and at 40 °C in MeOH under continuous irradiation with 420 nm light. For both 1z and 1β, no (significant) degradation was observed (Fig. S79 and S78†) nor visible precipitation.

## Conclusions

**Fig. 3:**
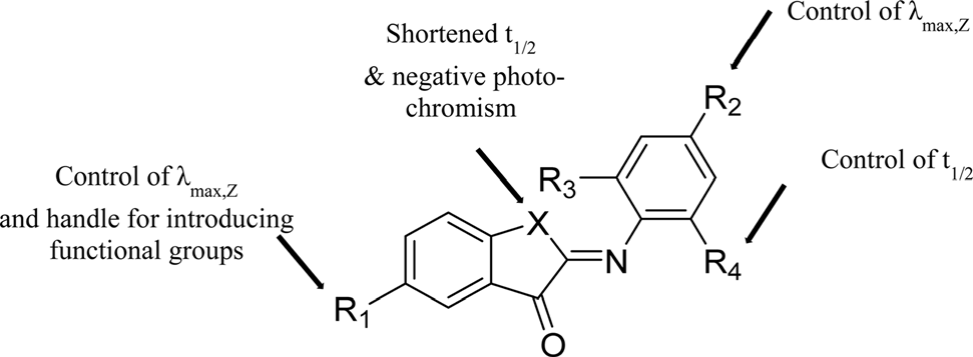
Structure–property relationships for ITI photoswitches, highlighting positions that can be selectively modified to tune key functional parameters, including the stability of isomers, absorption band positions and half-life of the metastable isomer.

We have designed a library of ITIs with increased thermal stabilities and red shifted absorption bands. Spectroscopic analyses showed that changing the aromatic imine part of the ITI photochrome is most useful for independently tuning the photophysical features of ITIs (Fig. 3) with the *para*-position (R_2_) dominantly influencing the absorption maximum and the *ortho*-positions (R_3_ & R_4_) giving control over the thermal stability of the metastable *E*-isomer. Both computational and spectroscopic analyses have shown that substitution on the thioindoxyl moiety at R_1_ can be used for introducing functional groups without affecting spectrochemical properties too much, provided that R_1_ is not too strongly electron-donating.

Based on computational data, we have been able to identify the most advantageous substituents for increasing the thermal stability of the *E*-isomer, with the overall conclusion that the higher the charge imbalance (*i.e.* polarization) between the thioindoxyl and phenyl moieties introduced by substituents is, the higher the thermal stability is. This has enabled us to come to the rational design of a perfluoro-ITI (1z) with a half-life of nearly 0.2 seconds, which is in the range aimed for in photopharmacological applications.

Compound 1z has been shown to undergo an S_N_Ar reaction with ethanol and l-cysteine, demonstrating its usefulness for introducing nucleophilic functionalities or linkers, or for protein labeling. It is also a favorable starting point for improving the solubility of ITIs in aqueous media at concentrations relevant for (bio)medical applications. Due to their solubility in various media, these ITIs can also be used for other applications that require fast responses, such as optical data writing, super-resolution imaging, or optical control of ion channels. Substitution of the sulfur atom by a larger atom or bulkier group has been shown to pave the way for designing ITIs with an even shorter half-live or negative photochromism.

Our studies have demonstrated that the ITI scaffold is a unique starting point for tailoring the spectroscopic and photophysical properties of ITI-based compounds to the application at hand. The analyzed molecules thereby break new ground for using small, visible-light-responsive tools for a variety of applications that require half-lives in the millisecond to sub-second range.

## Author contributions

M. E. B., M. W. H. H. and W. S. conceived the project and designed the molecules. All calculations were performed by M. M., M. O. and A. D. L., M. E. B., M. W. H. H. and A. C. A. W. performed the synthesis. Nanosecond TA spectroscopy was performed by M. E. B., M. W. H. H., M. F. H. and W. J. B. UV-vis experiments were performed by M. E. B., M. W. H. H. and A. C. A. W. The manuscript was written by M. E. B., M. M., M. O., A. D. L., M. D. D., W. J. B. and W. S. The research was supervised by W. S., M. M., W. J. B., M. D. D. and B. L. F. All authors discussed the results and progress in all stages.

## Conflicts of interest

The authors declare no conflict of interest.

## Supplementary Material

SC-015-D4SC01457J-s001

SC-015-D4SC01457J-s002

## Data Availability

The data supporting this article have been included as part of the ESI.†
