# Supplementary material for: Getting a molecular grip on the half-lives of iminothioindoxyl photoswitches
Source: Chem Sci. 2024 Jul 26;15(35):14379–89. doi: 10.1039/d4sc01457j (PMC11331343; doi:10.1039/d4sc01457j)
Supplement: SC-015-D4SC01457J-s001 [file SC-015-D4SC01457J-s001.pdf]

Supplementary Information (SI) for Chemical Science.
This journal is © The Royal Society of Chemistry 2024

Getting a molecular grip on the half-lives of iminothioindoxyl photoswitches

Melody E. Boëtius, Mark W.H. Hoorens, Martin Ošťadnický, Adèle Laurent, Mariangela di Donato, Aldo C. A. van Wingaarden, Michiel F. Hilbers, Ben L. Feringa, Wybren Jan Buma,* Miroslav Medveď,* Wiktor Szymanski*

Table of Contents

S1. General methods.....3

S1.1. Organic synthesis.....3

S1.2. Analytical procedures.....3

S1.3. Computational details.....3

S1.4. Nanosecond transient absorption spectroscopy.....4

S1.5. Preparative HPLC analysis & purification.....5

S2. Experimental Procedures.....5

S2.1. Synthetic procedures.....5

S2.2. Stability tests of 1z and 1β.....70

S3. Nanosecond transient absorption spectra.....72

S4. Computational analysis.....98

S5. Quantum yield calculations.....135

S6. References.....136

S1. General methods

S1.1. Organic synthesis.

All reported starting materials, chemical reagents and organic solvents in this study were bought from Sigma–Aldrich, Enamine, Acros, Fluka, Fischer Scientific, TCI and were used as received. Dry DCM was purified by passage through an MBraun SPS-800 solvent purification column. All aqueous solutions were prepared using deionized water. Kieselgel 60, F254 silica gel plates (Merck, TLC silica gel 60 F254) were used for TLC (Thin Layer Chromatography) analysis and UV light of 254 nm and potassium permanganate solution ($KMnO_4$) were used for the detection of compounds. Drying of solutions was performed using dry $MgSO_4$ and solvents and other volatiles were removed using a rotary evaporator.

Compounds were purified with a Buchi Pure C-815 Flash Chromatography instrument with EcoFlex Silica 50 µm irregular columns of 4 g or 12 g.

S1.2. Analytical procedures.

Nuclear Magnetic Resonance (NMR) spectra were recorded using an Agilent Technologies 400-MR (400/54 Premium Shielded) spectrometer (400 MHz), at room temperature (22–24 °C), unless indicated otherwise. The multiplicities of the signals are reported as follows: s (singlet), d (doublet), t (triplet), app td (apparent triplet of doublets), q (quartet) or m (multiplet). All ^{13}C-NMR spectra are 1H-broadband decoupled.

Melting points (Mp) were measured using a Stuart analogue capillary melting point SMP11 apparatus. Infrared (IR) spectra were measured on a Shimadzu IRSpirit-LX FTIR spectrophotometer. The major peaks are reported in cm^{-1}. High-resolution mass spectrometric (HRMS) measurements were performed using a Thermo scientific LTQ OrbitrapXL spectrometer, which is equipped with ESI ionization. In the experimental procedures, the mass of the molecule-ion $[M+H]^+$ are reported in m/z-units. Absorption spectra were measured using an Agilent 8453 UV/Vis diode array. All solutions for absorption spectra were prepared in Uvasol® grade solvents and were measured in quartz cuvettes with a 1 cm path-length.

Purity was determined using LCMS, for which the following setup was used: Column: ACQUITY UPLC® HSS T3 1.8 µm, 2.1 × 150 mm. Detection program **1**: Total Ion Count (TIC), λ_1 = 254 nm, λ_2 = 430 nm; Detection program **2**: Total Ion Count (TIC), λ_1 = 254 nm, λ_2 = 407 nm, λ_2 = 500 nm. For both programs: Flow: 0.3 mL/min; Eluent A: 0.1% formic acid in ULC/MS-CC/SFC-grade water; Eluent B: 0.1% formic acid in LC-MS-grade acetonitrile. Gradient Program **1**: (0–1 min) 5% eluent B; (1–8 min) linear gradient to 90% eluent B; (8–11 min) 90% eluent B; (11–12 min) linear gradient to 5% eluent B; (12–17 min) 5% eluent B. Gradient program **2**: (0-8.5 min, pre-program) 10% eluent B; (8.5-14.5 min) linear gradient to 70% eluent B; (14.5-16.5 min) linear gradient to 90% eluent B; (16.5-18 min) linear gradient to 100% eluent B; (18-23 min) 100% gradient B.

S1.3. Computational details

All systems were theoretically investigated using a composite DFT approach, employing the M06-2X functional[1], in combination with the 6-31+G(d) and 6-311++G(2df,2p) basis sets[2]. For compounds containing bromine, the cc-pVTZ basis set[3] was used for light atoms and the scalar relativistic effects were included by using the effective core potential cc-pVTZ-PP basis set for bromine[4] as well as the all-electron 2nd order Douglas-Kroll-Hess (DKH2) approach[5-8] in combination with the cc-pVTZ-DK basis set.[9-11] Geometry optimizations and the subsequent frequency analysis rendering the thermal

contributions to the Gibbs energy were performed employing the smaller double-ζ basis set. For the geometry optimizations, tight convergence criteria (*opt=tight* keyword in G16) were applied. Transition state (TS) structures were checked against the presence of a single imaginary frequency. For the optimized structures, electronic energies were computed using the larger triple-ζ basis set. Solvent effects were taken into consideration by employing the implicit solvation model based on density (SMD).[12] To account for the solvent effects on electronic transitions, vertical excitation energies (VEEs) were computed using the non-equilibrium corrected linear response scheme[13] referred to as cLR^2 approach which includes the dynamical response of the solvent to the solute transition density (so-called excited state (ES) dispersion contribution captured by the linear-response (LR) approach) as well as a perturbative correction of the state-specific polarization of the solvent due to the ES density (corrected linear response, cLR).[14] This approach was shown to be superior to both LR and cLR for various types of transitions.[13]
All computations were performed using the Gaussian09[15] and Gaussian16[16] programs.

S1.4. Nanosecond transient absorption spectroscopy.

Nanosecond transient absorptions were recorded with an in-house assembled setup. For all ITIs and all solvents, an excitation wavelength of either 420 or 430 nm was used. This wavelength was generated using a tunable Nd:YAG-laser system (NT342B, Ekspla) comprising the pump laser (NL300) with harmonics generators (SHG, THG) producing 355 nm to pump an optical parametric oscillator (OPO) with SHG connected in a single device. Normally, the laser system was operated at a repetition rate of 5 Hz while the probe light, generated by a high-stability short arc xenon flash lamp (FX-1160, Excelitas Technologies) using a modified PS302 controller (EG&G), was operated at 10 Hz. However, for compounds for which the half-life was too long, appropriate lower repetition rates were employed. Using a 50/50 beam splitter, the probe light was split equally into a signal beam and a reference beam with and focused on the entrance slit of a spectrograph (SpectraPro-150, Princeton Instruments). The probe beam (A = 1 mm2) was passed through the sample cell and orthogonally overlapped with the excitation beam on a 1 mm × 1 cm area. The excitation energy was recorded by measuring the excitation power at the back of an empty sample holder. In order to correct for fluctuations in the flash lamp spectral intensity, the reference was used to normalize the signal. Both beams were recorded simultaneously using a gated intensified CCD camera (PI-MAX3, Princeton Instruments) which has an adjustable gate of minimal 2.9 ns.

A delay generator (DG535, Stanford Research Systems, Inc.) was used to time the excitation pulse, the flash lamp, and the gate of the camera. The setup was controlled by an in-house written LabView program.

Transient absorption spectra were globally analyzed, that is, fitting the time evolution of the data at all wavelengths simultaneously, using the Glotaran software.[17] Such analyses showed that the data could well be fitted with a single exponential decay function. For some compounds a slightly better fit could be obtained by adding a component with an -on the timescale of the experiment- infinitely long decay time. This component, however, only had a very minor contribution and gave rise to changes in the decay time obtained with a single-exponential fit that were within the error margin given for the decay time. We attribute this second component to an incomplete thermal back-isomerization reaction on the timescale of the experiment, or due to diffusion.

S1.5. Preparative HPLC analysis & purification

Preparative HPLC analysis was performed on a Shimadzu HPLC system with an XTerra® MS C18 3.5 µm 3.0x150mm column. Flow rate: 0.350 mL/min. Eluent A: Milli-Q-grade water with 0.14% triethyl amine and 0.057% acetic acid; Eluent B: HPLC-grade acetonitrile. Gradient program: (0-5 min) 5% eluent B, (5-15 min) linear gradient to 80% eluent B, (15-19 min) 80% eluent B, (19-20 min) linear gradient to 5% eluent B, (20-40 min) 5% eluent B.

Preparative HPLC purification was performed on a Shimadzu HPLC system with a Phenomenex® Kinetex 5 µm EVO C18 100 Å column. Wavelengths used: λ_1 = 407 nm, λ_2 = 500 nm.
Flow rate: 45 mL/min. Eluent A: Milli-Q-grade water; Eluent B: HPLC-grade acetonitrile. Gradient program: (0-5 min) 10% eluent B, (5-25 min) linear gradient to 90% eluent B, (25-30 min) 90% eluent B, (30-32 min) linear gradient to 10% eluent B, (32-37 min) 10% eluent B.

S2. Experimental procedures

S2.1. Organic synthesis.

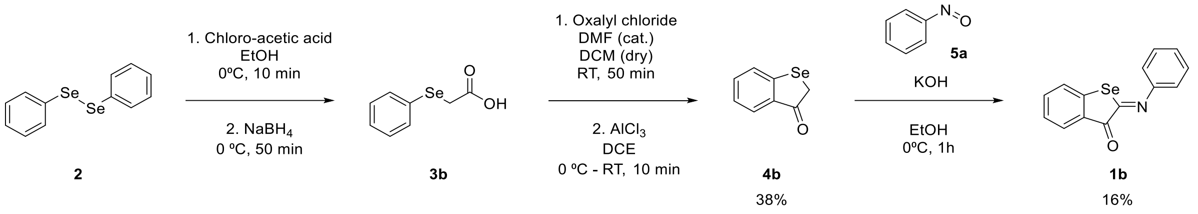

Scheme S1 Synthesis of **1b**.

3b: 2-(Phenylselanyl)acetic acid

1,2-Diphenyldiselane **2** (1.0 g, 3.3 mmol, 1.0 eq) and chloro-acetic acid (0.16 g, 1.7 mmol, 0.52 eq) were added to EtOH (30 mL), and the reaction mixture was stirred at 0 °C until the reactants fully dissolved. $NaBH_4$ was added portion-wise until the yellow reaction mixture became colorless. The reaction mixture was then stirred for 75 min at room temperature under nitrogen atmosphere. White precipitate was formed in the reaction mixture, which was separated through filtration and washed with pentane. The product was obtained as a white solid (0.53 g, 2.46 mmol, 38 % yield). Mp: > 250 °C. ^{1}H NMR (400 MHz, DMSO-d6) δ 7.38 (d, J = 8.1 Hz, 2H, Ar**H**), 7.19 (t, J = 7.4 Hz, 2H, Ar**H**), 7.11 (t, J = 7.3 Hz, 1H, Ar**H**), 3.48 (s, 2H, C**H**$_2$).

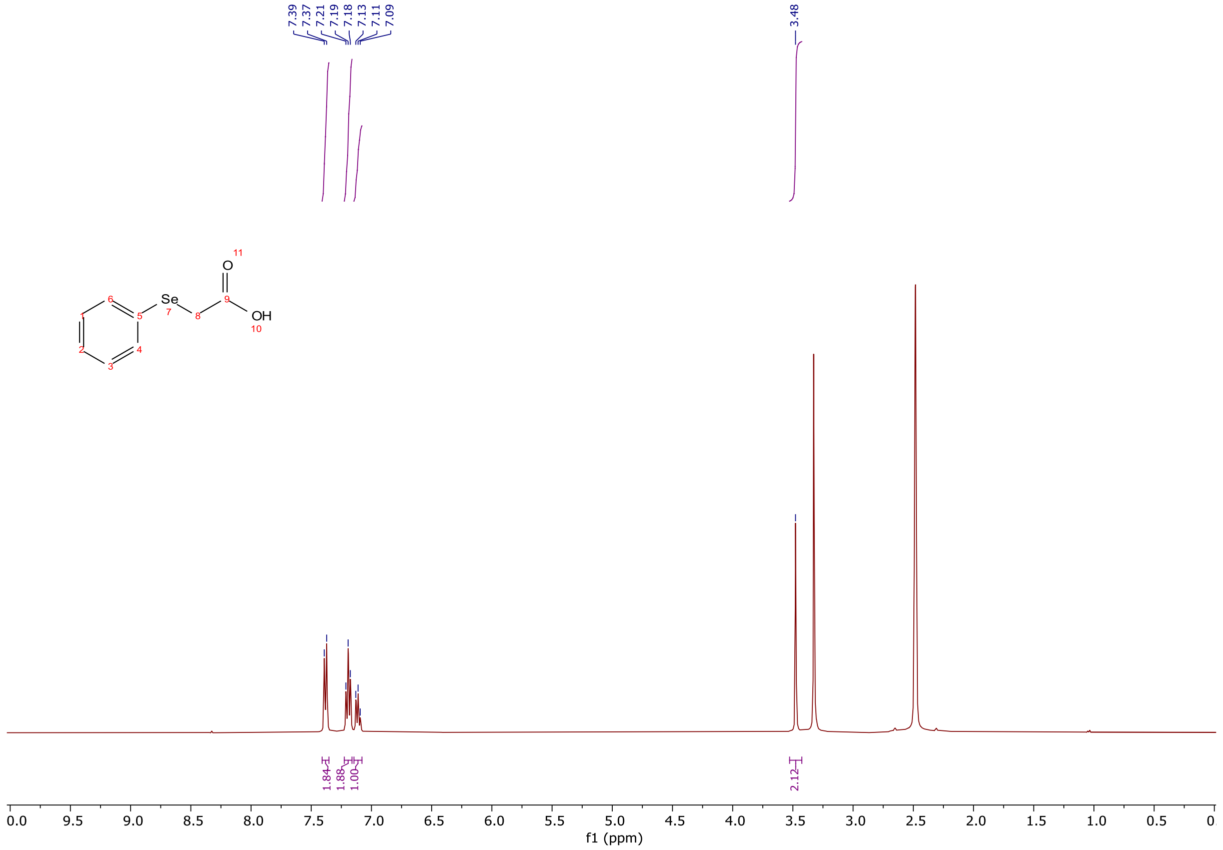

Figure S1 ^{1}H NMR spectrum of compound **3b** in $CDCl_3$.

4b: benzo[b]selenophen-3(2H)-one

2-(Phenylselanyl)acetic acid **3b** (0.25 g, 1.2 mmol, 1.0 eq) was dissolved in DCM (5 mL, dry) and oxalyl chloride (0.3 mL, 3.5 mmol, 2.9 eq) and 1 drop of DMF were added. The reaction mixture was stirred until gas evolution stopped (50 min). The reaction mixture was concentrated *in vacuo* and the remaining oil was redissolved in dichloroethane (5 mL) and cooled on an ice-water bath. $AlCl_3$ (0.52 g, 3.9 mmol) was added portion-wise, and the mixture was stirred for 10 minutes. After completion, DCM (50 mL) and H_2O (50 mL) were added, and the layers were separated. The aqueous layer was extracted with DCM (3 x 50 mL). The combined organic layers were washed with water (25 mL) and brine (25 mL), dried over $MgSO_4$, and concentrated *in vacuo*. The crude product was used without further purification and directly used in the next step to prevent degradation.

1b: (*Z*)-2-(phenylimino)benzo[b]selenophen-3(2H)-one

Crude benzo[b]selenophen-3(2H)-one **4b** was dissolved in benzene (5 mL), after which nitrosobenzene **5a** (0.33 g, 3.0 mmol) and 1 drop of piperidine were added. The reaction mixture was stirred vigorously at room temperature under nitrogen atmosphere. After 2 h, TLC indicated complete consumption of the starting material. Then, DCM (50 mL) and H_2O (50 mL) were added, and the layers were separated. The aqueous layer was extracted with DCM (3 x 50 mL). The organic layers were combined and washed with sat. aq. $NaHCO_3$ (25 mL) and brine (25 mL), dried over $MgSO_4$, and concentrated *in vacuo*. The product was purified by flash chromatography (Silicagel 40 – 63 nm, 0-6% Et_2O in pentane). The product was obtained as a brown solid (53 mg, 0.19 mmol, 16 % yield over 2 steps). Mp: 120-122 °C. ^1H NMR (400 MHz, $CDCl_3$) δ 7.95 (d, J = 7.7 Hz, 1H, Ar**H**), 7.58 (t, J = 7.5 Hz, 1H, Ar**H**), 7.46 (m, 3H, Ar**H**), 7.32 (m, 2H, Ar**H**), 7.21 (d, J = 7.8 Hz, 2H, Ar**H**). ^{13}C NMR (101 MHz, $CDCl_3$) δ 188.2, 158.9, 151.2, 140.7,

137.0, 129.5, 129.0, 128.1, 127.4, 127.1, 119.8. HRMS (ESI+) calc. for $[M+H]^+$ ($C_{14}H_{10}NOSe^+$) 287.9922, found: 287.9921.

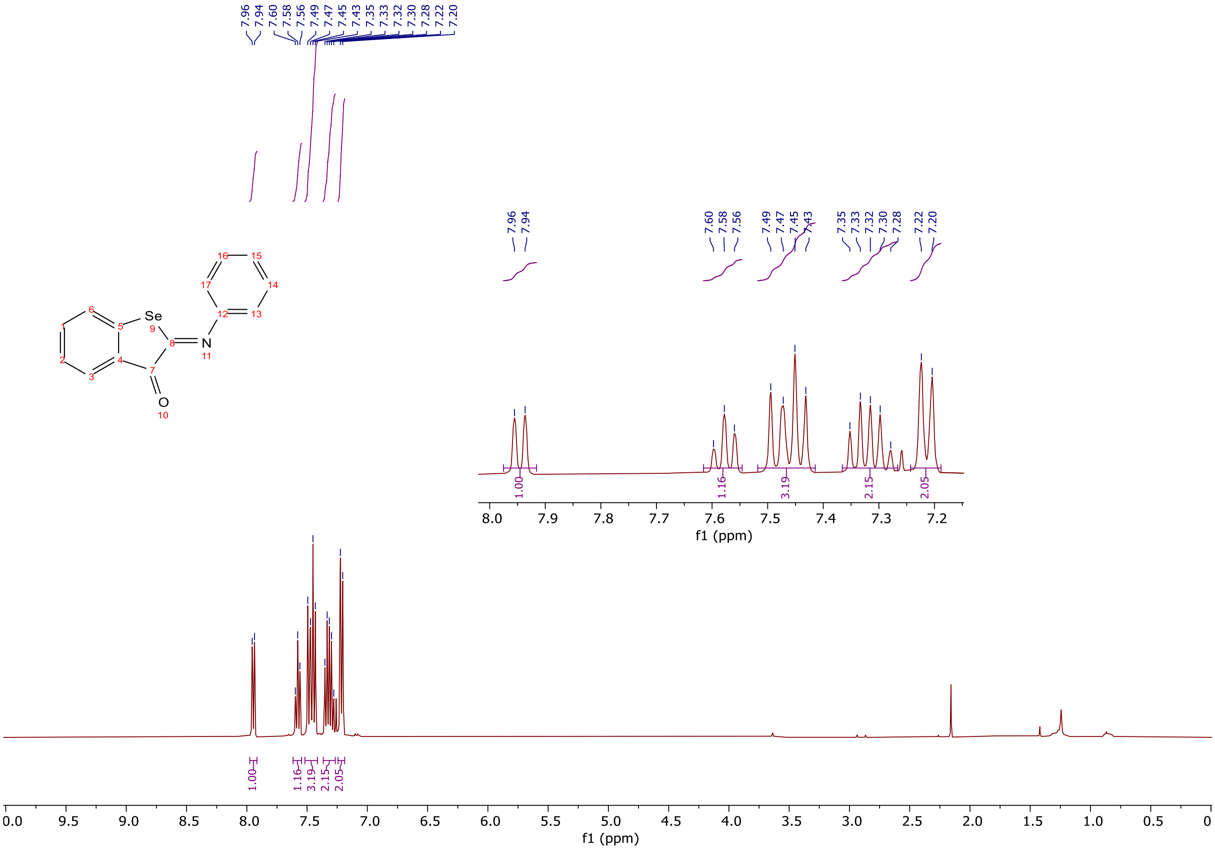

Figure S2 ^{1}H NMR spectrum of compound **1b** in $CDCl_3$.

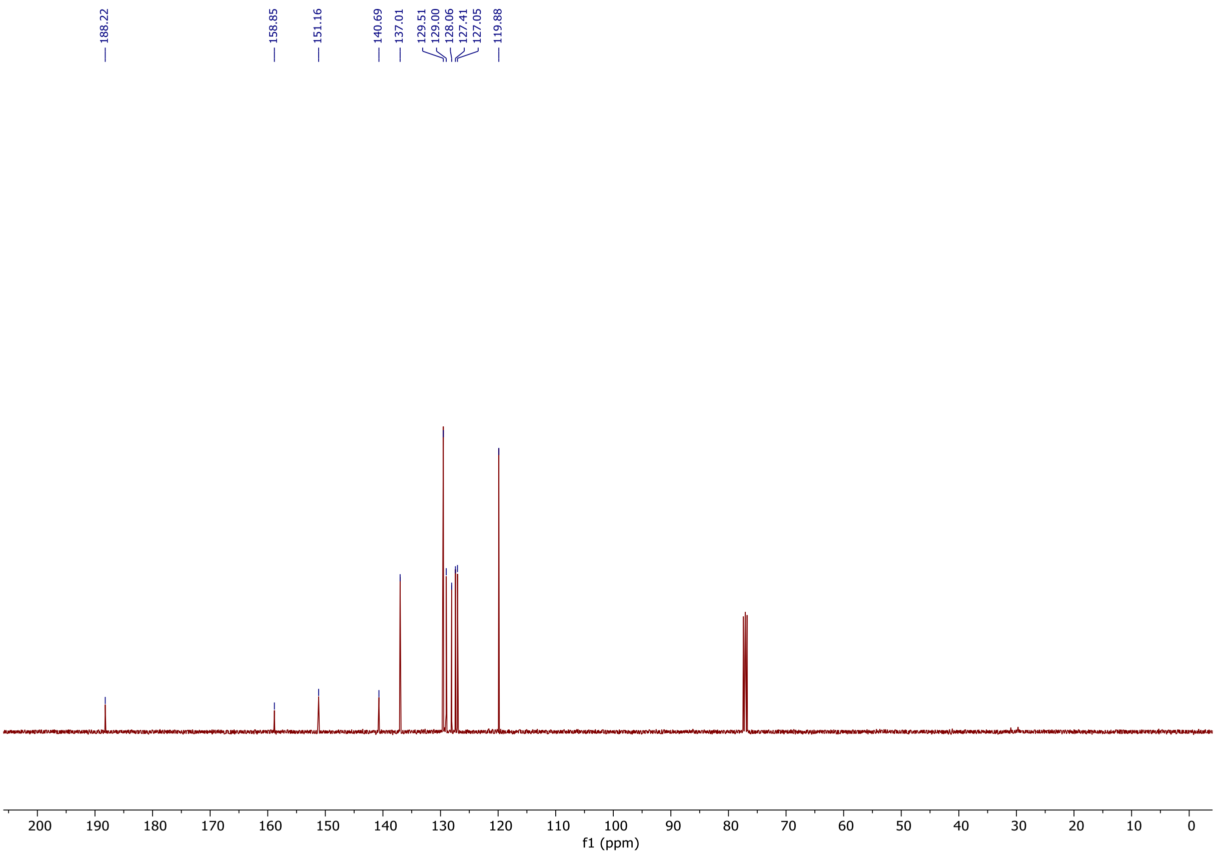

Figure S3 ^{13}C NMR spectrum of compound **1b** in $CDCl_3$.

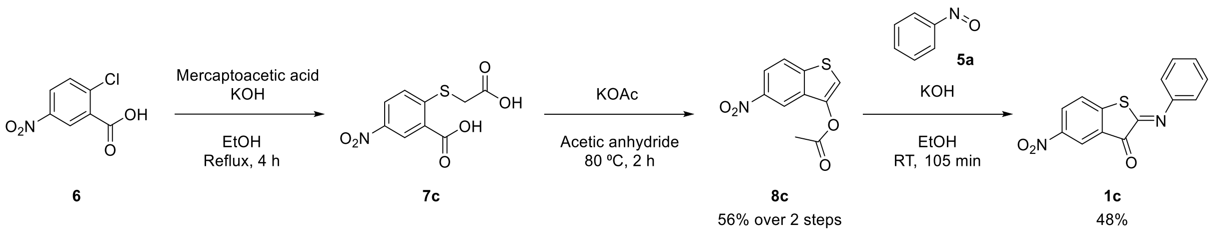

Scheme S2 Synthesis of **1c**.

7c: 2-((carboxymethyl)thio)-5-nitrobenzoic acid

2-Chloro-5-nitrobenzoic acid **6** (0.25 g, 1.2 mmol, 1.0 eq) was dissolved in EtOH (5 mL) and mercaptoacetic acid (85 µL, 1.2 mmol, 1.0 eq) and KOH (0.30 g, 5.3 mmol, 4.4 eq) were added. The reaction mixture was heated under reflux and stirred vigorously. After 4 h, TLC indicated complete consumption of the starting material. Then, DCM (50 mL) and aq. 1 N HCl (50 mL) were added, and the layers were separated. The aqueous layer was extracted with DCM (3 x 50 mL). The combined organic layers were washed with brine (25 mL), dried over $MgSO_4$, and concentrated *in vacuo*. The product was obtained as a yellow solid and was used without further purification.

8c: 5-nitrobenzo[b]thiophen-3-yl acetate

Crude 2-((carboxymethyl)thio)-5-nitrobenzoic acid **2c** (0.11 g, 0.43 mmol, 1.0 eq) was dissolved in acetic anhydride (1 mL) and KOAc (0.12 g, 1.2 mmol, 2.8 eq) was added. The reaction mixture was stirred vigorously at 80 °C. After 2 h, TLC indicated complete consumption of the starting material.

Then, EtOAc (50 mL) and H_2O (50 mL) were added, and the layers separated. The aqueous layer was extracted with EtOAc (3 x 50 mL), and the combined organic layers were washed with brine (25 mL), dried over $MgSO_4$, and concentrated *in vacuo*. The product was purified by flash chromatography (Silicagel 40 – 63 nm, DCM). The product was obtained as a white solid (70 mg, 0.30 mmol, 56% yield over two steps). Mp: 143 – 145 °C. ^{1}H NMR (400 MHz, $CDCl_3$) δ 8.58 (s, 1H, Ar**H** (3)), 8.21 (d, J = 8.9 Hz, 1H, Ar**H** (1)), 7.90 (d, J = 8.9 Hz, 1H, Ar**H** (6)), 7.62 (s, 1H, C=C**H** (8)), 2.44 (s, 3H, C**H**$_3$ (13)). ^{13}C NMR (101 MHz, $CDCl_3$) δ 167.8, 145.5, 142.2, 141.2, 132.1, 123.6, 119.4, 116.6, 115.1, 21.0. HRMS (ESI+) calc. for $[M+Na]^+$ ($C_{10}H_7NO_4SNa^+$) 259.9993, found: 259.9988.

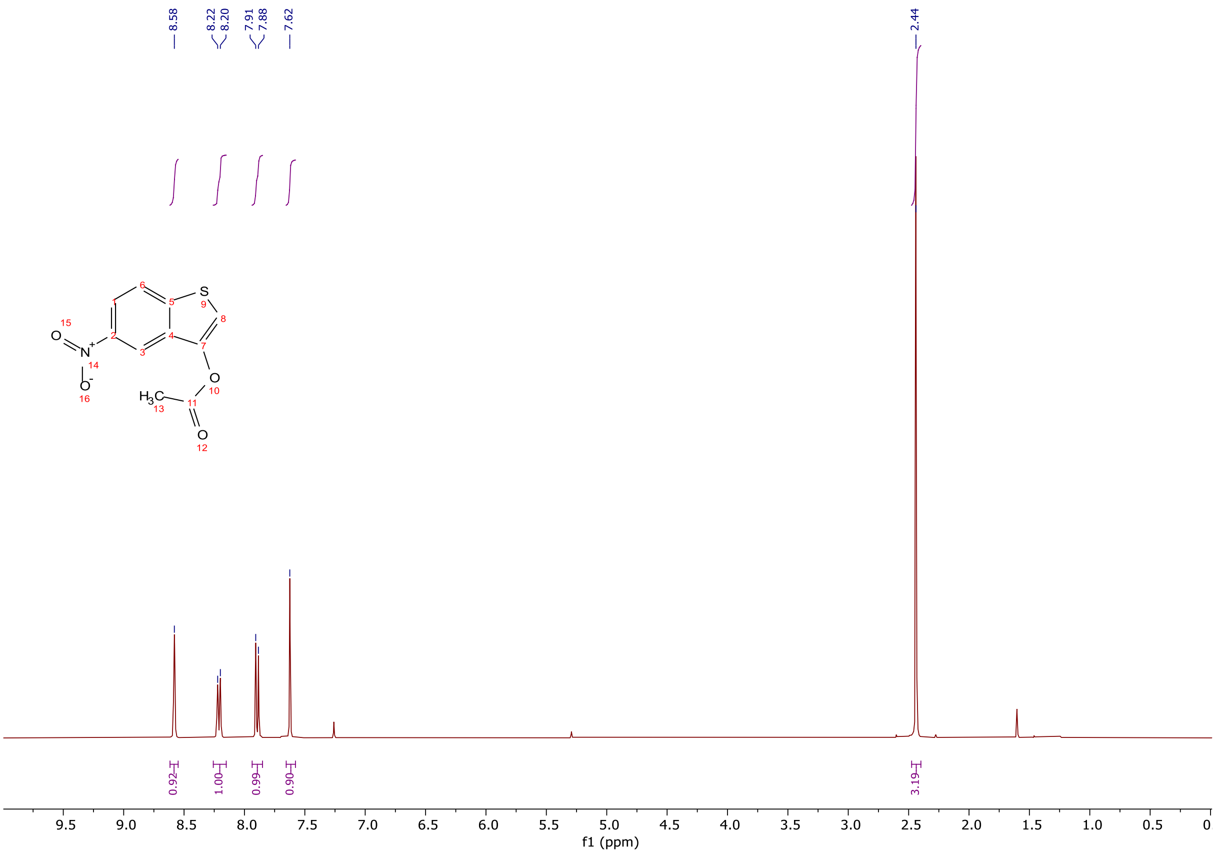

Figure S4 ^{1}H NMR spectrum of compound **8c** in $CDCl_3$.

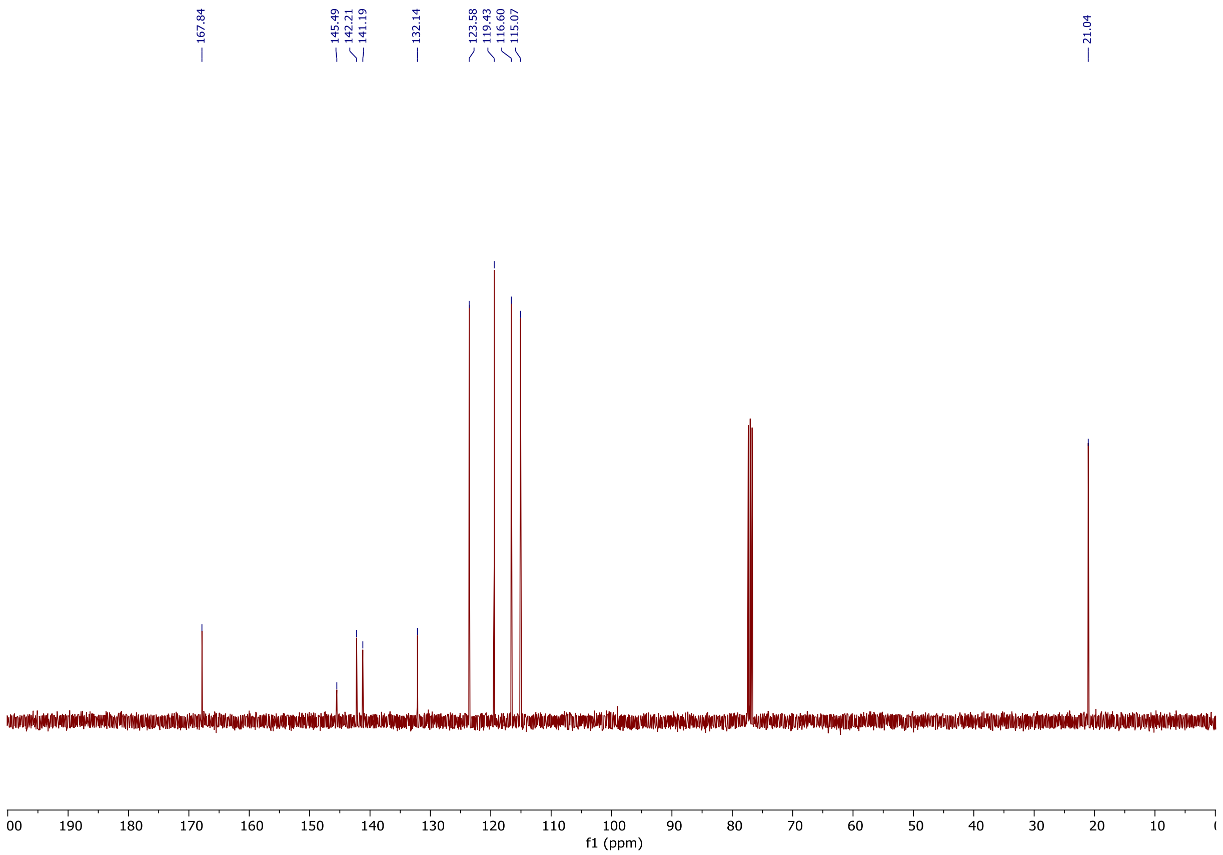

Figure S5 ^{13}C NMR spectrum of compound **8c** in $CDCl_3$.

1c: (*Z*)-5-nitro-2-(phenylimino)benzo[b]thiophen-3(2H)-one

5-Nitrobenzo[b]thiophen-3-yl acetate **3c** (51 mg, 0.21 mmol) was dissolved in EtOH (2 mL) and nitrosobenzene **5a** (50 mg, 0.48 mmol) and 12 drops of a KOH solution (25 mg/mL in EtOH) were added. The reaction mixture was stirred vigorously at room temperature. After 105 min, TLC indicated complete consumption of the starting material. Then, DCM (50 mL) and H_2O (50 mL) were added, and the layers were separated. The aqueous layer was extracted with DCM (3 x 50 mL) and the combined organic layers were washed with water (25 mL) and brine (25 mL), dried over $MgSO_4$, and concentrated *in vacuo*. The product was purified by precipitation from EtOAc upon the addition of pentane. The product was obtained as dark yellow needle-shaped crystals (29 mg, 0.1 mmol, 48 % yield). Mp: 193 – 195 °C. ^{1}H NMR (400 MHz, $CDCl_3$) δ 8.79 (d, J = 2.3 Hz, 1H, Ar**H** (3)), 8.49 (dd, J = 8.6, 2.3 Hz, 1H, Ar**H** (1)), 7.62 (d, J = 8.6 Hz, 1H, Ar**H** (6)), 7.50 (t, J = 7.8 Hz, 2H, Ar**H** (14 and 16)), 7.36 (t, J = 7.4 Hz, 1H, Ar**H** (15)), 7.29 (d, J = 8.2 Hz, 2H, Ar**H** (13 and 17)). ^{13}C NMR (101 MHz, $CDCl_3$) δ 183.4, 153.9, 151.6, 148.6, 146.8, 130.8, 129.6, 128.3, 128.2, 125.7, 122.6, 121.2. HRMS (ESI+) calc. for $[M+H]^+$ ($C_{14}H_8N_2O_3S$) 285.0328, found: 285.0326.

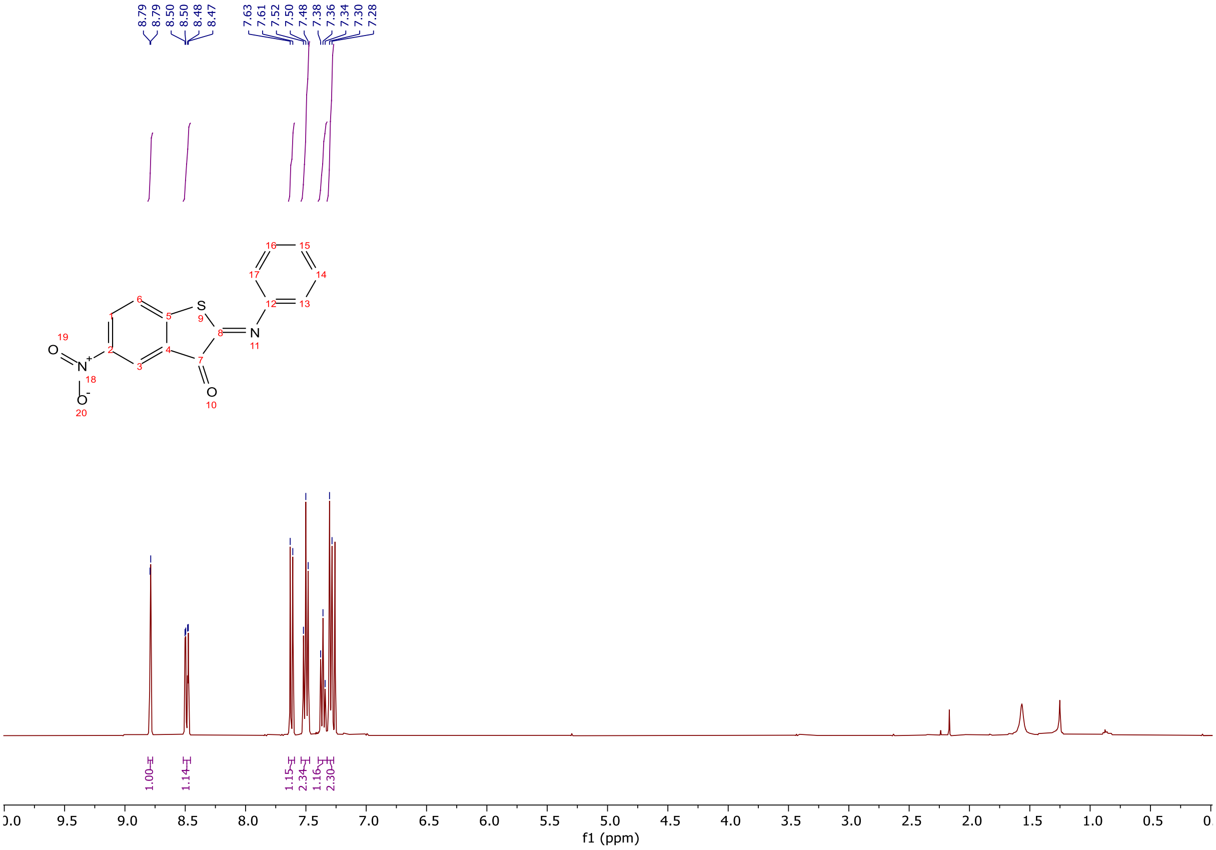

Figure S6 ^{1}H NMR spectrum of compound **1c** in $CDCl_3$.

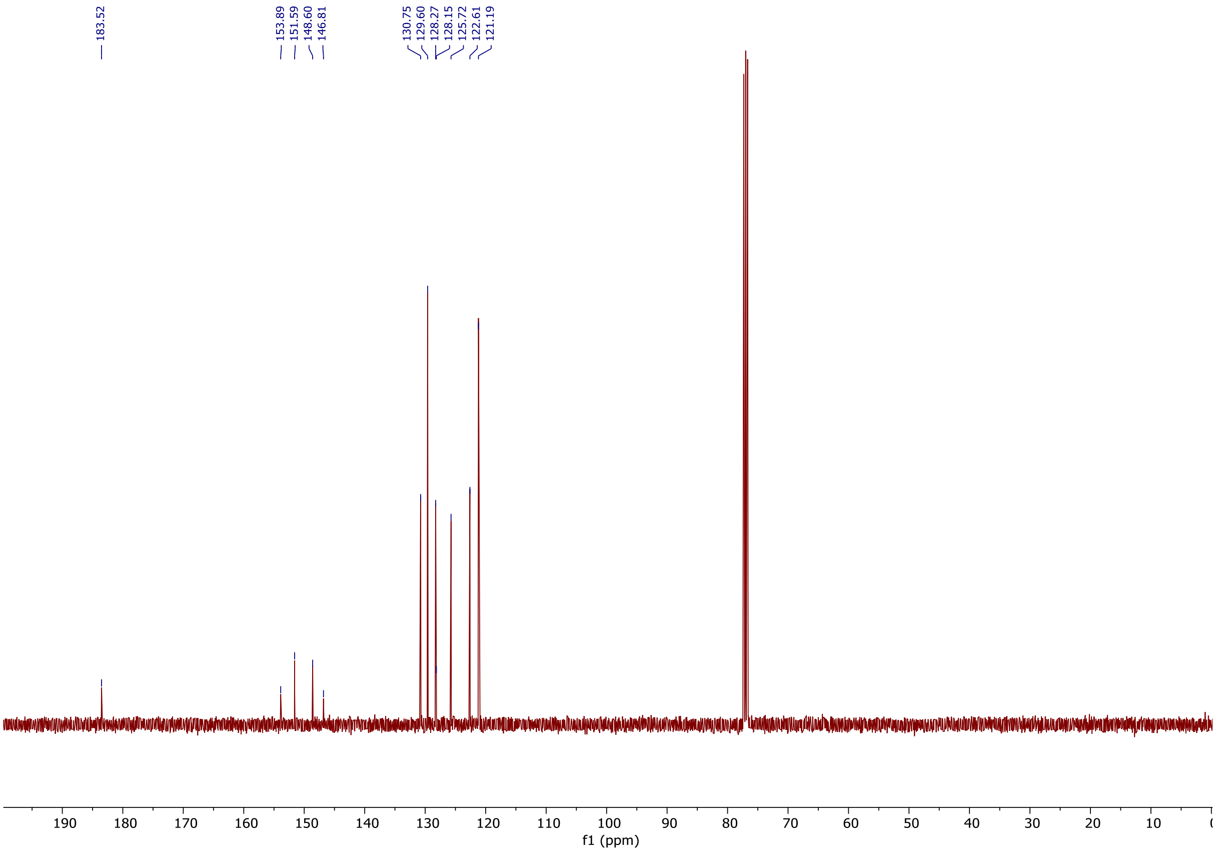

Figure S7 ^{13}C NMR spectrum of compound **1c** in $CDCl_3$.

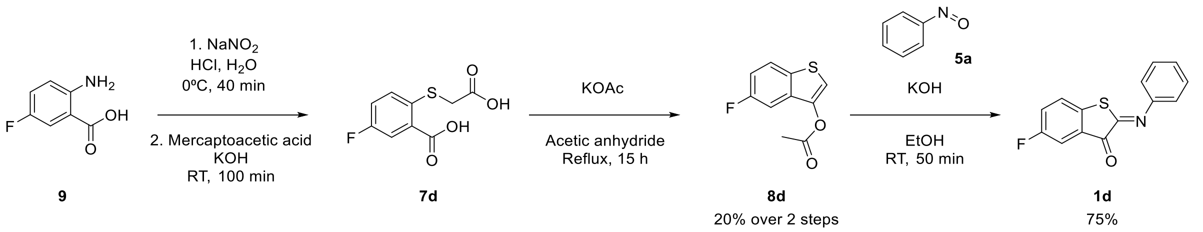

Scheme S3 Synthesis of **1d**.

7d: 2-((carboxymethyl)thio)-5-fluorobenzoic acid

2-Amino-5-fluorobenzoic acid **9** (1.0 g, 6.5 mmol, 1.0 eq) was dissolved in aq. 1 N HCl (25 mL) and cooled on an ice-water bath. $NaNO_2$ (0.74 g, 11 mmol, 1.6 eq) was added portion-wise and the solution was stirred on an ice-bath for 40 minutes. A solution of mercaptoacetic acid (0.6 mL, 8.7 mmol, 1.3 eq) and KOH (1.9 g, 33 mmol, 5.1 eq) in H_2O (10 mL) was added drop-wise, and the reaction mixture was stirred vigorously at room temperature. After 100 min, TLC indicated complete consumption of the starting material. Then, DCM (50 mL) and aq. 1 N HCl (50 mL) were added, and the layers were separated. The aqueous layer was washed with DCM (3 x 50 mL) and the combined organic layers were washed with brine (25 mL), dried over $MgSO_4$, and concentrated *in vacuo*. The crude product was used without further purification and characterization.

8d: 5-fluorobenzo[b]thiophen-3-yl acetate

Crude 2-((carboxymethyl)thio)-5-fluorobenzoic acid **7d** was dissolved in acetic anhydride (10 mL) and KOAc (1.0 g, 10 mmol) was added. The reaction mixture was heated under reflux and stirred vigorously. After 15 h, TLC indicated complete consumption of the starting material. Then, the reaction mixture was cooled to room temperature. Et_2O (50 mL) and H_2O (50 mL) were added, and the layers were separated. The aqueous layer was extracted with Et_2O (3 x 50 mL), and the combined organic layers were washed with water (25 mL) and brine (25 mL), dried over $MgSO_4$, and concentrated *in vacuo*. The product was purified by flash chromatography (Silicagel 40 – 63 nm, 0-25% Et_2O in pentane) The product was obtained as a colorless oil (0.27 g, 1.3 mmol, 20% yield over 2 steps). ^{1}H NMR (400 MHz, $CDCl_3$) δ 7.68 (dd, *J* = 8.8, 4.6 Hz, 1H, Ar**H** (3 or 6)), 7.47 (s, 1H, Ar**H** (3 or 6)), 7.35 (d, *J* = 9.1 Hz, 1H, C=C**H** (8)), 7.12 (t, *J* = 8.8 Hz, 1H, Ar**H** (1)), 2.36 (s, 3H, C**H**$_3$ (13)). ^{13}C NMR (101 MHz, $CDCl_3$) δ 168.1, 162.0, 159.6, 140.3 (d, *J* = 4.5 Hz), 132.2 (d, *J* = 1.5 Hz), 124.2 (d, *J* = 9.3 Hz), 114.3, 114.2 (d, *J* = 25.4 Hz), 106.2 (d, *J* = 24.2 Hz), 20.9. HRMS (ESI+) calc. for $[M+H]^+$ ($C_8H_6FOS^+$) 169.0118, found: 169.0115.

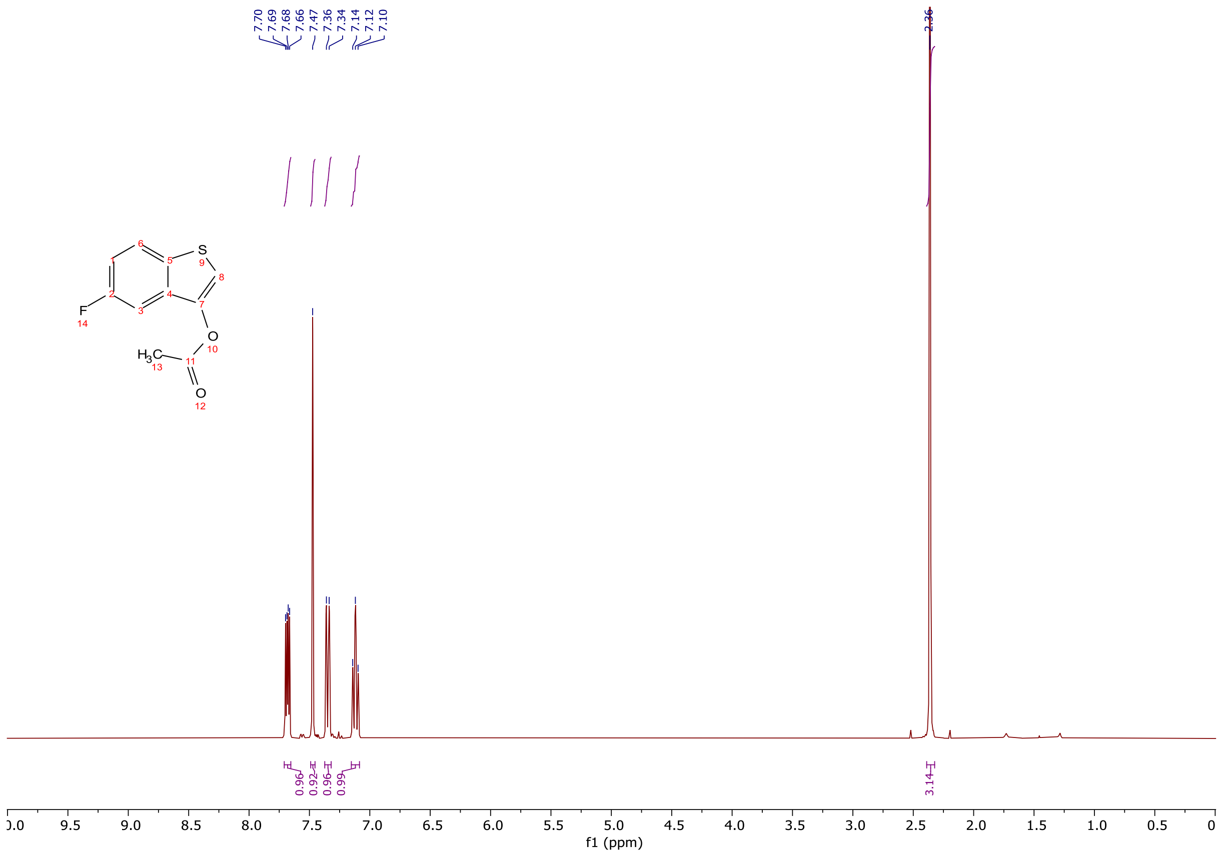

Figure S8 ^{1}H NMR spectrum of compound **8d** in $CDCl_3$.

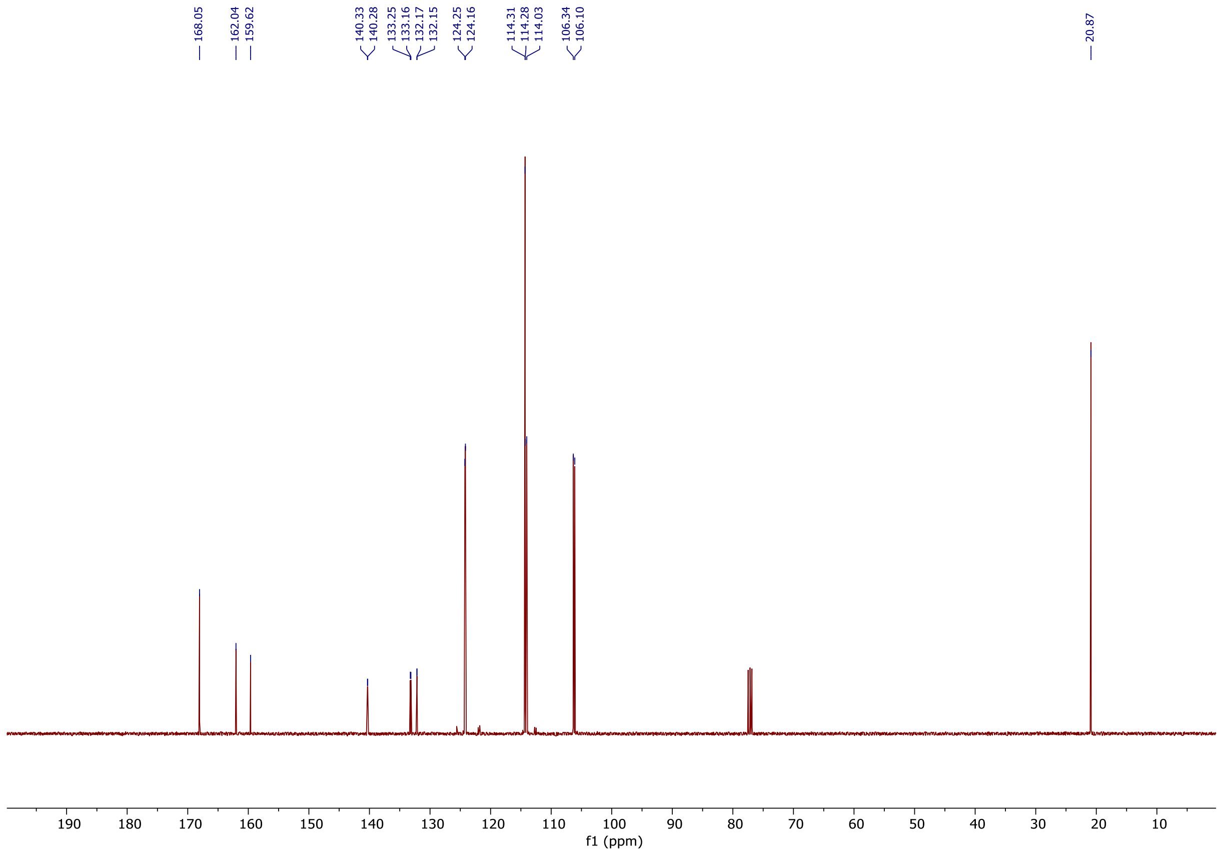

Figure S9 ^{13}C NMR spectrum of compound **8d** in $CDCl_3$.

1d: (*Z*)-5-fluoro-2-(phenylimino)benzo[b]thiophen-3(2H)-one

5-Fluorobenzo[b]thiophen-3-yl acetate **3d** (84 mg, 0.40 mmol, 1.0 eq) was dissolved in EtOH (4 mL). Nitrosobenzene **5a** (49 mg, 0.46 mmol, 1.2 eq) was added and the reaction mixture was cooled on an ice-bath. KOH (25 g/L in EtOH) was added dropwise (10 drops) and the mixture was allowed to reach room temperature and was stirred vigorously. After 50 min, TLC indicated complete consumption of the starting material. Then, Et_2O (50 mL) and H_2O (50 mL) were added, and the layers were separated. The aqueous layer was extracted with Et_2O (3 x 50 mL) and the combined organic layers were washed with water and brine, dried over $MgSO_4$ and concentrated *in vacuo*. The product was purified by flash chromatography (Silicagel 40 – 63 nm, toluene). The product was obtained as an orange solid (77 mg, 0.30 mmol, 75 % yield). Mp: 146 – 148 °C. ^{1}H NMR (400 MHz, $CDCl_3$) δ 7.65 (d, *J* = 6.3 Hz, 1H, Ar**H** (3)), 7.46 (t, *J* = 7.7 Hz, 2H, Ar**H** (14 and 16)), 7.37 (d, *J* = 5.4 Hz, 2H, Ar**H** (1 and 6)), 7.31 (t, *J* = 7.5 Hz, 1H, Ar**H** (15)), 7.27 (d, *J* = 7.9 Hz, 2H, Ar**H** (13 and 17)). ^{13}C NMR (101 MHz, $CDCl_3$) δ 184.8, 162.8, 160.3, 156.3, 149.1, 139.5 (d, *J* = 2.6 Hz), 129.4, 127.7, 126.3 (d, *J* = 7.3 Hz), 124.6 (d, *J* = 23.7 Hz), 121.2, 114.2 (d, *J* = 23.5 Hz). HRMS (ESI+) calc. for $[M+H]^+$ ($C_{14}H_9FNOS^+$) 258.0383, found: 258.0386.

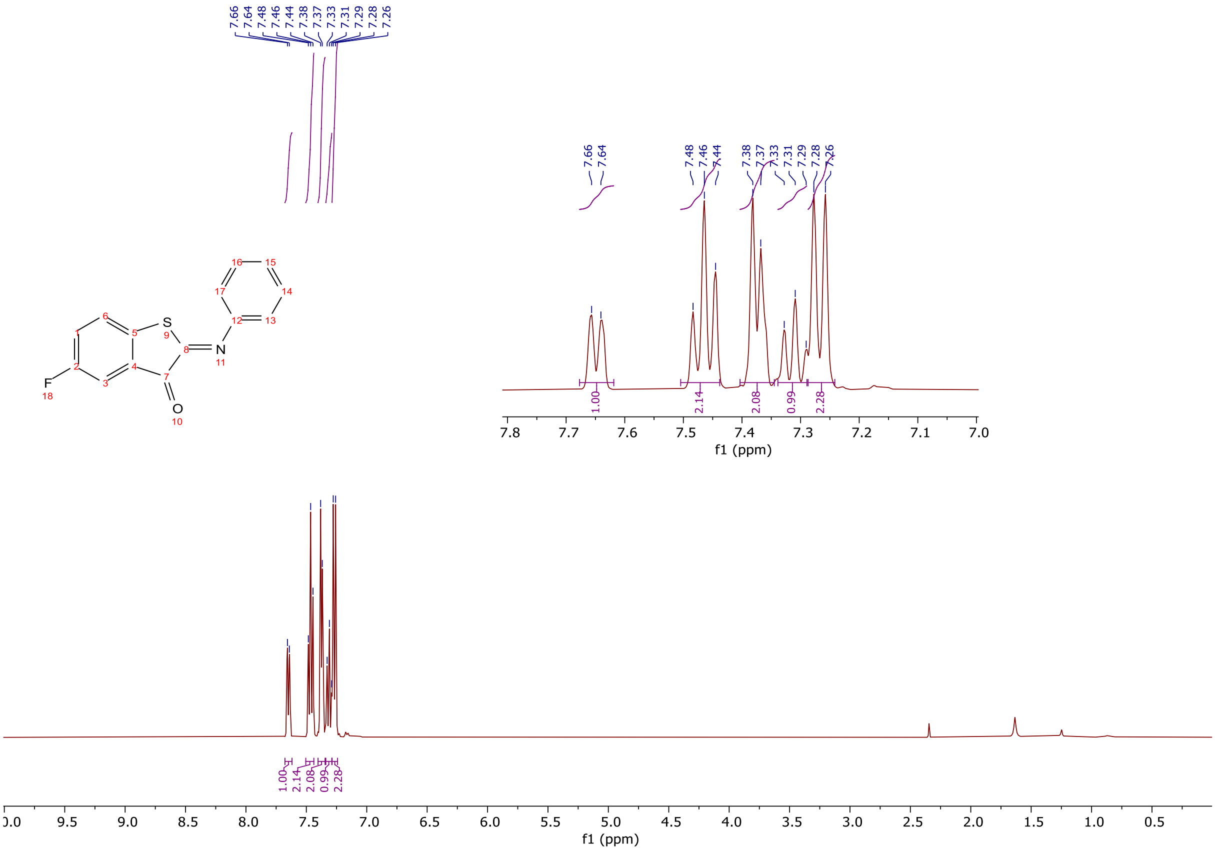

Figure S10 ^{1}H NMR spectrum of compound **1d** in $CDCl_3$.

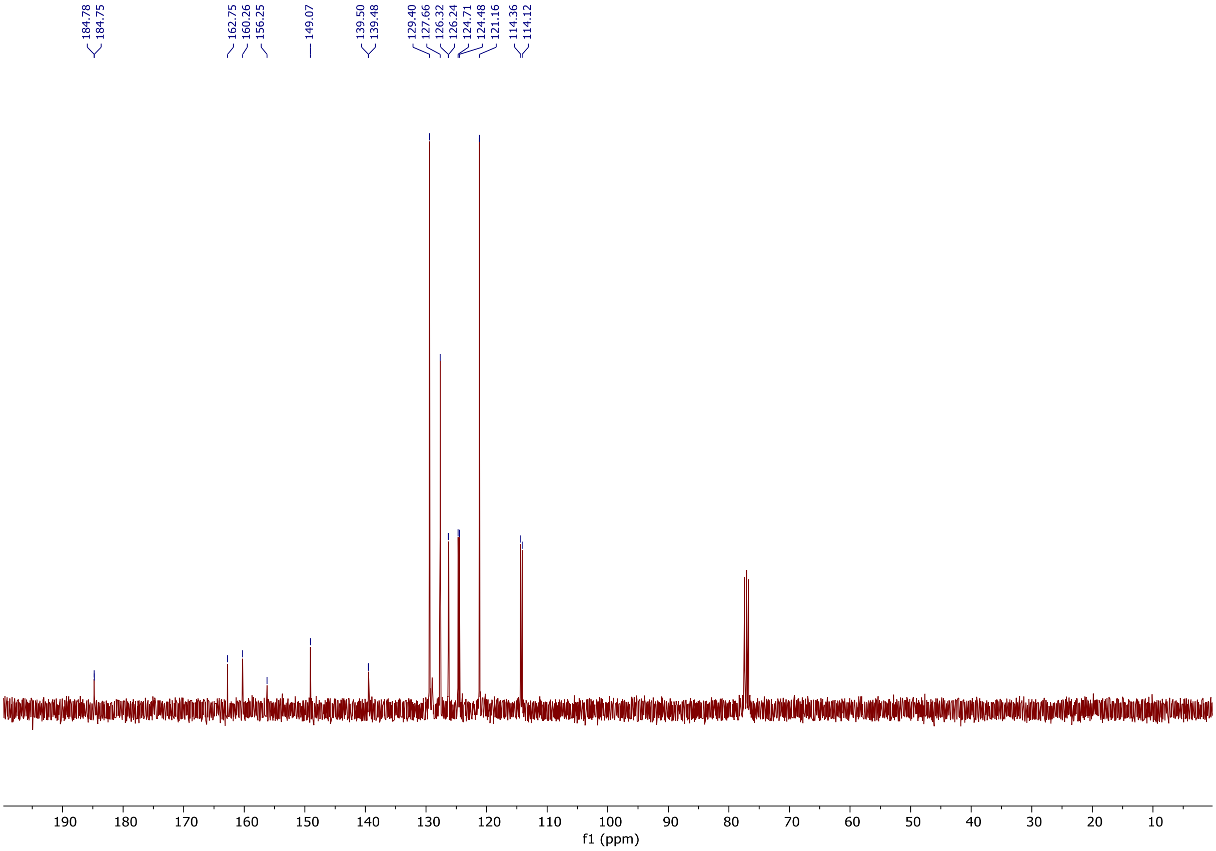

Figure S11 ^{13}C NMR spectrum of compound **1d** in $CDCl_3$.

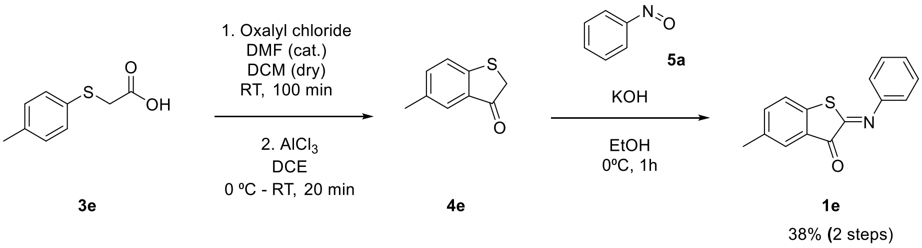

Scheme S4 Synthesis of **1e**.

4e: 5-methylbenzo[b]thiophen-3(2H)-one

2-(*p*-Tolylthio)acetic acid **3e** (1.03 g, 5.5 mmol, 1.0 eq) was dissolved in dry DCM (10 mL) and oxalyl chloride (1.00 mL, 11.8 mmol, 2.1 eq) and DMF (1 drop) were added. The reaction mixture was stirred at room temperature while gas formation was observed. After 100 minutes, gas formation stopped, after which the reaction mixture was concentrated *in vacuo* and the residue redissolved in DCE (10 mL) and cooled to 0 °C. $AlCl_3$ (1.0 g, 7.8 mmol, 1.4 eq) was added portion-wise. The reaction mixture was stirred for 20 minutes at room temperature and after completion (monitored by TLC) the reaction mixture was quenched on ice. DCM (50 mL) and H_2O (50 mL) were added, and the layers were separated. The aqueous layer was extracted with DCM (3 x 50 mL) and the combined organic layers were washed with water (2 x 25 mL) and brine (2 x 25 mL), dried over $MgSO_4$, and concentrated *in vacuo*. The crude product was used without further purification. The product was purified flushing over a plug of silica (Silicagel 40 – 63 nm, Et_2O). The product was yielded as a deep purple solid (0.59

g, 3.6 mmol, 66% yield). Mp: 66 – 68 °C. ^{1}H NMR (400 MHz, $CDCl_3$) δ 7.58 (s, 1H, Ar**H** (3)), 7.37 (d, J = 8.1 Hz, 1H, Ar**H** (6)), 7.31 (d, J = 8.1 Hz, 1H, Ar**H** (1)), 3.79 (s, 2H, C**H**$_2$ (8)), 2.36 (s, 3H, C**H**$_3$ (11)). ^{13}C NMR (101 MHz, $CDCl_3$) δ 20.7, 39.6, 124.2, 126.6, 131.0, 134.8, 137.0, 151.2, 200.1. HRMS (APCI+) calc. for $[M+H]^+$ ($C_9H_9OS^+$) 165.0369, found: 165.0367.

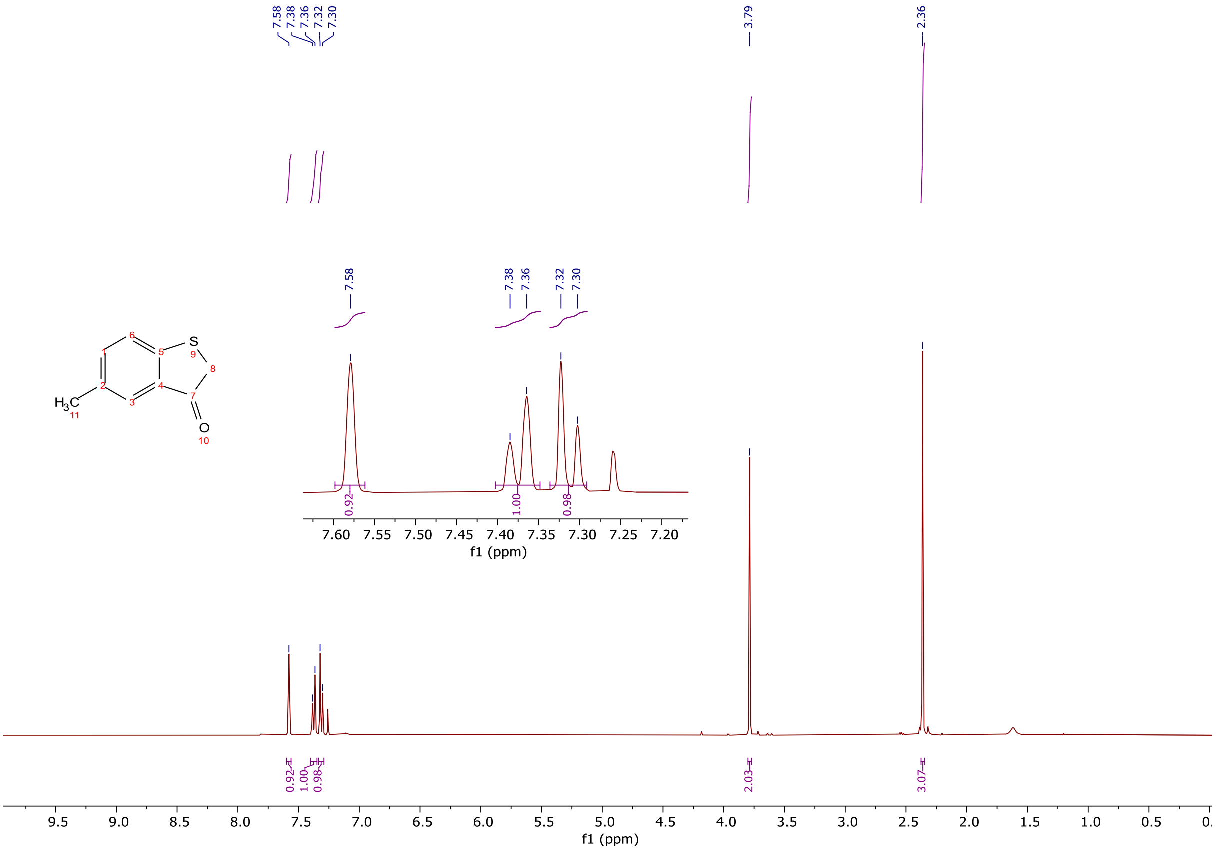

Figure S12 ^{1}H NMR spectrum of compound **4e** in $CDCl_3$.

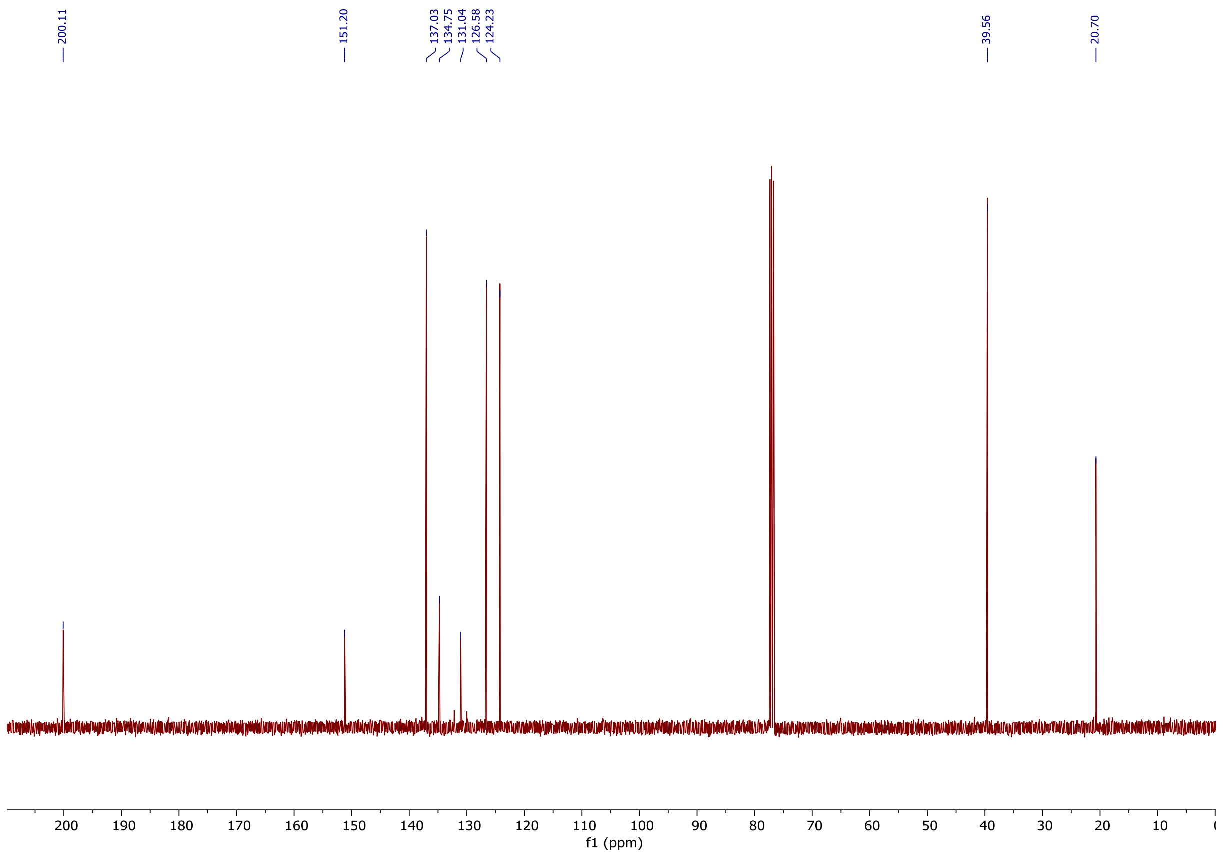

Figure S13 ^{13}C NMR spectrum of compound **7e** in $CDCl_3$.

1e: (*Z*)-5-methyl-2-(phenylimino)benzo[b]thiophen-3(2H)-one

5-Methylbenzo[b]thiophen-3(2H)-one **7e** (0.11 g, 0.67 mmol) and nitrosobenzene (80 mg, 0.75 mmol) were dissolved in EtOH (6 mL) and the reaction mixture was cooled on an ice-bath. KOH (25 g/L in EtOH) was added dropwise (10 drops) and the mixture was allowed to reach room temperature and was stirred vigorously. After 1 h, TLC indicated complete consumption of the starting material. Then, DCM (50 mL) and H_2O (50 mL) were added, and the layers were separated. The aqueous layer was extracted with DCM (3 x 50 mL) and the combined organic layers were washed with water (25 mL) and brine (25 mL), dried over $MgSO_4$, and concentrated *in vacuo*. The product was purified by flash chromatography (Silicagel 40 – 63 nm, DCM). The product was obtained as an orange solid (0.10 g, 0.39 mmol, 38% yield over 2 steps). Mp: 146 – 148 °C. ^{1}H NMR (400 MHz, $CDCl_3$) δ 7.72 (s, 1H, Ar**H**), 7.41 (m, 3H, Ar**H**), 7.24 (m, 4H, Ar**H**), 2.36 (s, 3H, C**H**$_3$). ^{13}C NMR (101 MHz, $CDCl_3$) δ 185.6, 157.0, 149.4, 141.3, 138.1, 136.9, 129.3, 127.9, 127.7, 127.3, 124.6, 121.1, 20.9. HRMS (ESI+) calc. for $[M+H]^+$ ($C_{15}H_{12}NOS^+$) 254.0634, found: 254.0634.

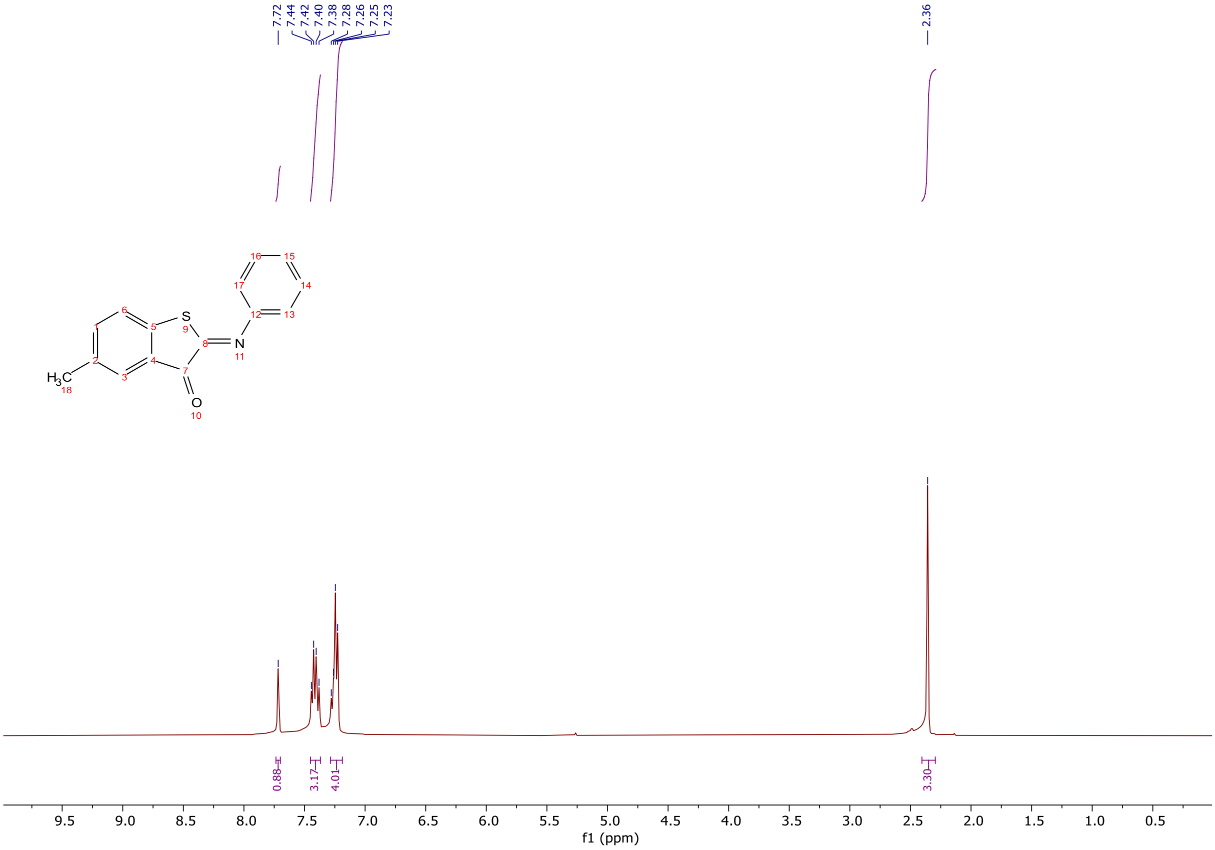

Figure S14 ^{1}H NMR spectrum of compound **1e** in $CDCl_3$.

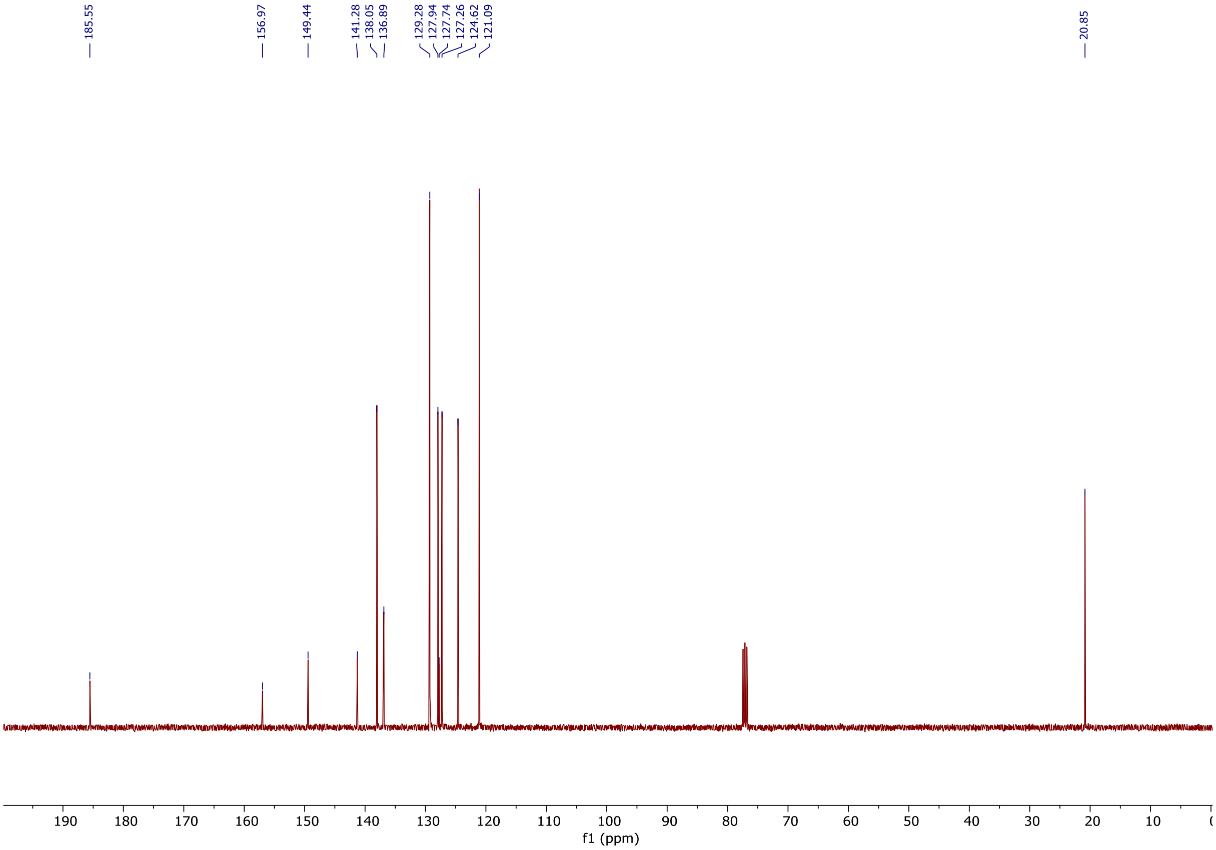

Figure S2.15 ^{13}C NMR spectrum of compound **1e** in $CDCl_3$.

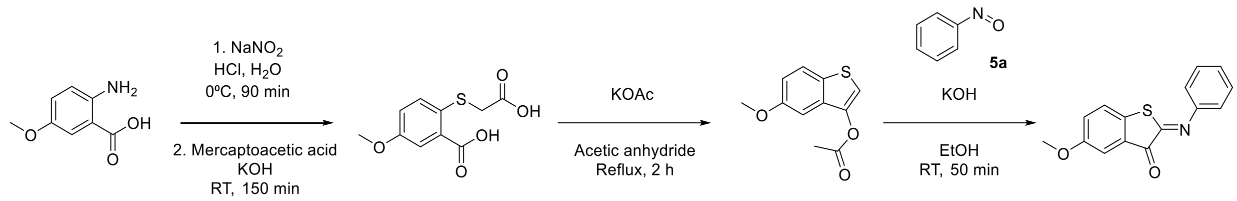

10 **7f** 53% **8f** 89% **1f** 47%

Scheme S5 Synthesis of **1f**.

7f: 2-((carboxymethyl)thio)-5-methoxybenzoic acid

To 2-amino-5-methoxybenzoic acid **10** (0.15 g, 0.9 mmol, 1.0 eq) was added aq. 1 N HCl (3.0 µL) and the reaction mixture was cooled on an ice-water bath. $NaNO_2$ (0.10 g, 1.5 mmol, 1.6 eq) was dissolved in H_2O (0.5 mL) and was added drop-wise to the solution, which was stirred on an ice-bath for 90 minutes. A solution of mercaptoacetic acid (84 µL, 1.2 mmol, 1.3 eq) and KOH (0.26 g, 4.6 mmol, 5.1 eq) in H_2O (1.0 mL) was added drop-wise, and the reaction mixture was stirred vigorously at room temperature. After 150 min, TLC indicated complete consumption of the starting material. Then, DCM (15 mL) and aq. 1 N HCl (15 mL) were added, and the layers were separated. The aqueous layer was washed with DCM (3 x 10 mL) and the combined organic layers were washed with brine (25 mL), dried over $MgSO_4$, and concentrated *in vacuo*. The crude product was crystallized in DCM and the residue was collected to give the product as an orange oil (0.12 g, 0.48 mmol, 53%). ^{1}H NMR (500 MHz, DMSO-d_6) δ 7.38 (d, J = 3.0 Hz, 1H, Ar**H**), 7.33 (d, J = 8.9 Hz, 1H, Ar**H**), 7.15 (dd, J = 8.8, 3.0 Hz, 1H, Ar**H**), 3.78 (s, 3H, C**H**$_3$), 3.74 (s, 2H, C**H**$_2$). HRMS (ESI+) calc. for $[M+H]^+$ ($C_{10}H_{11}O_5S^+$) 243.0322, found: 243.0320. The crude product was used without further purification.

8f: 5-methoxybenzo[b]thiophen-3-yl acetate

2-((Carboxymethyl)thio)-5-methoxybenzoic acid **7f** (78 mg, 0.32 mmol, 1.0 eq) was dissolved in acetic anhydride (1.5 mL) and KOAc (32 mg, 0.33 mmol, 1.0 eq) was added. The reaction mixture was heated under reflux and stirred vigorously. After 2 h, TLC indicated complete consumption of the starting material. Then, the reaction mixture was cooled to room temperature. Et_2O (25 mL) and H_2O (25 mL) were added, and the layers were separated. The aqueous layer was extracted with Et_2O (3 x 10 mL), and the combined organic layers were washed with water (25 mL) and brine (25 mL), dried over $MgSO_4$, and concentrated *in vacuo* to obtain the product as a brown oil (64 mg, 0.29 mmol, 31% yield over 2 steps). ^{1}H NMR (400 MHz, $CDCl_3$) δ 7.64 (d, J = 8.8 Hz, 1H, Ar**H** (6)), 7.38 (s, 1H, Ar**H** (3)), 7.07 (d, J = 2.5 Hz, 1H, C=C**H** (8)), 7.01 (dd, J = 8.8, 2.5 Hz, 1H, Ar**H** (1)), 3.87 (s, 3H, OC**H**$_3$ (15)), 2.38 (s, 3H, C**H**$_3$CO (13)). ^{13}C NMR (101 MHz, $CDCl_3$) δ 168.2, 157.6, 140.2, 133.0, 129.3, 123.7, 115.7, 113.1, 102.3, 55.6, 21.1. HRMS (ESI+) calc. for $[M+H]^+$ ($C_{11}H_{11}O_3S^+$) 223.0423, found: 223.0422.

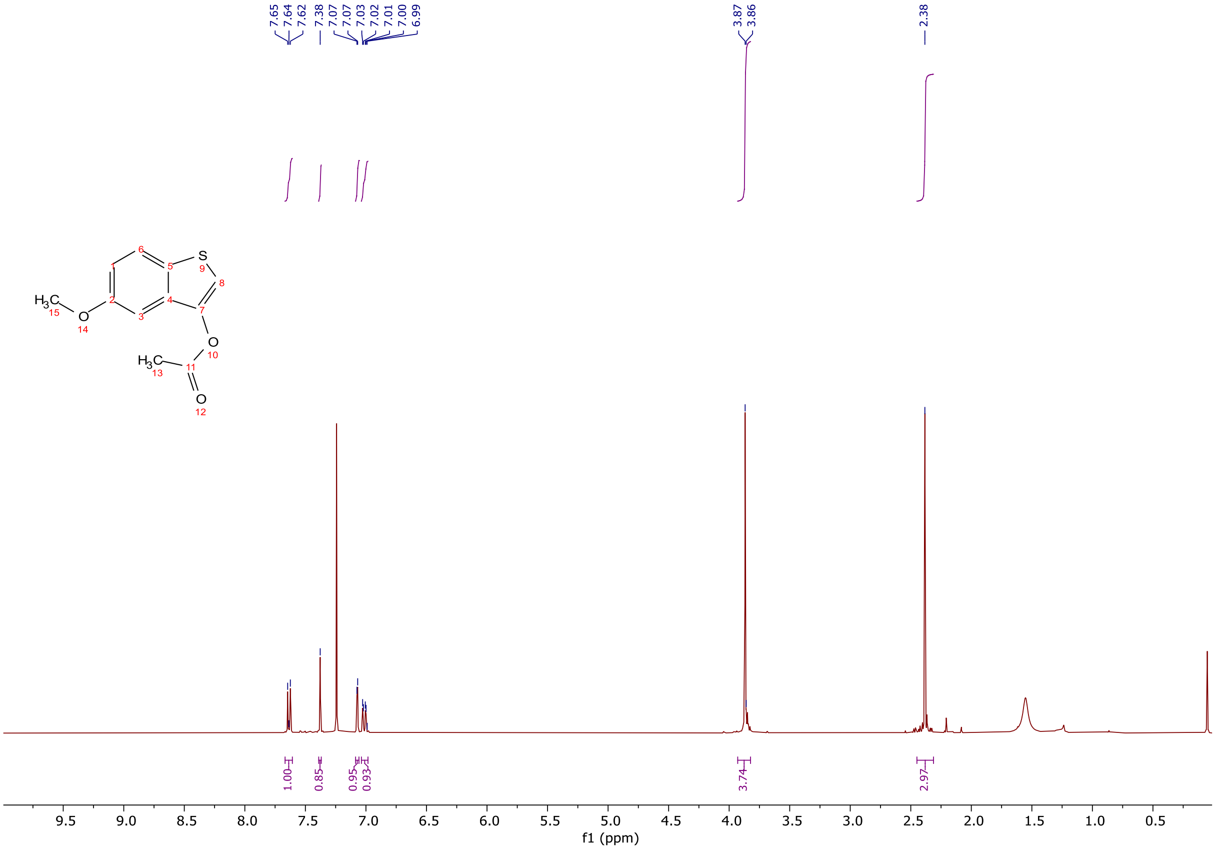

Figure S16 ^{1}H NMR spectrum of compound **8f** in $CDCl_3$.

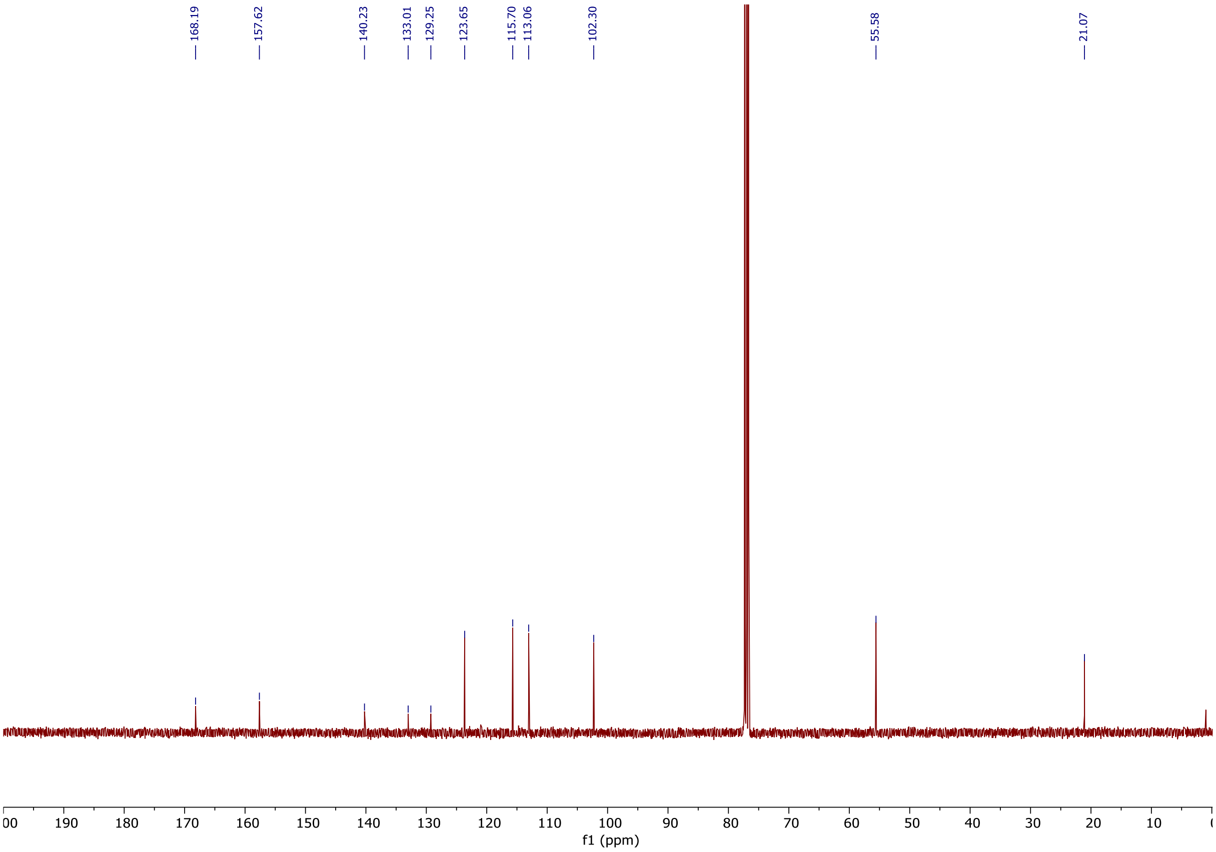

Figure S17 ^{13}C NMR spectrum of compound **8f** in $CDCl_3$.

1f: (*Z*)-5-methoxy-2-(phenylimino)benzo[b]thiophen-3(2H)-one

5-methoxybenzo[b]thiophen-3-yl acetate **8f** (64 mg, 0.29 mmol) and nitrosobenzene (39 mg, 0.36 mmol) were dissolved in EtOH (5 mL) and the reaction mixture was cooled on an ice-bath. KOH (25 g/L in EtOH, 0.32 mL, 0.14 mmol, 0.49 eq) was added dropwise and the mixture was allowed to reach room temperature and stirred vigorously. After 1 h, TLC indicated complete consumption of the starting material. Then, DCM (10 mL) and H_2O (10 mL) were added, and the layers were separated. The aqueous layer was extracted with DCM (3 x 10 mL) and the combined organic layers were washed with water (25 mL) and brine (25 mL), dried over $MgSO_4$, and concentrated *in vacuo*. The product was purified by precipitation from EtOAc upon the addition of pentane. The filtrate was collected and concentrated in vacuo to obtain the product as an orange solid (36 mg, 0.13 mmol, 47% yield). Mp: >250 °C. ^{1}H NMR (400 MHz, $CDCl_3$) δ 7.47 – 7.41 (m, 3H, Ar**H** (3, 14, and 16)), 7.30 – 7.25 (m, 3H, Ar**H** (13, 15, and 17)), 7.26 (s, 1H, Ar**H** (6)), 7.21 (dd, *J* = 8.6, 2.7 Hz, 1H, Ar**H** (1)), 3.85 (s, 3H, C**H**$_3$). ^{13}C NMR (101 MHz, $CDCl_3$) δ 185.6, 158.9, 157.4, 149.4, 136.1, 129.3, 128.6, 127.3, 125.8, 125.6, 121.1, 110.2, 55.9. IR (cm-1): 1703 (C=O, stretch), 1473 (C-C, stretch), 1318 (O-H, bend), 1278 (C-N, stretch), 1024 (C-O, stretch), 773 (C-H, bend), 693 (C-H, bend). HRMS (ESI+) calc. for $[M+H]^+$ ($C_{15}H_{12}NO_2S^+$) 270.0583, found: 270.0583.

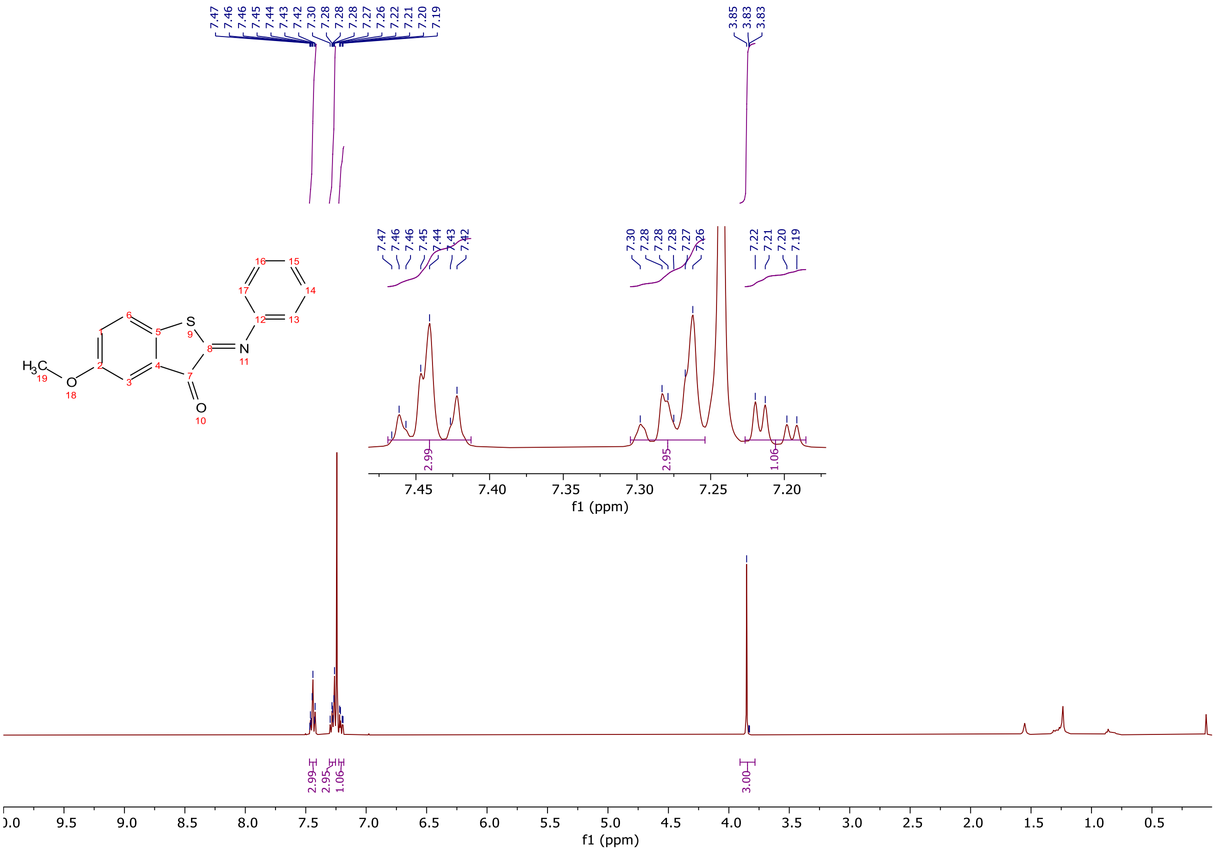

Figure S18 ^{1}H NMR spectrum of compound **1f** in $CDCl_3$.

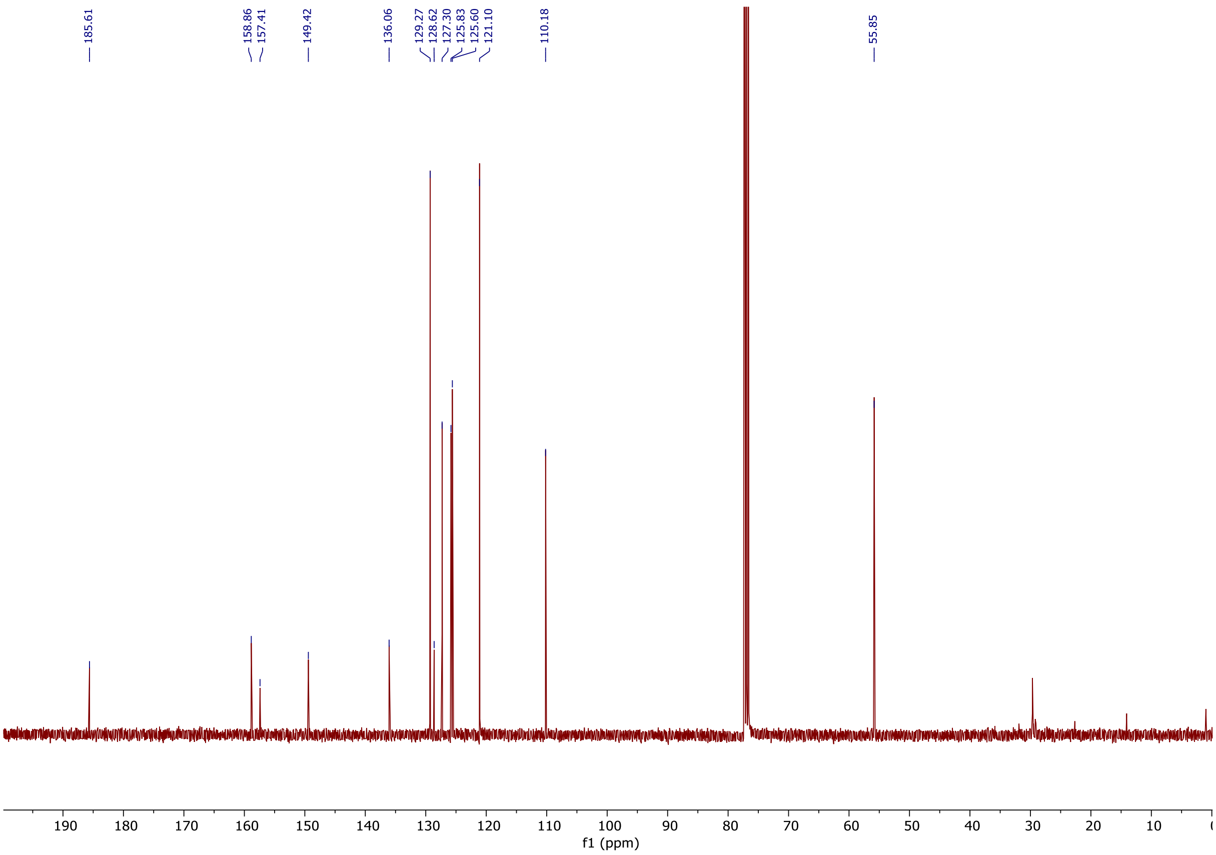

Figure S19 ^{13}C NMR spectrum of compound **1f** in $CDCl_3$.

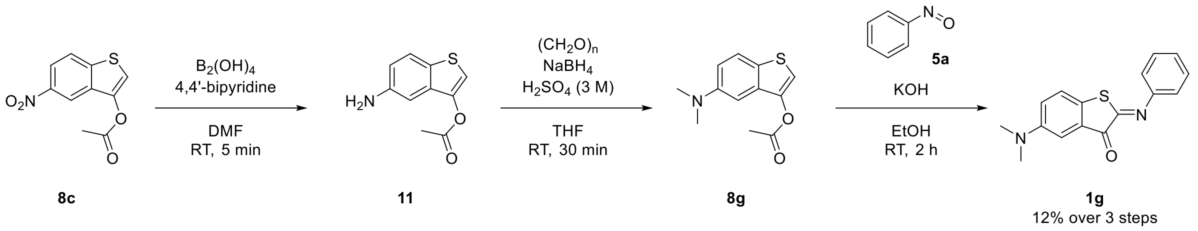

Scheme S6 Synthesis of **1g**.

11: 5-aminobenzo[b]thiophen-3-yl acetate

Synthesized according to a modified procedure by *Jang et al.*[18]:

5-Nitrobenzo[b]thiophen-3-yl acetate **8c** (79 mg, 0.34 mmol, 1.0 eq), $B_2(OH)_4$ (0.13 g, 1.5 mmol, 4.4 eq), and 4,4'-bipyridine (1.5 mg, 0.010 mmol, 0.029 eq) were added to a vial. Then, DMF (2.2 mL) was added and the reaction mixture stirred at room temperature for 5 min. Then, DCM (10 mL) and H_2O (10 mL) were added, the layers were separated, and the aqueous layer extracted with DCM (3 x 10 mL). The combined organic layers were washed with brine (25 mL), dried using $MgSO_4$, and concentrated *in vacuo*. The residual DMF was removed by freeze-drying to afford **11** as a yellow oil. ^1H NMR (500 MHz, $CDCl_3$) δ 7.58 (d, J = 8.2 Hz, 1H, Ar**H**), 7.34 (s, 1H, C=C**H**), 6.96 (s, 1H, Ar**H**), 6.87 – 6.80 (m, 1H, Ar**H**), 3.84 (s, 2H, N**H**$_2$), 2.40 (s, J = 5.8 Hz, 3H, C**H**$_3$). The crude product was used without further purification and characterization.

8g: 5-(dimethylamino)benzo[b]thiophen-3-yl acetate

Synthesized according to a modified procedure by *Kink et al.*[19]:

A suspension of crude N,N-diethyl-5-amino-2-(methylthio)benzamide and powdered sodium borohydride (64 mg, 1.7 mmol) in THF (1.7 mL) was added to a stirred solution of aq. 37% paraformaldehyde (0.11 mL, 1.4 mmol) in aq. 3 M H_2SO_4 (0.19 mL, 0.57 mmol) at room temperature. Stirring was continued for 60 min. Then, DCM (10 mL) and H_2O (10 mL) were added, and the layers were separated. The aqueous layer was extracted with DCM (3 x 10 mL) and the combined organic layers were washed with water (1 x 25 mL), dried over $MgSO_4$, and concentrated *in vacuo* to afford **8g** as a green oil. ^{1}H NMR (500 MHz, $CDCl_3$) *δ* 7.64 (d, *J* = 8.9 Hz, 1H, Ar**H**), 7.35 (s, 1H, Ar**H**), 6.99 (dd, *J* = 8.9, 2.6 Hz, 1H, Ar**H**), 6.90 (d, *J* = 2.5 Hz, 1H, Ar**H**), 3.03 (s, 6H, 2 x NC**H**$_3$), 2.42 (s, 3H, CC**H**$_3$). The crude product was used without further purification and characterization.

1g: (*Z*)-5-(dimethylamino)-2-(phenylimino)benzo[b]thiophen-3(2H)-one

5-(dimethylamino)benzo[b]thiophen-3-yl acetate **8g** and nitrosobenzene (21 mg, 0.21 mmol) were dissolved in EtOH (5 mL) and the reaction mixture was cooled on an ice-bath. KOH (25 g/L in EtOH, 0.2 mL, 0.080 mmol, 0.49 eq) was added dropwise and the mixture was allowed to reach room temperature and stirred vigorously and the mixture was allowed to reach room temperature and was stirred vigorously. After 1 h, TLC indicated complete consumption of the starting material. Then, DCM (10 mL) and H_2O (10 mL) were added, and the layers were separated. The aqueous layer was extracted with DCM (3 x 10 mL) and the combined organic layers were washed with water (25 mL) and brine (25 mL), dried over $MgSO_4$, and concentrated *in vacuo*. The crude product was purified by flash chromatography (Silicagel 40 – 63 nm, 0-20% EtOAc in petroleum ether). The product was obtained as a purple solid (12 mg, 0.041 mmol, 12% over 3 steps). Mp: >250 °C. ^{1}H NMR (400 MHz, $CDCl_3$) *δ* 7.46 – 7.39 (m, 2H, Ar**H** (14 and 16)), 7.27 – 7.22 (m, 4H, Ar**H** (3, 13, 15, and 17)), 7.19 (d, *J* = 8.6 Hz, 1H, Ar**H** (6)), 6.99 (dd, *J* = 8.7, 2.9 Hz, 1H, Ar**H** (1)), 2.99 (s, 6H, 2 x C**H**$_3$ (19 and 20)). ^{13}C NMR (101 MHz, $CDCl_3$) *δ* 186.4, 158.4, 149.7, 149.5, 130.2, 129.2, 128.3, 127.0, 125.0, 121.6, 121.1, 110.2, 40.6. IR (cm^{-1}): 2923 (N-H, stretch), 1706 (C=O, stretch), 1607 (N-H, bend), 1493 (C-C, stretch), 1354 (C-N, stretch), 1034 (C-N, stretch), 962 (C=C, bend), 773 (C-H, bend), 697 (C-H, bend). HRMS (ESI+) calc. for $[M+H]^+$ ($C_{16}H_{15}N_2OS^+$) 283.0990, found: 283.0894.

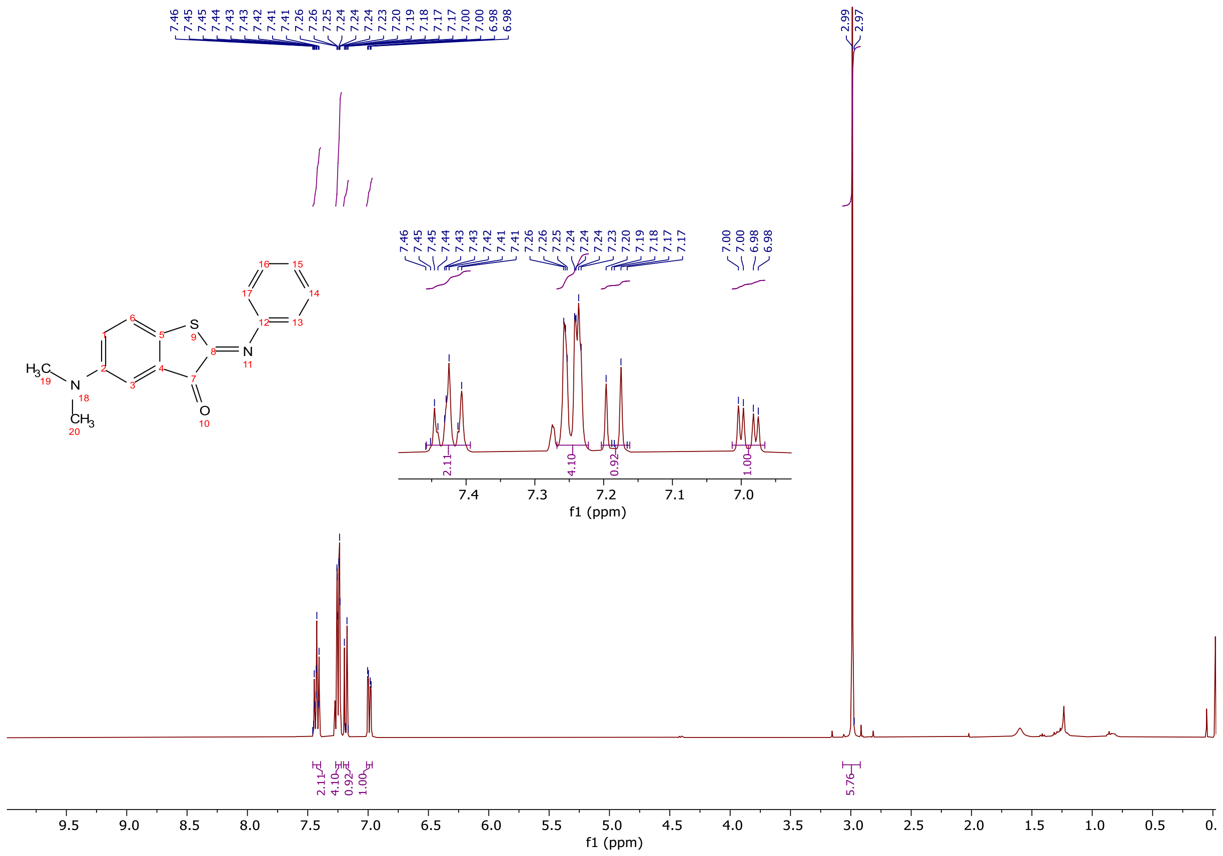

Figure S20 ^{1}H NMR spectrum of compound **1g** in $CDCl_3$.

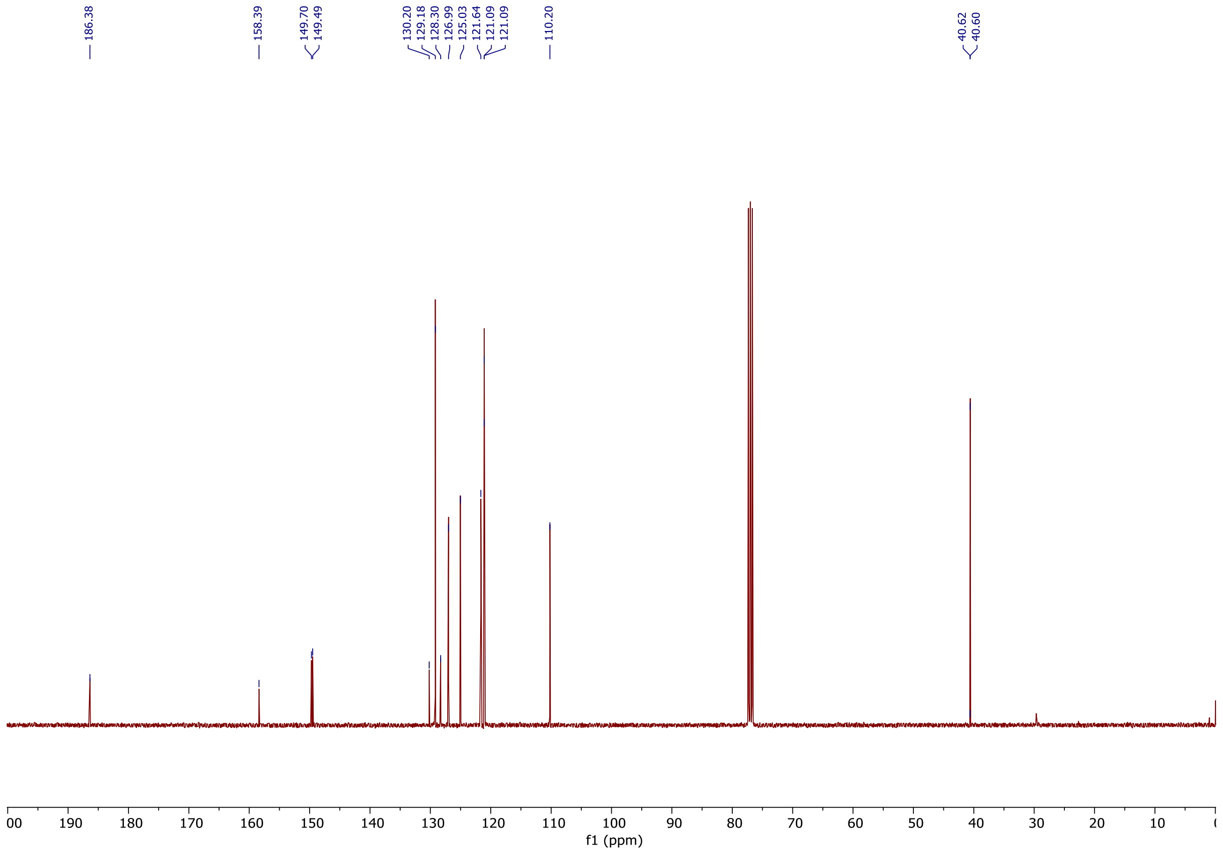

Figure S21 ^{13}C NMR spectrum of compound **1g** in $CDCl_3$.

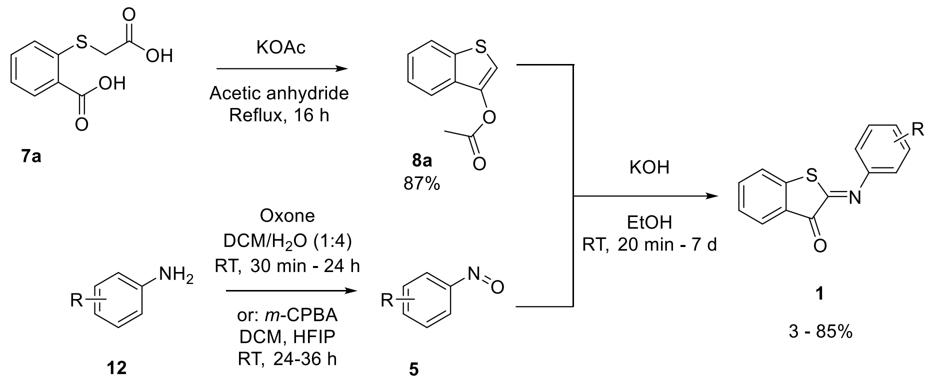

Scheme S7 Synthesis of ITIs **1h–y**, **1α**, and **1β**.

8a: benzo[b]thiophen-3-yl acetate

2-((Carboxymethyl)thio)benzoic acid **7a** (1.0 g, 4.8 mmol, 1.0 eq) was dissolved in acetic anhydride (10 mL) and KOAc (1.1 g, 4.8 mmol, 1.0 eq) was added. The reaction mixture was stirred and heated under reflux under nitrogen atmosphere. After 16 h, TLC indicated complete consumption of the starting material. Then, Et_2O (50 mL) and H_2O (50 mL) were added, the layers were separated, and the aqueous layer was extracted with Et_2O (3 x 50 mL). The combined organic layers were washed with water (25 mL) and brine (25 mL), dried using $MgSO_4$, concentrated *in vacuo* and co-evaporat ed with toluene (3 x 50 mL) to remove the residual acetic anhydride. The product was obtained as a light pink oil (0.87 g, 4.5 mmol, 87 % yield) ^{1}H NMR (400 MHz, $CDCl_3$) δ 7.83 – 7.78 (m, *J* = 7.4 Hz, 1H, Ar**H** (3)), 7.71 – 7.66 (m, *J* = 7.1 Hz, 1H, Ar**H** (6)), 7.42 – 7.39 (m, 1H, C=C**H** (8)), 7.39 – 7.35 (m, 2H, Ar**H** (1 and 2)), 2.40 (s, *J* = 2.0 Hz, 3H, C**H**$_3$ (13)). ^{1}H spectra correspond to literature.[18]

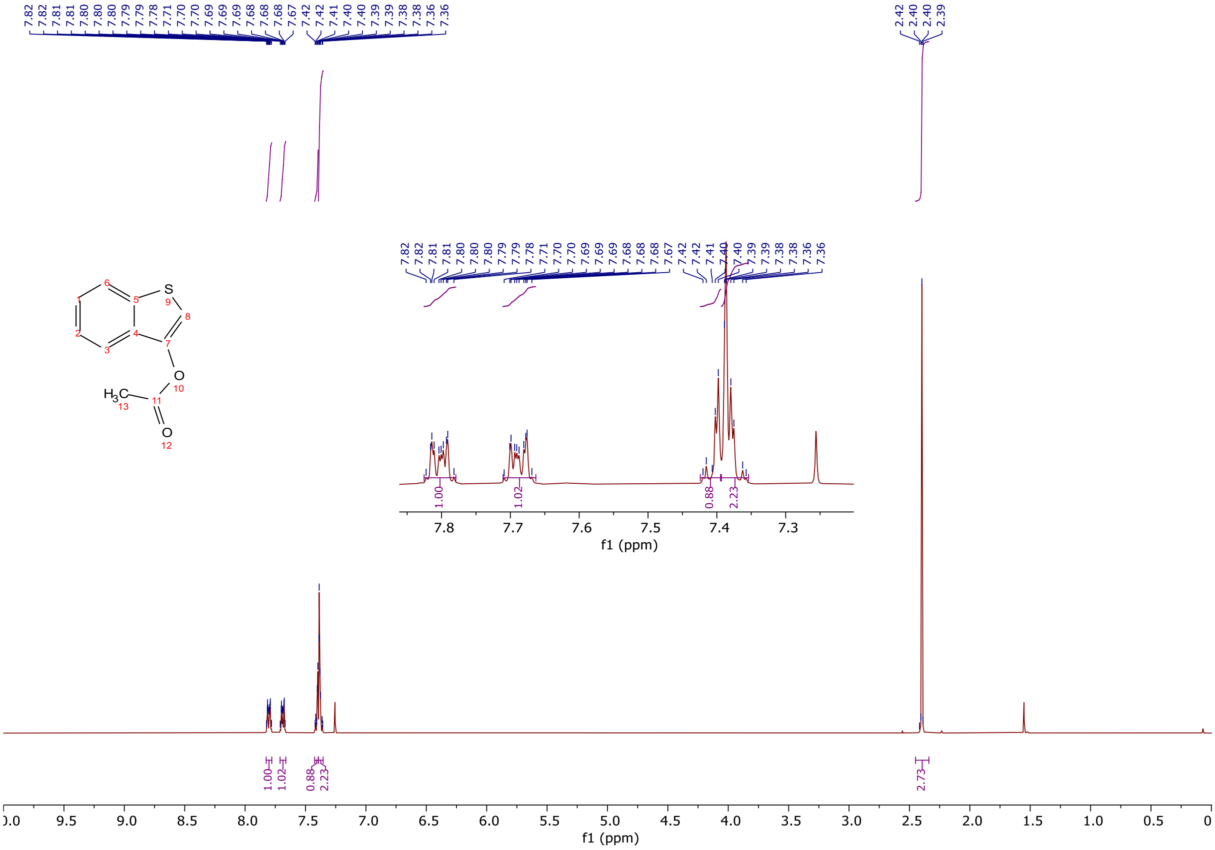

Figure S22 ^{1}H NMR spectrum of compound **3** in $CDCl_3$.

5i: 1-fluoro-4-nitrosobenzene

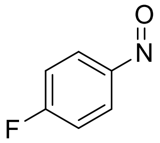

4-Fluoroaniline **4i** (0.45 mL, 4.5 mmol, 1.0 eq) and Oxone (2.7 g, 9.0 mmol, 2.0 eq) were dissolved in DCM/H_2O (1:9, v/v, 50 mL). The reaction mixture was vigorously stirred at room temperature. After 3 h, TLC indicated complete consumption of the starting material. Then, DCM (50 mL) and water (50 mL) were added, the layers separated, and the aqueous layer extracted with DCM (3 x 50 mL). The combined organic layers were washed with aq. 1 N HCl (50 mL), H_2O (50 mL) and brine (50 mL), dried over $MgSO_4$, and concentrated *in vacuo*. The product was obtained after flushing through a plug of silica gel in pentane and concentrating *in vacuo*. The product was used without further purification and characterization.

5l: 1-methoxy-2-nitrosobenzene

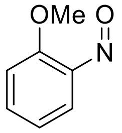

2-Methoxyaniline **4l** (0.30 mL, 2.7 mmol, 1.0 eq) and Oxone (3.2 g, 5.2 mmol, 2.0 eq) were dissolved in DCM/H_2O (1:5, v/v, 25 mL). The reaction mixture was stirred vigorously at room temperature under a nitrogen atmosphere. After 1 h, TLC indicated complete consumption of the starting material. Then, DCM (25 mL) was added, and the layers were separated. The aqueous layer was extracted with DCM (3 x 50 mL), and the combined organic layers were washed with aq. 3 N HCl (2 x 40 mL), sat. aq. $NaHCO_3$ (3 x 40 mL) and then water (3 x 40 mL), dried over $MgSO_4$, and the solvents evaporated. The product was purified by flash chromatography (Silicagel 40-63 nm, 0-10% EtOAc in *n*-heptane) to give yellow crystals (54 mg, 0.39 mmol, 15%). ^{1}H NMR (400 MHz, $CDCl_3$) δ 7.66 (ddd, J = 8.8, 7.2, 1.8 Hz, 1H, Ar**H** (3)), 7.34 (dd, J = 8.5, 1.1 Hz, 1H, Ar**H** (4)), 6.83 (ddd, J = 8.2, 7.2, 1.1 Hz, 1H, Ar**H** (2)), 6.27 (dd, J = 8.1, 1.8 Hz, 1H, Ar**H** (1)), 4.25 (s, J = 1.2 Hz, 3H, C**H**$_3$ (10)). ^{1}H spectra correspond to literature.[20]

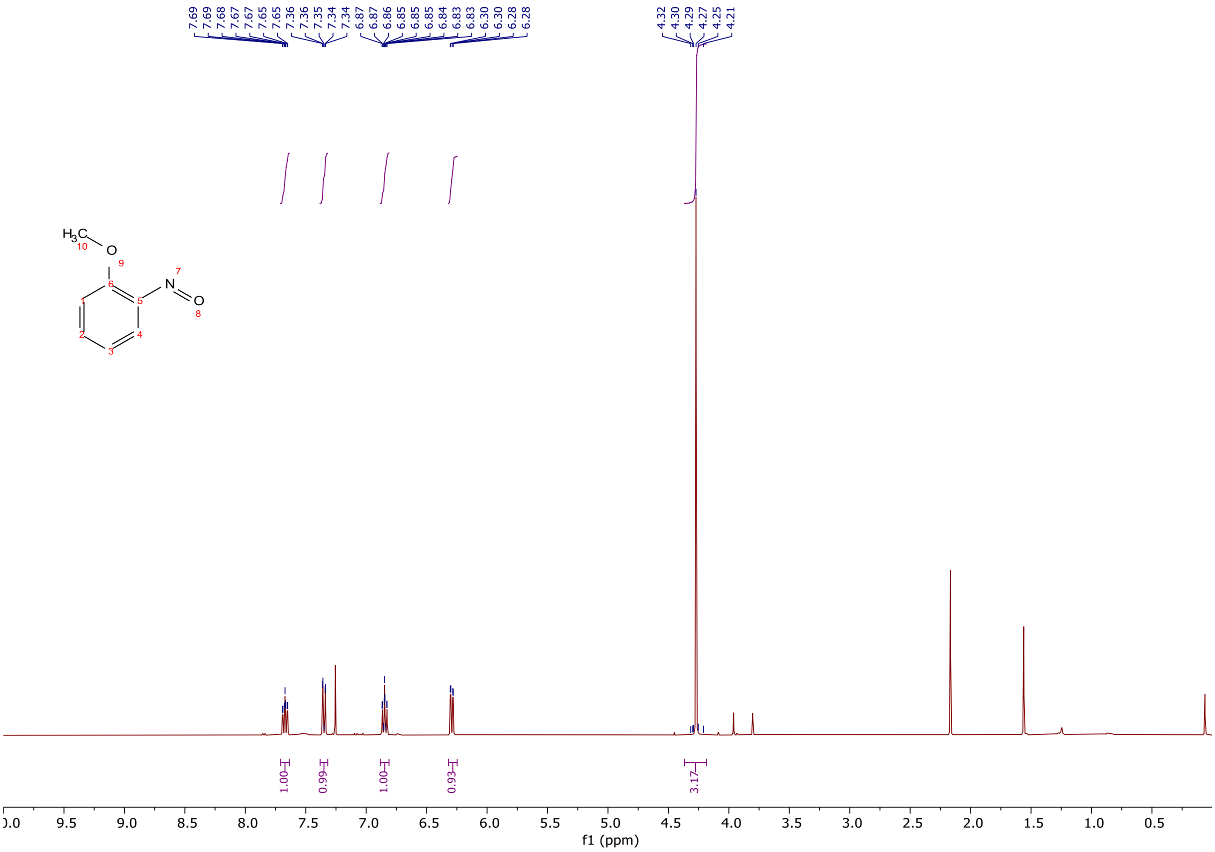

Figure S23 ^{1}H NMR spectrum of compound **5l** in $CDCl_3$.

5m: 1-methyl-2-nitrosobenzene

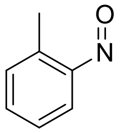

o-Toluidine **4m** (1.0 mL, 9.1 mmol, 1.0 eq) and Oxone (5.6 g, 18 mmol, 2.0 eq) were dissolved in DCM/H_2O (1:4, v/v, 45 mL). The reaction mixture was stirred vigorously at room temperature under a nitrogen atmosphere. After 1 h, TLC indicated complete consumption of the starting material. Then, DCM (20 mL) was added, and the layers were separated. The aqueous layer was extracted with DCM (3 x 20 mL) and the combined organic layers were washed with water (25 mL), dried over $MgSO_4$, and concentrated *in vacuo*. The product was purified by flash chromatography (Silicagel 40-63 nm, 0-10% Et_2O in pentane). The product was obtained as a light-yellow solid (0.41 g, 3.0 mmol, 33 % yield). Mp: 52 - 60°C. ^{1}H NMR (400 MHz, $CDCl_3$) δ 7.60 (t, J = 7.4 Hz, 1H, Ar**H** (3)), 7.54 (d, J = 7.6 Hz, 1H, Ar**H** (4)), 7.16 (t, J = 7.5 Hz, 1H, Ar**H** (2)), 6.29 (d, J = 8.1 Hz, 1H, Ar**H** (1)), 3.35 (s, 3H, C**H**$_3$ (7)). ^{1}H NMR spectrum corresponds to literature.[21]

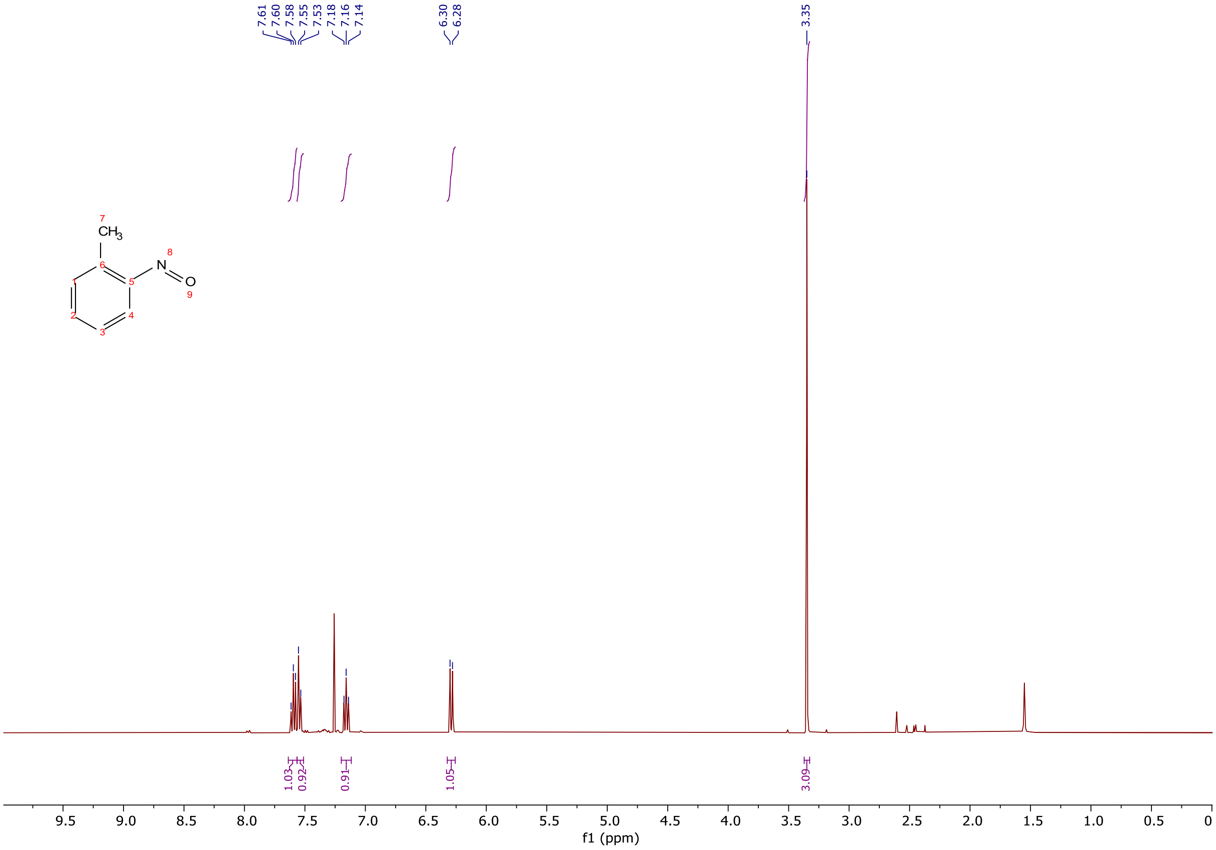

Figure S24 ^{1}H NMR spectrum of compound **5m** in $CDCl_3$.

5n: 1,3-dimethyl-2-nitrosobenzene

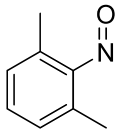

2,6-Dimethylaniline **4n** (1.0 mL, 8.3 mmol, 1.0 eq) and Oxone (5.1 g, 17 mmol, 2.0 eq) were dissolved in DCM/H_2O (1:3, v/v, 60 mL). The reaction mixture was stirred vigorously at room temperature under a nitrogen atmosphere. After 90 min, TLC indicated complete consumption of the starting material. Then, DCM (20 mL) was added, and the layers were separated. The aqueous layer was extracted with DCM (3 x 20 mL) and the combined organic layers were washed with aq. 1 N HCl (25 mL), brine (25 mL), dried over $MgSO_4$, and concentrated *in vacuo*. The product was obtained as a white solid (0.56 g, 4.1 mmol, 50%). ^{1}H NMR (400 MHz, $CDCl_3$) δ 7.31 (t, J = 7.6 Hz, 1H, Ar**H** (2)), 7.19 (d, J = 7.6 Hz, 2H, Ar**H** (1 and 3)), 2.47 (s, 6H, 2 x **CH**$_3$ (7 and 10)). ^{1}H spectra correspond to literature.[22]

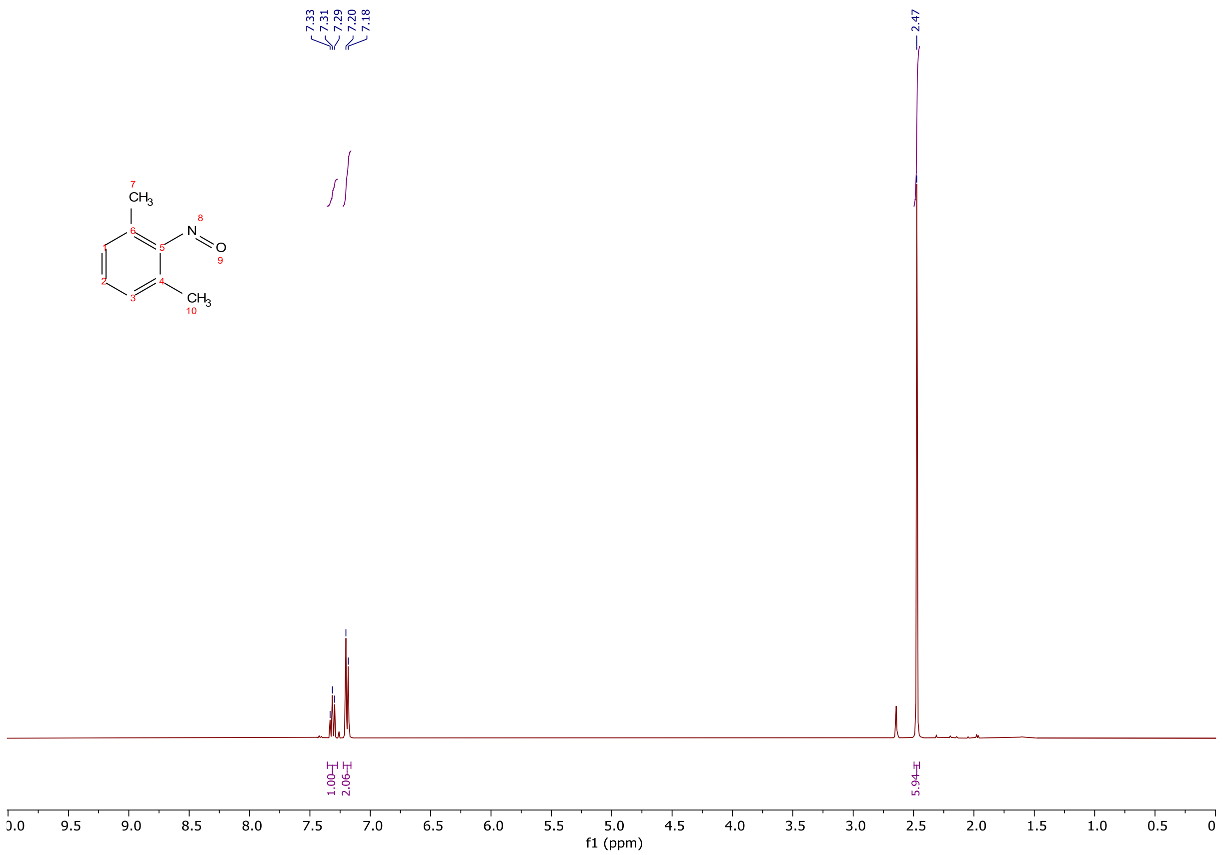

Figure S25 ^{1}H NMR spectrum of compound **5n** in $CDCl_3$.

5o: 1-ethyl-2-nitrosobenzene

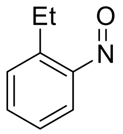

2-Ethylaniline **4o** (0.51 mL, 4.1 mmol, 1.0 eq) and Oxone (5.0 g, 8.1 mmol, 2.0 eq) were dissolved in DCM/H_2O (1:4, v/v, 45 mL). The reaction mixture was stirred at room temperature under a nitrogen atmosphere. After 35 min, TLC indicated complete consumption of the starting material. Then, DCM (50 mL) was added, and the layers were separated. The aqueous layer was extracted with DCM (2 x 50 mL), and the combined organic layers were washed with aq. 3 N HCl (2 x 40 mL), sat. aq. $NaHCO_3$ (2 x 50 mL), water (2 x 50 mL), and dried over $MgSO_4$. The volatiles were evaporated to give the product as brown crystals (0.17 g, 1.3 mmol, 30%), without the need for further purification. ^{1}H NMR (400 MHz, $CDCl_3$) δ 7.68 – 7.55 (m, 2H, Ar**H** (3 and 4)), 7.15 (ddd, *J* = 8.3, 6.9, 1.6 Hz, 1H, Ar**H** (2)), 6.19 (dd, *J* = 8.1, 1.4 Hz, 1H, Ar**H** (1)), 3.89 (q, *J* = 7.6 Hz, 2H, C**H**$_2$ (7)), 1.52 (t, *J* = 7.6 Hz, 3H, C**H**$_3$ (10)). ^{1}H spectra correspond to literature.[23]

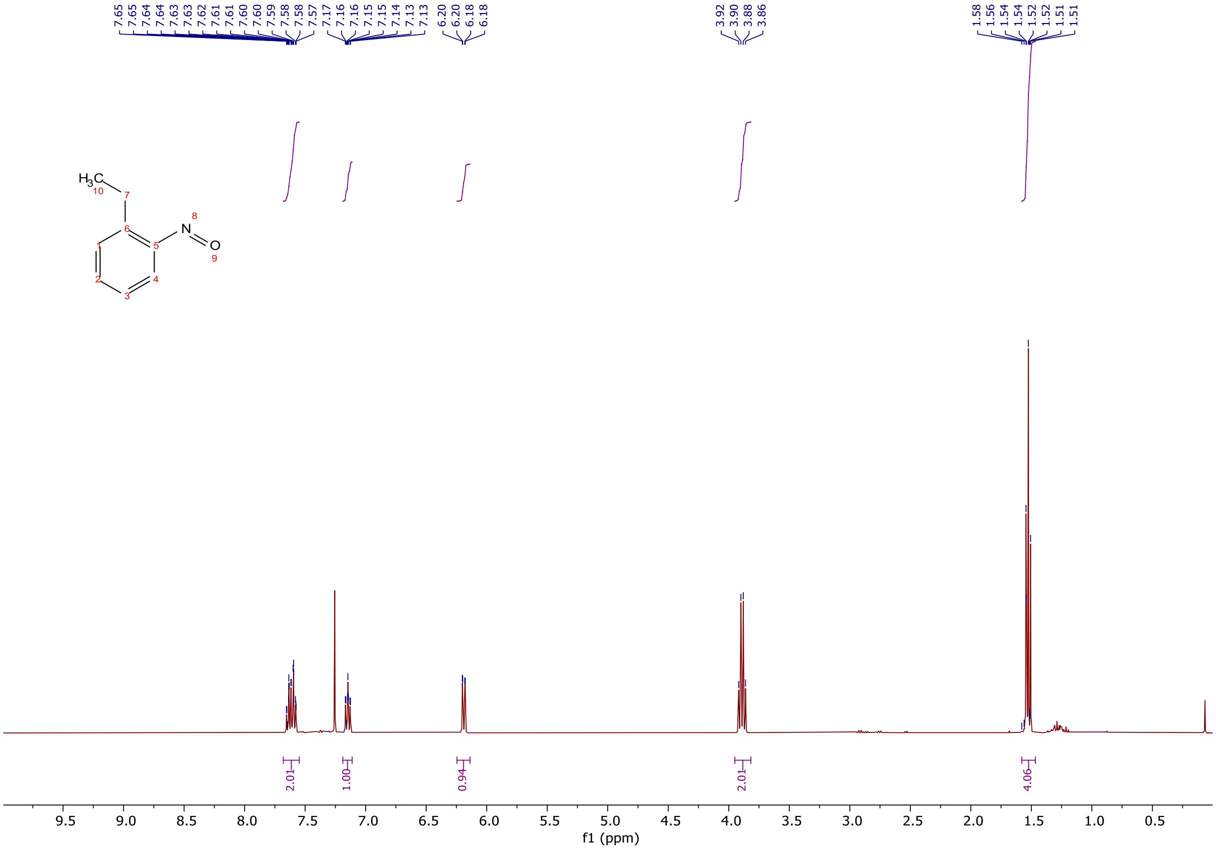

Figure S26 ^{1}H NMR spectrum of compound **5o** in $CDCl_3$.

5p: 1-isopropyl-2-nitrosobenzene

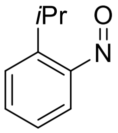

2-Isopropylaniline **4p** (0.25 mL, 2.21 mmol, 1.0 eq) and Oxone (2.7 g, 4.5 mmol, 2.0 eq) were dissolved in DCM/H_2O (1:4, v/v, 40 mL). The reaction mixture was stirred vigorously at room temperature under a nitrogen atmosphere. After 50 min, TLC indicated complete consumption of the starting material. Then, DCM (25 mL) was added, and the layers were separated. The aqueous layer was extracted with DCM (3 x 50 mL), and the combined organic layers were washed with aq. 3 N HCl (2 x 40 mL), sat. aq. $NaHCO_3$ (3 x 40 mL), water (3 x 40 mL), dried over $MgSO_4$, and the solvents evaporated. The product was purified by flash chromatography (Silicagel 40-63 nm, 0-10% EtOAc in *n*-heptane) to give the product as yellow crystals (92 mg, 0.62 mmol, 17%). ^{1}H NMR (400 MHz, $CDCl_3$) δ 7.74 – 7.62 (m, 2H, Ar**H**), 7.12 (ddd, *J* = 8.3, 6.8, 1.7 Hz, 1H, Ar**H**), 6.12 – 6.05 (m, 1H, Ar**H**), 5.29 (hept, *J* = 6.9 Hz, 1H, C**H**), 1.56 (d, *J* = 7.0 Hz, 6H, 2 x C**H**$_3$). ^{1}H spectra correspond to literature.[23]

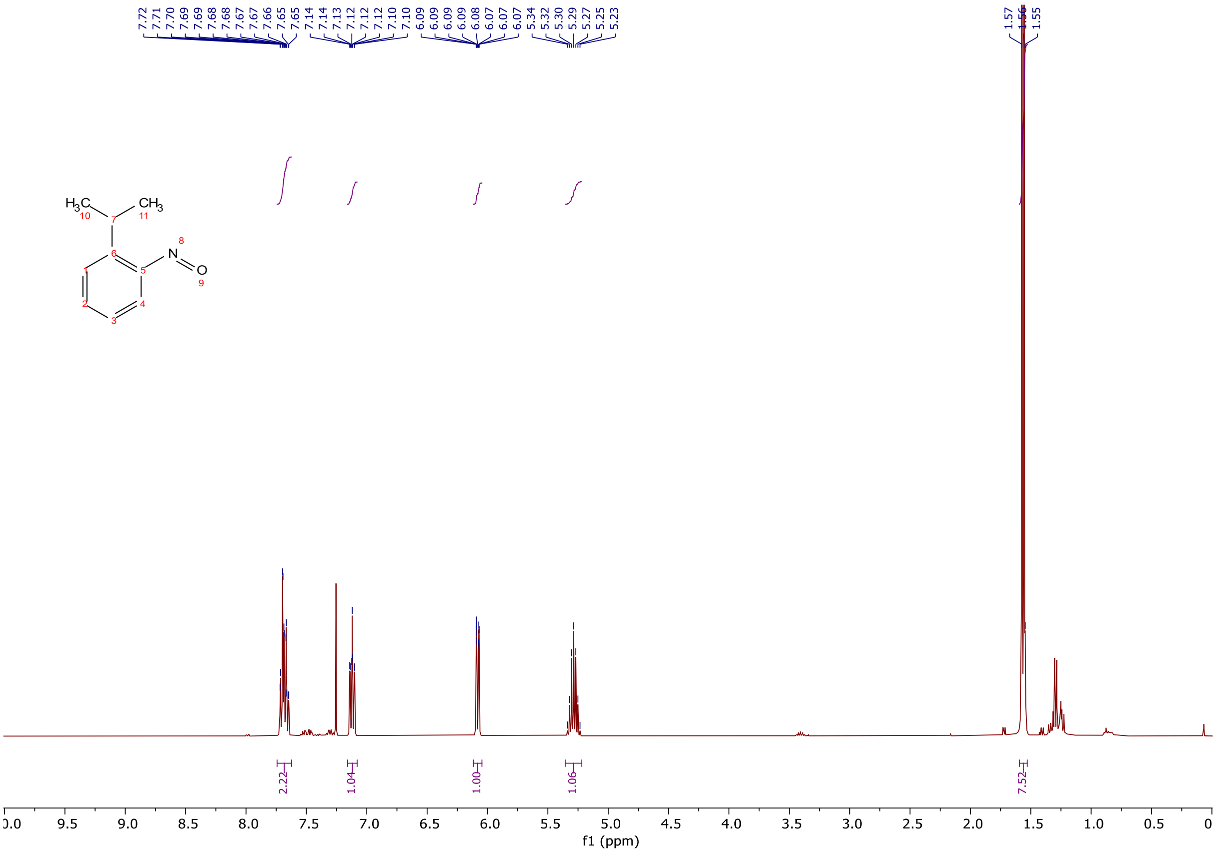

Figure S27 ^{1}H NMR spectrum of compound **5p** in $CDCl_3$.

5q: 1-bromo-2-nitrosobenzene

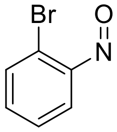

2-Bromoaniline **4q** (0.25 mL, 2.2 mmol, 1.0 eq) and Oxone (2.7 g, 4.5 mmol, 2.0 eq) were dissolved in DCM/H_2O (1:5, v/v, 6.0 mL). The reaction mixture was stirred vigorously at room temperature under a nitrogen atmosphere. After 1 h, TLC indicated complete consumption of the starting material. Then, DCM (20 mL) was added, and the layers were separated. The aqueous layer was extracted with DCM (3 x 50 mL), and the combined organic layers were washed with aq. 3 N HCl (3 x 40 mL), sat. aq. $NaHCO_3$ (3 x 50 mL), brine (3 x 40 mL), dried over $MgSO_4$, and the solvents evaporated. The product was purified by flash chromatography (Silicagel 40-63 nm, 0-5% EtOAc in *n*-heptane) to give the product as a brown powder (0.40 g, 2.1 mmol, 96%). ^{1}H NMR (400 MHz, $CDCl_3$) δ 7.99 (dd, J = 8.1, 1.2 Hz, 1H Ar**H** (4)), 7.54 (ddd, J = 8.0, 7.2, 1.7 Hz, 1H, Ar**H** (3)), 7.30 – 7.26 (m, 1H, Ar**H** (2)), 6.22 (dd, J = 8.0, 1.7 Hz, 1H, Ar**H** (1)). ^{1}H spectra correspond to literature.[21, 24]

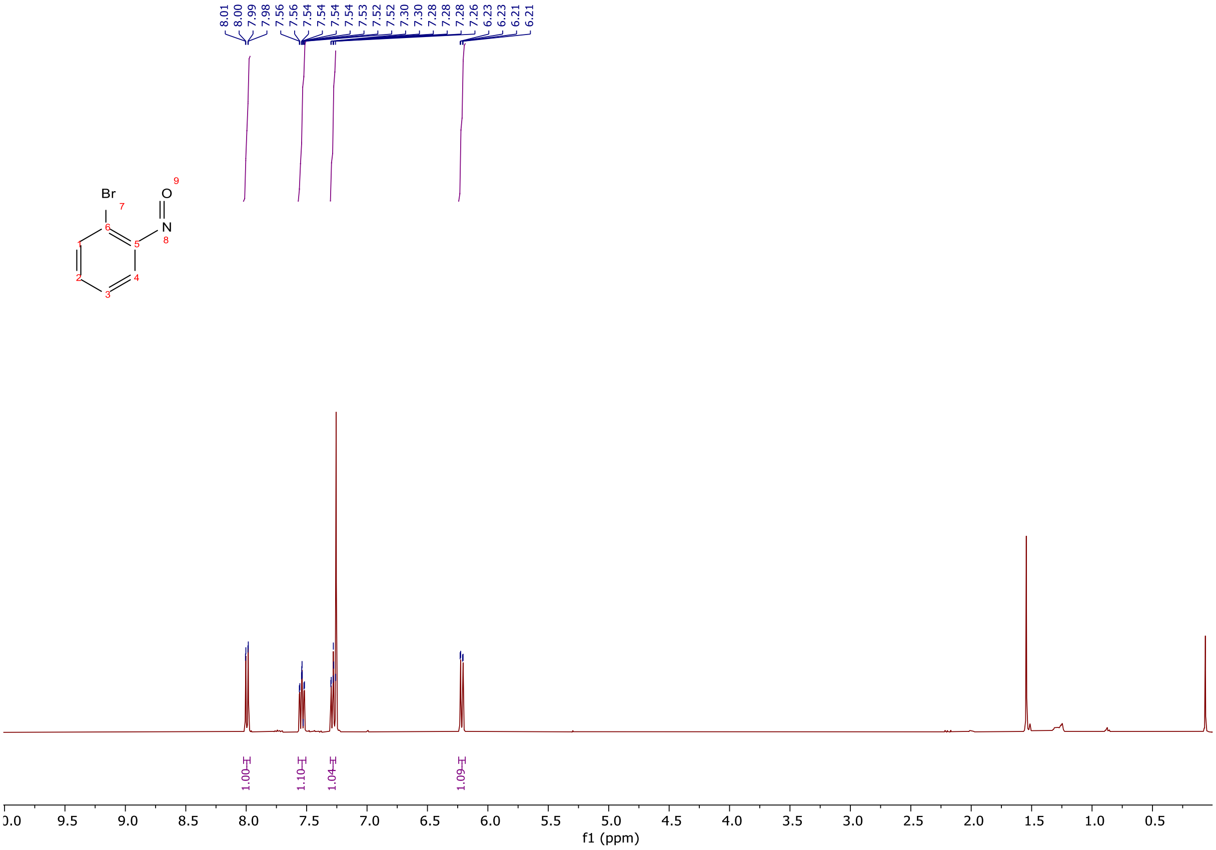

Figure S28 ^{1}H NMR spectrum of compound **5q** in $CDCl_3$.

5r: 1,3-dibromo-2-nitrosobenzene

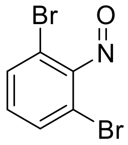

A solution of 2,6-dibromoaniline **4r** (0.30 g, 1.2 mmol, 1.0 eq) in DCM (7.0 mL) was added drop-wise to an ice-cooled solution of *m*-chloroperoxybenzoic acid (*m*CPBA) (0.25 g, 1.5 mmol, 1.2 eq) in DCM (7.0 mL). After stirring at room temperature for 5 h, a mint green mixture had formed and the precipitate of *m*CPBA that formed was filtered off. The resulting solution was washed with sat. aq. $NaHCO_3$ (50 mL), dried over $MgSO_4$, and concentrated *in vacuo*. The crude product was purified by flash chromatography (0-5% EtOAc in *n*-heptane) to give the product as yellow/orange crystals (51 mg, 0.19 mmol, 16%). ^{1}H NMR (400 MHz, $CDCl_3$) δ 7.36 (d, J = 8.0 Hz, 2H, Ar**H** (1 and 3)), 6.48 (t, J = 8.0 Hz, 1H, Ar**H** (2)). The product was used without further characterization.

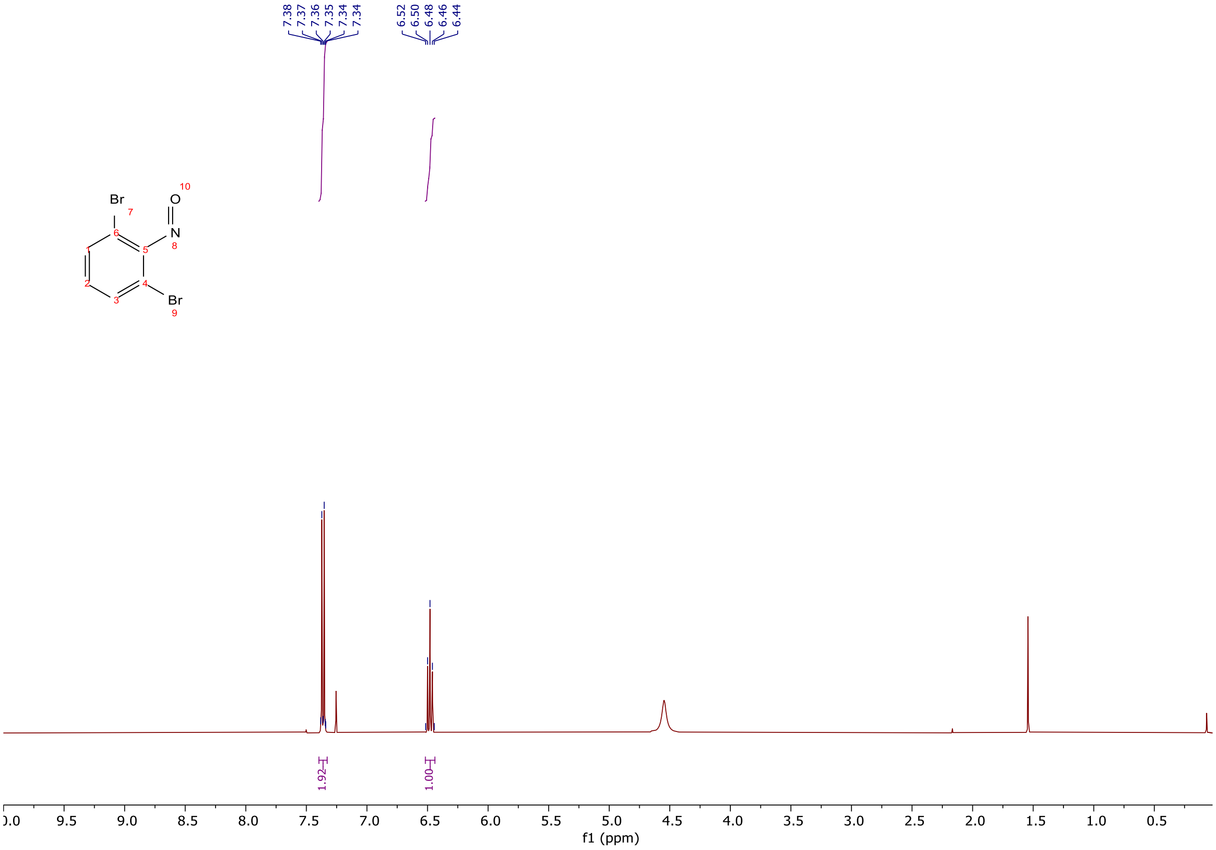

Figure S29 ^{1}H NMR spectrum of compound **5r** in $CDCl_3$.

5s: 1-chloro-2-nitrosobenzene

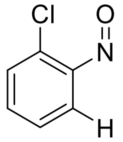

2-Chloroaniline **4s** (0.30 mL, 2.9 mmol, 1.0 eq) and Oxone (3.7 g, 6.1 mmol, 2.1 eq) were dissolved in DCM/H_2O (1:5, v/v, 30 mL). The reaction mixture was stirred vigorously at room temperature under a nitrogen atmosphere. After 24 h, TLC indicated complete consumption of the starting material. Then, DCM (20 mL) was added, and the layers were separated. The aqueous layer was extracted with DCM (2 x 50), and the combined organic layers were washed with aq. 3 N HCl (40 mL), sat. aq. $NaHCO_3$ (50 mL) and water (50 mL), dried over $MgSO_4$, and the solvents evaporated. The product was purified by flash chromatography (Silicagel 40-63 nm, 0-5% EtOAc in *n*-heptane) to give the product as brown crystals (0.37 g, 2.6 mmol, 93%). ^{1}H NMR (400 MHz, $CDCl_3$) δ 7.79 (dd, J = 8.1, 1.2 Hz, 1H Ar**H** (4)), 7.63 (app td, J = 8.1, 1.7 Hz, 1H, Ar**H** (3)), 7.28 – 7.20 (m, 1H, Ar**H** (2)), 6.24 (dd, J = 8.1, 1.7 Hz, 1H, Ar**H** (1)). ^{1}H NMR spectrum corresponds to literature.[21, 24]

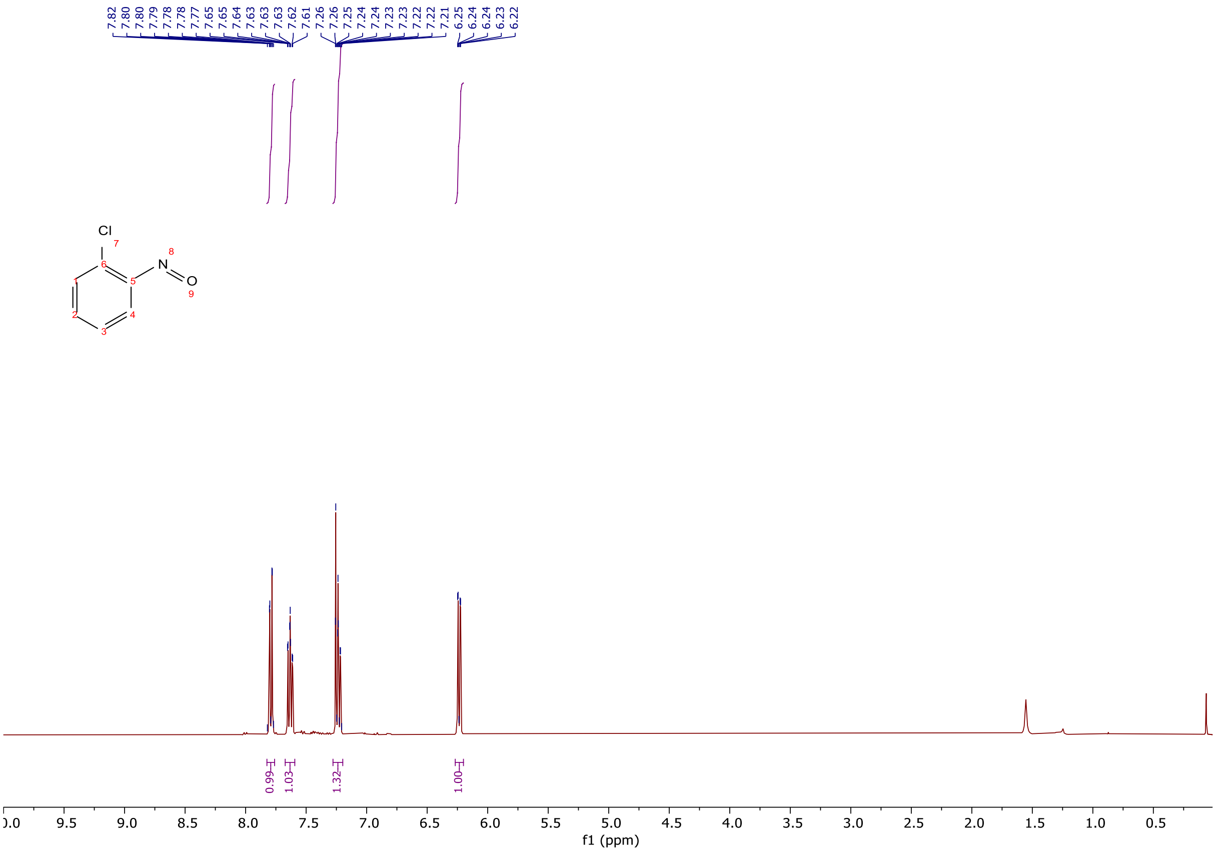

Figure S30 ^{1}H NMR spectrum of compound **5s** in $CDCl_3$.

5u: 1-fluoro-2-nitrosobenzene

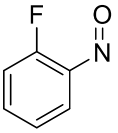

2-Fluoroaniline **4u** (0.43 mL, 4.5 mmol, 1.0 eq) and Oxone (2.8 g, 9.1 mmol, 2.0 eq) were dissolved in DCM/H_2O (1:3, v/v, 20 mL). The reaction mixture was stirred vigorously at room temperature under nitrogen atmosphere. After 4 h, TLC indicated complete consumption of the starting material Then, DCM (20 mL) was added, and the layers were separated. The aqueous layer was extracted with DCM (2 x 20 mL) and the combined organic layers were washed with aq. 1 N HCl (25 mL), sat. aq. $NaHCO_3$ (20 mL), and brine (20 mL), dried over $MgSO_4$, and concentrated *in vacuo*. The product was purified by flash chromatography (Silicagel 40-63 nm, 0-5% EtOAc in pentane). The product was obtained as a viscous oil (0.16 g, 1.3 mmol, 28 %). ^{1}H NMR (400 MHz, $CDCl_3$) δ 7.72 (m, J = 7.2 Hz, 1H, Ar**H**), 7.51 (t, J = 9.3 Hz, 1H, Ar**H**), 7.14 (t, J = 7.7 Hz, 1H, Ar**H**), 6.49 (t, J = 7.5 Hz, 1H, Ar**H**). ^{1}H NMR spectrum corresponds to literature.[21]

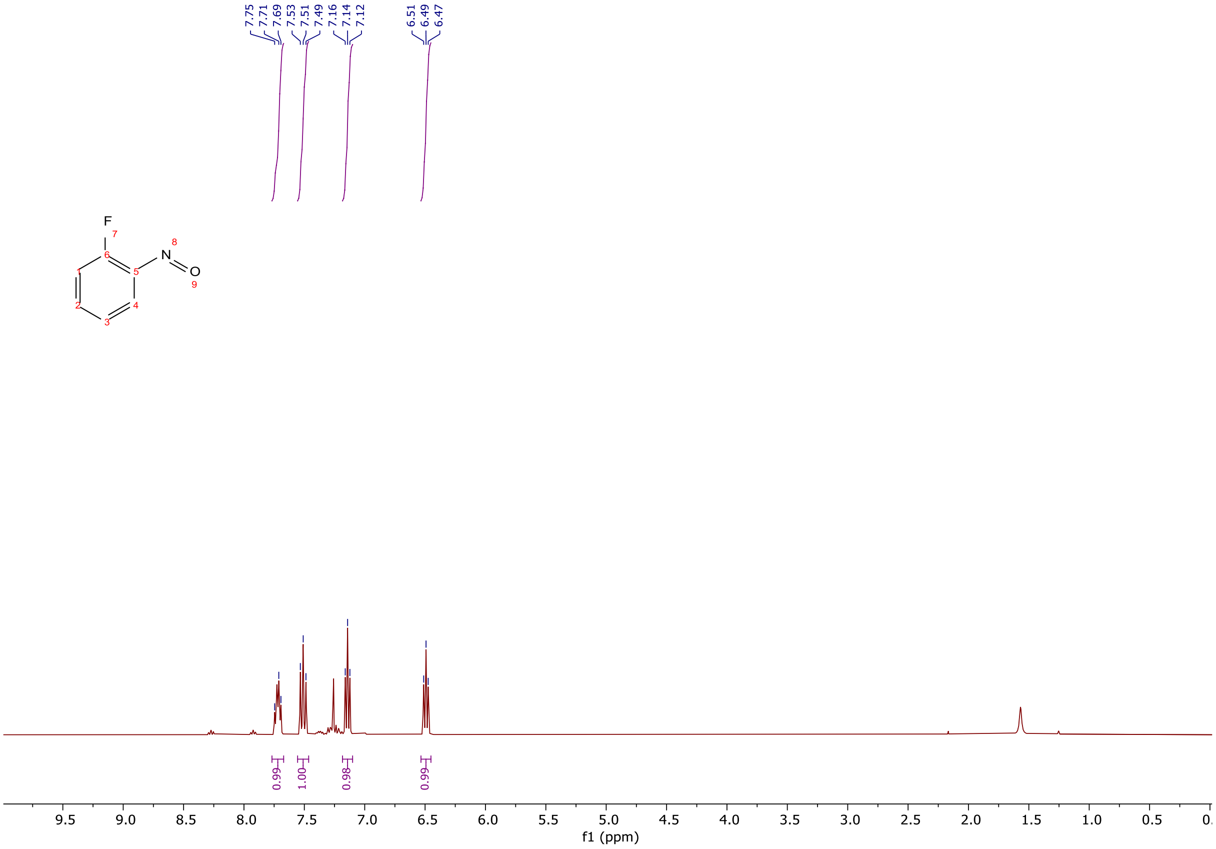

Figure S31 ^{1}H NMR spectrum of compound **5u** in $CDCl_3$.

5w: 1-nitroso-2-(trifluoromethyl)benzene

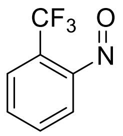

2-(trifluoromethyl)aniline **4w** (0.50 mL, 4.0 mmol, 1.0 eq) in DCM (21 mL) was added portion-wise to an ice-cooled solution of *m*CPBA (1.4 g, 8.0 mmol, 2.0 eq) in HFIP (21 mL). After stirring at room temperature for 4 h, an olive-green mixture had formed and the precipitate of *m*CPBA that formed was filtered off. The resulting solution was washed with sat. aq. $NaHCO_3$ (2 x 25 mL), dried over $MgSO_4$, and concentrated *in vacuo*. The crude product was purified by flash chromatography (0-5% EtOAc in *n*-heptane) to give the product as an olive-green oil (36 mg, 0.21 mmol, 5%). ^{1}H NMR (400 MHz, $CDCl_3$) δ 8.06 (d, J = 7.8 Hz, 1H, Ar**H**), 7.82 (t, J = 7.6 Hz, 1H, Ar**H**), 7.63 – 7.54 (t, 1H, Ar**H**), 6.25 (d, J = 8.0 Hz, 1H, Ar**H**). ^{1}H spectra correspond to literature.[25]

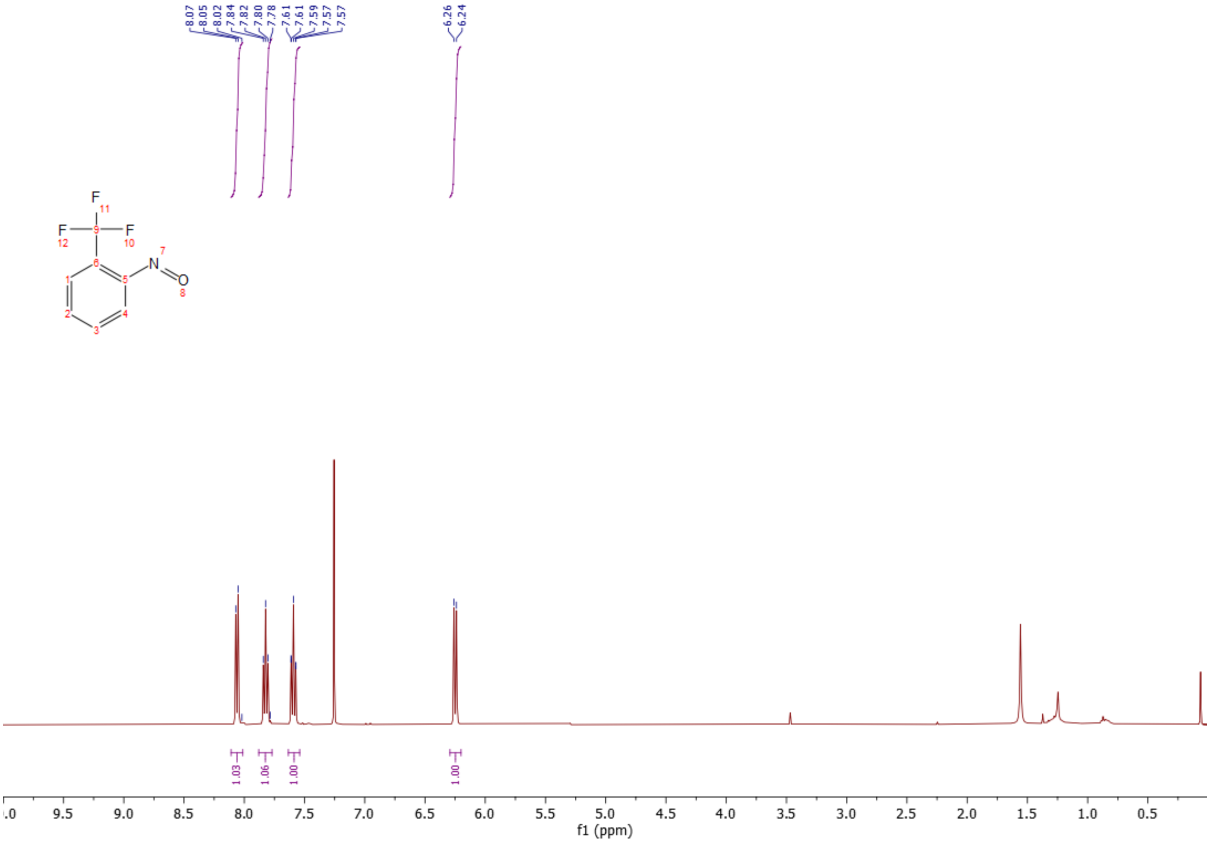

Figure S32 ^{1}H NMR spectrum of compound **5w** in $CDCl_3$.

5x: 1,3,5-trifluoro-2-nitrosobenzene

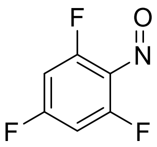

2,4,6-Trifluoroaniline **4x** (0.30 g, 2.1 mmol, 1.0 eq) in DCM (5.0 mL) was added portion-wise to an ice-cooled solution of *m*CPBA (0.75 g, 4.4 mmol, 2.0 eq) in HFIP (5.0 mL). After stirring at room temperature for 12 h, a yellow mixture had formed and the precipitate of *m*CPBA that formed was filtered off. The resulting solution was washed with sat. aq. $NaHCO_3$ (2 x 50 mL), dried over $MgSO_4$, and concentrated *in vacuo* to give the product as brown crystals. The product was used without further purification. ^{1}H NMR (400 MHz, $CDCl_3$) δ 6.91 – 6.76 (m, 2H). ^{19}F NMR (376 MHz, $CDCl_3$) δ -91.8 (m, J = 12.9, 8.4 Hz), -125.1 (m). ^{1}H and ^{19}F spectra correspond to literature.[26]

5y: 1,3-difluoro-5-methoxy-2-nitrosobenzene

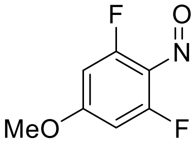

2,6-Difluoro-4-methoxyaniline **4y** (0.56 g, 3.2 mmol, 1.0 eq) in DCM (4.0 mL) was added portion-wise to an ice-cooled solution of *m*CPBA (1.4 g, 8.0 mmol, 2.0 eq) in HFIP (4.0 mL). After stirring at room temperature for 4 h, a yellow mixture had formed and the precipitate of *m*CPBA that formed was filtered off. The resulting solution was washed with sat. aq. $NaHCO_3$ (2 x 50 mL), dried over $MgSO_4$, and concentrated *in vacuo*. The crude product was purified by flash chromatography (0-5% EtOAc in *n*-heptane) to give the product as orange crystals (47 mg, 0.27 mmol, 17%). ^{1}H NMR (400 MHz, $CDCl_3$) δ 6.61 – 6.50 (m, 2H, Ar**H**), 3.94 (s, 3H, C**H**$_3$). The product was used without further characterization.

5z: 1,2,3,4,5-pentafluoro-6-nitrosobenzene

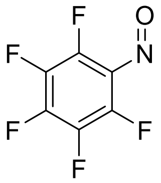

2,3,4,5,6-Pentafluoroaniline (2.0 g, 10.9 mmol, 1.0 eq) and Oxone (14 g, 23 mmol, 2.1 eq) were dissolved in DCM/H_2O (1:3, v/v, 100 mL). The reaction mixture was stirred at room temperature under a nitrogen atmosphere. After 24 h, TLC indicated complete consumption of the starting material. Then, DCM (25 mL) was added, and the layers were separated. The aqueous layer was extracted with DCM (3 x 50 mL), and the combined organic layers were washed with aq. 3 N HCl (2 x 40 mL), sat. aq. $NaHCO_3$ (2 x 60 mL), water (3 x 40 mL), dried over $MgSO_4$, and the solvents evaporated to give the crude product as brown/green crystals. ^{19}F NMR (376 MHz, $CDCl_3$) δ -141.7 (tt, *J* = 20.8, 7.5 Hz), -159.5 (m), 160.3 (m). The product was used without further purification and characterization.

5β: 1,2,3,4,5-pentachloro-6-nitrosobenzene

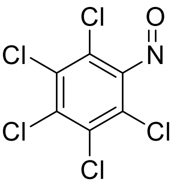

2,3,4,5,6-Pentachlooroaniline **4β** (50 mg, 0.19 mmol, 1.0 eq) in DCM (3.0 mL) was added portion-wise to an ice-cooled solution of *m*CPBA (70 mg, 0.41 mmol, 2.2 eq) in HFIP (3.0 mL). After stirring at room temperature for 8 h, TLC indicated completion and the precipitate of *m*CPBA that formed was filtered off. The resulting solution was washed with sat. aq. $NaHCO_3$ (2 x 25 mL), dried over $MgSO_4$, and concentrated *in vacuo*. The product was used without further purification and characterization.

1i: (*Z*)-2-((4-fluorophenyl)imino)benzo[b]thiophen-3(2H)-one

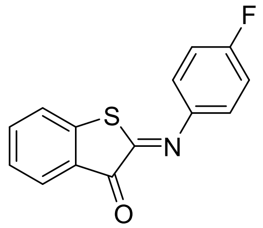

Benzo[b]thiophen-3-yl acetate **3** (70 mg, 0.37 mmol) and crude 1-fluoro-4-nitrosobenzene **5g** were dissolved in EtOH (2 mL). The mixture was cooled on an ice-water bath. KOH (25 g/L in EtOH) was added dropwise (10 drops) and the mixture was allowed to reach room temperature and was stirred vigorously. After 1 h, TLC indicated complete consumption of starting material. Then, Et_2O (50 mL) and H_2O (50 mL) were added, the layers were separated, and the aqueous layer was extracted with Et_2O (3 x 50 mL). The combined organic layers were washed with sat. aq. $NaHCO_3$, water and brine, dried using $MgSO_4$ and concentrated *in vacuo*. The product was purified with flash chromatography (Silicagel 40 - 63 nm, Toluene). The product was obtained as an orange solid (51 mg, 0.20 mmol, 53% yield). Mp: 141 - 143 °C. ^{1}H NMR (400 MHz, $CDCl_3$) δ 7.94 (d, *J* = 7.6 Hz, 1H, Ar**H** (3)), 7.61 (t, *J* = 7.6 Hz, 1H, Ar**H** (2)), 7.40 (d, *J* = 7.8 Hz, 1H, Ar**H** (6)), 7.34 (d, *J* = 7.4 Hz, 1H, Ar**H** (1)), 7.25 – 7.31 (m, 2H, Ar**H** (13 and 17)), 7.14 (t, *J* = 8.6 Hz, 2H, Ar**H** (14 and 16)). ^{13}C NMR (101 MHz, $CDCl_3$) δ 185.4, 162.9, 160.4, 156.0 (d, *J* = 2.0 Hz), 145.1 (d, *J* = 3.0 Hz), 144.1, 137.0, 127.8, 127.7, 126.8, 125.0, 123.4 (d, *J* = 9.0 Hz), 116.3 (d, *J* = 23.2 Hz). ^{19}F NMR (376 MHz, $CDCl_3$) δ -113.7 (septet, *J* = 13.3, 8.4, 4.8 Hz). HRMS (ESI+) calc. for. $[M+Na]^+$ ($C_{14}H_9FNOSNa^+$) 280.0203 found: 280.0207.

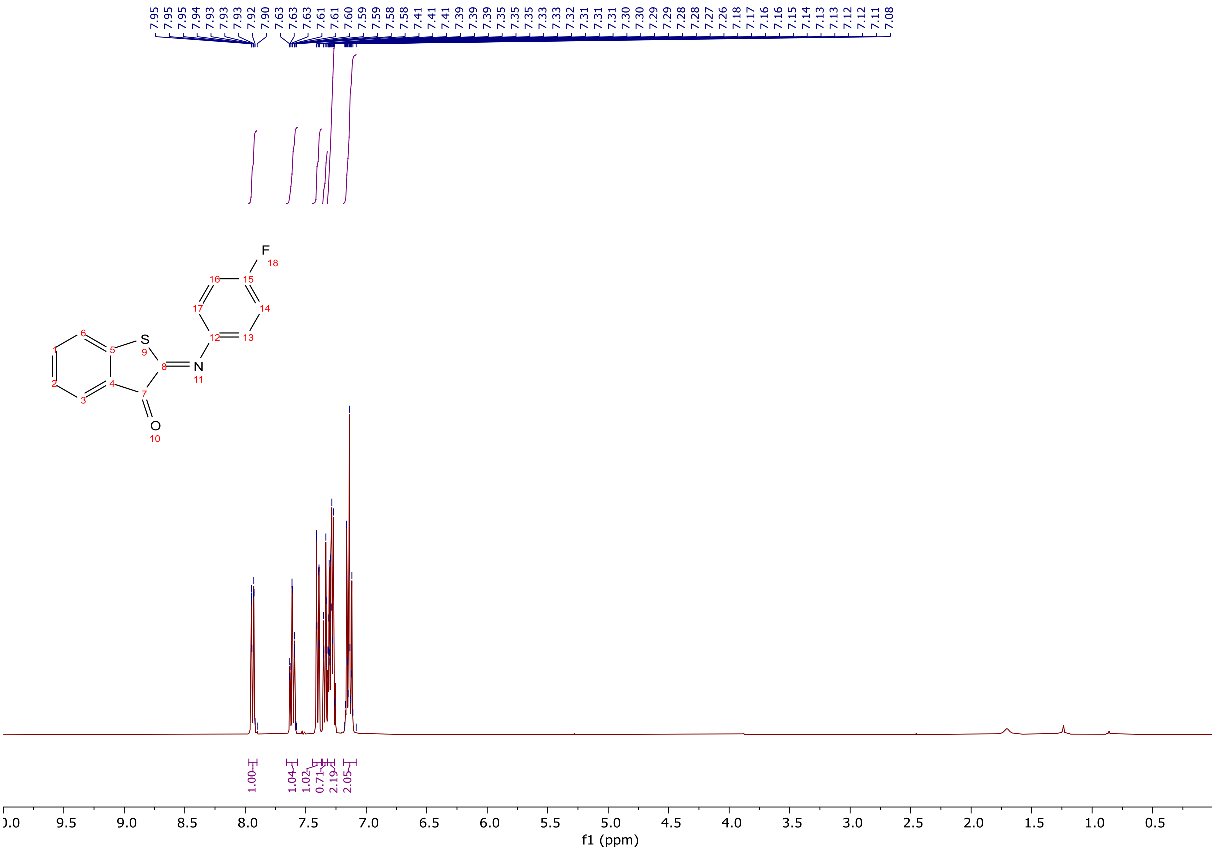

Figure S33 ^{1}H NMR spectrum of compound **1i** in $CDCl_3$.

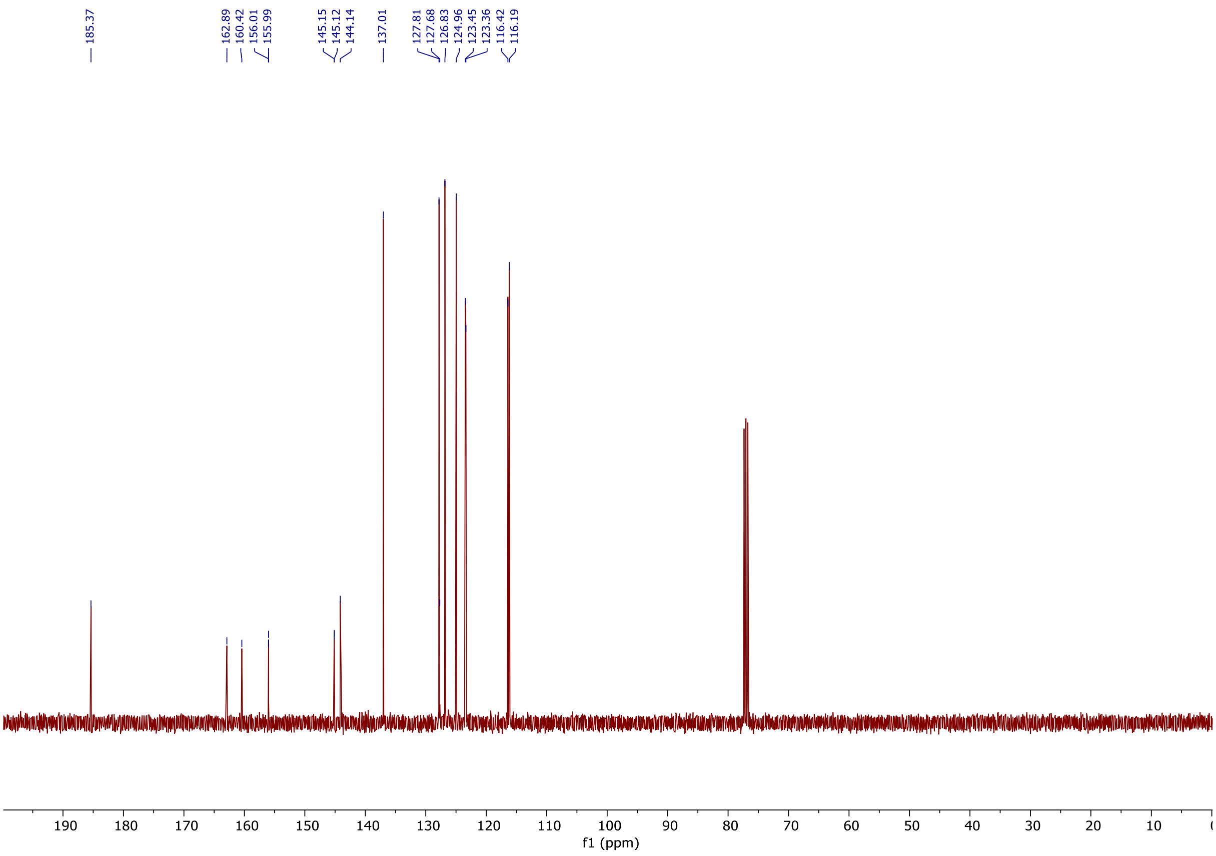

Figure S34 ^{13}C NMR spectrum of compound **1i** in $CDCl_3$.

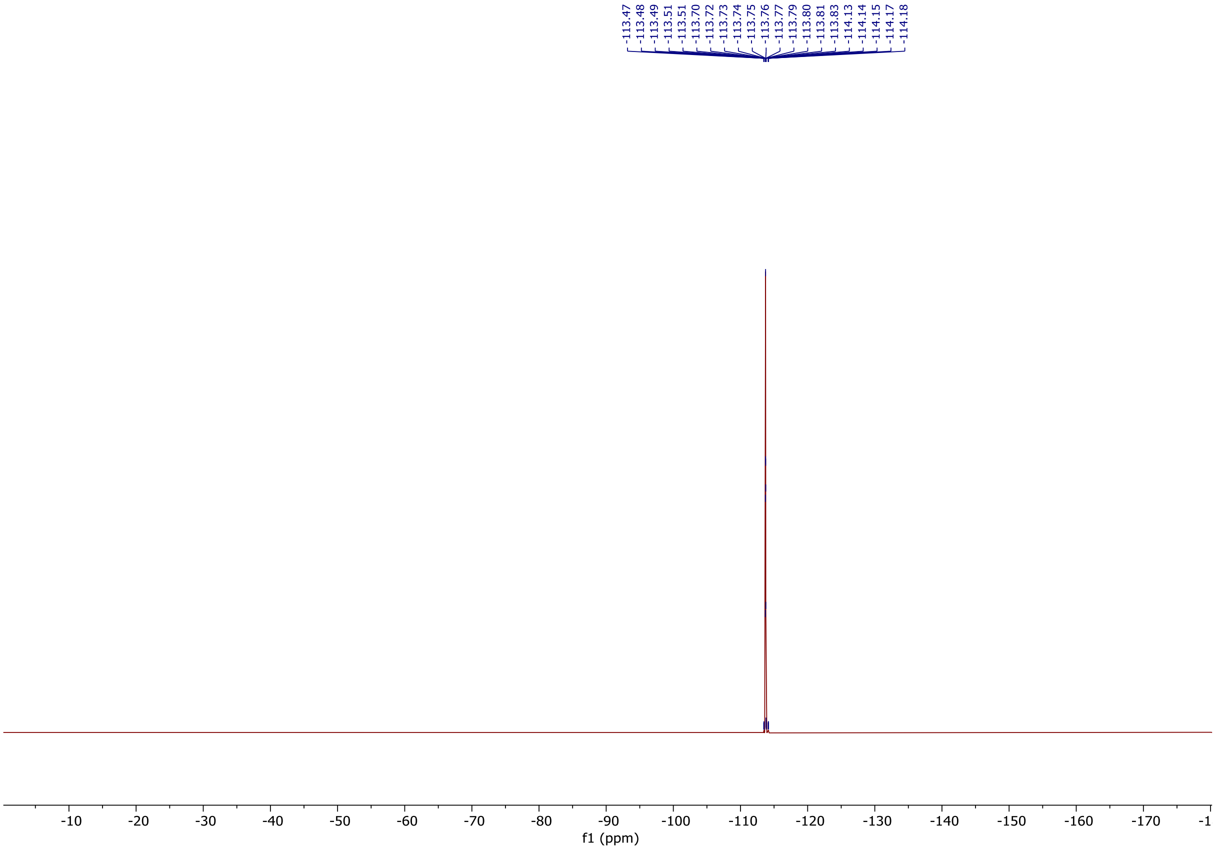

Figure S35 ^{19}F NMR spectrum of compound **1i** in $CDCl_3$.

1k: (*Z*)-2-((4-(dimethylamino)phenyl)imino)benzo[b]thiophen-3(2H)-one

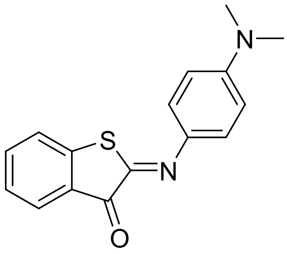

Benzo[b]thiophen-3-yl acetate **8a** (75 mg, 0.39 mmol, 1.0 eq) and *N*,*N*-dimethyl-4-nitrosobenzene **5k** (70 mg, 0.39 mmol, 1.0 eq) were dissolved in EtOH (2 mL). The mixture was cooled on an ice-water bath. KOH (25 g/L in EtOH) was added dropwise (10 drops) and the mixture was allowed to reach room temperature and stirred vigorously. After 1 h, TLC indicated completion. Then, Et_2O (50 mL) and H_2O (50 mL) were added, the layers were separated, and the aqueous layer was extracted with Et_2O (3 x 50 mL). The combined organic layers were washed with sat. aq. $NaHCO_3$, water and brine, dried using $MgSO_4$ and concentrated *in vacuo*. The product was purified by flash chromatography (Silicagel 40-63 nm, 0-10% EtOAc in toluene). The product was obtained as a purple solid (60 mg, 0.21 mmol, 54% yield). Mp: 162 - 163 °C. ^1H NMR (400 MHz, $CDCl_3$) δ 7.95 (d, *J* = 6.0 Hz, 1H, Ar**H** (3)), 7.57 (t, *J* = 7.6 Hz, 1H, Ar**H** (2)), 7.52 (d, *J* = 7.2 Hz, 2H, Ar**H** (13 and 17)), 7.44 (d, *J* = 7.8 Hz, 1H, Ar**H** (6)), 7.30 (t, *J* = 8.5 Hz, 1H, Ar**H** (1)), 6.78 (d, *J* = 7.8 Hz, 2H, Ar**H** (14 and 16)), 3.06 (s, 6H, 2 x C**H**$_3$ (19 and 20)). ^{13}C NMR (101 MHz, $CDCl_3$) δ 186.0, 150.6, 146.7, 144.5, 135.9, 135.8, 128.2, 127.3, 126.7, 126.3, 124.6, 112.0, 40.2. IR (cm^{-1}): 1697 (C=O, stretch), 1612 (N-H, bend), 1593 (C-C, stretch), 1521 (C=C, stretch), 1448 (C-C, stretch), 1368 (C-H, rock), 1285 (C-N, stretch), 1172 (C-N, stretch), 1029 (C-N, stretch), 1014 (C-N, stretch). IR, ^1H and ^{13}C NMR spectra correspond to literature.[27]

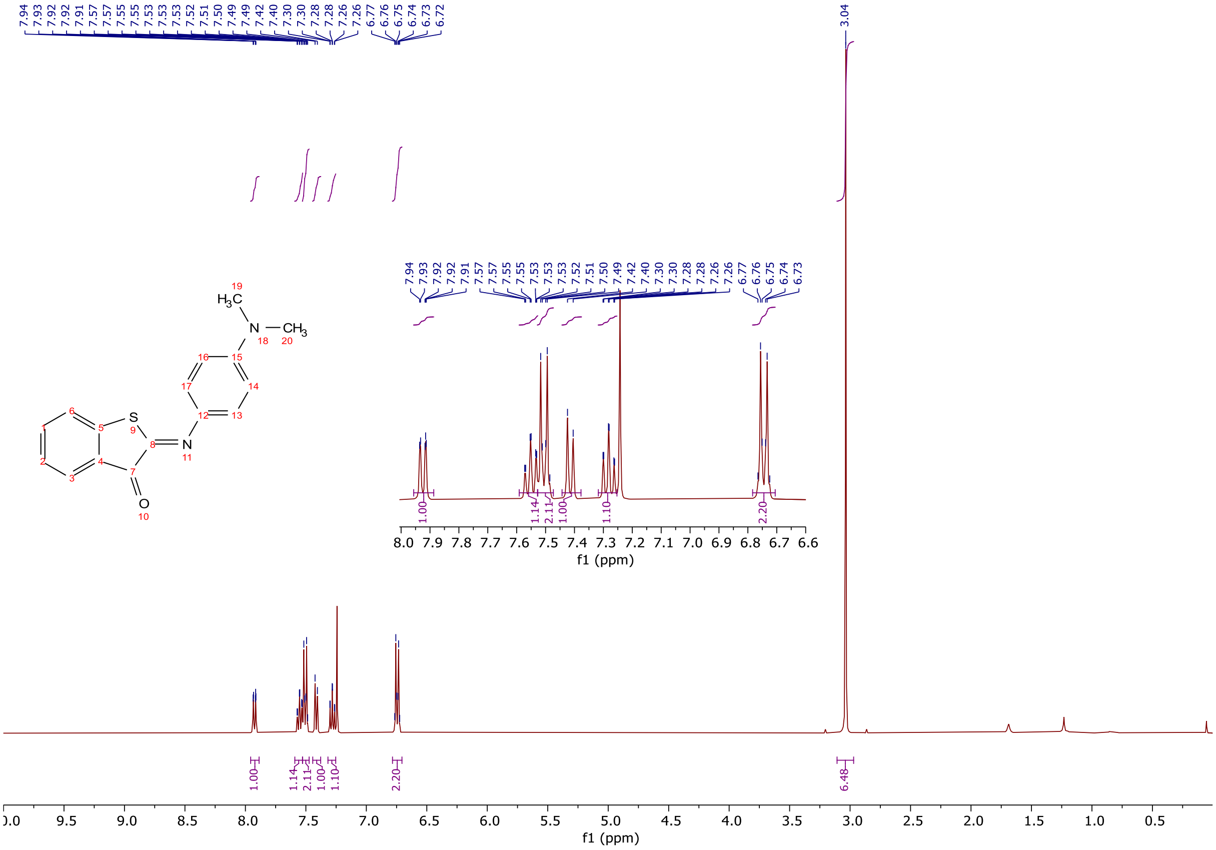

Figure S36 ^{1}H NMR spectrum of compound **1k** in $CDCl_3$.

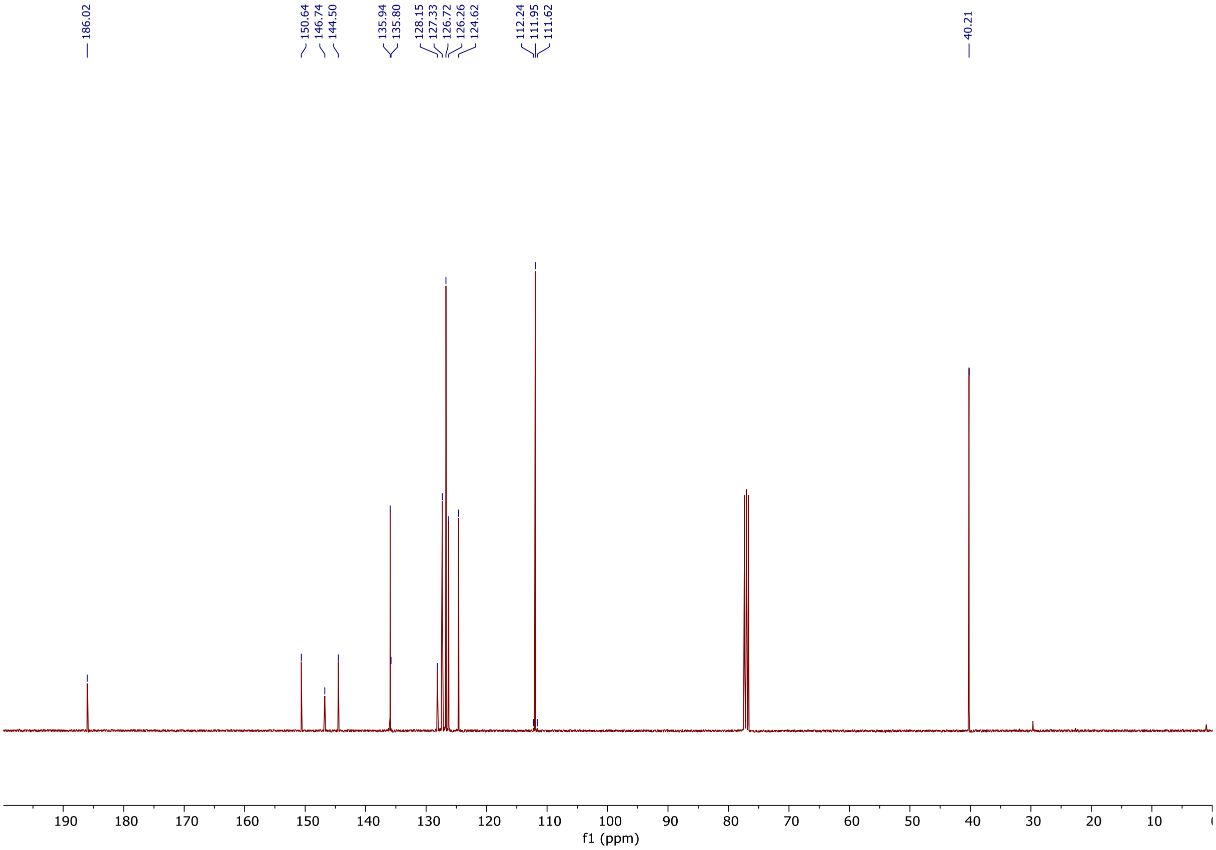

Figure S37 ^{13}C NMR spectrum of compound **1k** in $CDCl_3$.

1l: (*Z*)-2-((2-methoxyphenyl)imino)benzo[b]thiophen-3(2H)-one

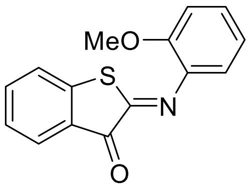

Benzo[b]thiophen-3-yl acetate **8a** (39 mg, 0.20 mmol, 1.0 eq) and 2-methoxynitrosobenzene **5l** (35 mg, 0.26 mmol, 1.3 eq) were dissolved in ethanol (4 mL). The mixture was cooled in an ice-water bath. KOH (25 g/L in EtOH, 0.2 mL, 0.093 mmol, 0.36 eq) was added dropwise and the mixture was allowed to reach room temperature and stirred vigorously. After 15 min, TLC indicated completion. Then, DCM (30 mL) was added, and the mixture washed with brine (50 mL). The aqueous layer was extracted with DCM (2 x 25 mL) and the combined organic layers were dried over $MgSO_4$ and concentrated *in vacuo*. The crude product was purified by flash chromatography (Silicagel 40 – 63 nm, 0-10% EtOAc in *n*-heptane). The product was obtained as an orange solid (37 mg, 0.14 mmol, 54%). Mp: 145-146 °C. ^{1}H NMR (400 MHz, $CDCl_3$) δ 7.93 (ddd, *J* = 7.7, 1.4, 0.6 Hz, 1H, Ar**H**, 7.62 – 7.55 (m, 1H, Ar**H**), 7.38 – 7.21 (m, 3H, Ar**H**), 7.08 (dd, *J* = 7.7, 1.7 Hz, 1H, Ar**H**), 7.04 – 6.96 (m, 2H, Ar**H**), 3.85 (s, 3H, C**H**$_3$).^{13}C NMR (101 MHz, $CDCl_3$) δ 185.1, 157.6, 150.8, 144.3, 138.9, 136.8, 128.2, 128.1, 127.7, 126.6, 124.9, 120.7, 119.8, 111.9, 55.6. IR (cm^{-1}): 1706 (C=O, stretch), 1591 (C-C, stretch), 1488 (C-C, stretch), 1451 (C-H, bend), 1284 (C-N, stretch), 1250 (C-N, stretch), 740 (C=C, bend). HRMS (ESI+) calc. for. $[M+Na]^+$ ($C_{15}H_{11}NO_2SNa^+$) 292.0403, found: 292.0398.

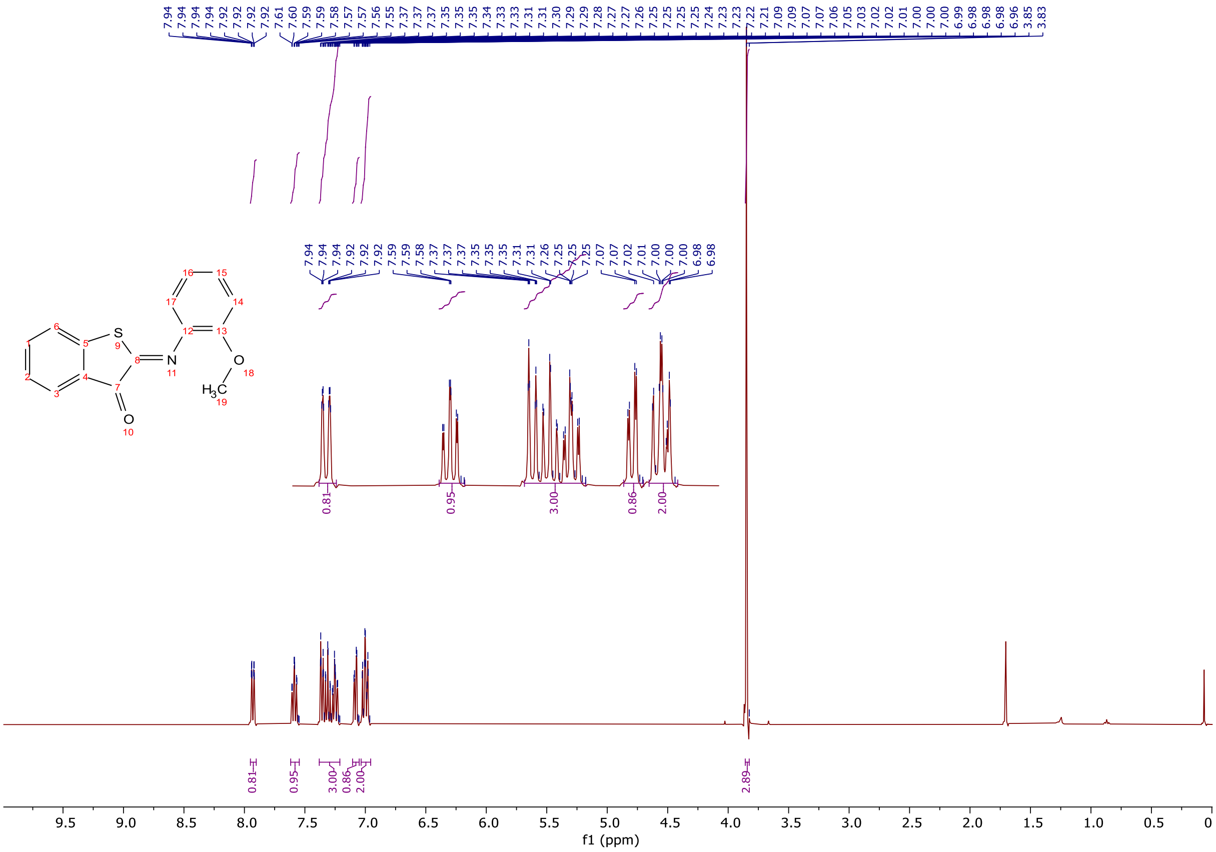

Figure S38 ^{1}H NMR spectrum of compound **1j** in $CDCl_3$.

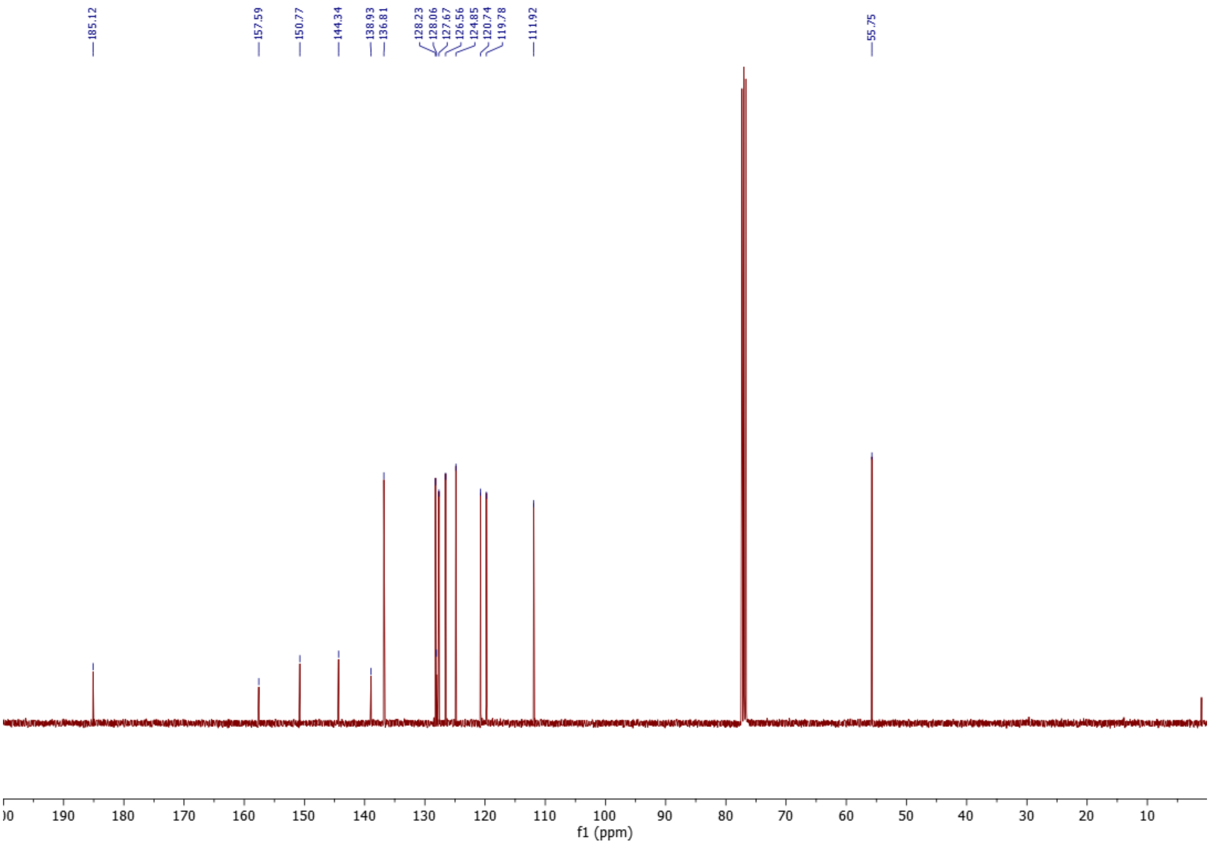

Figure S39 ^{13}C NMR spectrum of compound **1j** in $CDCl_3$.

1m: (*Z*)-2-(o-tolylimino)benzo[b]thiophen-3(2H)-one

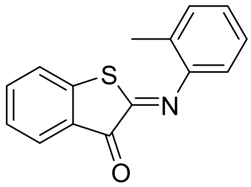

Benzo[*b*]thiophen-3-yl acetate **8a** (50 mg, 0.26 mmol, 1.0 eq) and 2-methylnitrosobenzene **5m** (56 mg, 0.41 mmol, 1.6 eq) were dissolved in ethanol (2 mL). This mixture was cooled on an ice-water bath. KOH (25 g/L in EtOH) was added dropwise (7 drops) and the mixture was allowed to reach room temperature and stirred vigorously. After 10 min, TLC indicated completion. Then, the product was filtered off and washed with water (1 x 20 mL). The product was dissolved in acetone, dried over $MgSO_4$ and concentrated *in vacuo*. The product was obtained as an orange solid (48 mg, 0.19 mmol, 73%). Mp: 130 – 132 °C. ^{1}H NMR (400 MHz, $CDCl_3$) δ 7.96 (d, J = 7.7 Hz, 1H, Ar**H** (3)), 7.61 (t, J = 7.6 Hz, 1H, Ar**H** (2)), 7.35 (dd, J = 18.4, 7.7 Hz, 2H, Ar**H** (15 and 16)), 7.30 – 7.22 (m, 2H, Ar**H** (13 and 14)), 7.18 (t, J = 7.4 Hz, 1H, Ar**H** (1)), 6.97 (d, J = 7.7 Hz, 1H, Ar**H** (6)), 2.26 (s, 3H, C**H**$_3$ (18)).^{13}C NMR (101 MHz, $CDCl_3$) δ 185.1, 157.2, 149.2, 144.5, 137.0, 130.8, 130.2, 128.1, 127.7, 126.8, 126.6, 126.6, 125.0, 117.3, 29.7. ^{1}H spectrum corresponds to literature.[28] HRMS (ESI+) calc. for. $[M+H]^+$ ($C_{15}H_{11}NOS^+$) Exact Mass: 254.0569, found: 254.0634.

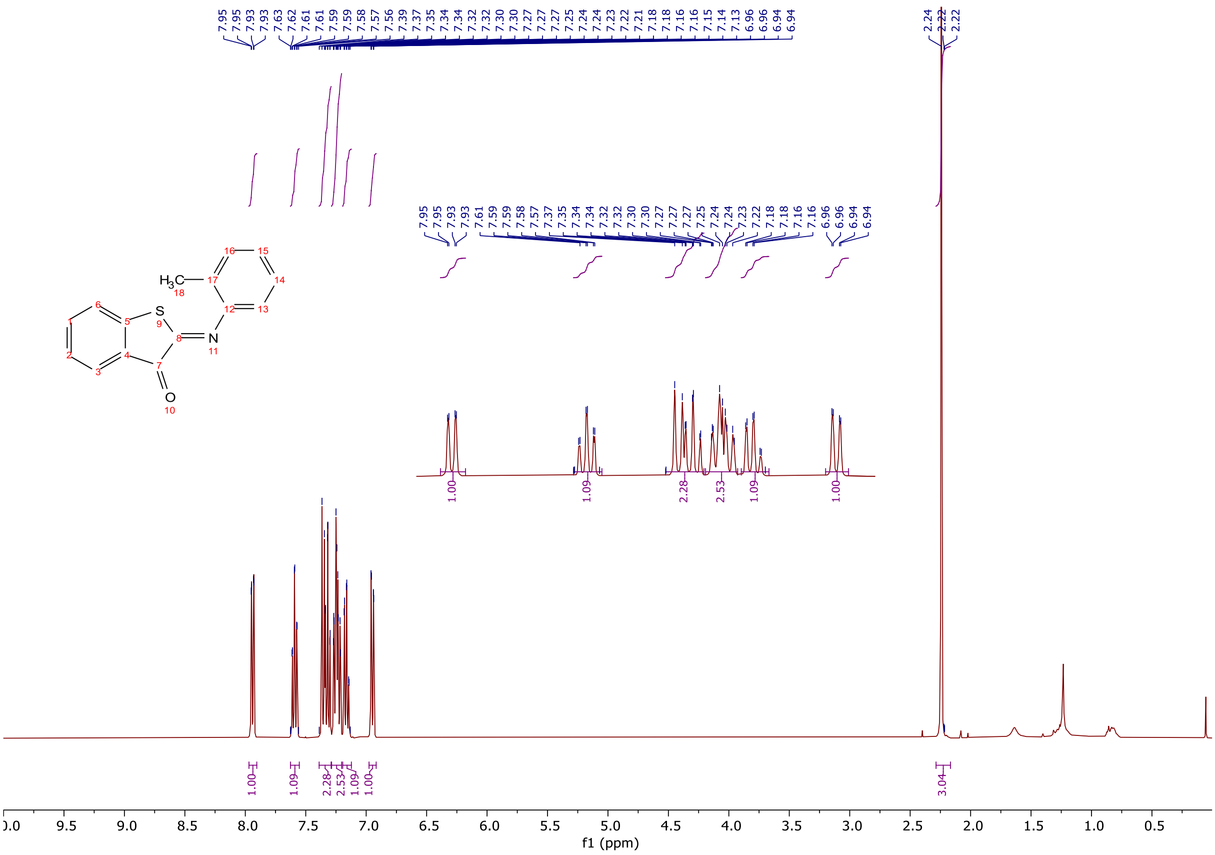

Figure S40 ^{1}H NMR spectrum of compound **1m** in $CDCl_3$.

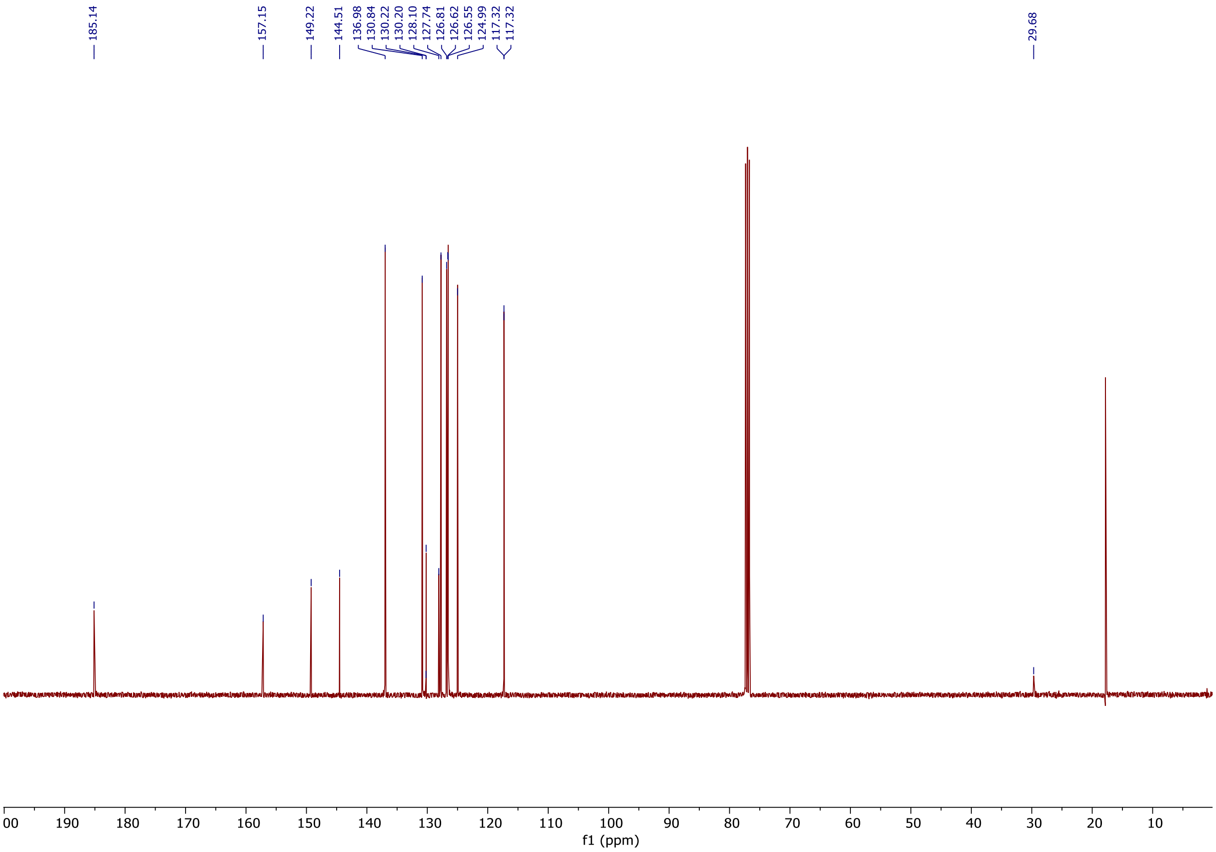

Figure S41 ^{13}C NMR spectrum of compound **1m** in $CDCl_3$.

1n: (*Z*)-2-((2,6-dimethylphenyl)imino)benzo[b]thiophen-3(2H)-one

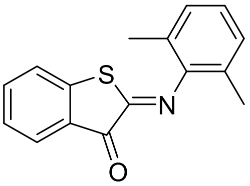

Benzo[*b*]thiophen-3-yl acetate (66 mg, 0.34 mmol, 1.0 eq) **8a** and 2,6-dimethylnitrosobenzene **5n** (71 mg, 0.53 mmol, 1.6 eq) were dissolved in ethanol (2 mL). The mixture was cooled on an ice-water bath. KOH (25mg/mL in EtOH) was added dropwise (8 drops) and the mixture was allowed to reach room temperature and stirred vigorously. After 4.5 h, TLC indicated completion. Then, DCM (30 mL) and water (30 mL) were added, and the layers were separated. The aqueous layers were extracted with DCM (2 x 20 mL). The combined organic layers were washed with sat. aq. $NaHCO_3$ (25 mL), brine (25 mL), dried over $MgSO_4$, and concentrated *in vacuo*. The product was purified by flash chromatography (Silicagel 40 – 63 nm, 0-5% EtOAc in pentane). The product was obtained as an orange oil (13 mg, 0.048 mmol, 14%). ^{1}H NMR (400 MHz, $CDCl_3$) *δ* 7.96 (d, *J* = 7.6 Hz, 1H, Ar**H** (3)), 7.61 (t, *J* = 7.6 Hz, 1H, Ar**H** (2)), 7.38 – 7.30 (m, 2H, Ar**H** (1 and 6)), 7.12 – 7.00 (m, 3H, Ar**H** (14, 15, and 16)), 2.10 (s, 6H, 2 x C**H**$_3$ (18 and 19)).^{13}C NMR (101 MHz, $CDCl_3$) *δ* 184.8, 159.6, 149.4, 143.9, 137.3, 128.4, 128.3, 127.8, 126.7, 125.2, 125.2, 125.0, 17.8. HRMS (ESI+) calc. for $[M+H]^+$ ($C_{16}H_{13}NOS^+$) 268.0726 found: 268.0791.

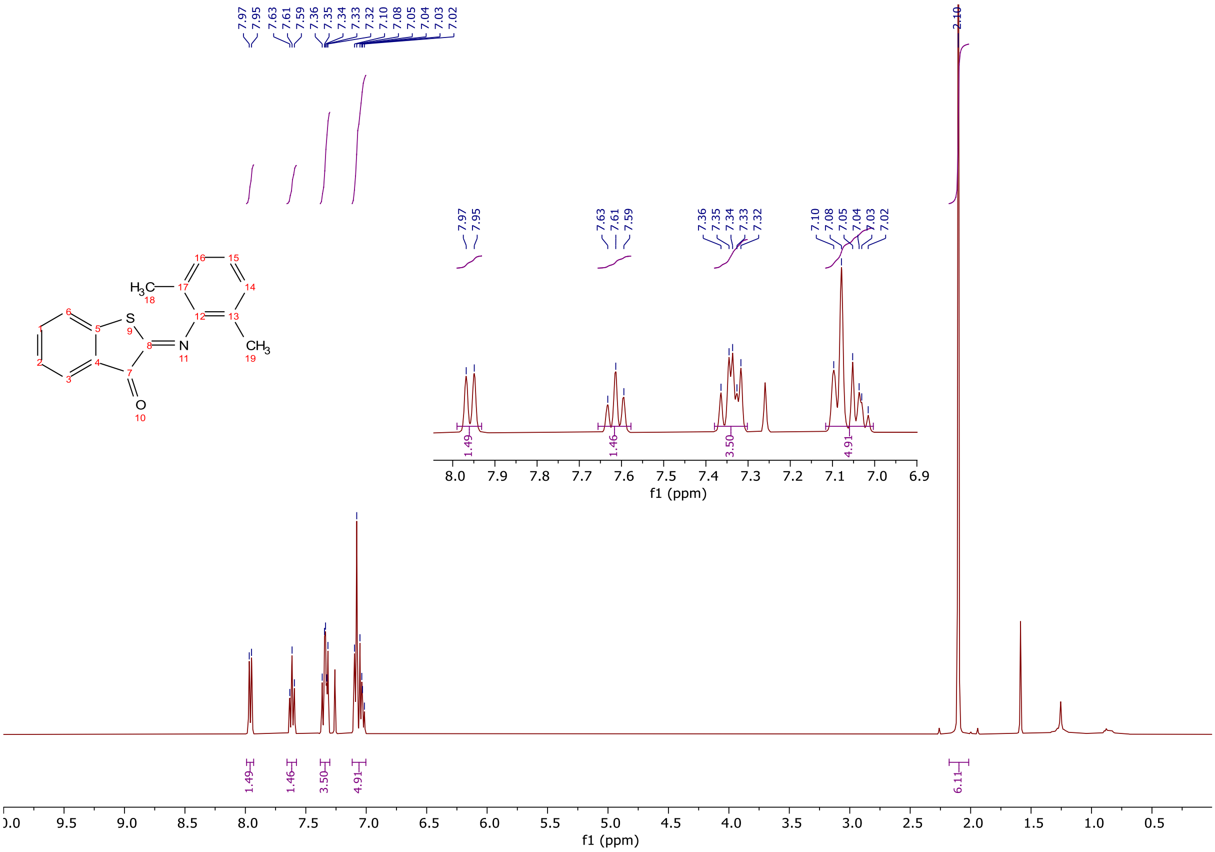

Figure S42 ^{1}H NMR spectrum of compound **1n** in $CDCl_3$.

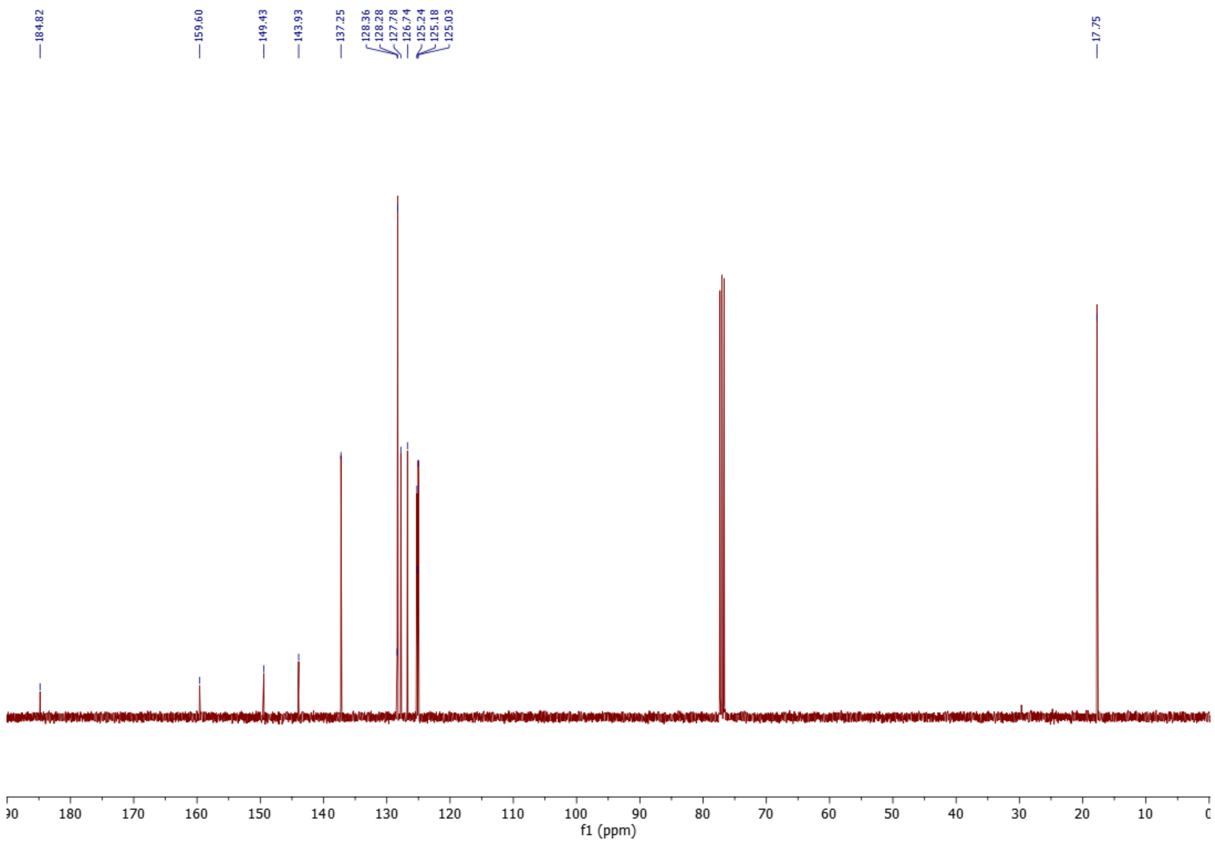

Figure S43 ^{13}C NMR spectrum of compound **1n** in $CDCl_3$.

1o: (*Z*)-2-((2-ethylphenyl)imino)benzo[b]thiophen-3(2H)-one

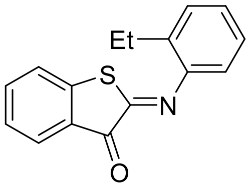

Benzo[b]thiophen-3-yl acetate **8a** (56 mg, 0.29 mmol, 1.0 eq) and 2-ethylnitrosobenzene **5o** (51 mg, 0.38 mmol, 1.3 eq) were dissolved in ethanol (6 mL). The mixture was cooled in an ice-water bath. KOH (25 g/L in EtOH, 0.31 mL, 0.14 mmol, 0.36 eq) was added dropwise and the mixture was allowed to reach room temperature and stirred vigorously. After 2 h, TLC indicated completion. Then, DCM (30 mL) was added, and the mixture washed with brine (50 mL). The aqueous layer was extracted with DCM (2 x 25 mL) and the combined organic layers were dried over $MgSO_4$ and concentrated *in vacuo*. The crude product was purified by flash chromatography (Silicagel 40 – 63 nm, 0-10% EtOAc in *n*-heptane). The product was obtained as an orange solid (86 mg, 0.32 mmol, 85%). Mp: 68-70 °C. ^{1}H NMR (400 MHz, $CDCl_3$) δ 7.98 – 7.92 (m, 1H, Ar**H**), 7.60 (app td, *J* = 7.6, 1.4 Hz, 1H, Ar**H**), 7.40 – 7.28 (m, 3H, Ar**H**), 7.28 – 7.18 (m, 2H, Ar**H**), 6.95 (dd, *J* = 7.4, 1.8 Hz, 1H, Ar**H**), 2.64 (q, *J* = 7.5 Hz, 2H, C**H**$_2$), 1.14 (t, *J* = 7.5 Hz, 3H, C**H**$_3$). ^{13}C NMR (101 MHz, $CDCl_3$) δ 185.1, 157.2, 148.7, 144.6, 137.0, 136.6, 129.1, 128.1, 127.7, 127.1, 126.6, 126.6, 125.0, 117.4, 24.6, 14.8. HRMS (ESI+) calc. for $[M+H]^+$ ($C_{16}H_{14}NOS^+$) 268.0791, found: 268.0786.

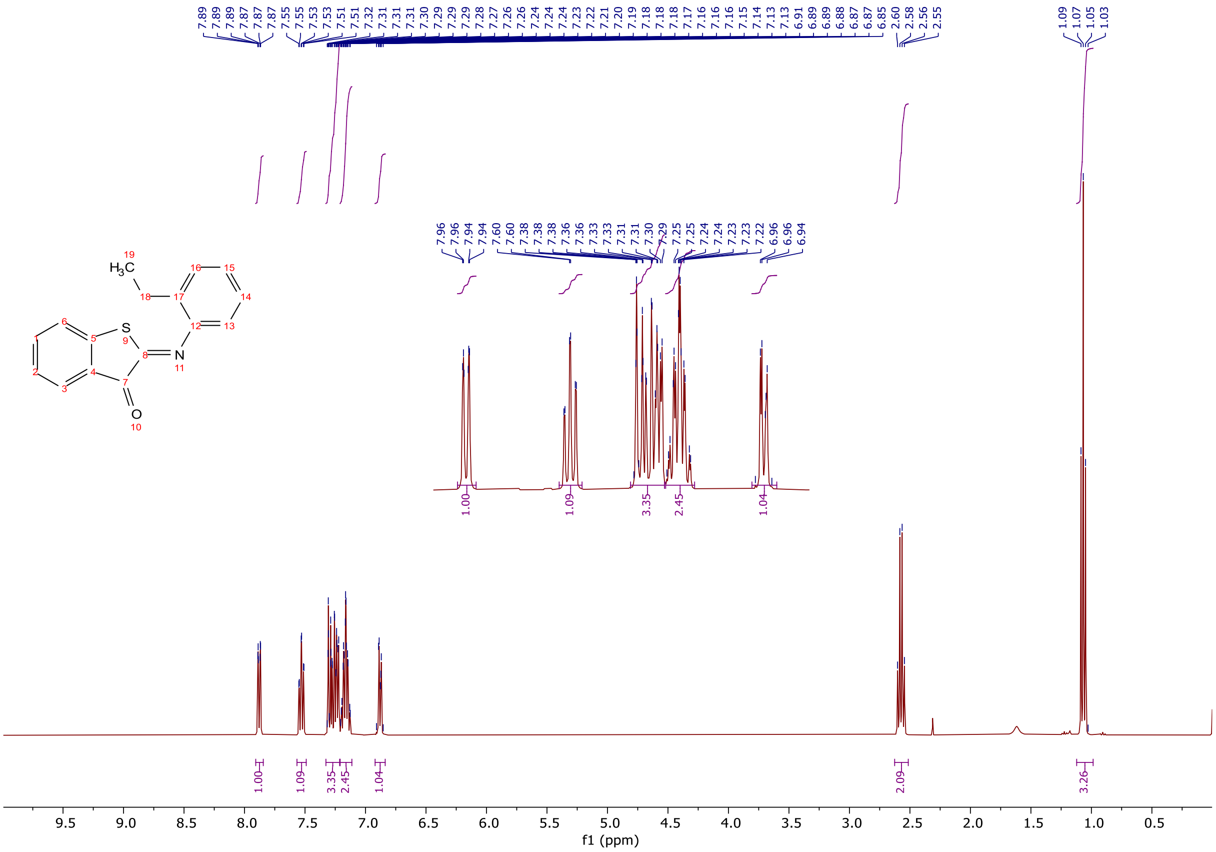

Figure S44 ^{1}H NMR spectrum of compound **1o** in $CDCl_3$.

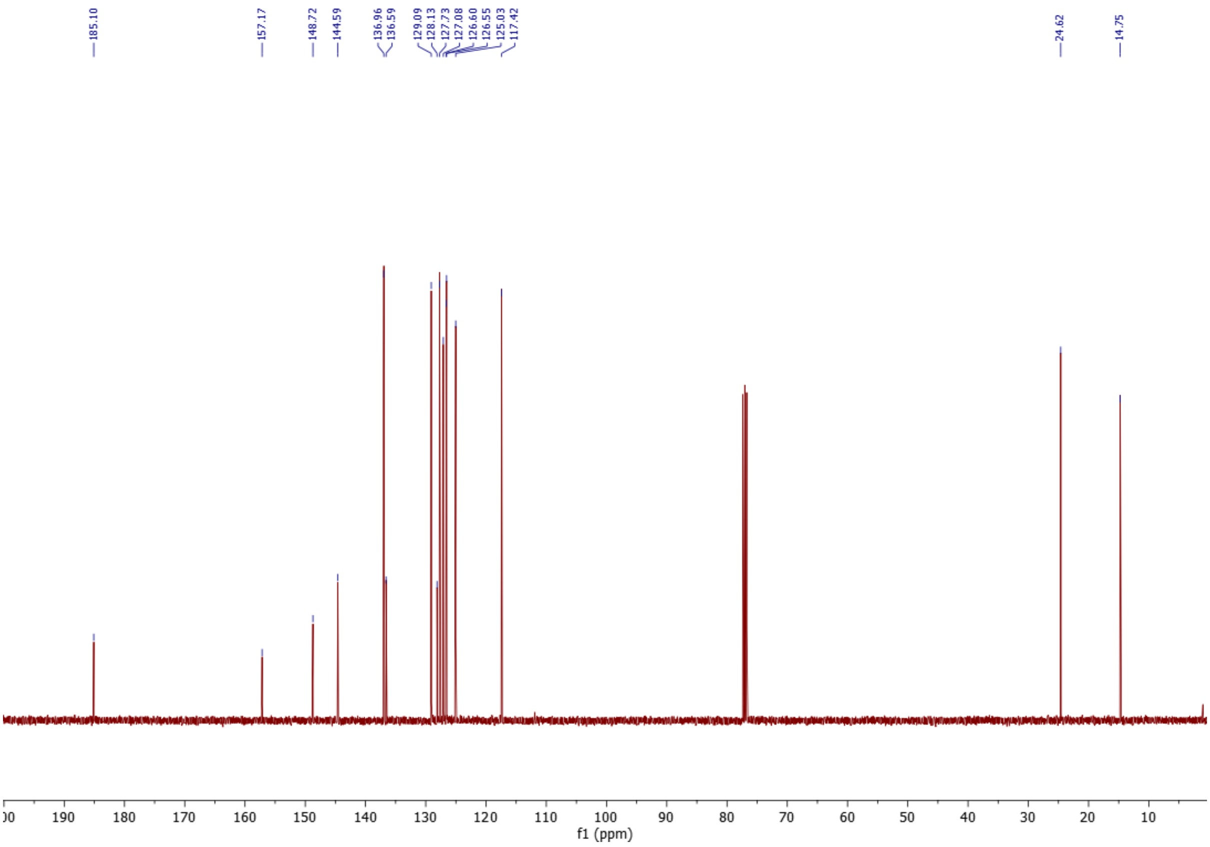

Figure S45 ^{13}C NMR spectrum of compound **1o** in $CDCl_3$.

1p: (*Z*)-2-((2-isopropylphenyl)imino)benzo[b]thiophen-3(2H)-one

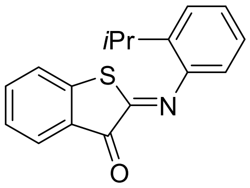

Benzo[b]thiophen-3-yl acetate **8a** (45 mg, 0.23 mmol, 1.0 eq) and 2-isopropylnitrosobenzene **5p** (51 mg, 0.38 mmol, 1.7 eq) were dissolved in ethanol (6 mL). The mixture was cooled in an ice-water bath. KOH (25 g/L in EtOH, 0.3 mL, 0.11 mmol, 0.36 eq) was added dropwise and the mixture was allowed to reach room temperature and stirred vigorously. After 35 min, TLC indicated completion. Then, DCM (30 mL) was added, and the mixture washed with brine (50 mL). The aqueous layer was extracted with DCM (2 x 25 mL) and the combined organic layers were dried over $MgSO_4$ and concentrated *in vacuo*. The crude product was purified by flash chromatography (Silicagel 40 – 63 nm, 0-10% EtOAc in *n*-heptane). The product was obtained as an orange solid (60 mg, 0.21 mmol, 68%). Mp: 89-91 °C. ^1H NMR (400 MHz, $CDCl_3$) δ 7.96 (ddd, *J* = 7.7, 1.4, 0.7 Hz, 1H, Ar**H**, 7.60 (app td, *J* = 7.6, 1.4 Hz, 1H, Ar**H**), 7.40 – 7.30 (m, 3H, Ar**H**), 7.29 – 7.20 (m, 2H, Ar**H**), 6.96 – 6.89 (m, 1H, Ar**H**), 3.22 (hept, *J* = 6.9 Hz, 1H, C**H**), 1.17 (d, *J* = 6.9 Hz, 7H, 2 x C**H**$_3$).^{13}C NMR (101 MHz, $CDCl_3$) δ 185.1, 157.2, 148.2, 144.7, 141.1, 137.0, 128.2, 127.8, 127.2, 126.6, 126.4, 126.1, 125.1, 117.4, 28.4, 23.3. HRMS (ESI+) calc. for. $[M+H]^+$ ($C_{17}H_{16}NOS^+$) 282.0947, found: 282.0945.

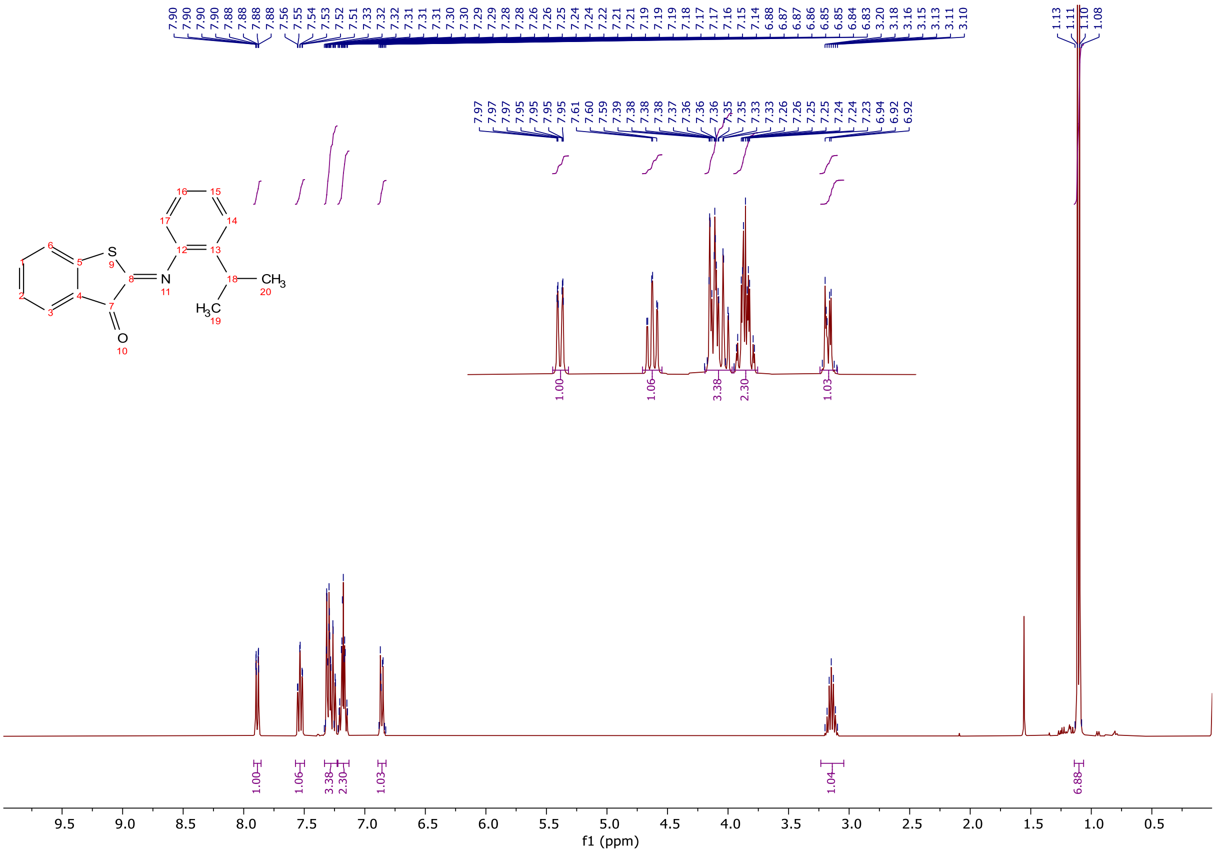

Figure S46 ^1H NMR spectrum of compound **1p** in $CDCl_3$.

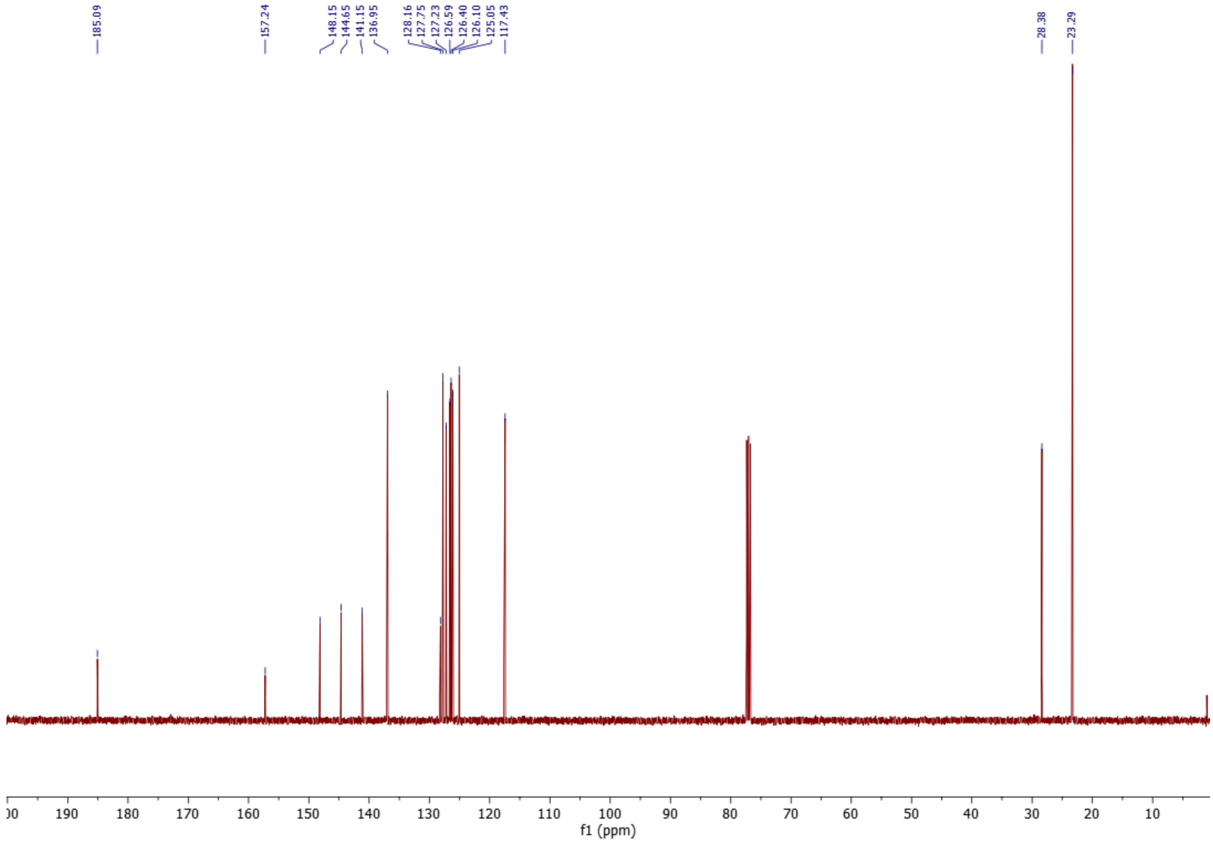

Figure S47 ^{13}C NMR spectrum of compound **1p** in $CDCl_3$.

1q: (*Z*)-2-((2-bromophenyl)imino)benzo[b]thiophen-3(2H)-one

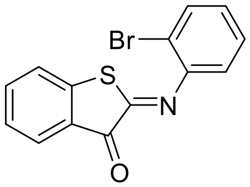

Benzo[b]thiophen-3-yl acetate **8a** (52 mg, 0.27 mmol, 1.0 eq) and 2-bromonitrosobenzene **5q** (65 mg, 0.35 mmol, 1.3 eq) were dissolved in ethanol (5 mL). The mixture was cooled in an ice-water bath. KOH (25 g/L in EtOH, 0.28 mL, 0.13 mmol, 0.36 eq) was added dropwise and the mixture was allowed to reach room temperature and stirred vigorously. After 2 h, TLC indicated completion. Then, DCM (30 mL) was added, and the mixture washed with brine (25 mL). The aqueous layer was extracted with DCM (2 x 25 mL) and the combined organic layers were dried over $MgSO_4$ and concentrated *in vacuo*. The crude product was purified by flash chromatography (Silicagel 40 – 63 nm, 0-10% EtOAc in *n*-heptane). The product was obtained as an orange solid (45 mg, 0.16 mmol, 50%). Mp: 112-114°C. ^{1}H NMR (400 MHz, $CDCl_3$) δ 8.00 – 7.95 (m, 1H, Ar**H**), 7.70 – 7.60 (m, 2H, Ar**H**), 7.41 – 7.35 (m, 3H, Ar**H**), 7.13 (app td, *J* = 7.8, 1.6 Hz, 1H, Ar**H**), 7.03 (dd, *J* = 7.9, 1.6 Hz, 1H, Ar**H**). ^{13}C NMR (101 MHz, $CDCl_3$) δ 143.8, 137.2, 133.5, 128.2, 127.9, 127.9, 127.6, 126.9, 125.0, 119.1. (IR, cm^{-1}): 2930 (C-H, stretch), 1712 (C=O, stretch), 1593 (C-C, stretch), 1284, 1071 (C-N, stretch), 1005 (=C-H, bend). HRMS (ESI+) calc. for. $[M+H]^+$ ($C_{14}H_9BrNOS^+$) 317.9583, found: 317.9580.

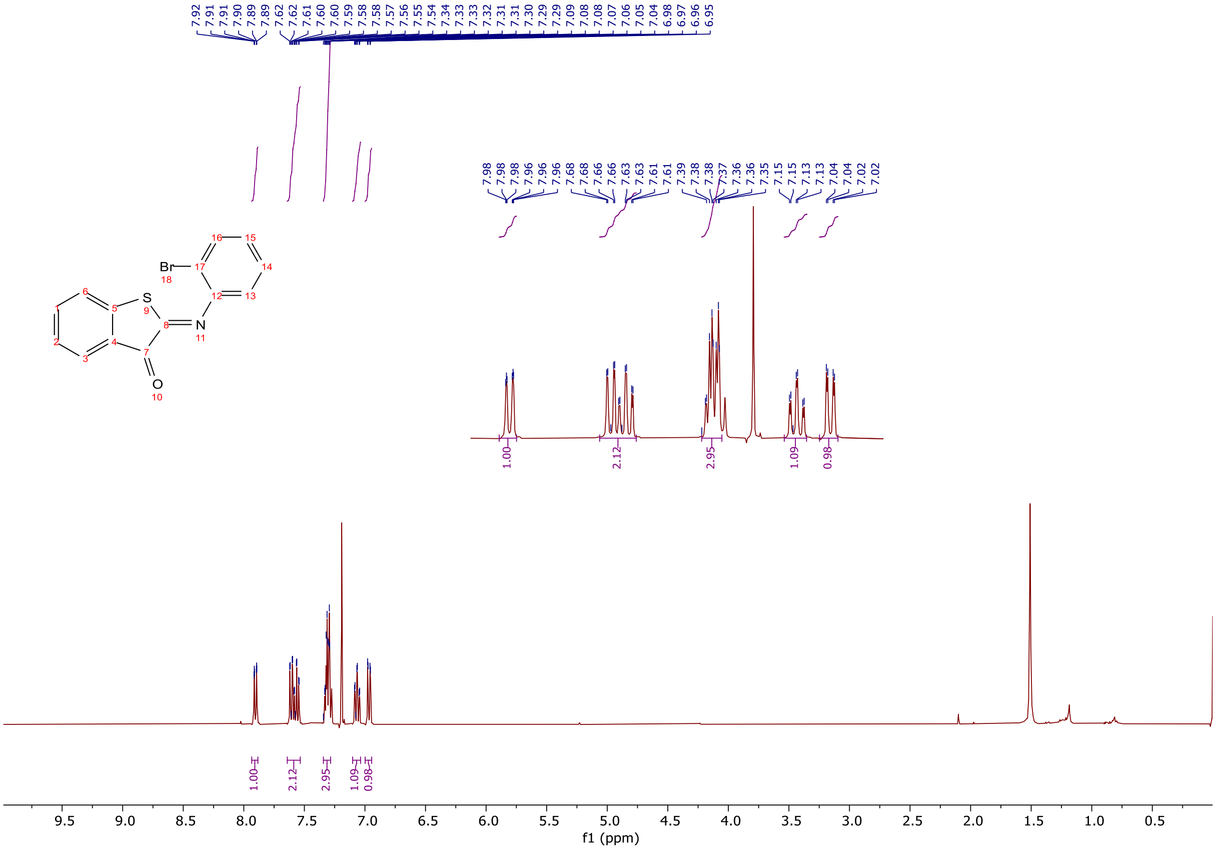

Figure S48 ^{1}H NMR spectrum of compound **1q** in $CDCl_3$.

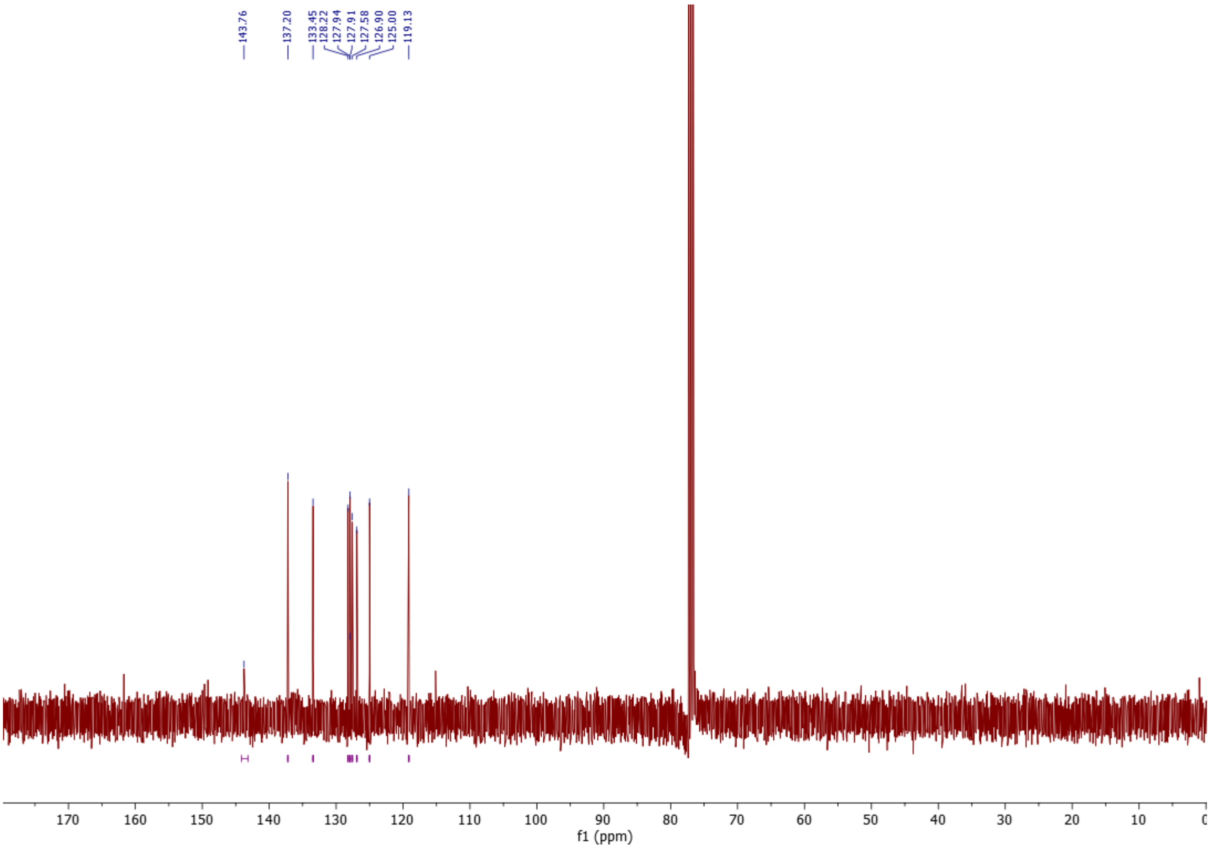

Figure S49 ^{13}C NMR spectrum of compound **1q** in $CDCl_3$.

1r: (*Z*)-2-((2,6-dibromophenyl)imino)benzo[b]thiophen-3(2H)-one

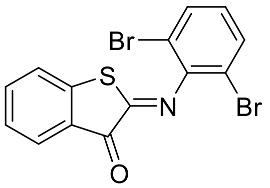

Benzo[b]thiophen-3-yl acetate **8a** (27 mg, 0.14 mmol, 1.0 eq) and 2,6-dibromonitrosobenzene **5r** (47 mg, 0.18 mmol, 1.3 eq) were dissolved in ethanol (5 mL). The mixture was cooled in an ice-water bath. KOH (25 g/L in EtOH, 0.2 mL, 0.067 mmol, 0.38 eq) was added dropwise and the mixture was allowed to reach room temperature and stirred vigorously. After 2 h, TLC indicated completion. Then, DCM (30 mL) was added, and the mixture washed with brine (75 mL). The aqueous layer was extracted with DCM (2 x 25 mL) and the combined organic layers were dried with $MgSO_4$ and concentrated *in vacuo*. The crude product was purified by flash chromatography (Silicagel 40 – 63 nm, 0-10% EtOAc in *n*-heptane). The product was obtained as a viscous orange liquid (51 mg, 0.13 mmol, 72%). ^{1}H NMR (400 MHz, $CDCl_3$) δ 8.00 – 7.90 (m, 1H, Ar**H** (3)), 7.68 – 7.54 (m, 3H, Ar**H** (2, 14, and 16)), 7.36 (m, *J* = 9.8, 7.6, 1.6 Hz, 2H, Ar**H** (1 and 6)), 6.95 (t, *J* = 8.1 Hz, 1H, Ar**H** (15)).^{13}C NMR (101 MHz, $CDCl_3$) δ 184.3, 163.3, 148.2, 143.0, 137.6, 132.4, 128.0, 127.9, 127.3, 127.1, 125.0, 112.4. IR (cm^{-1}): 1710 (C=O, stretch), 1629 (C=C, stretch), 1586 (C=C, stretch), 1451 (C-C, stretch), 1421 (C-C, stretch), 1284, 740 (C=C, bend). HRMS (ESI+) calc. for. $[M+H]^+$ ($C_{14}H_8Br_2NOS^+$) 397.8667, found: 397.8658.

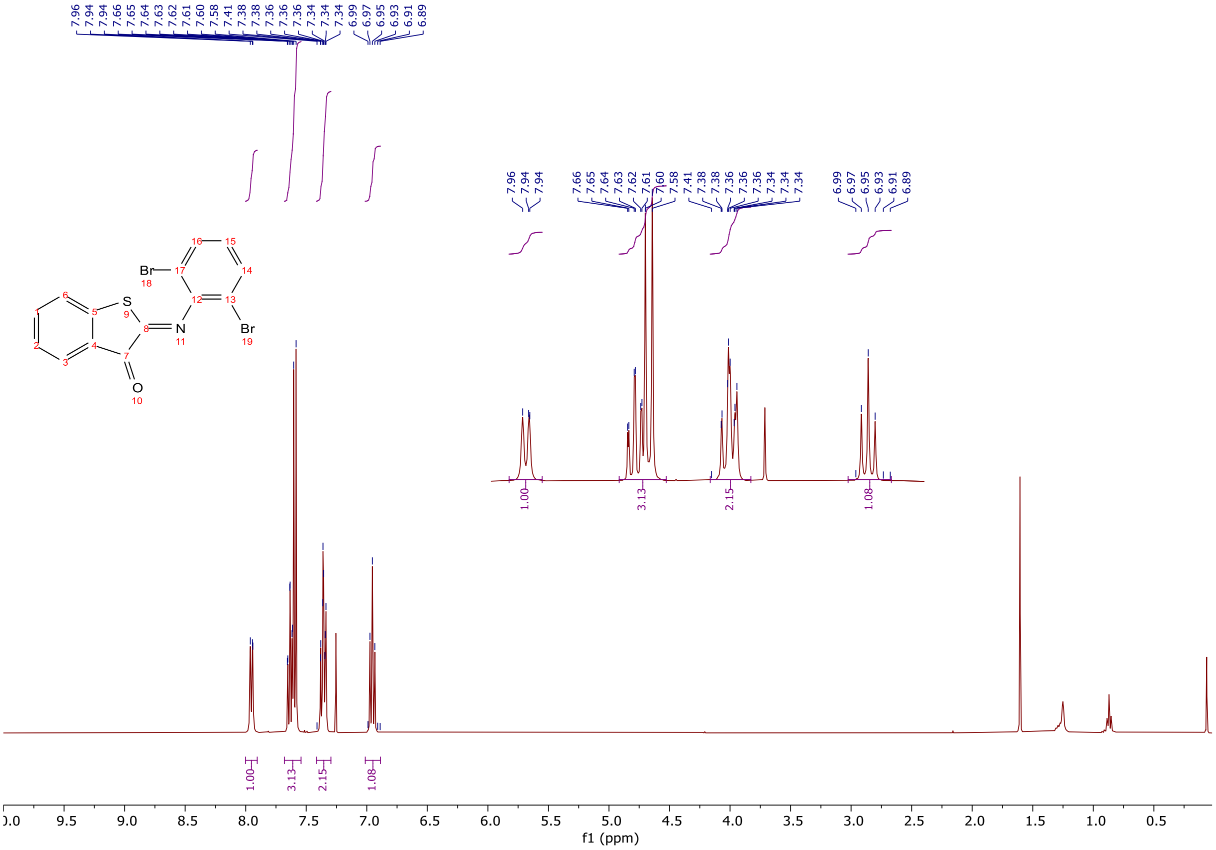

Figure S50 ^{1}H NMR spectrum of compound **1r** in $CDCl_3$.

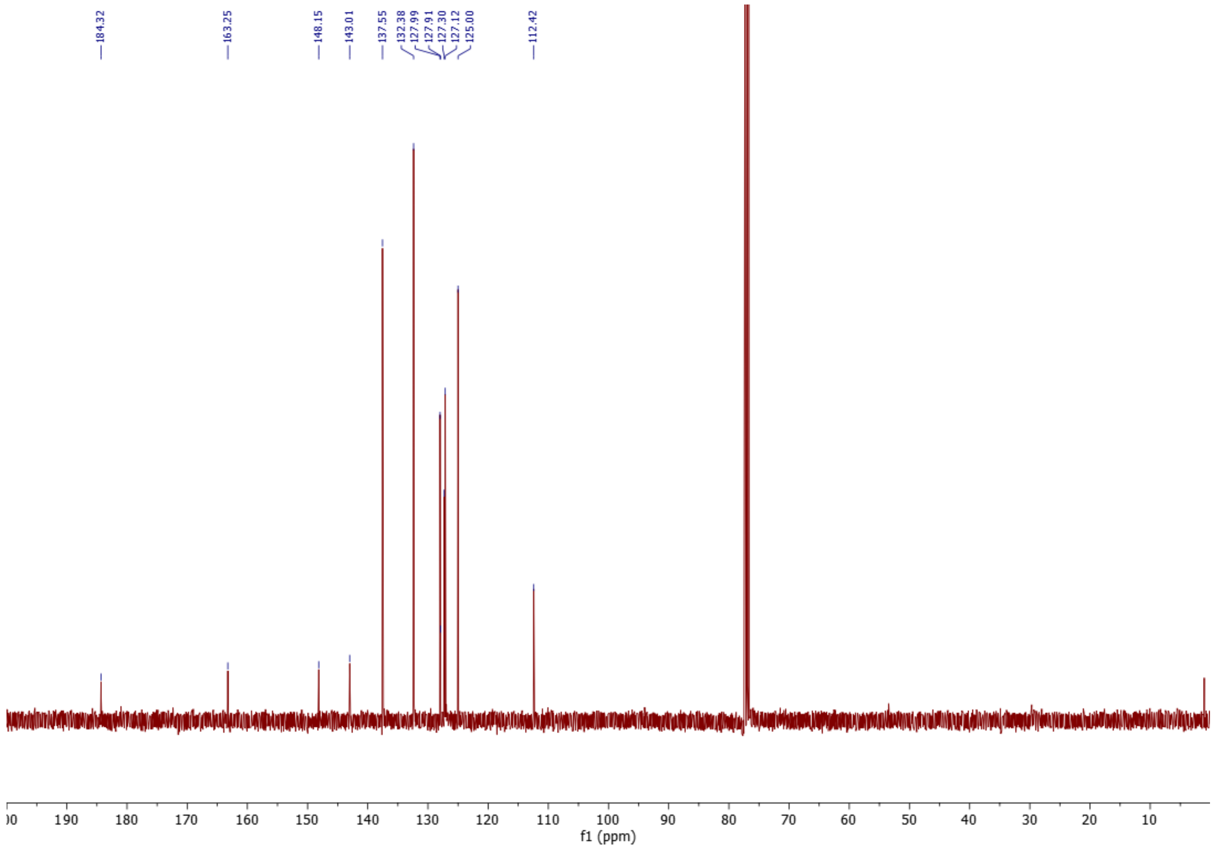

Figure S51 ^{1}H NMR spectrum of compound **1r** in $CDCl_3$.

1s: (*Z*)-2-((2-chlorophenyl)imino)benzo[b]thiophen-3(2H)-one

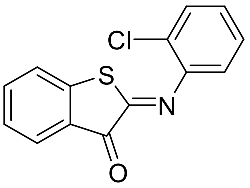

Benzo[b]thiophen-3-yl acetate **8a** (53 mg, 0.28 mmol, 1.0 eq) and 2-chloronitrosobenzene **5s** (50 mg, 0.35 mmol, 1.3 eq) were dissolved in ethanol (5 mL). The mixture was cooled on an ice-water bath. KOH (25 g/L in EtOH, 0.3 mL, 0.13 mmol, 0.36 eq) was added dropwise and the mixture was allowed to reach room temperature and stirred vigorously. After 1 h, TLC indicated completion. Then, DCM (50 mL) was added, and the mixture washed with brine (50 mL). The aqueous layer was extracted with DCM (2 x 25 mL) and the combined organic layers were dried with $MgSO_4$ and concentrated *in vacuo*. The crude product was purified by flash chromatography (Silicagel 40 – 63 nm, 0-10% EtOAc in *n*-heptane). The product was obtained as an orange solid (9.0 mg, 0.033 mmol, 9%). Mp: 117-118 °C. ^{1}H NMR (400 MHz, $CDCl_3$) δ 8.00 – 7.92 (m, 1H, Ar**H**), 7.62 (app td, J = 7.7, 1.4 Hz, 1H, Ar**H**), 7.48 (dd, J = 8.0, 1.4 Hz, 1H, Ar**H**), 7.39 – 7.29 (m, 3H, Ar**H**), 7.20 (app td, J = 7.7, 1.6 Hz, 1H, Ar**H**), 7.05 (dd, J = 7.9, 1.6 Hz, 1H, Ar**H**). ^{13}C NMR (101 MHz, $CDCl_3$) δ 184.7, 159.9, 147.7, 143.7, 137.2, 130.4, 127.9, 127.5, 127.4, 126.9, 125.6, 125.0, 119.4. HRMS (ESI+) calc. for $[M+H]^+$ ($C_{14}H_9ClNOS^+$) 274.0088, found: 274.0088.

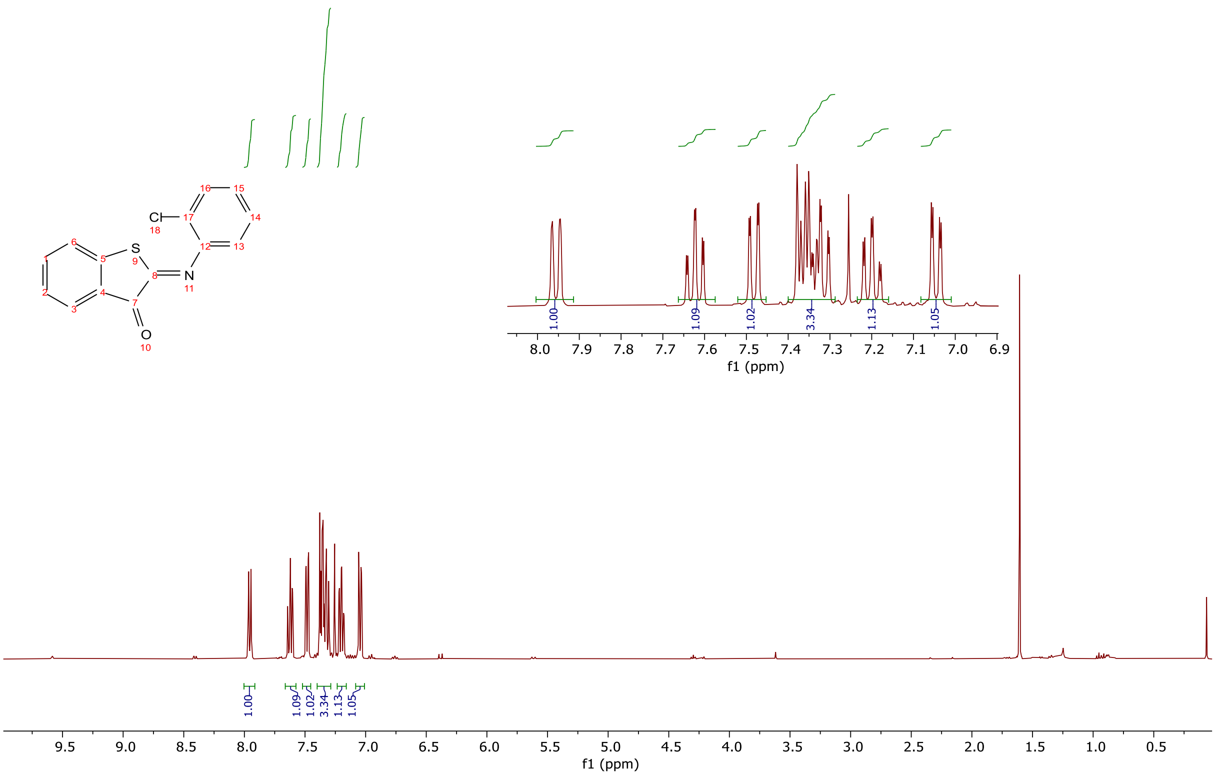

Figure S52 ^{1}H NMR spectrum of compound **1s** in $CDCl_3$.

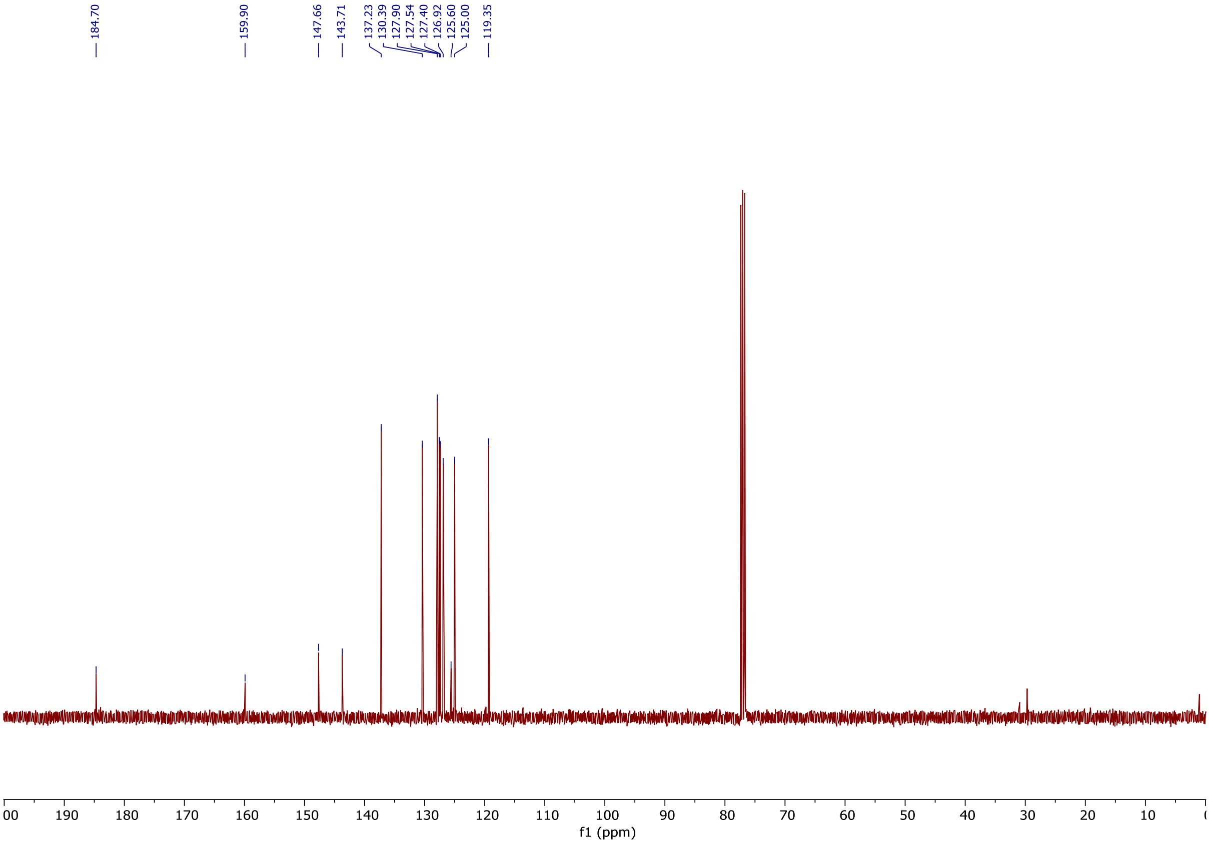

Figure S53 ^{13}C NMR spectrum of compound **1s** in $CDCl_3$.

1t: (*Z*)-2-((2,6-dichlorophenyl)imino)benzo[b]thiophen-3(2H)-one

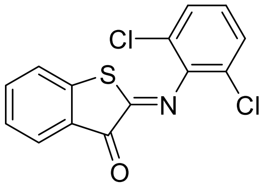

Benzo[b]thiophen-3-yl acetate **8a** (50 mg, 0.26 mmol, 1.0 eq) and 2,6-dichloronitrosobenzene **5t** (60 mg, 0.34 mmol, 1.3 eq) were dissolved in ethanol (5 mL). The mixture was cooled in an ice-water bath. KOH (25 g/L in EtOH, 0.3 mL, 0.12 mmol, 0.36 eq) was added dropwise and the mixture was allowed to reach room temperature and stirred vigorously. After 15 min, TLC indicated completion. Then, DCM (30 mL) was added, and the mixture washed with brine (25 mL). The aqueous layer was extracted with DCM (2 x 25 mL) and the combined organic layers were dried with $MgSO_4$ and concentrated *in vacuo*. The crude product was purified by flash chromatography (Silicagel 40 – 63 nm, n-0-10% EtOAc in *n*-heptane). The product was obtained as an orange solid (63 mg, 0.20 mmol, 60%). Mp: 118-121 °C. ^{1}H NMR (400 MHz, $CDCl_3$) δ 7.95 (dd, *J* = 7.6, 1.4 Hz, 1H, Ar**H** (3)), 7.63 (app td, *J* = 7.6, 1.4 Hz, 1H, Ar**H** (2)), 7.42 – 7.31 (m, 4H, Ar**H** (6, 14, 15, and 16)), 7.10 (t, *J* = 8.1 Hz, 1H, Ar**H** (1)). ^{13}C NMR (101 MHz, $CDCl_3$) δ 184.2, 163.2, 142.9, 137.5, 128.6, 128.0, 127.9, 127.1, 126.5, 125.0, 124.0. HRMS (ESI+) calc. for $[M+H]^+$ ($C_{14}H_8Cl_2NOS^+$) 307.9698, found: 307.9694.

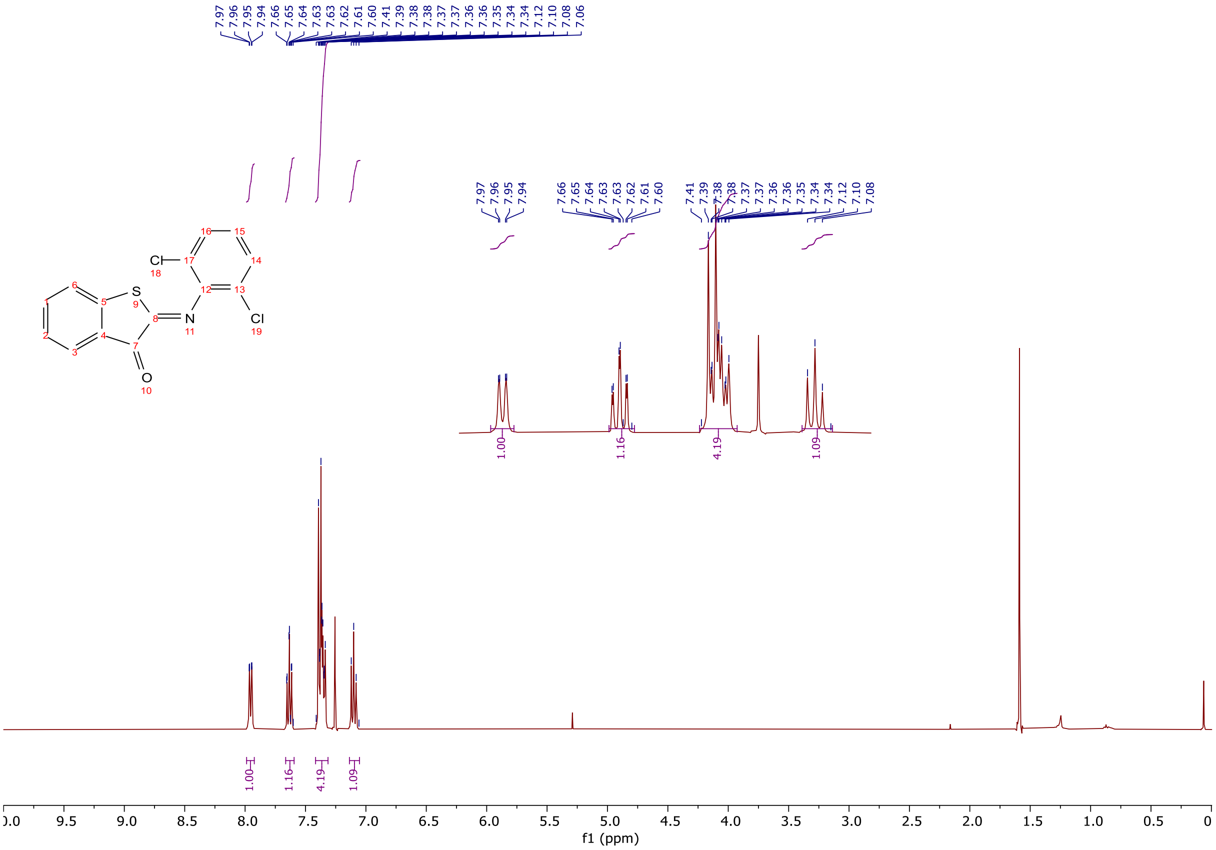

Figure S54 ^{1}H NMR spectrum of compound **1t** in $CDCl_3$.

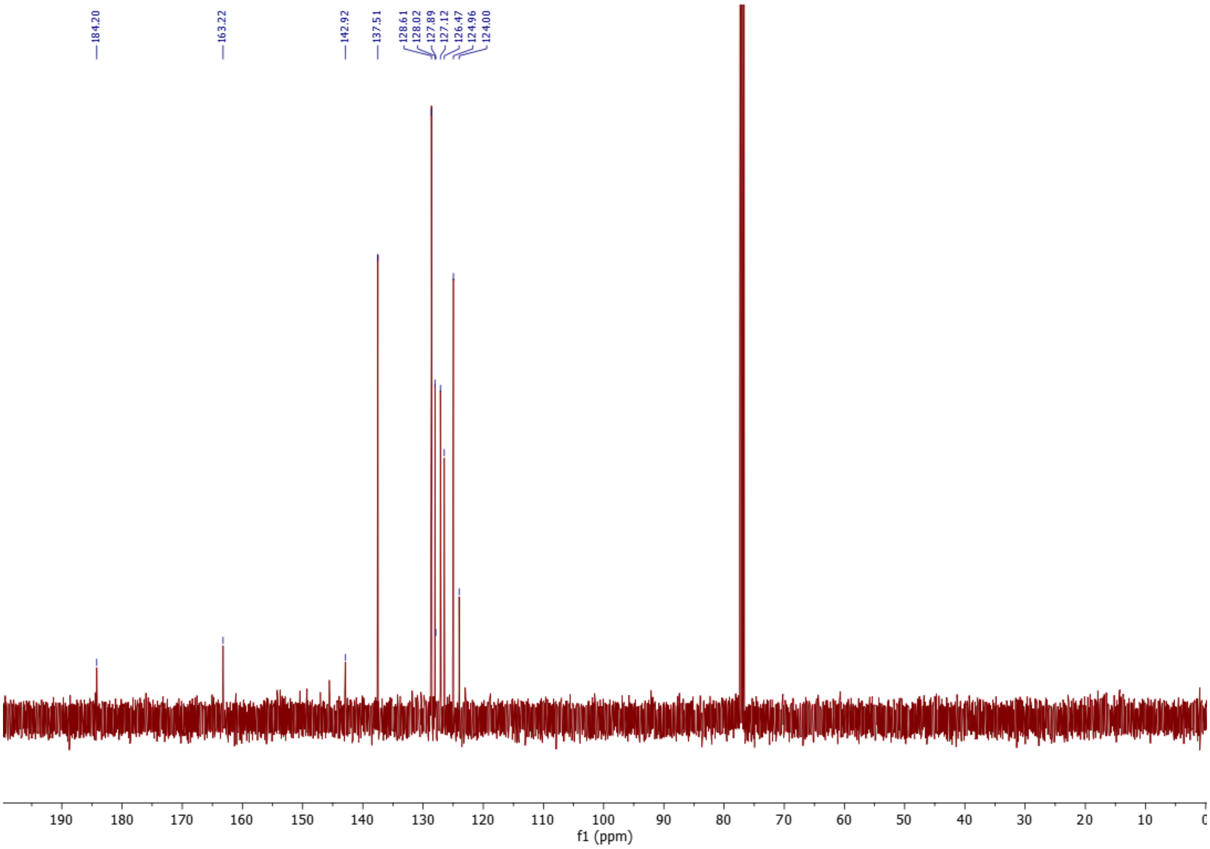

Figure S55 ^{13}C NMR spectrum of compound **1t** in $CDCl_3$.

1u: (*Z*)-2-((2-fluorophenyl)imino)benzo[b]thiophen-3(2H)-one

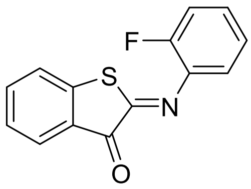

Benzo[b]thiophen-3-yl acetate **8a** (80 mg, 0.42 mmol, 1.0 eq) and 1-fluoro-2-nitrosobenzene **5u** (77 mg, 0.62 mmol, 1.5 eq) were dissolved in ethanol (2 mL). The mixture was cooled on an ice-water bath. KOH (25 g/L in EtOH) was added dropwise (8 drops) and the mixture was allowed to reach room temperature and stirred vigorously. After 2 h, TLC indicated completion. Then, the product was filtered off and washed with water (1 x 20 mL). The product was dissolved in acetone, dried with $MgSO_4$ and concentrated *in vacuo*. The product was purified by flash chromatography (Silicagel 40 – 63 nm, 0-5% EtOAc in pentane). The product was obtained as an orange solid (26 mg, 0.10 mmol, 32%). Mp: 158 - 160 °C. ^1H NMR (400 MHz, $CDCl_3$) δ 7.92 – 7.99 (d, 1H, Ar**H** (3)), 7.63 (app td, *J* = 7.8, 1.4 Hz, 1H, Ar**H** (2)), 7.32 – 7.42 (m, 2H, Ar**H** (1 and 6)), 7.12 – 7.31 (m, 4H, Ar**H** (13, 14, 15, and 16)). ^{13}C NMR (101 MHz, $CDCl_3$) δ 184.8, 159.8, 152.9 (d, *J* = 251.3 Hz), 143.7, 137.8 (d, *J* = 11.8 Hz), 137.2, 128.2 (d, *J* = 7.5 Hz), 127.9, 127.8, 126.9, 124.9, 124.5 (d, *J* = 3.9 Hz), 121.0 (d, *J* = 1.3 Hz), 116.7 (d, *J* = 19.4 Hz). ^{19}F NMR (376 MHz, $CDCl_3$) δ -122.1 (m). HRMS (ESI+) calc. for. $[M+H]^+$ ($C_{14}H_9FNOS^+$) 258.0391 found: 258.0383.

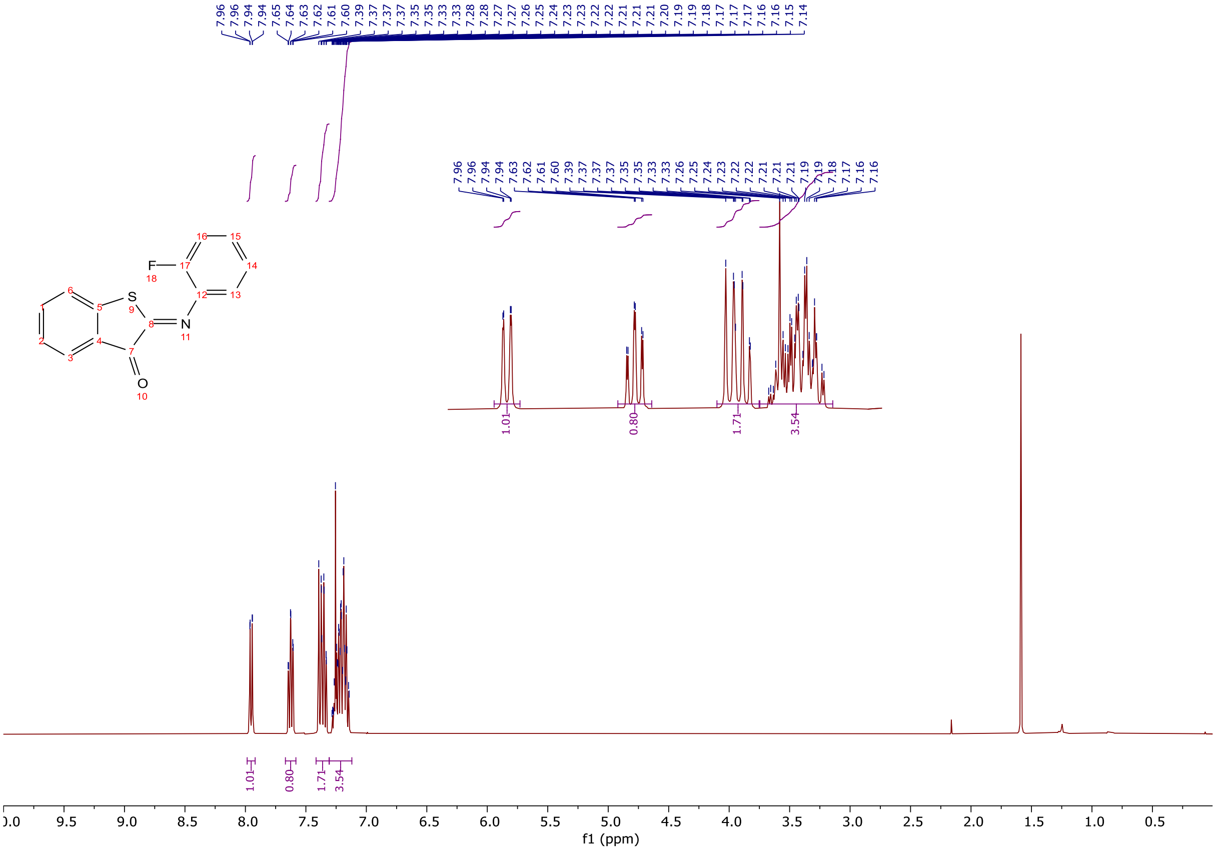

Figure S56 ^{1}H NMR spectrum of compound **1u** in $CDCl_3$.

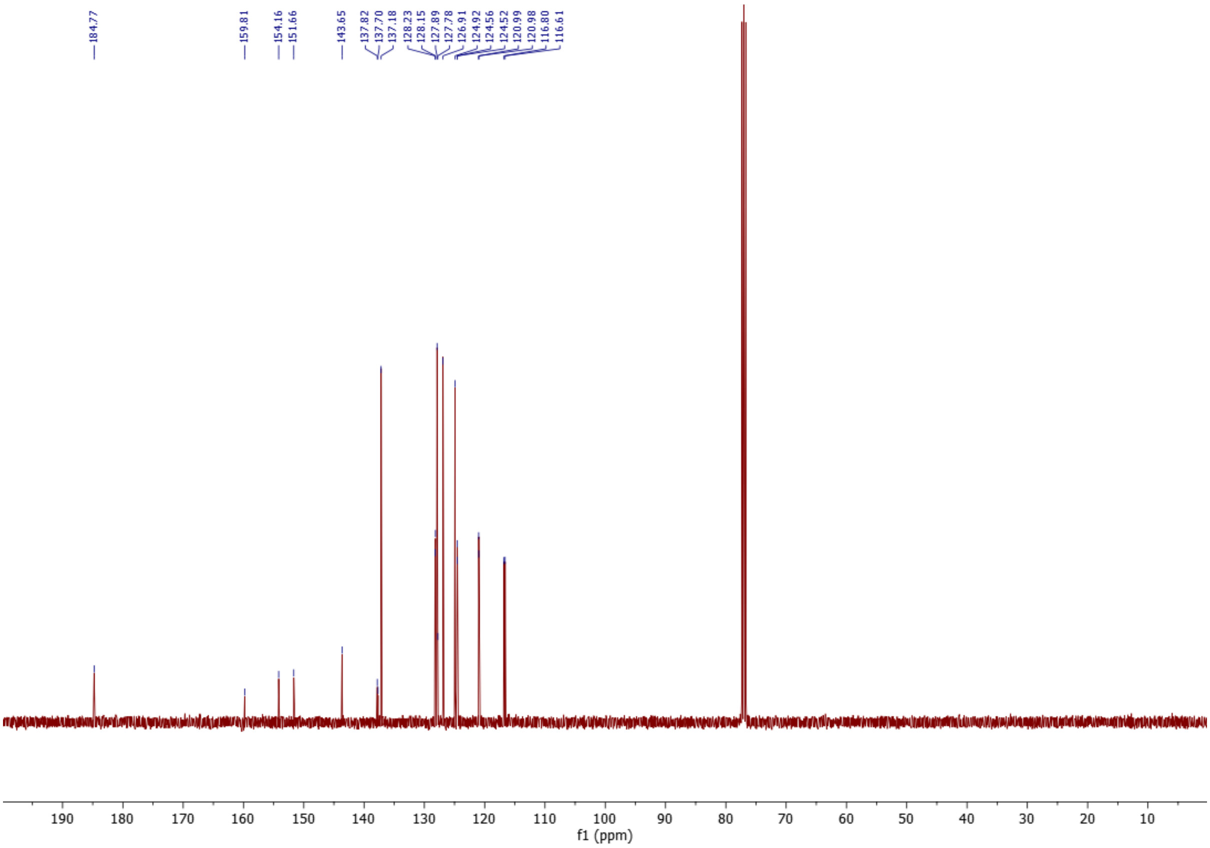

Figure S57 ^{13}C NMR spectrum of compound **1u** in $CDCl_3$.

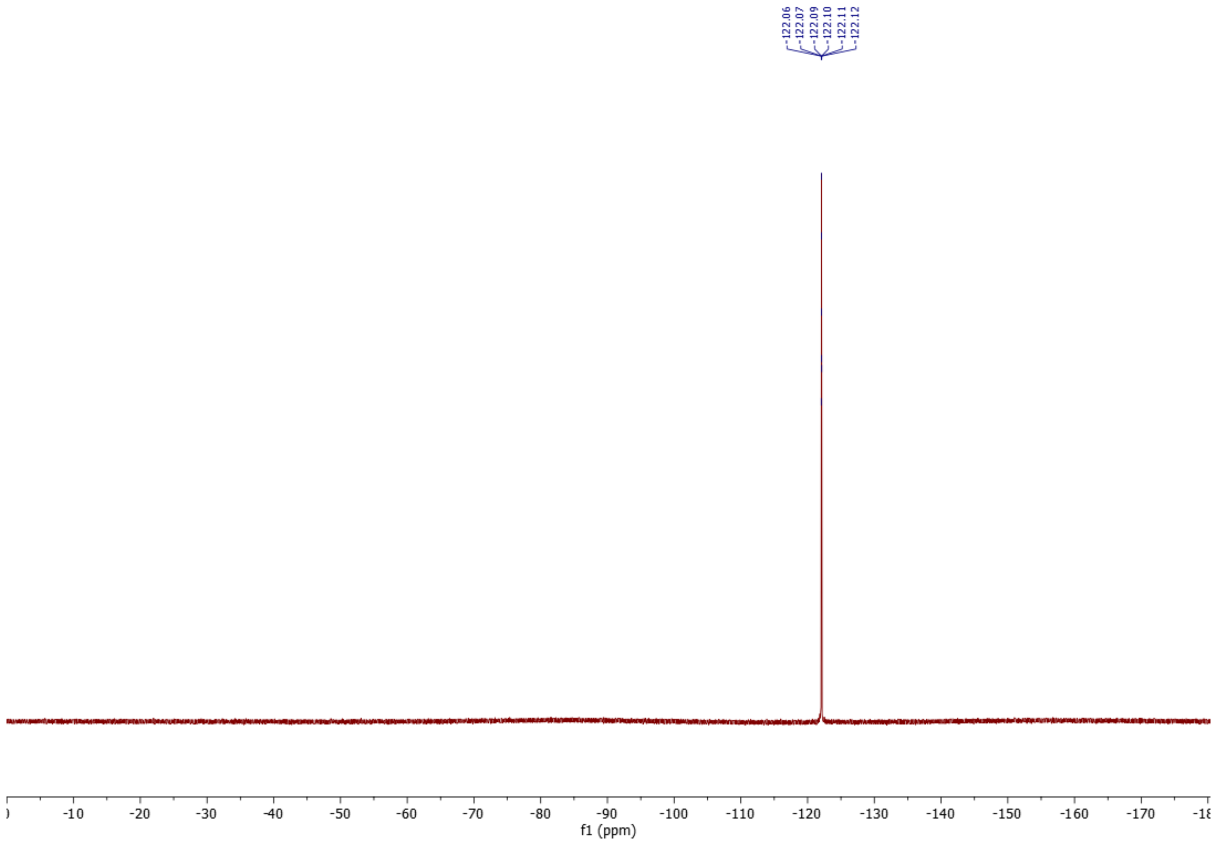

Figure S58 ^{19}F NMR spectrum of compound **1u** in $CDCl_3$.

1v: (Z)-2-((2,6-difluorophenyl)imino)benzo[b]thiophen-3(2H)-one

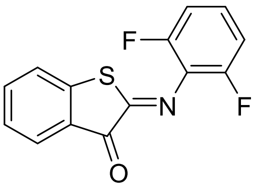

Benzo[*b*]thiophen-3-yl acetate **8a** (63 mg, 0.33 mmol, 1.0 eq) and 2,6-difluoronitrosobenzene **5v** (61 mg, 0.43 mmol, 1.3 eq) were dissolved in ethanol (2 mL). The mixture was cooled on an ice-water bath. KOH (25 g/L in EtOH) was added dropwise (7 drops) and the mixture was allowed to reach room temperature and stirred vigorously. After 15 min, TLC indicated completion. Then, DCM (30 mL) and water (30 mL) were added, and the layers were separated. The aqueous layer was extracted with DCM (2 x 20 mL). The combined organic layers were washed with brine (25 mL), dried with $MgSO_4$, and concentrated *in vacuo*. The product was purified by flash chromatography (Silicagel 40 – 63 nm, 0-5% EtOAc in pentane). The product was obtained as an orange solid (45 mg, 0.16 mmol, 50%). Mp: 149 – 152 °C. ^1H NMR (400 MHz, $CDCl_3$) δ 7.94 (d, *J* = 7.2 Hz, 1H, Ar**H** (3)), 7.64 (t, *J* = 7.6 Hz, 1H, Ar**H** (2)), 7.41 – 7.33 (m, 2H, Ar**H** (1 and 6)), 7.23 – 7.14 (m, 1H, Ar**H** (15)), 7.01 (t, *J* = 8.1 Hz, 2H, Ar**H** (14 and 16)). ^{13}C NMR (101 MHz, $CDCl_3$) δ 184.1, 163.7, 153.6 (d, *J* = 5.1 Hz), 151.1 (d, *J* = 5.1 Hz), 142.8, 137.4, 128.0, 127.8, 127.1, 126.8 (t, *J* = 9.4 Hz), 124.9, 112.1 (dd, *J* = 5.0 Hz, 18.0 Hz). ^{19}F NMR (376 MHz, $CDCl_3$) δ -120.5 (m). HRMS (ESI+) calc. for. $[M+H]^+$ ($C_{14}H_7F_2NOS^+$) 276.0224 found: 276.0290.

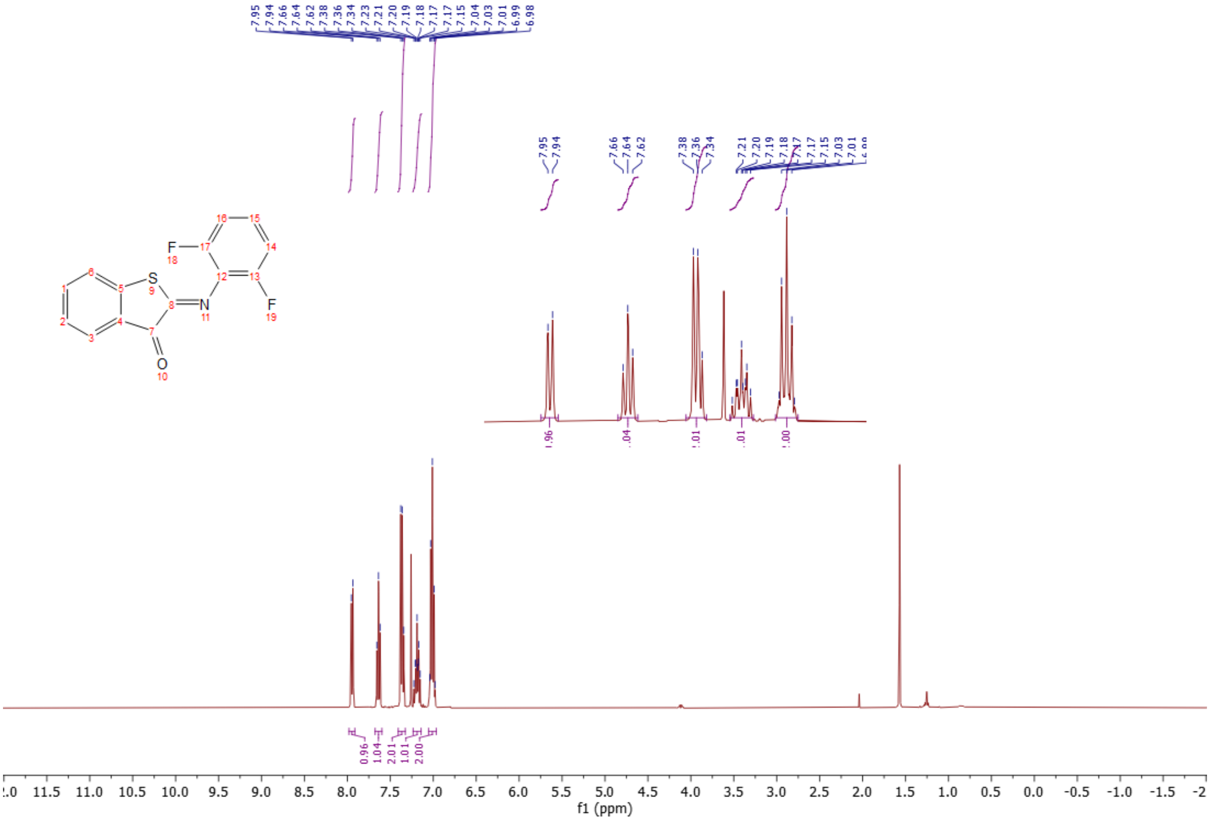

Figure S59 ^{1}H NMR spectrum of compound **1v** in $CDCl_3$.

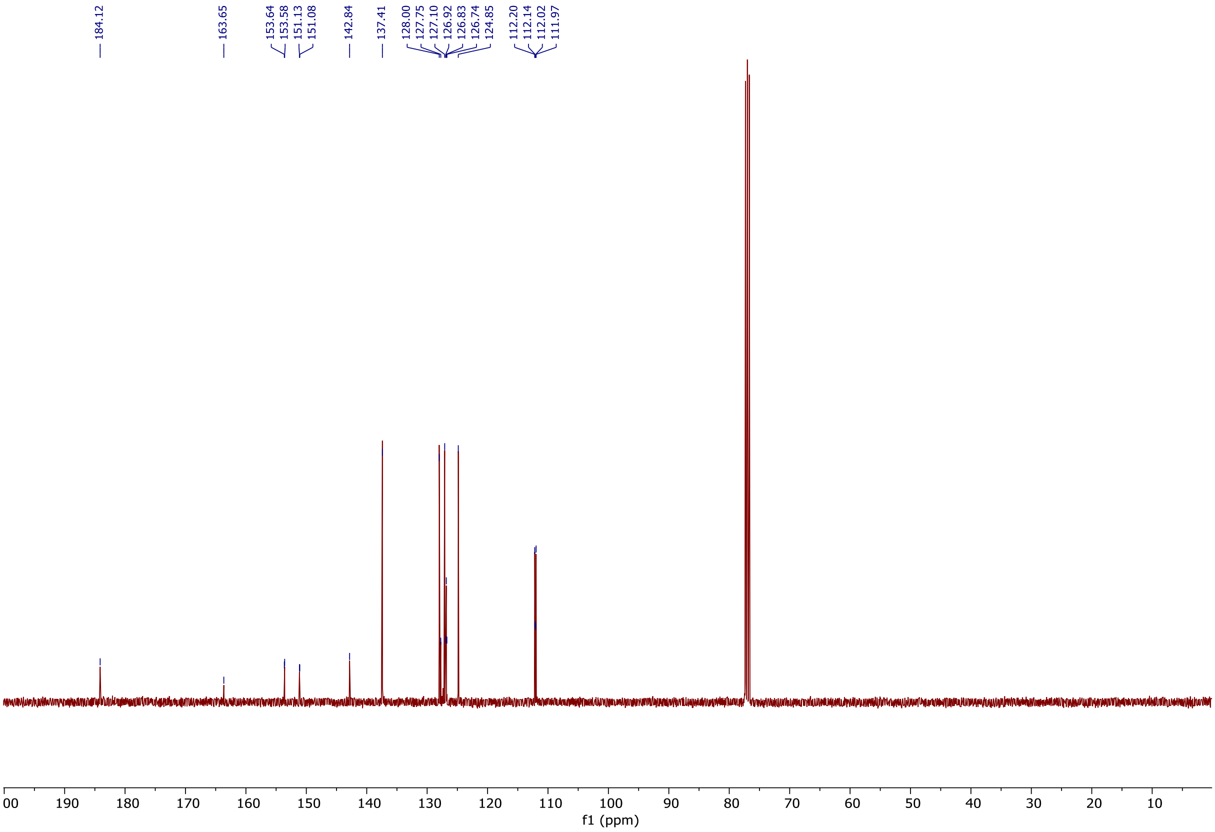

Figure S60 ^{13}C NMR spectrum of compound **1v** in $CDCl_3$.

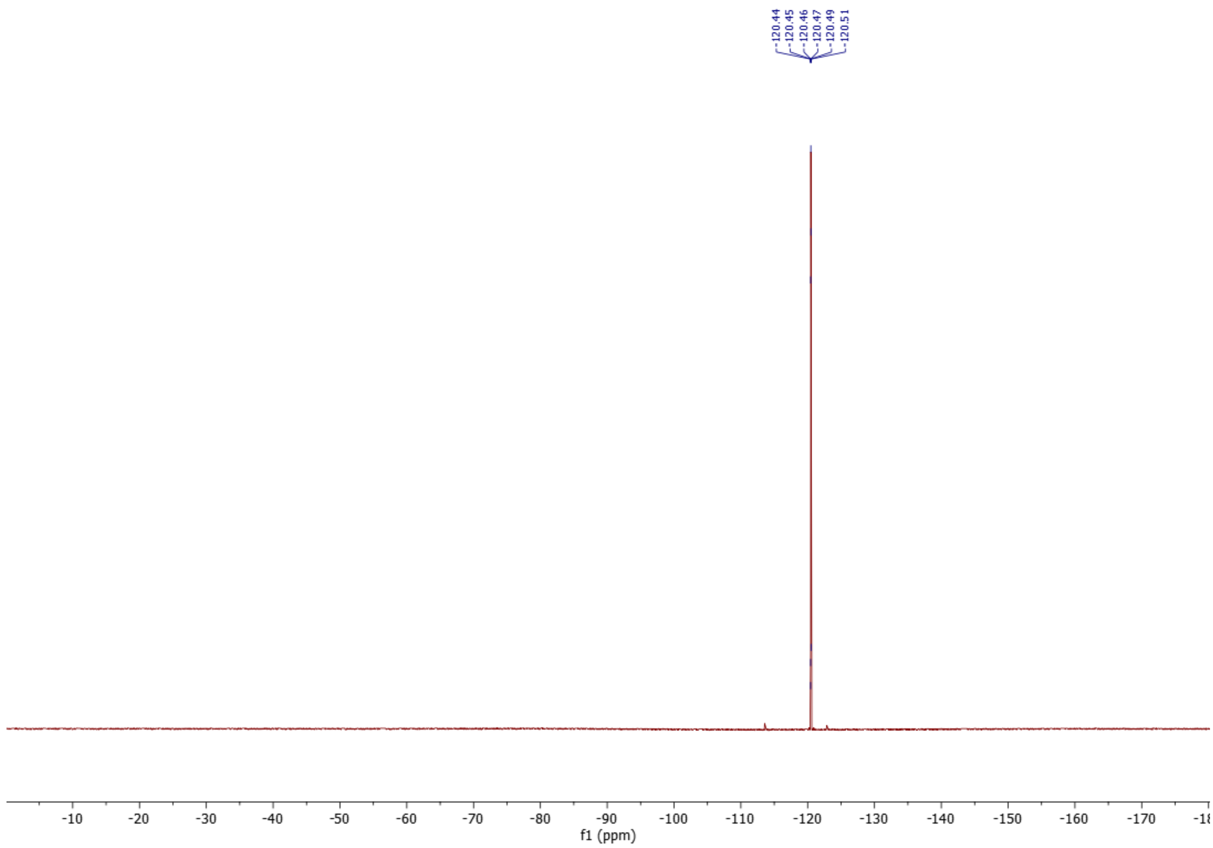

Figure S61 ^{19}F NMR spectrum of compound **1v** in $CDCl_3$.

1w: (*Z*)-2-((2-(trifluoromethyl)phenyl)imino)benzo[b]thiophen-3(2H)-one

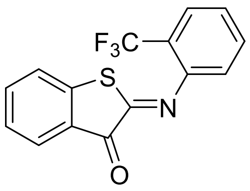

Benzo[b]thiophen-3-yl acetate **8a** (30 mg, 0.16 mmol, 1.0 eq) and 2-trifluoromethylnitrosobenzene **5w** (36 mg, 0.21 mmol, 1.3 eq) were dissolved in ethanol (4 mL). The mixture was cooled in an ice-water bath. KOH (25 g/L in EtOH, 0.2 mL, 0.075 mmol, 0.36 eq) was added dropwise and the mixture was allowed to reach room temperature and stirred vigorously. After 24 h, TLC indicated completion. Then, DCM (30 mL) was added, and the mixture washed with brine (50 mL). The aqueous layer was extracted with DCM (2 x 25 mL) and the combined organic layers were dried with $MgSO_4$ and concentrated *in vacuo*. The crude product was purified by flash chromatography (Silicagel 40 – 63 nm, 0-10% EtOAc in *n*-heptane). The product was obtained as an orange solid (32.0 mg, 0.10 mmol, 51%). Mp: 111-115 °C. ^{1}H NMR (400 MHz, $CDCl_3$) δ 7.96 (dd, *J* = 8.1, 1.4 Hz, 1H, Ar**H** (3)), 7.73 (dd, *J* = 7.8, 1.4 Hz, 1H, Ar**H** (16)), 7.67 – 7.55 (m, 2H, Ar**H** (1 and 6), 7.40 – 7.30 (m, 3H, Ar**H** (2, 13, 15)), 7.03 (d, *J* = 7.9 Hz, 1H Ar**H** (14)). ^{13}C NMR (101 MHz, $CDCl_3$) δ 184.3, 160.1, 148.8, 143.5, 137.3, 132.9, 128.0, 127.8, 127.0, 126.8 (q, *J* = 5.1 Hz), 126.0, 125.0, 124.7, 121.9, 118.6. ^{19}F NMR (376 MHz, $CDCl_3$) δ -61.2. HRMS (ESI+) calc. for. $[M+H]^+$ ($C_{15}H_9F_3NOS^+$) 308.0352 , found: 308.0345.

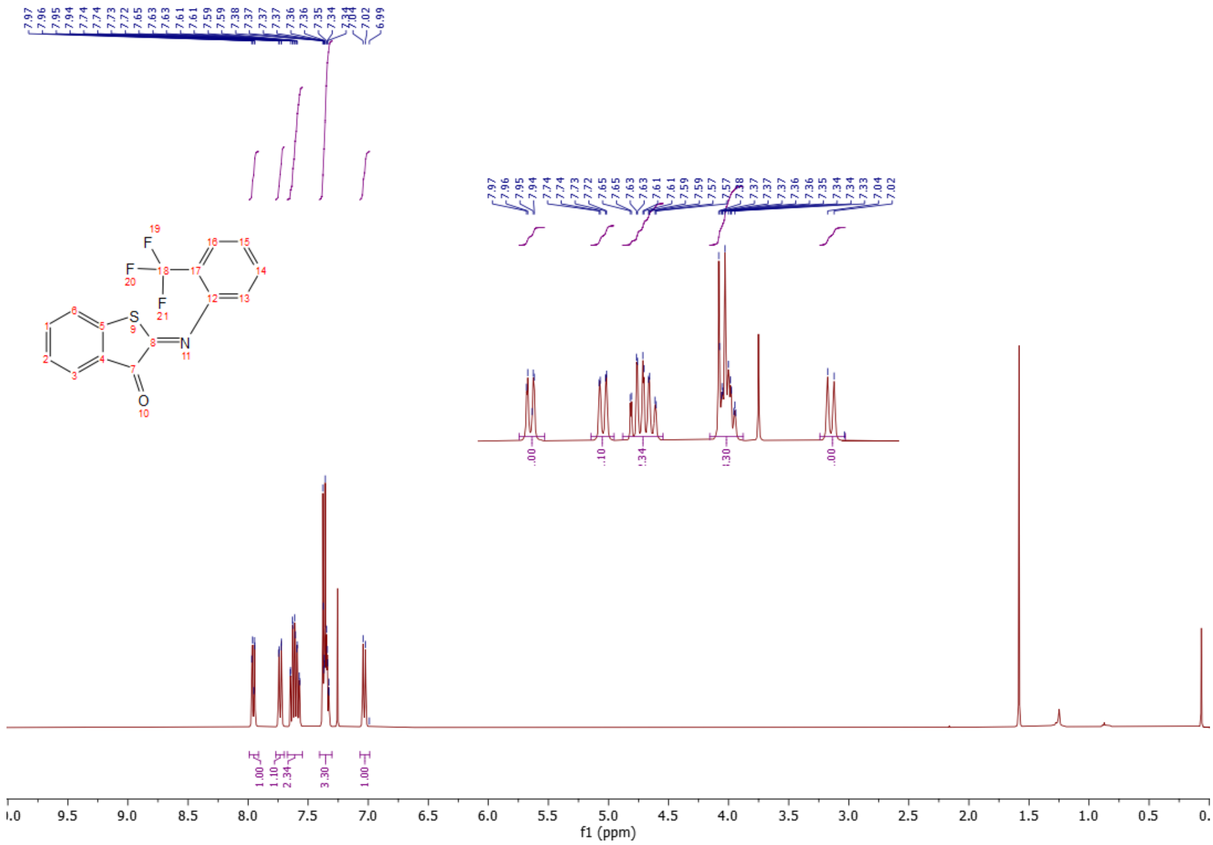

Figure S62 ^{1}H NMR spectrum of compound **1w** in $CDCl_3$.

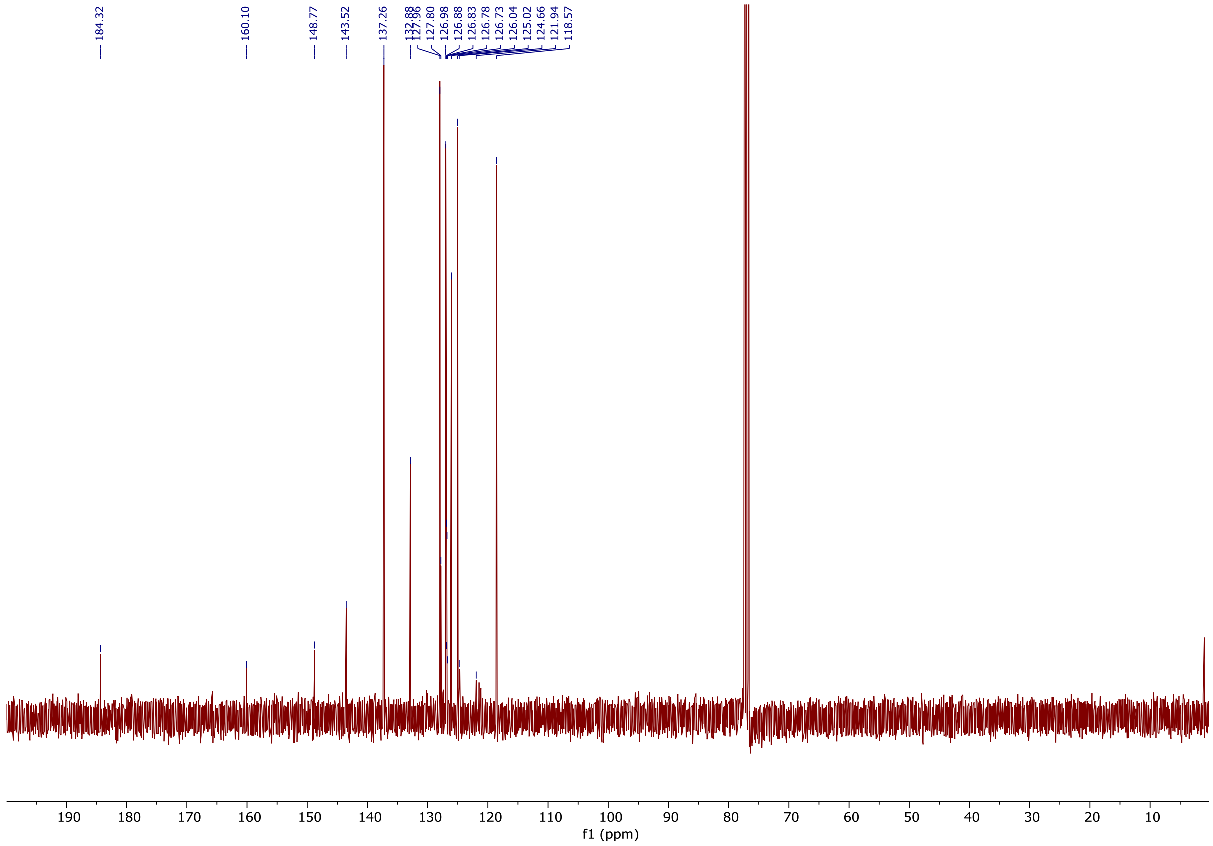

Figure S63 ^{13}C NMR spectrum of compound **1w** in $CDCl_3$.

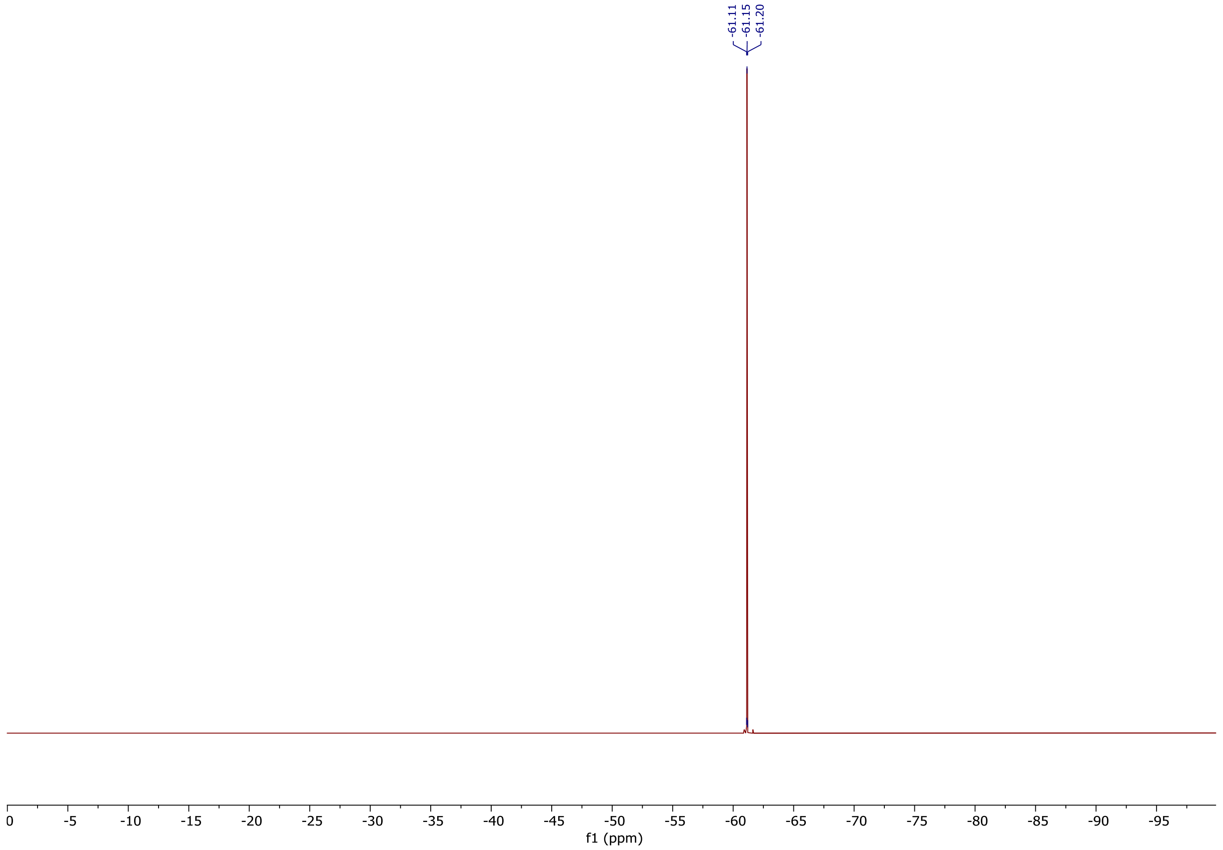

Figure S64 ^{19}F NMR spectrum of compound **1w** in $CDCl_3$.

1x: (*Z*)-2-((2,4,6-trifluorophenyl)imino)benzo[b]thiophen-3(2H)-one

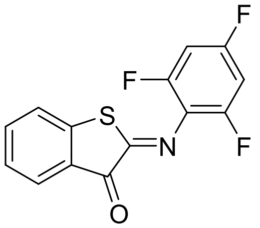

Benzo[b]thiophen-3-yl acetate **3** (45 mg, 0.23 mmol) and crude 2,4,6-trifluoronitrosobenzene **5x** were dissolved in ethanol (4.0 mL). The mixture was cooled on an ice-water bath. KOH (25 g/L in EtOH, 0.3 mL, 0.12 mmol) was added dropwise and the mixture was allowed to reach room temperature and stirred vigorously. After 5 h, TLC indicated completion. Then, DCM (50 mL) was added, and the mixture washed with brine (2 x 50 mL). The aqueous layer was extracted with DCM (2 x 25 mL) and the combined organic layers were dried over $MgSO_4$ and concentrated *in vacuo*. The crude product was purified by flash chromatography (Silicagel 40 – 63 nm, 0-10% EtOAc in *n*-heptane). The product was obtained as an orange solid (50 mg, 0.17 mmol, 61%). Mp: 117-119 °C. ^{1}H NMR (400 MHz, $CDCl_3$) δ 7.95 (dt, *J* = 7.3, 1.2 Hz, 1H, Ar**H** (3)), 7.68 – 7.61 (m, 1H, Ar**H** (2)), 7.41 – 7.34 (m, 2H, Ar**H** (1 and 6)), 6.85 – 6.76 (m, 2H, Ar**H** (14 and 16)). ^{13}C NMR (101 MHz, $CDCl_3$) δ 184.1, 161.5, 153.8, 142.6, 137.5, 128.1, 127.7, 127.2, 124.9, 101.3 (d, J = 2.4 Hz), 101.0 (d, J = 2.2 Hz), 100.8. ^{19}F NMR (376 MHz, $CDCl_3$) δ -110.5 (tt, *J* = 8.4, 4.2 Hz), -116.8 (m). HRMS (ESI+) calc. for. $[M+H]^+$ ($C_{14}H_7F_3NOS^+$) 294.0195, found: 294.0197.

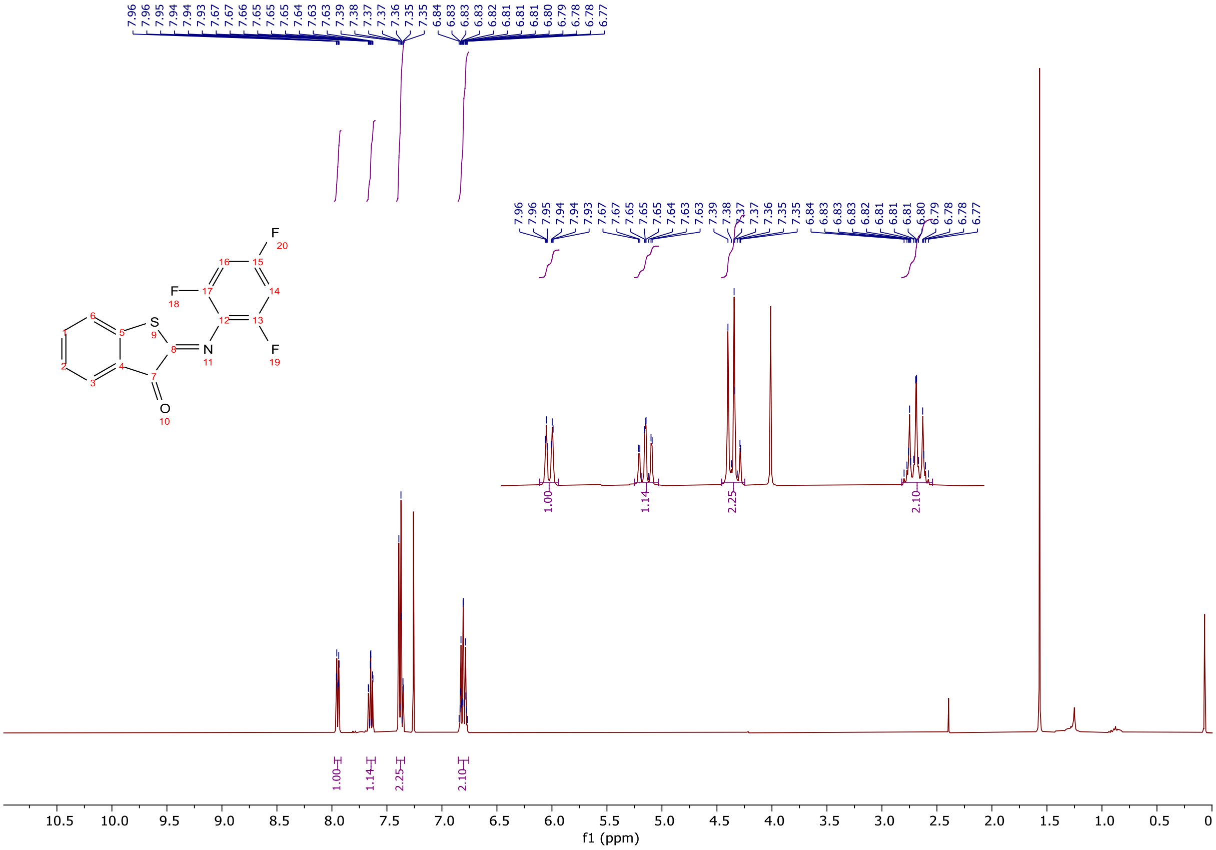

Figure S65 ^{1}H NMR spectrum of compound **1x** in $CDCl_3$.

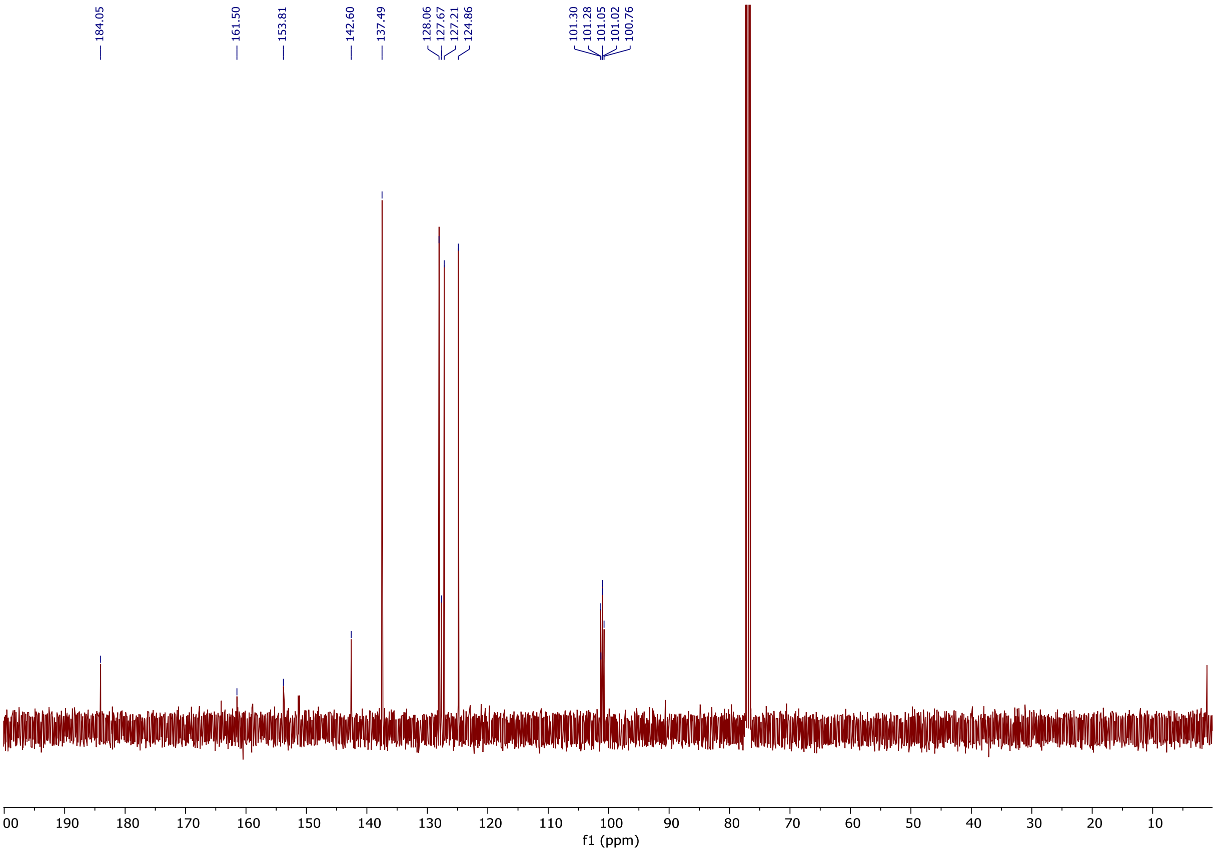

Figure S66 ^{13}C NMR spectrum of compound **1x** in $CDCl_3$.

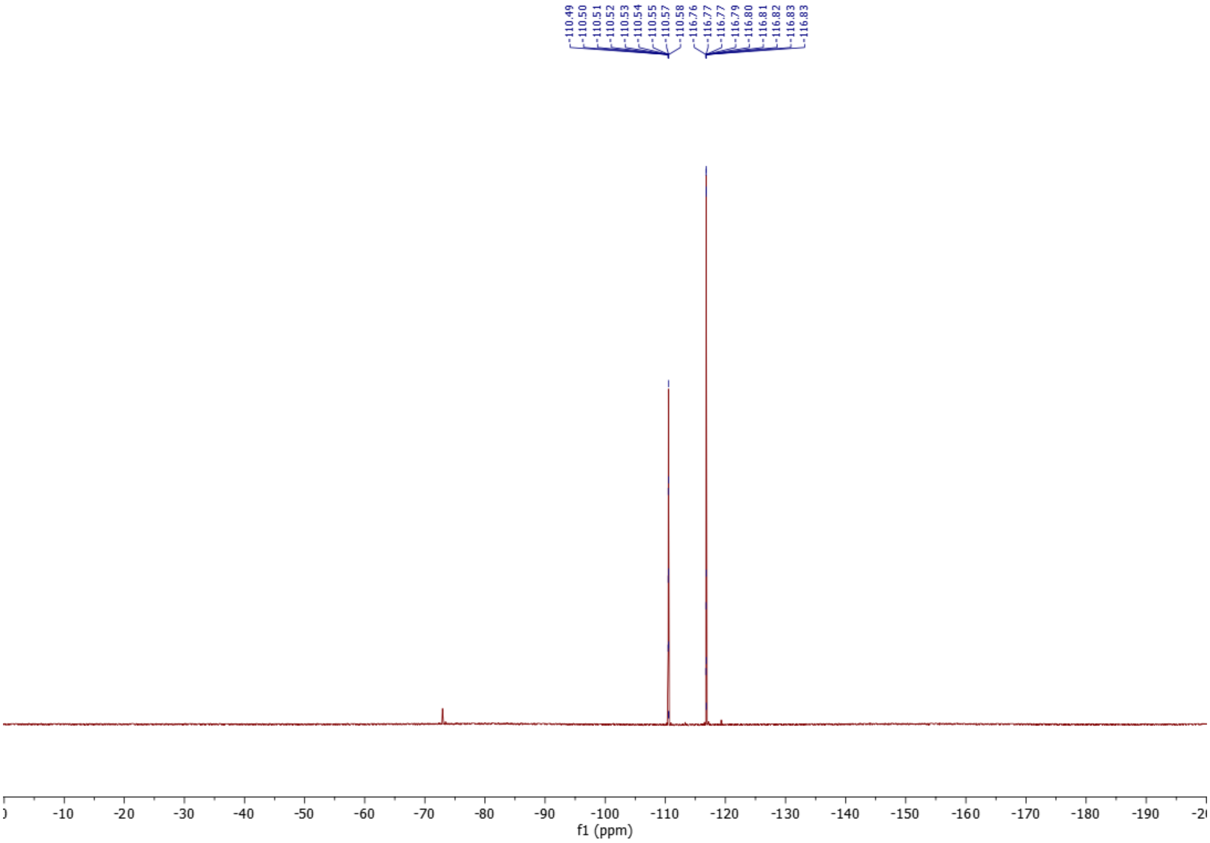

Figure S67 ^{19}F NMR spectrum of compound **1x** in $CDCl_3$.

1y: (*Z*)-2-((2,6-difluoro-4-methoxyphenyl)imino)benzo[b]thiophen-3(2H)-one

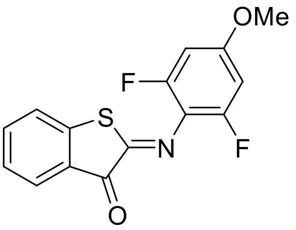

Benzo[b]thiophen-3-yl acetate **8a** (43 mg, 0.22 mmol, 1.0 eq) and 2,6-difluoro-4-methoxynitrosobenzene **5y** (47 mg, 0.27 mmol, 1.2 eq) were dissolved in ethanol (4 mL). The mixture was cooled in an ice-water bath. KOH (25 g/L in EtOH, 0.2 mL, 0.10 mmol, 0.37 eq) was added dropwise and the mixture was allowed to reach room temperature and stirred vigorously. After 3 h, TLC indicated completion. Then, DCM (50 mL) was added, and the mixture washed with brine (2 x 50 mL). The aqueous layer was extracted with DCM (2 x 25 mL) and the combined organic layers were dried over $MgSO_4$ and concentrated *in vacuo*. The crude product was purified by flash chromatography (Silicagel 40 – 63 nm, 0-10% EtOAc in *n*-heptane). The product was obtained as an orange solid (41 mg, 0.13 mmol, 50%). Mp: 134-136 °C. ^{1}H NMR (400 MHz, $CDCl_3$) δ 7.97 – 7.87 (m, 1H, Ar**H** (3)), 7.62 (app td, *J* = 7.6, 1.4 Hz, 1H, Ar**H** (2)), 7.40 – 7.30 (m, 2H, Ar**H** (1 and 6)), 6.63 – 6.50 (m, 2H, Ar**H** (14 and 16)), 3.82 (s, 3H, C**H**$_3$).^{13}C NMR (101 MHz, $CDCl_3$) δ 184.4, 162.2, 159.0 (t, *J* = 13.0 Hz), 154.6 (d, *J* = 8.0 Hz), 152.1 (d, *J* = 8.0 Hz), 143.1, 137.2, 127.9 (d, *J* = 4.8 Hz), 126.9, 124.8, 120.3, 98.6 (m), 56.0.^{19}F NMR (376 MHz, $CDCl_3$) δ -117.6 (m). IR (cm^{-1}): 1713 (C=O, stretch), 1630 (C=C, stretch), 1591 (C=C, stretch), 1493 (C-C, stretch), 1450 (C-C, stretch), 1283 (C-N, stretch), 1147 (C-F, stretch), 1048 (C-F, stretch), 742 (C-H, bend). HRMS (ESI+) calc. for. $[M+H]^+$ ($C_{15}H_{10}F_2NO_2S^+$) 306.0395, found: 306.0395.

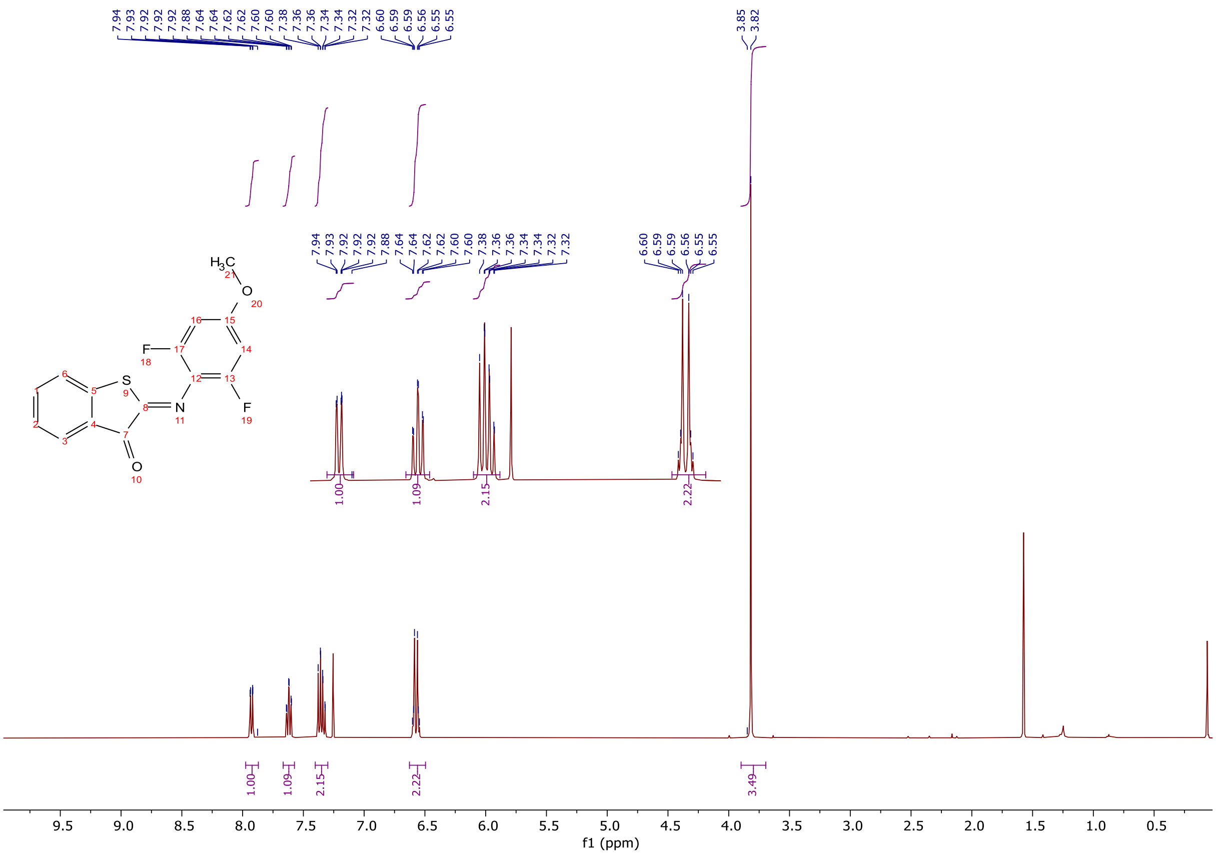

Figure S68 ^{1}H NMR spectrum of compound **1y** in $CDCl_3$.

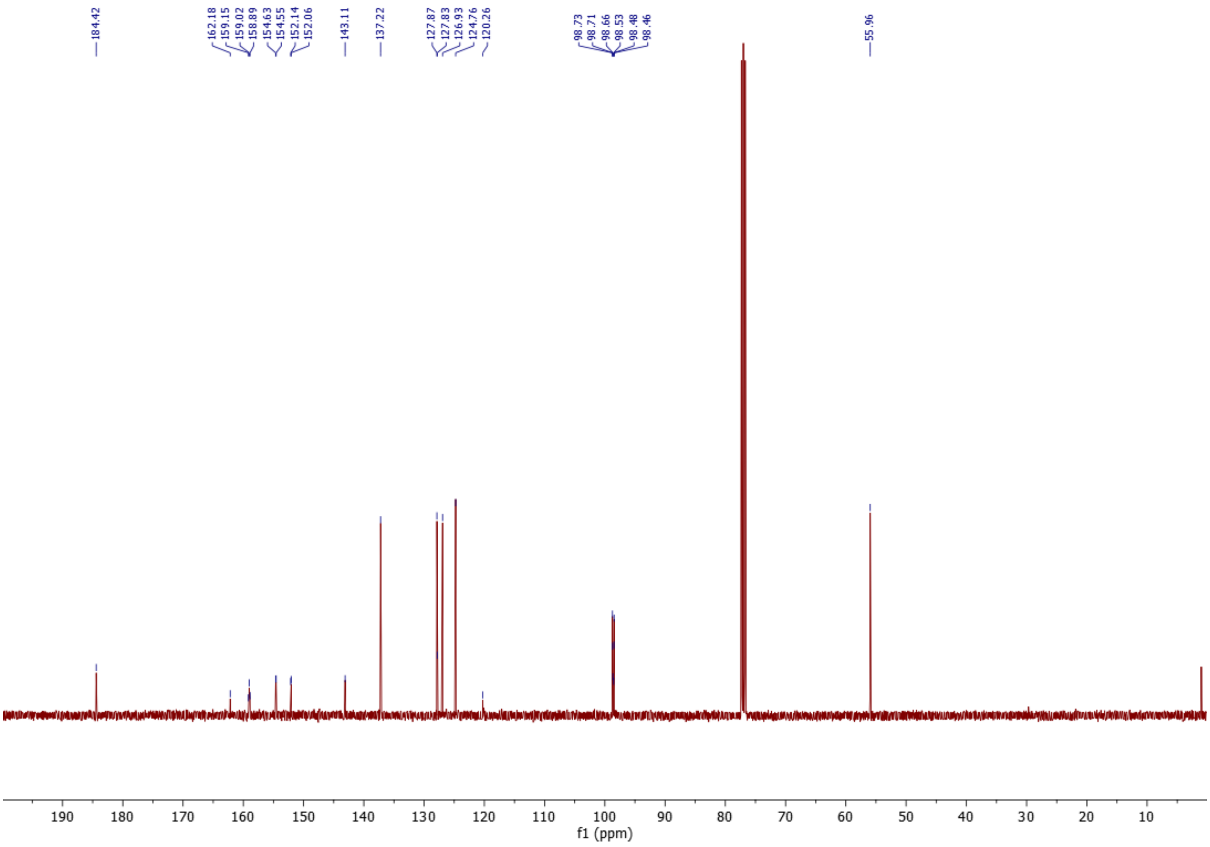

Figure S69 ^{13}C NMR spectrum of compound **1y** in $CDCl_3$.

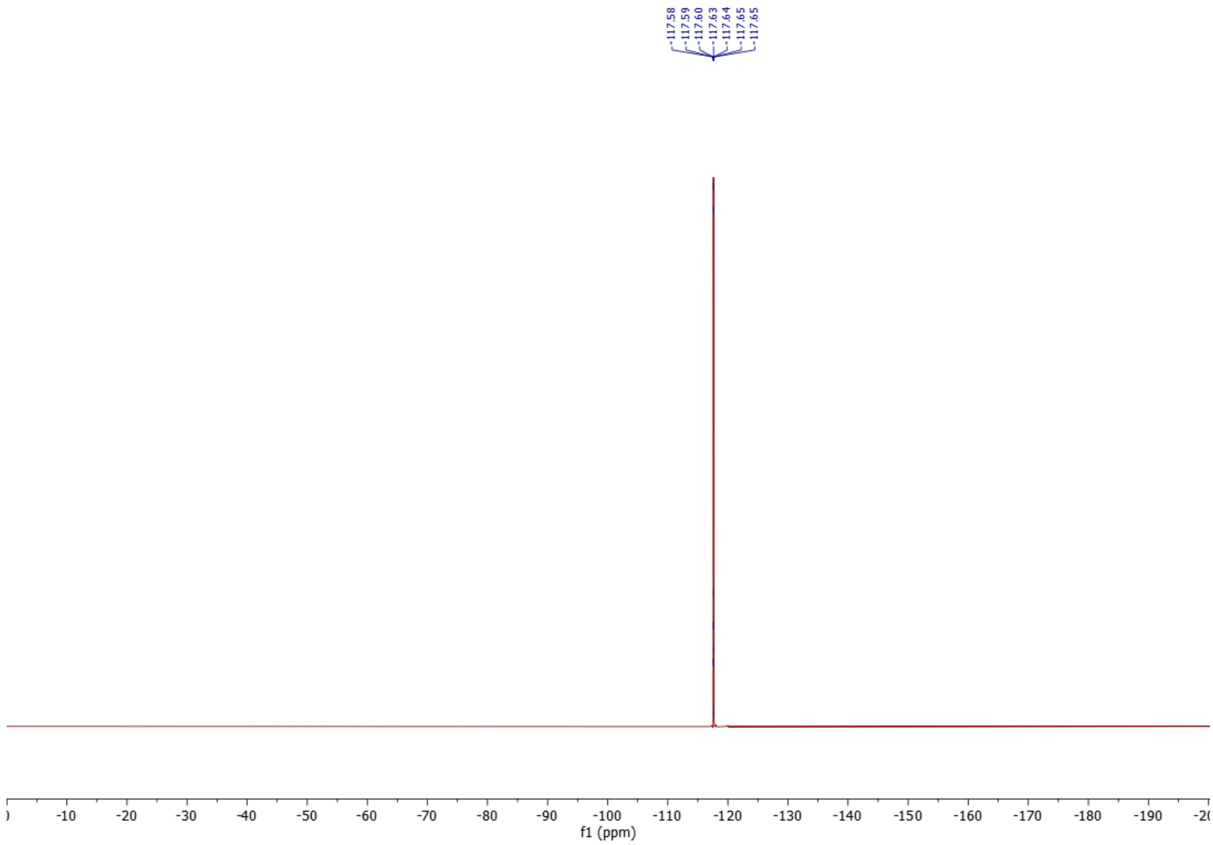

Figure S70 ^{19}F NMR spectrum of compound **1y** in $CDCl_3$.

1z: (*Z*)-2-((perfluorophenyl)imino)benzo[b]thiophen-3(2H)-one

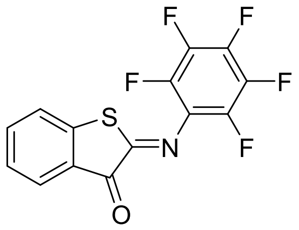

Benzo[b]thiophen-3(2H)-one* **4z** (0.34 g, 2.2 mmol) was dissolved in benzene (17 mL). Crude 2,3,4,5,6-pentafluoronitrosobenzene **5z** and piperidine (0.2 mL, 0.076 mmol) were added. The reaction mixture was stirred under nitrogen atmosphere overnight. After completion (monitored by TLC & LC-MS), DCM (50 mL) and water (50 mL) were added, and the mixture extracted with DCM (3 x 20 mL). The combined organic layers were washed with aq. 1 N HCl (2 x 50 mL), sat. aq. $NaHCO_3$ (2 x 50 mL) and brine (50 mL), dried over $MgSO_4$ and concentrated in vacuo. The crude product was purified by flash chromatography (Silicagel 40 – 63 nm, 0-10% EtOAc in *n*-heptane). The product was obtained as an orange solid (0.44 g, 1.3 mmol, 40%). Mp: 117-120 °C. ^{1}H NMR (400 MHz, $CDCl_3$) δ 7.95 (dd, *J* = 7.9, 1.4 Hz, 1H, Ar**H** (3)), 7.67 (app td, *J* = 7.7, 1.4 Hz, 1H, Ar**H** (2)), 7.44 – 7.33 (m, 2H, Ar**H** (1 and 6)). ^{13}C NMR (101 MHz, $CDCl_3$) δ 183.5, 166.3, 143.0, 141.8, 137.8, 137.4, 128.3, 128.0, 127.6, 127.4, 125.0, 124.8. ^{19}F NMR (376 MHz, $CDCl_3$) δ -148.6 (m), -158.0 (t, *J* = 21.4 Hz), -161.3 (m). IR (cm^{-1}): 1716 (C=O, stretch), 1629 (C=C, stretch), 1591 (C=C, stretch), 1503 (C-C, stretch), 1451 (C-C, stretch), 1281 (C-N, stretch), 1038 (C-F, stretch), 986 (C=C, bend), 743 (C-H, stretch). HRMS (ESI+) calc. for. $[M+H]^+$ ($C_{14}H_5F_5NOS^+$) Exact Mass: 330.00, found: 330.00.

* Benzo[b]thiophen-3(2H)-one was synthesized according to a literature procedure[29]

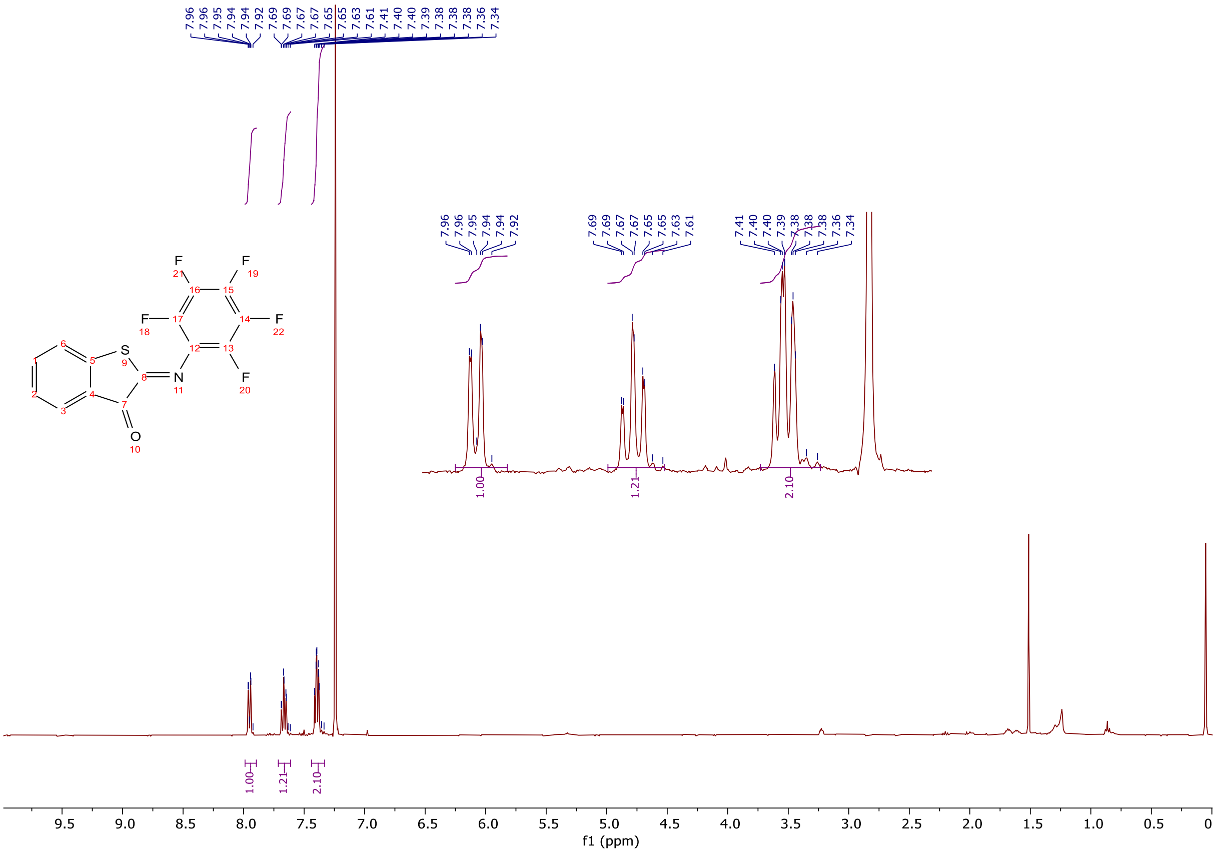

Figure S71 ^{1}H NMR spectrum of compound **1z** in $CDCl_3$.

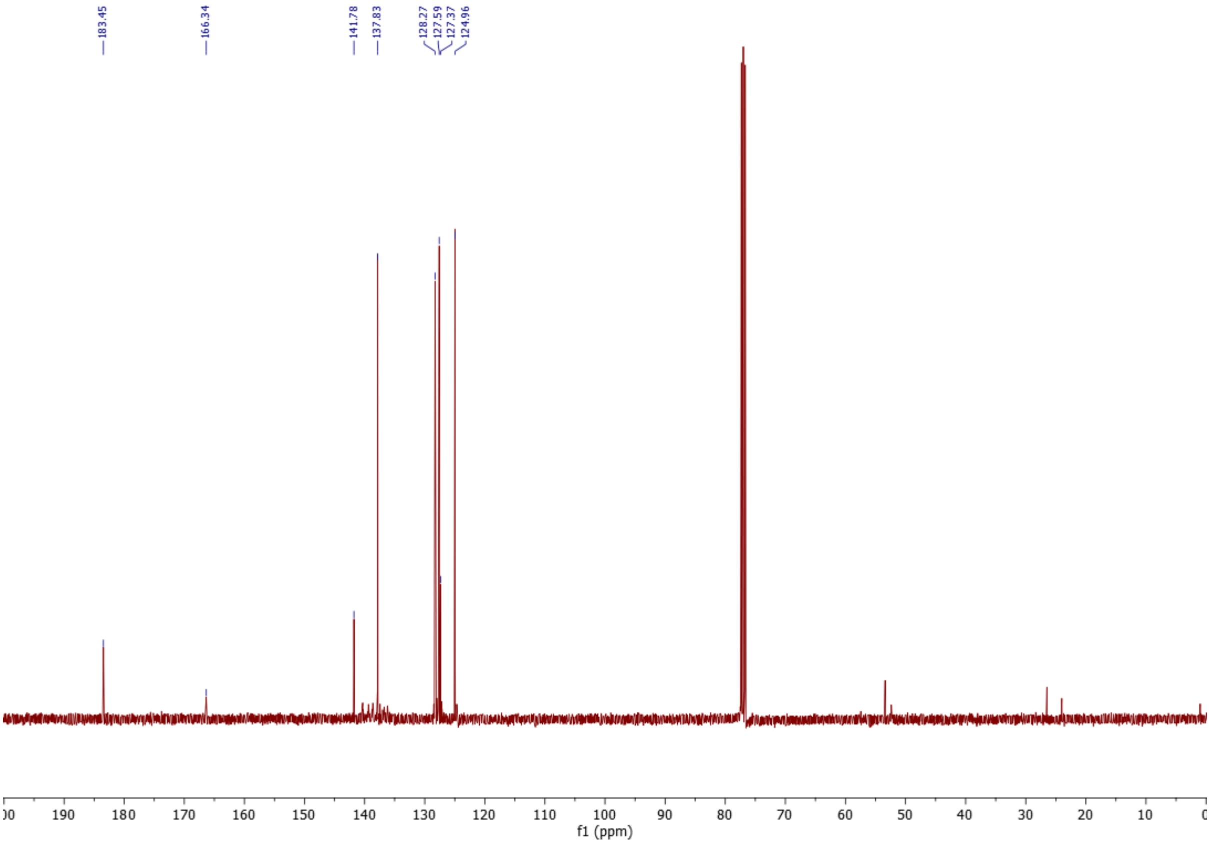

Figure S72 ^{13}C NMR spectrum of compound **1z** in $CDCl_3$.

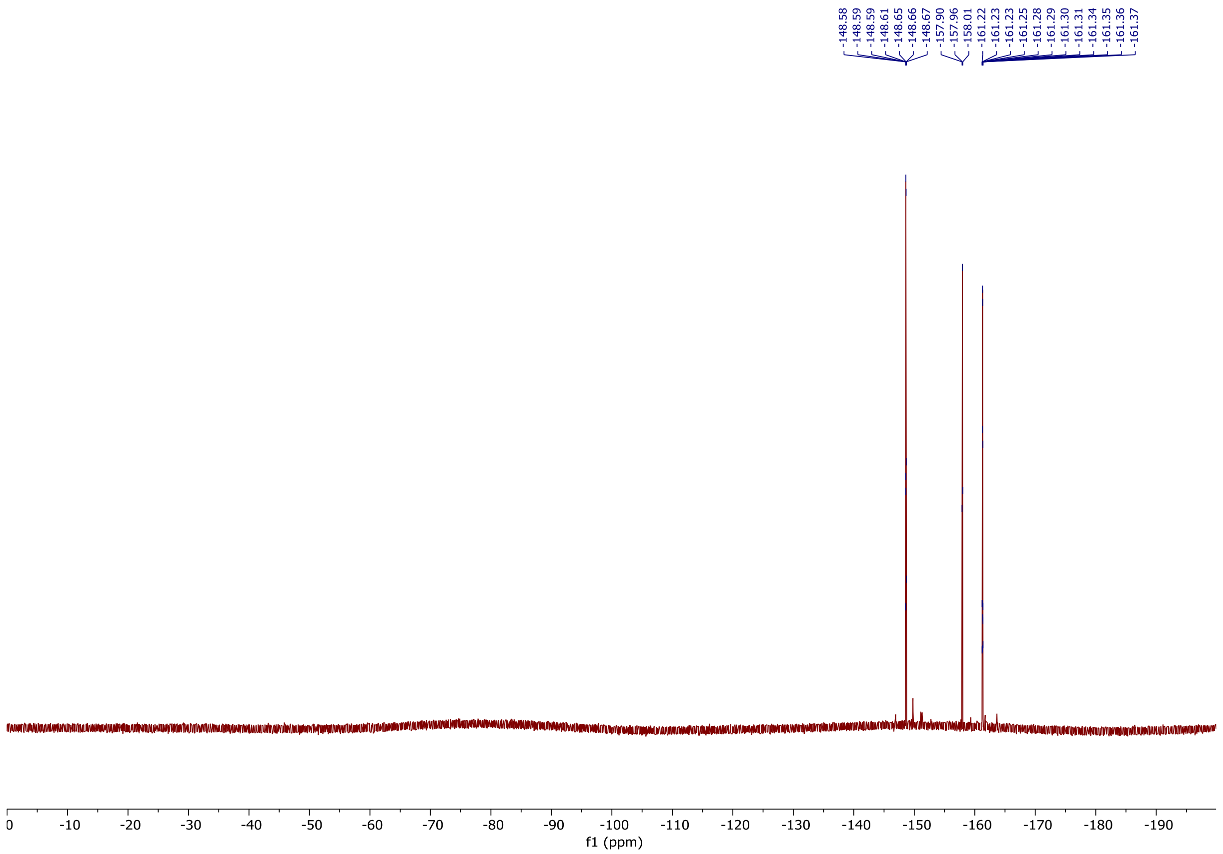

Figure S73 ^{19}F NMR spectrum of compound **1z** in $CDCl_3$.

1α: (*Z*)-2-((4-ethoxy-2,3,5,6-tetrafluorophenyl)imino)benzo[b]thiophen-3(2H)-one

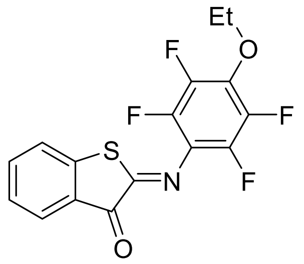

Benzo[b]thiophen-3-yl acetate **8a** (0.17 g, 0.88 mmol) and crude 2,3,4,5,6-pentafluoronitrosobenzene **5z** were dissolved in ethanol (20 mL). The mixture was cooled in an ice-water bath. KOH (25 g/L in EtOH, 0.9 mL, 0.42 mmol) was added dropwise and the mixture was allowed to reach room temperature and stirred vigorously. After 7 d, LC-MS indicated completion. Then, DCM (75 mL) was added, and the mixture washed with brine (2 x 50 mL). The aqueous layer was extracted with DCM (2 x 25 mL) and the combined organic layers were dried over $MgSO_4$ and concentrated *in vacuo*. The crude product was purified by flash chromatography (Silicagel 40 – 63 nm, 0-10% EtOAc in *n*-heptane). The product was obtained as an orange solid (17 mg, 0.048 mmol, 4%). Mp: 115-120 °C. ^{1}H NMR (400 MHz, $CDCl_3$) δ 7.93 (dd, J = 7.9, 1.4 Hz, 1H, Ar**H**), 7.65 (td, J = 7.6, 1.4 Hz, 1H, Ar**H**), 7.40 – 7.32 (m, 2H, Ar**H**), 4.31 (q, J = 7.0 Hz, 2H, C**H**$_2$), 1.43 (t, J = 7.0 Hz, 3H, C**H**$_3$). ^{19}F NMR (376 MHz, $CDCl_3$) δ -149.9 (m), -156.7 (m). IR (cm^{-1}): 1721 (C=O, stretch), 1593 (C=C, stretch), 1496 (C-C, stretch), 1281 (C-N, stretch), 1042 (C-F, stretch), 989 (C=C, bend), 743 (C-H, bend). HRMS (ESI+) calc. for. $[M+H]^+$ ($C_{16}H_{10}F_4NO_2S^+$) Exact Mass: 356.04, found: 356.04.

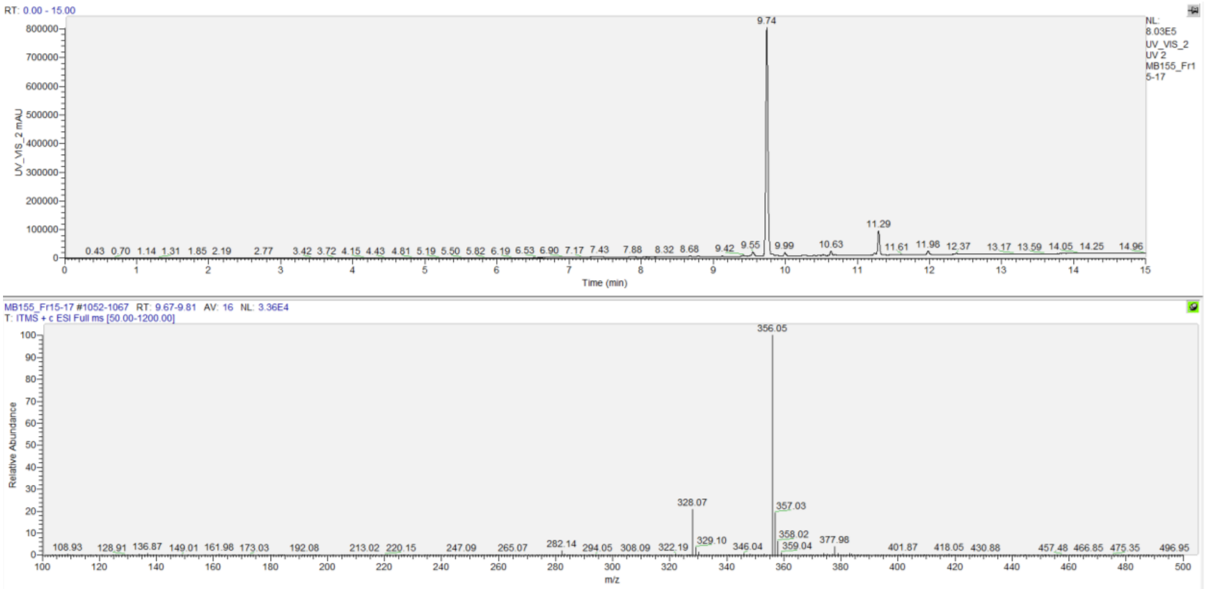

Figure S74 Mass of purified **1α** measured by LC-MS (bottom) at retention time 9.74 minutes (top).

1β: (*Z*)-2-((perchlorophenyl)imino)benzo[b]thiophen-3(2H)-one

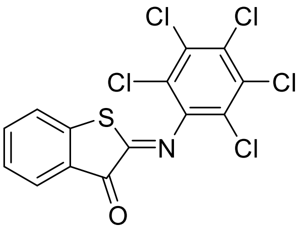

Benzo[b]thiophen-3-yl acetate **8a** (25 mg, 0.13 mmol) and crude 2,3,4,5,6-pentachloronitrosobenzene **5β** were dissolved in ethanol (1.0 mL). The mixture was cooled on an ice-water bath. KOH (25 g/L in EtOH, 0.2 mL, 0.89 mmol) was added dropwise and the mixture was allowed to reach room temperature and stirred vigorously. After 4 h, LC-MS indicated completion. Then, DCM (15 mL) was added, and the mixture washed with brine (2 x 25 mL). The aqueous layer was extracted with DCM (3 x 10 mL) and the combined organic layers were dried over $MgSO_4$ and concentrated *in vacuo*. The crude product was purified by flash chromatography (Silicagel 40 – 63 nm, 0-5% EtOAc in petroleum ether). The product was obtained as an orange solid (3.4 mg, 0.0080 mmol, 5%). ^1H NMR (400 MHz, $CDCl_3$) δ 7.96 (d, *J* = 7.6 Hz, 1H, Ar**H** (3)), 7.66 (app td, *J* = 7.7, 1.4 Hz, 1H, Ar**H** (2)), 7.38 (m, *J* = 7.7 Hz, 2H, Ar**H** (1 and 6)). HRMS (ESI+) calc. for. $[M+Na]^+$ ($C_{14}H_4Cl_5NOS^+$) Exact Mass: 433.8314, found: 433.8319.

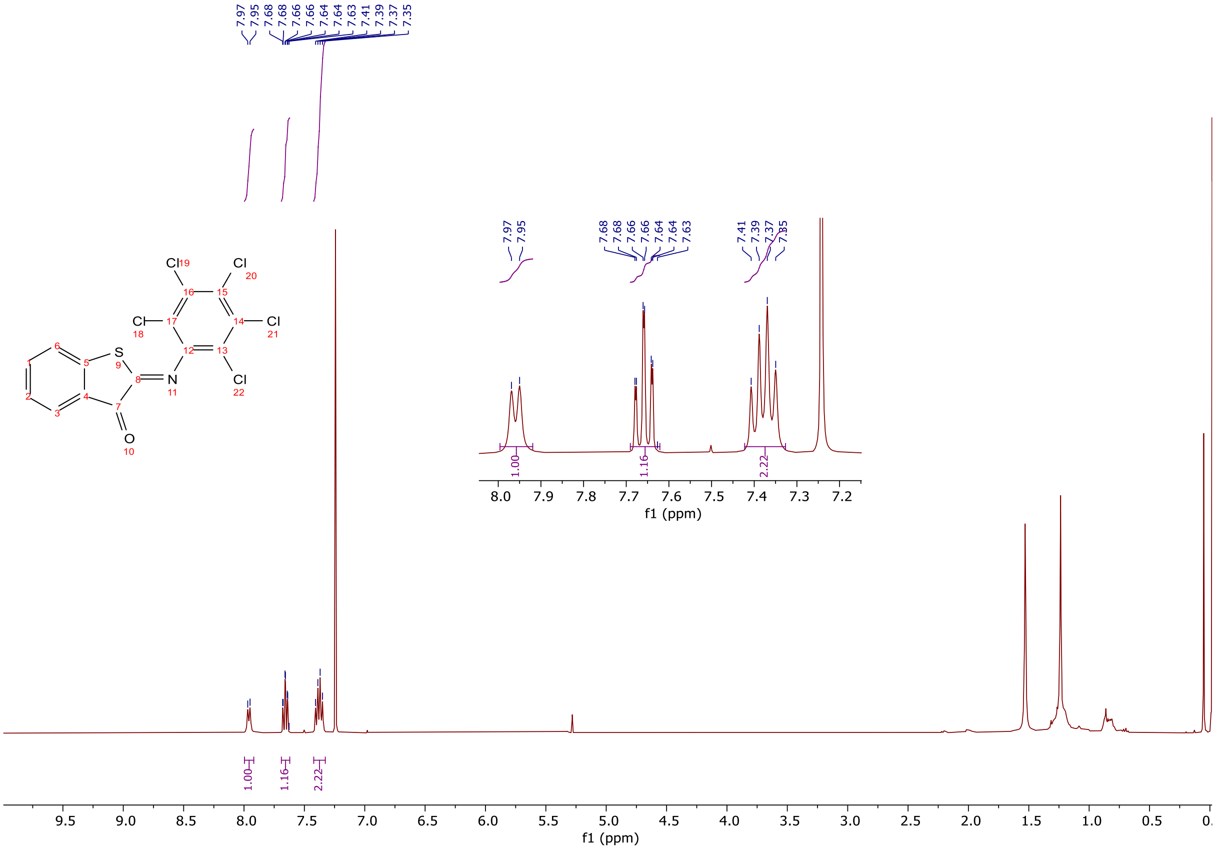

Figure S75 ^{1}H NMR spectrum of compound **1β** in $CDCl_3$.

1γ: (*Z*)-S-(2,3,5,6-tetrafluoro-4-((3-oxobenzo[b]thiophen-2(3H)-ylidene)amino)phenyl)-*L*-cysteine

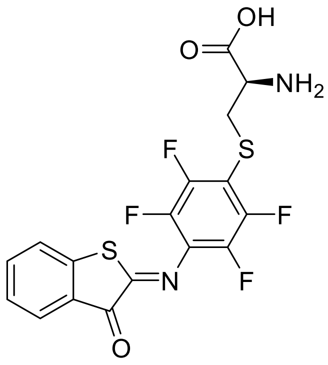

2,3,4,5,6-pentafluoronitrosobenzene **1z** (0.10 g, 0.31 mmol, 1.0 eq) and *L*-cysteine (39 mg, 0.32 mmol, 1.0 eq) were dissolved in DMSO (15 mL). The mixture was stirred at room temperature for 12 h and was purified by preparatory-HPLC to yield the product as an orange solid (2.0 mg, 0.0050 mmol, 2%). HRMS (ESI+) calc. for. $[M+H]^+$ ($C_{17}H_{10}F_4N_2O_3S_2^+$) Exact Mass: 431.0142, found: 431.0137.

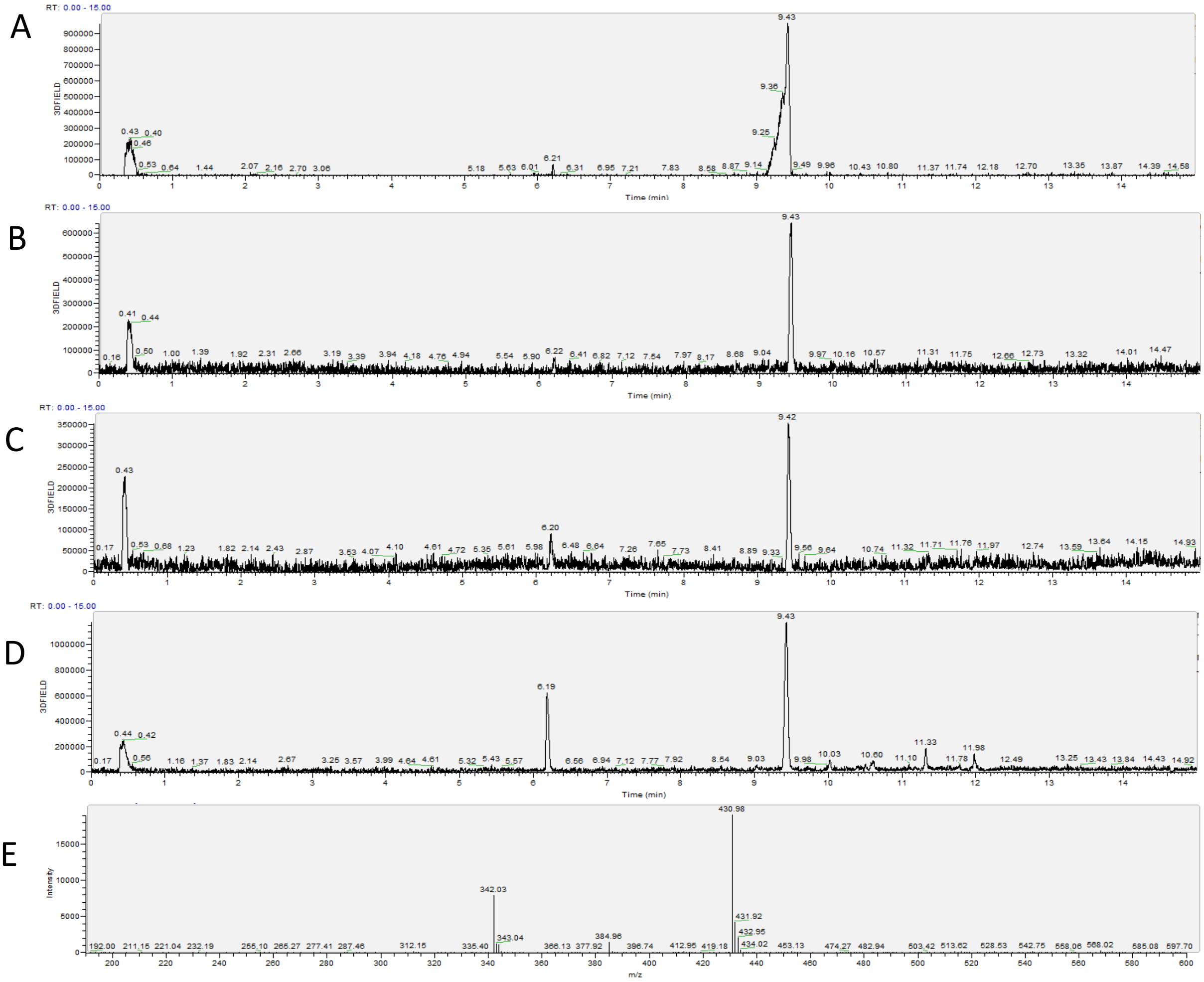

Figure S2.76 LC-trace of a sample of **1γ** taken after 2 hours (**A**), 4 hours (**B**), 6 hours, and 8 hours (**D**). **E**) Mass-spectrum (positive mode) of the peak corresponding to 6.20 minutes.

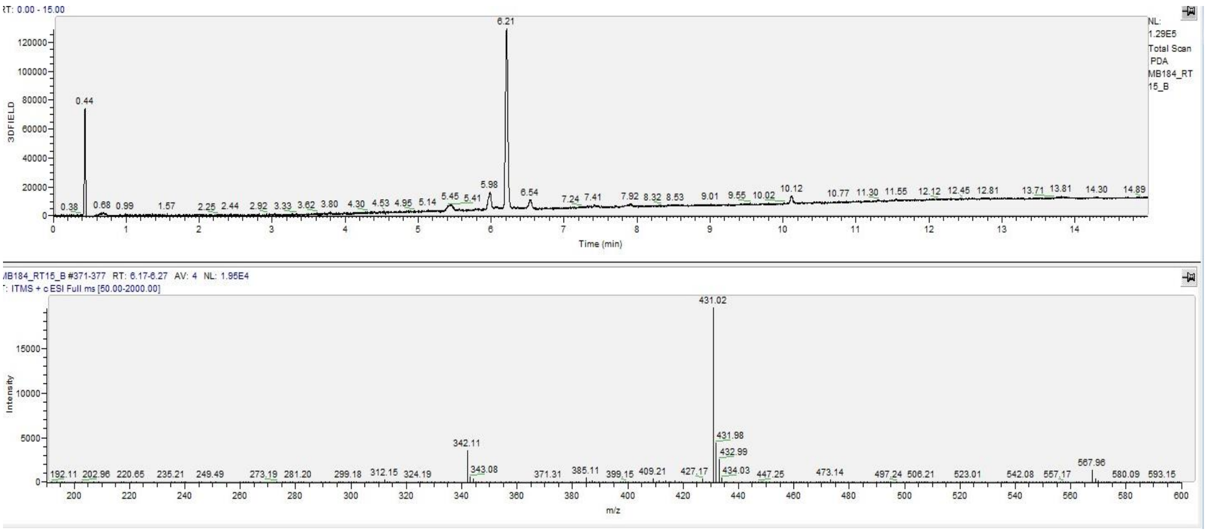

Figure S77 Mass of purified **1γ** measured by LC-MS (bottom) at retention time 6.21 minutes (top).

S2.1. Stability tests of 1z and 1β.

The absorbance at λ = 420 nm of a solution of ~150 μM of **1z** or **1β** in MeOH was monitored at 20 °C for 1 hour in the dark, followed by an additional hour under continuous irradiation with λ = 420 nm light. This was repeated with a fresh 150 μM solution of **1z** or **1β** at 40 °C.

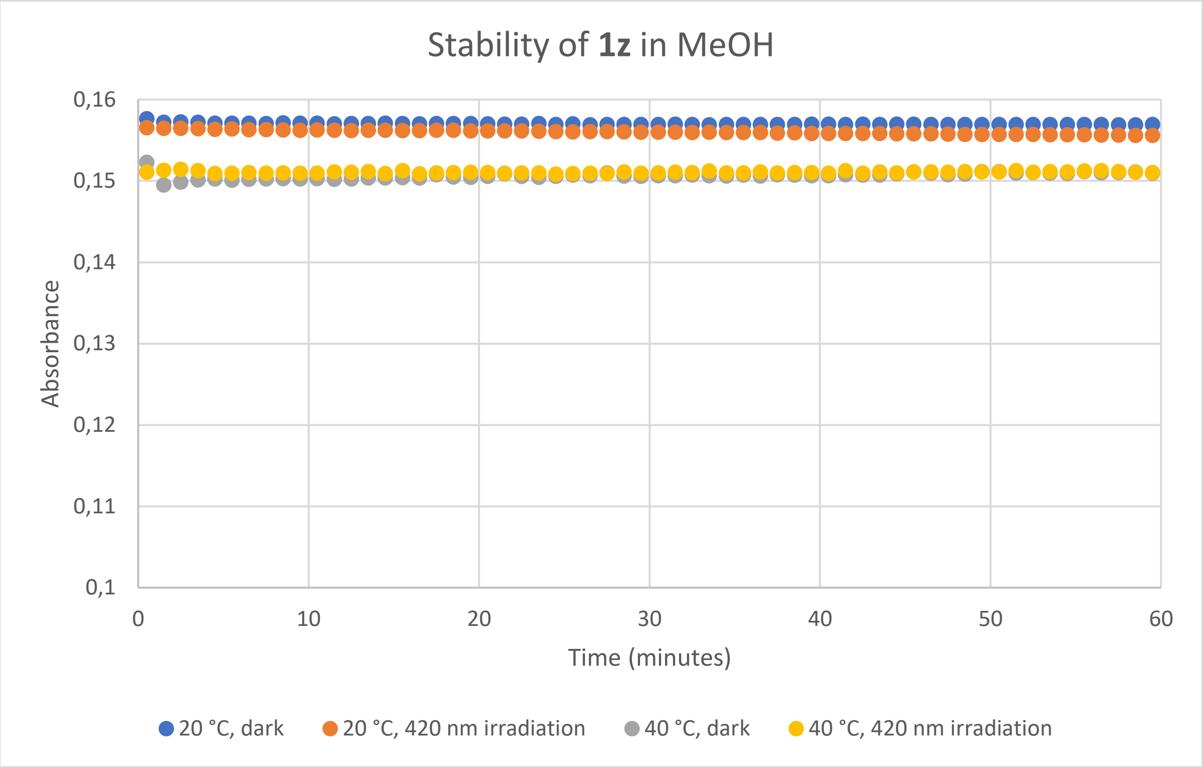

Figure S78 Absorbance of **1z** measured at λ = 420 nm at 20 °C and at 40 °C, with or without irradiation at λ = 420 nm.

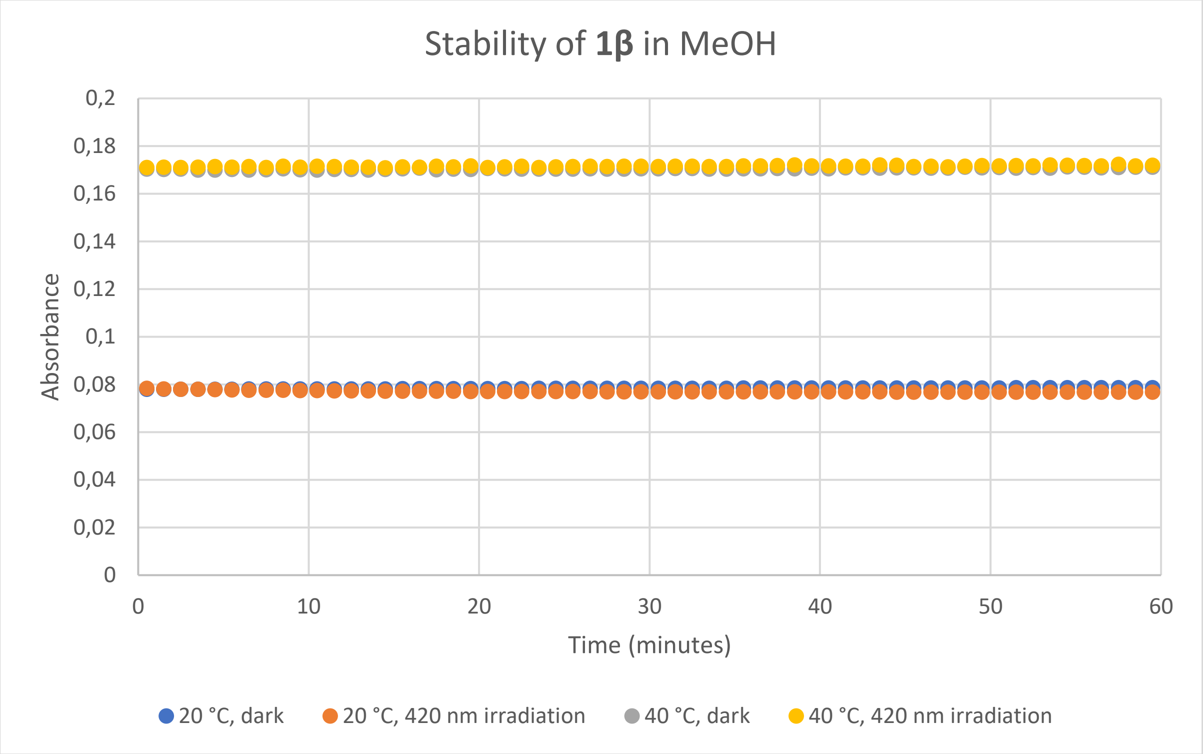

Figure S79 Absorbance of **1β** measured at λ = 420 nm at 20 °C and at 40 °C, with or without irradiation at λ = 420 nm.

S3. Nanosecond transient absorption spectroscopy

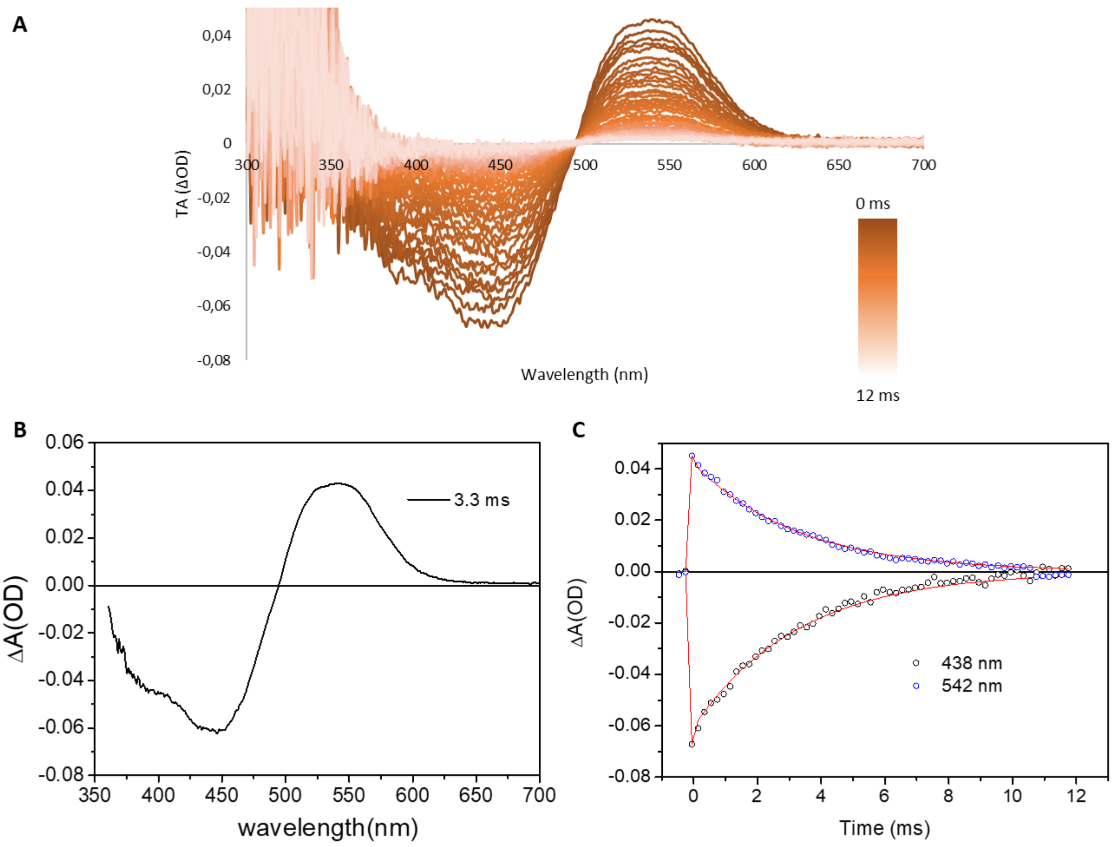

Figure S80 A) Transient absorption of **1b** in MeOH at room temperature. The sample was irradiated with a 455 nm light pulse, upon which the spectrum was recorded in steps of 0.2 ms increasing delay. **B**) EADS obtained by global analysis of the transient absorption data reported in **Figure S80a**. The exponential decay time extracted from global analysis is 3.3 ms. **C**) Kinetic traces recorded at the maximum of the bleaching and excited state absorption signal measured for **1b** in MeOH and fit from global analysis.

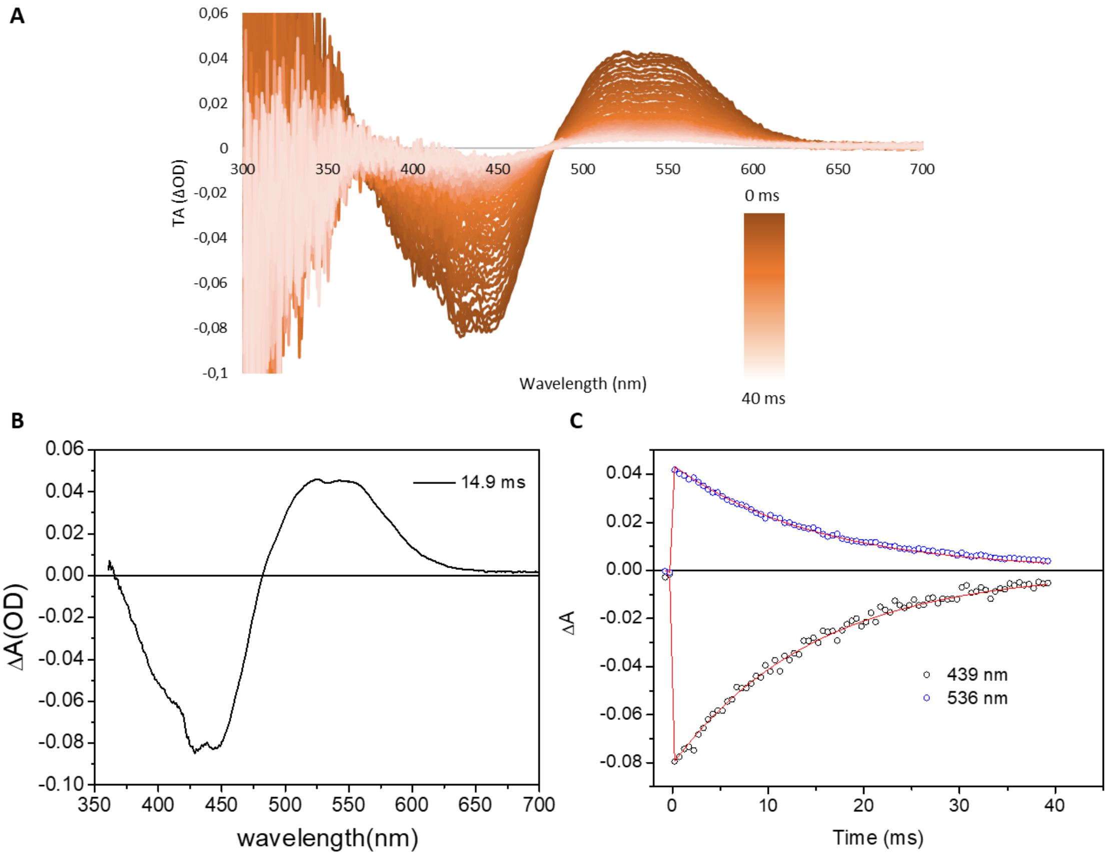

Figure S81 A) Transient absorption of **1c** in MeOH at room temperature. The sample was irradiated with a 430 nm light pulse, upon which the spectrum was recorded in steps of 0.5 ms increasing delay. **B**) EADS obtained by global analysis of the transient absorption data reported in **Figure S81a**. The exponential decay time extracted from global analysis is 14.9 ms. **C**) Kinetic traces recorded at the maximum of the bleaching and excited state absorption signal measured for **1c** in MeOH and fit from global analysis.

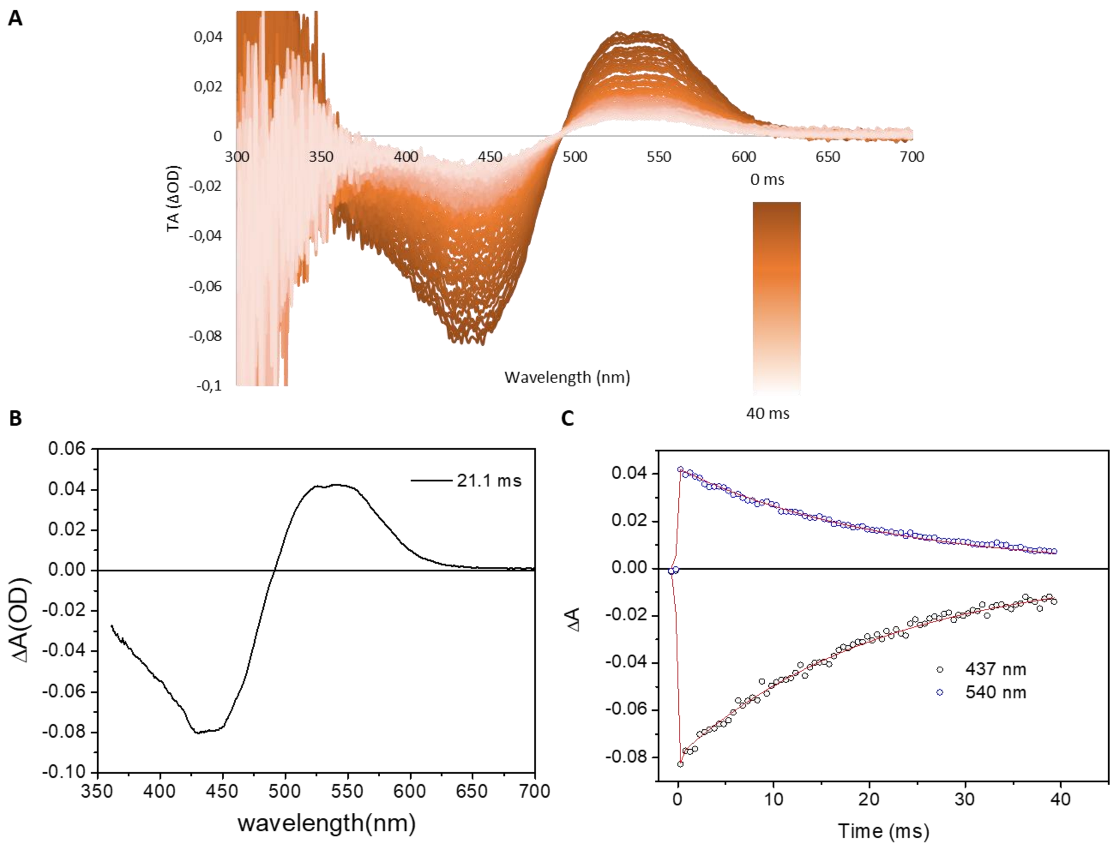

Figure S82 A) Transient absorption of ITI **1d** in MeOH at room temperature. The sample was irradiated with a 430 nm light pulse, upon which the spectrum was recorded in steps of 0.5 ms increasing delay. **B**) EADS obtained by global analysis of the transient absorption data reported in **Figure S82a**. The exponential decay time extracted from global analysis is 21.1 ms. **C**) Kinetic traces recorded at the maximum of the bleaching and excited state absorption signal measured for **1d** in MeOH and fit from global analysis.

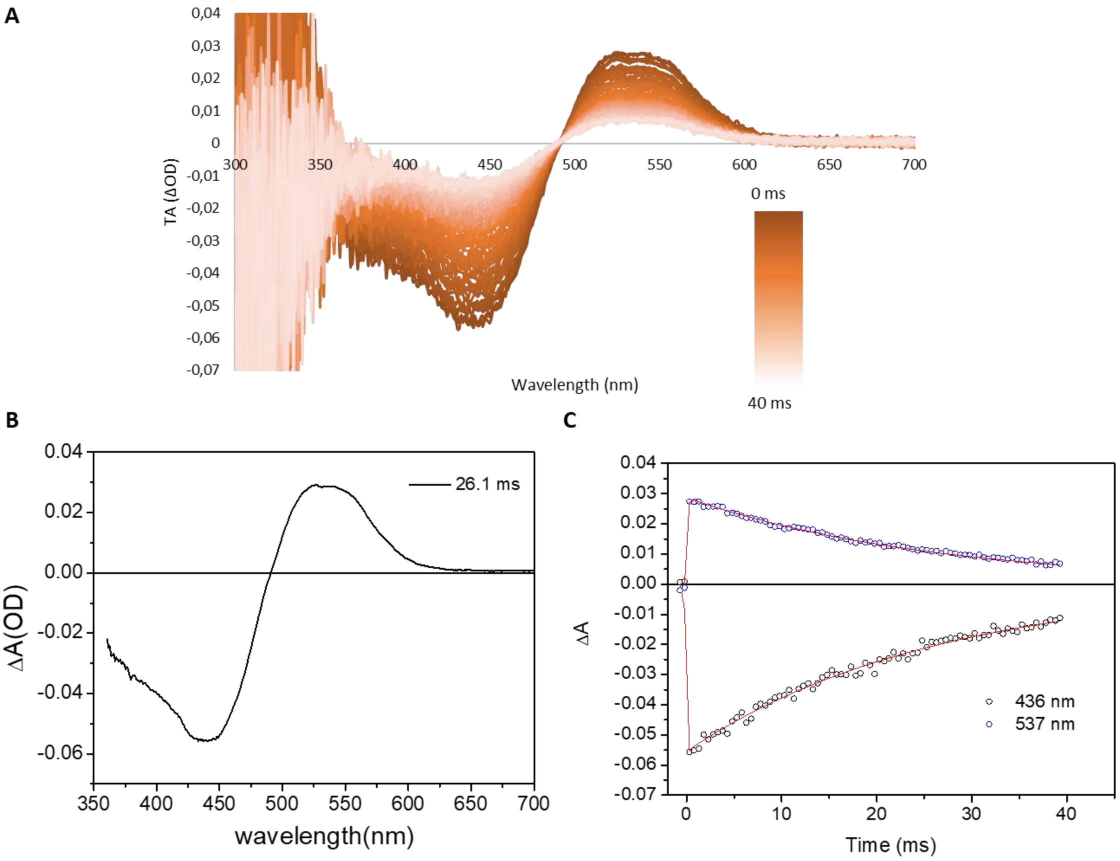

Figure S83 A) Transient absorption of **1e** in MeOH at room temperature. The sample was irradiated with a 430 nm light pulse, upon which the spectrum was recorded in steps of 0.5 ms increasing delay. **B**) EADS obtained by global analysis of the transient absorption data reported in **Figure S83a**. The exponential decay time extracted from global analysis is 26.1 ms. **C**) Kinetic traces recorded at the maximum of the bleaching and excited state absorption signal measured for **1e** in MeOH and fit from global analysis.

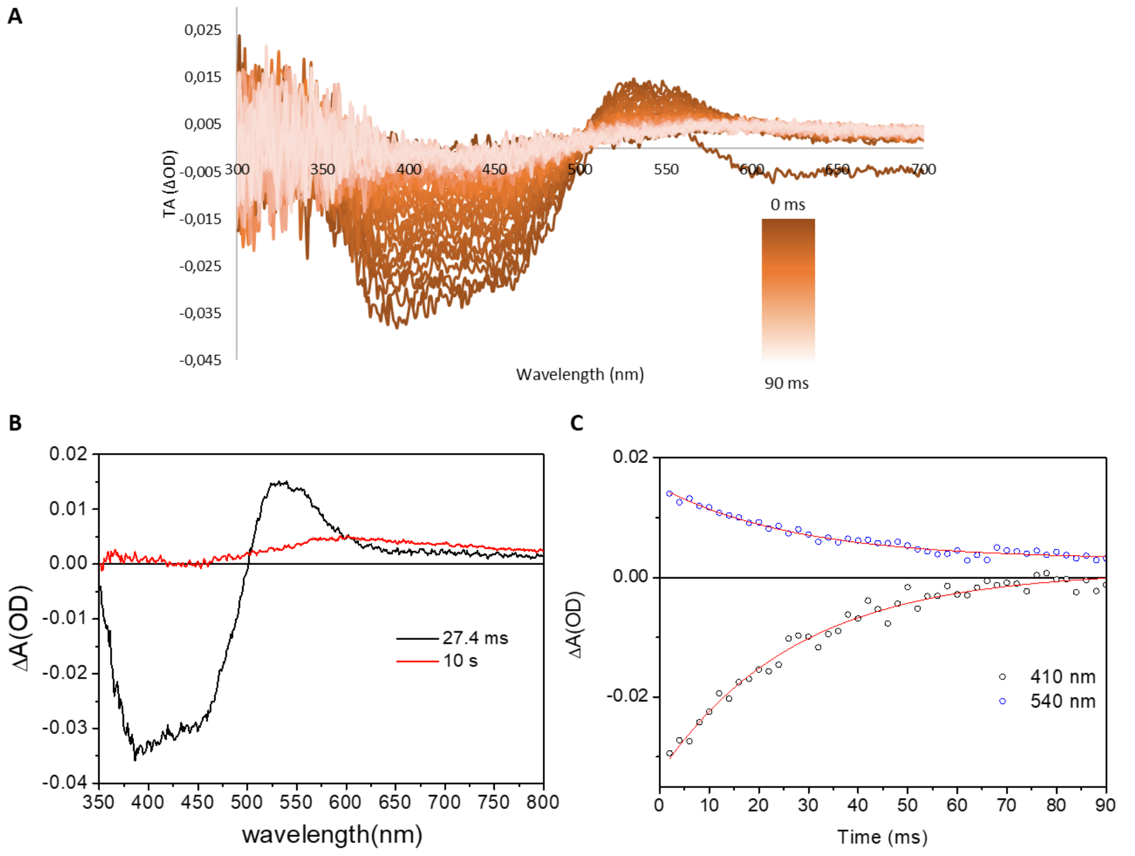

Figure S84 A) Transient absorption of **1f** in MeOH at room temperature. The sample was irradiated with a 460 nm light pulse, upon which the spectrum was recorded in steps of 2 ms increasing delay. **B**) EADS obtained by global analysis of the transient absorption data reported in **Figure S84a**. The exponential decay time extracted from global analysis is 27.4 ms. **C**) Kinetic traces recorded at the maximum of the bleaching and excited state absorption signal measured for **1f** in MeOH and fit from global analysis.

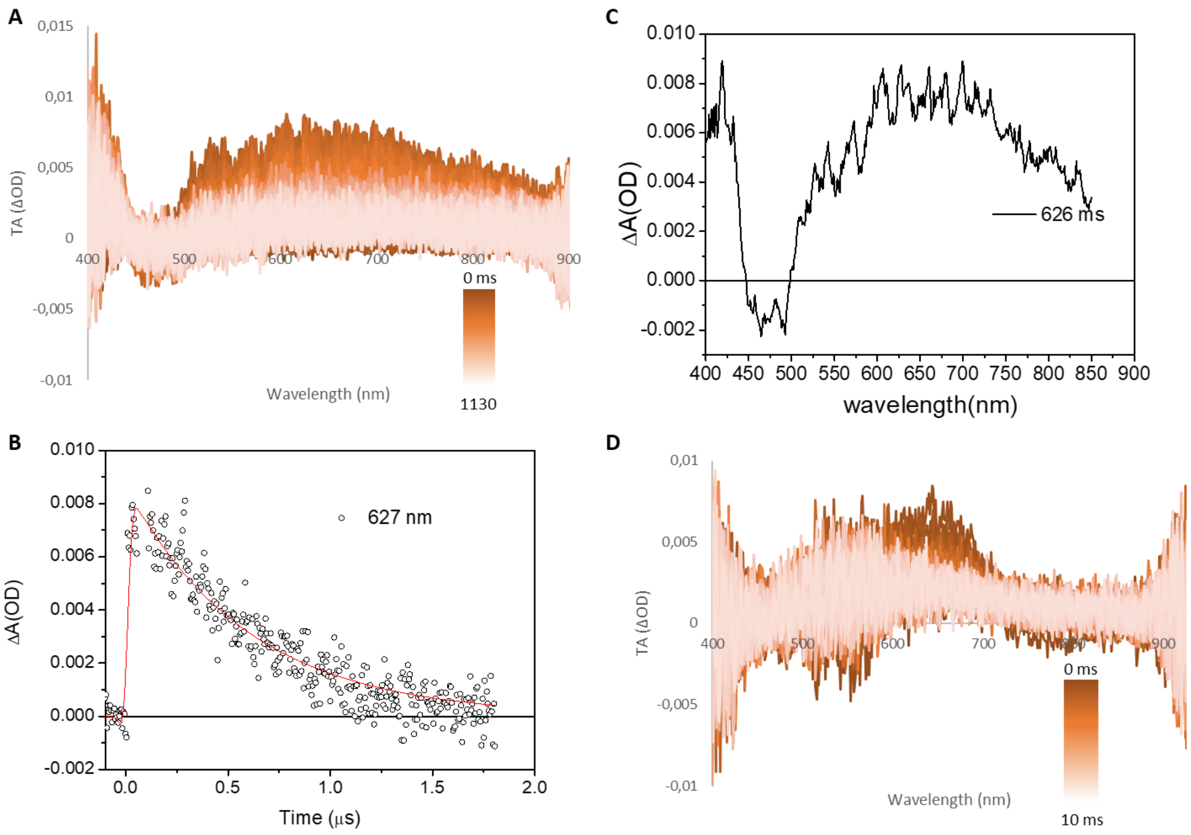

Figure S85 A) Transient absorption of **1g** in MeOH at room temperature. The sample was irradiated with a 430 nm light pulse, upon which the spectrum was recorded in steps of 5 ms increasing delay. **B**) EADS obtained by global analysis of the transient absorption data reported in **Figure S85a**. The exponential decay time extracted from global analysis is 626 ms. **C**) Kinetic traces recorded at the maximum of the bleaching and excited state absorption signal measured for **1g** in MeOH and fit from global analysis. **D**) Transient absorption of **1g** in MeOH at room temperature. The sample was irradiated with a 390 nm light pulse, upon which the spectrum was recorded in steps of 0.2 ms increasing delay.

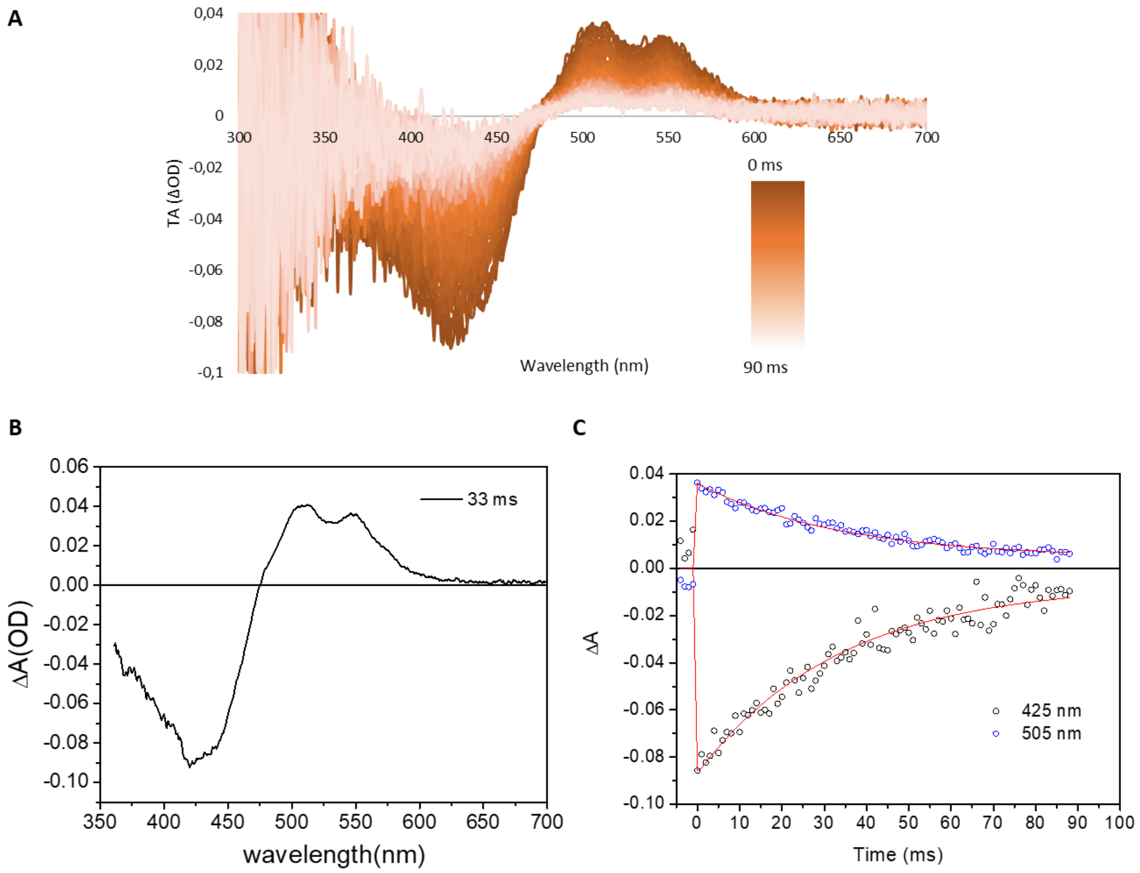

Figure S86 A) Transient absorption of **1i** in MeOH at room temperature. The sample was irradiated with a 430 nm light pulse, upon which the spectrum was recorded in steps of 1 ms increasing delay. **B**) EADS obtained by global analysis of the transient absorption data reported in **Figure S86a**. The exponential decay time extracted from global analysis is 33 ms. **C**) Kinetic traces recorded at the maximum of the bleaching and excited state absorption signal measured for **1i** in MeOH and fit from global analysis.

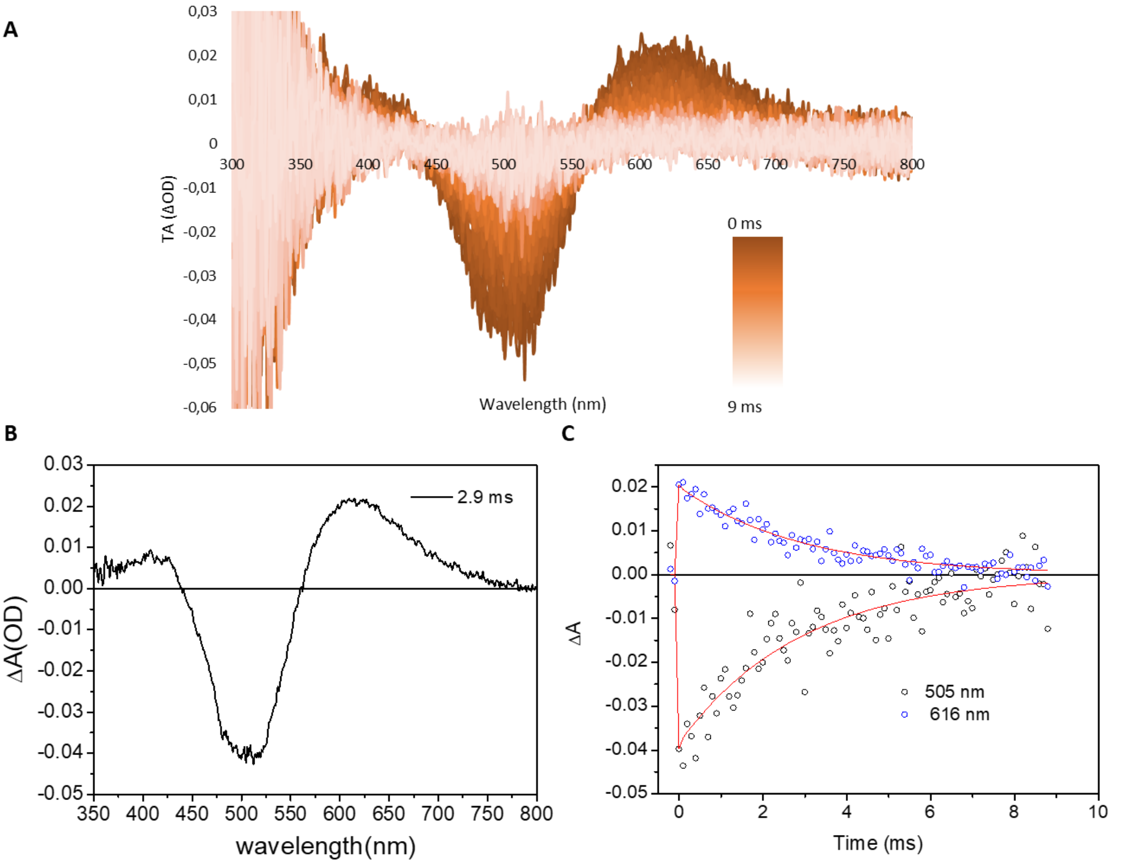

Figure S87 A) Transient absorption of **1k** in MeOH at room temperature. The sample was irradiated with a 430 nm light pulse, upon which the spectrum was recorded in steps of 0.1 ms increasing delay. **B**) EADS obtained by global analysis of the transient absorption data reported in **Figure S87a**. The exponential decay time extracted from global analysis is 2.9 ms. **C**) Kinetic traces recorded at the maximum of the bleaching and excited state absorption signal measured for **1k** in MeOH and fit from global analysis.

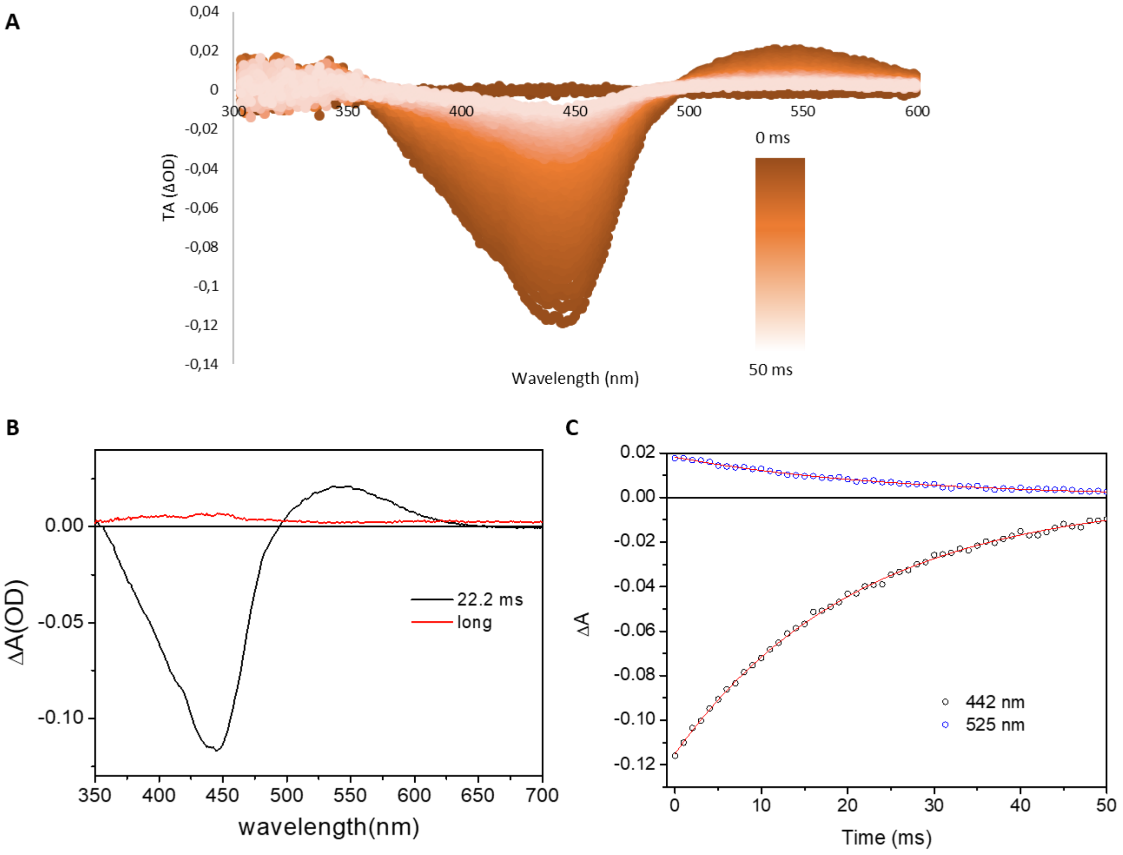

Figure S88 A) Transient absorption of **1l** in MeOH at room temperature. The sample was irradiated with a 430 nm light pulse, upon which the spectrum was recorded in steps of 1 ms increasing delay. **B**) EADS obtained by global analysis of the transient absorption data reported in **Figure S88a**. The exponential decay time extracted from global analysis is 22.2 ms. **C**) Kinetic traces recorded at the maximum of the bleaching and excited state absorption signal measured for **1l** in MeOH and fit from global analysis.

A

B

C

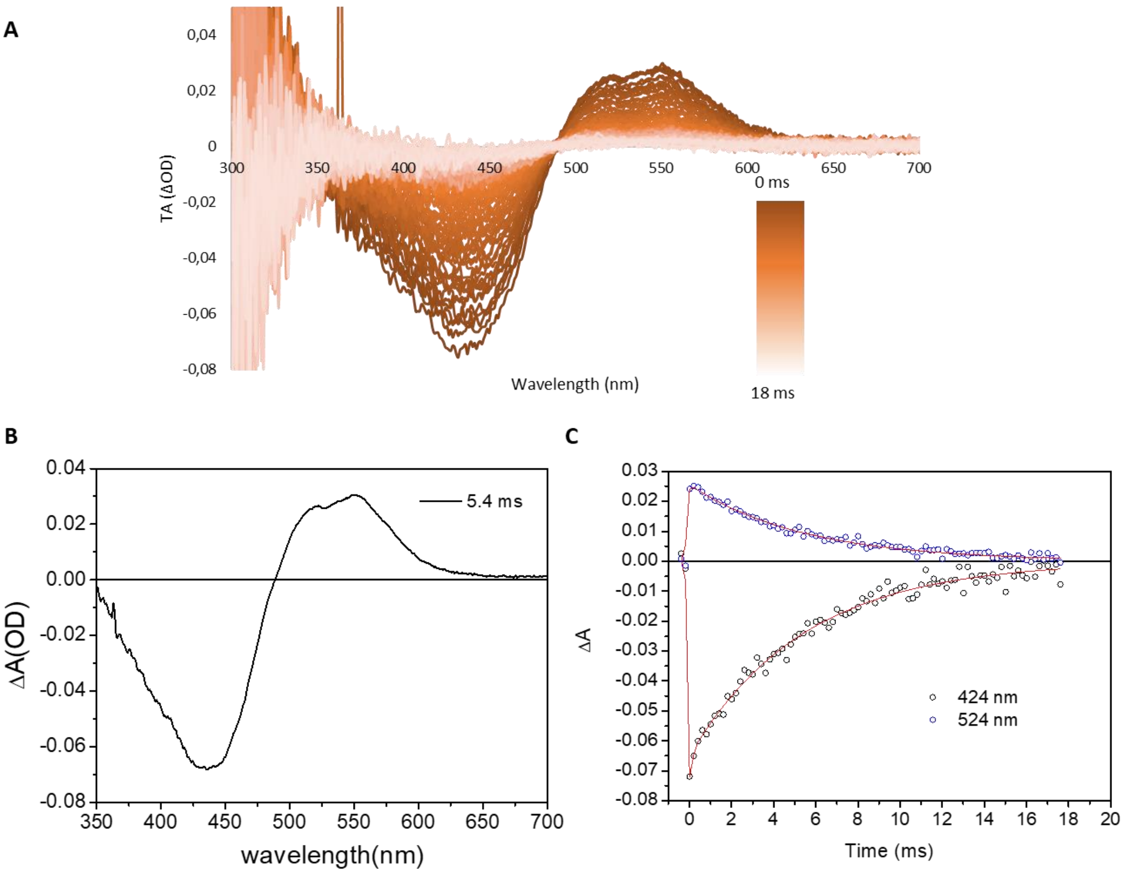

Figure S89 A) Transient absorption of **1m** in MeOH at room temperature. The sample was irradiated with a 430 nm light pulse, upon which the spectrum was recorded in steps of 0.2 ms increasing delay. **B**) EADS obtained by global analysis of the transient absorption data reported in **Figure S89a**. The exponential decay time extracted from global analysis is 5.4 ms. **C**) Kinetic traces recorded at the maximum of the bleaching and excited state absorption signal measured for **1m** in MeOH and fit from global analysis.

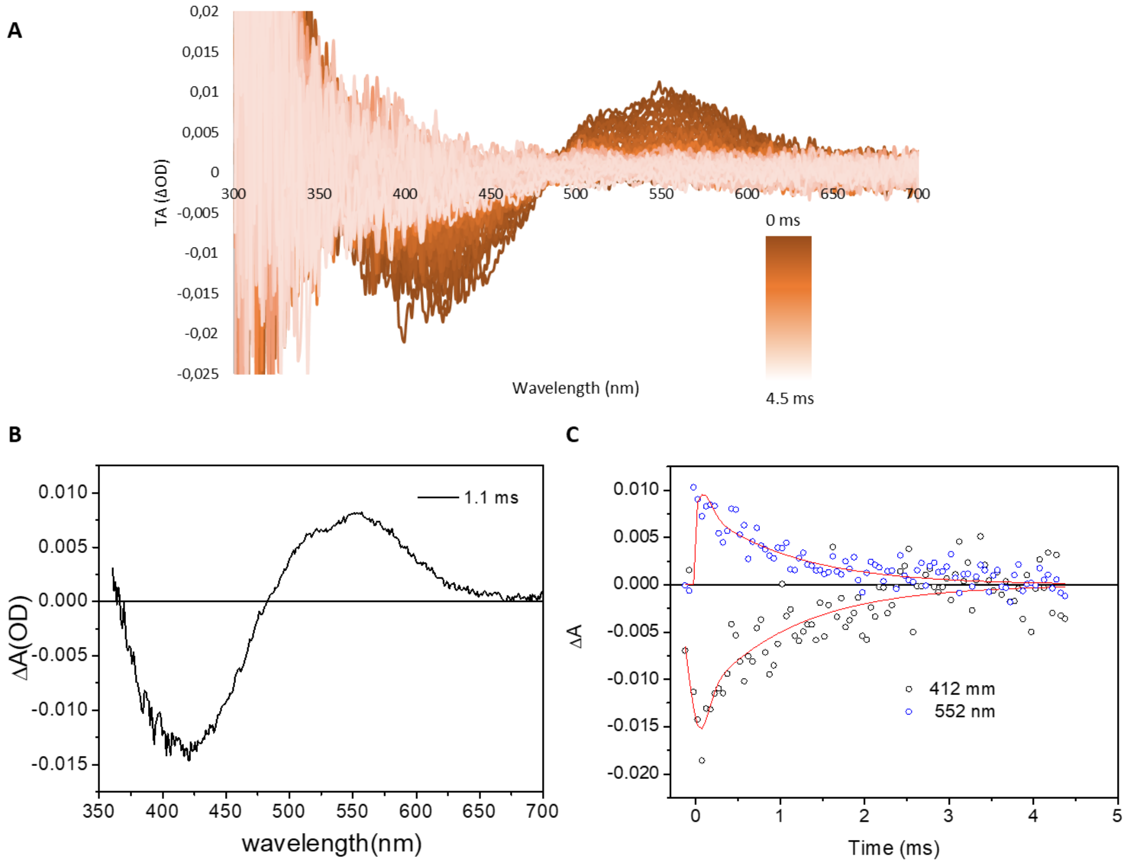

Figure S90 A) Transient absorption of **1n** in MeOH at room temperature. The sample was irradiated with a 420 nm light pulse, upon which the spectrum was recorded in steps of 0.05 ms increasing delay. **B**) EADS obtained by global analysis of the transient absorption data reported in **Figure S90a**. The exponential decay time extracted from global analysis is 1.1 ms. **C**) Kinetic traces recorded at the maximum of the bleaching and excited state absorption signal measured for **1n** in MeOH and fit from global analysis.

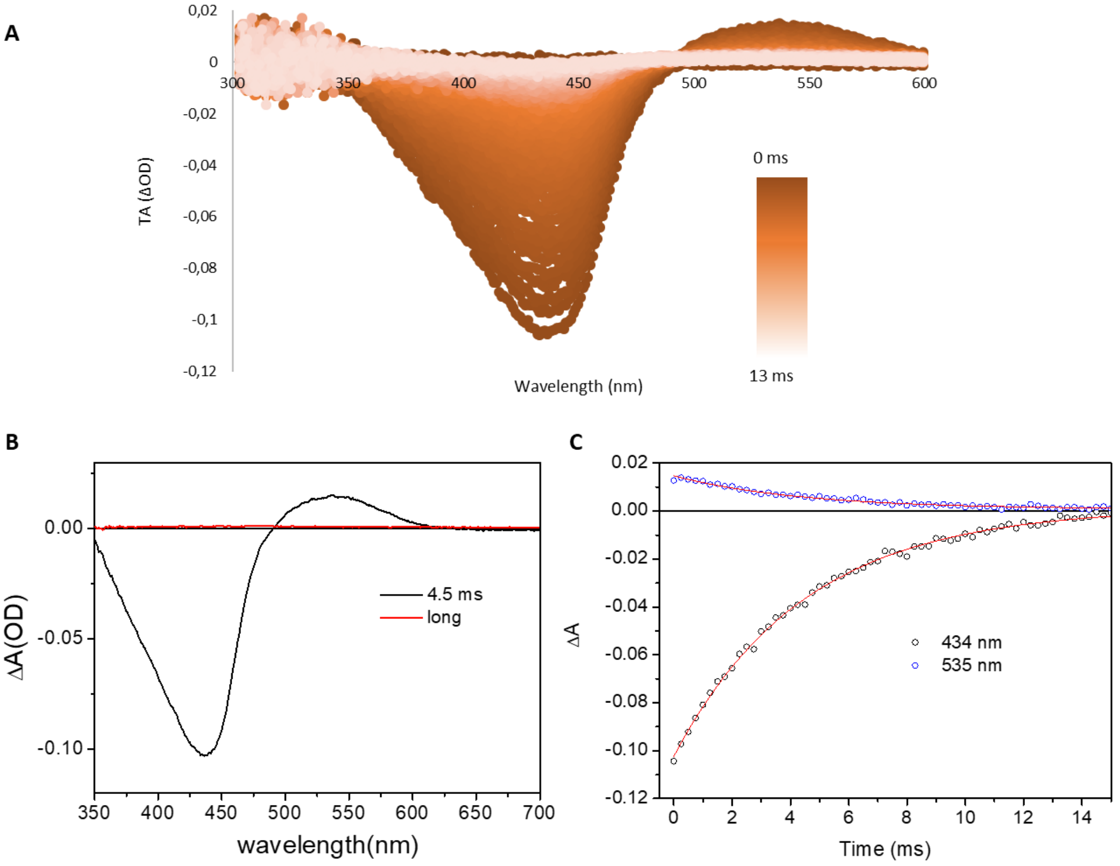

Figure S91 A) Transient absorption of **1o** in MeOH at room temperature. The sample was irradiated with a 430 nm light pulse, upon which the spectrum was recorded in steps of 0.25 ms increasing delay. **B**) EADS obtained by global analysis of the transient absorption data reported in **Figure S91a**. The exponential decay time extracted from global analysis is 4.5 ms. **C**) Kinetic traces recorded at the maximum of the bleaching and excited state absorption signal measured for **1o** in MeOH and fit from global analysis.

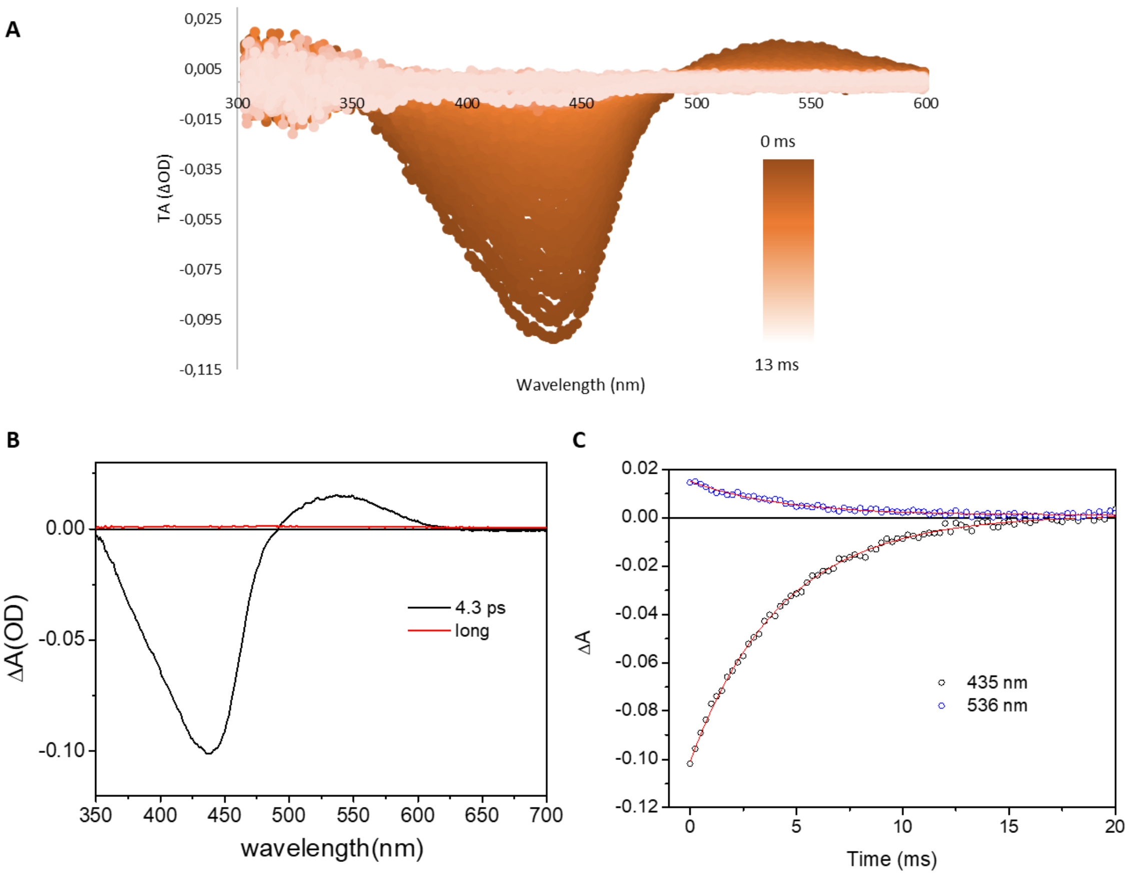

Figure S92 A) Transient absorption of **1p** in MeOH at room temperature. The sample was irradiated with a 430 nm light pulse, upon which the spectrum was recorded in steps of 0.25 ms increasing delay. **B**) EADS obtained by global analysis of the transient absorption data reported in **Figure S92a**. The exponential decay time extracted from global analysis is 4.3 ms. **C**) Kinetic traces recorded at the maximum of the bleaching and excited state absorption signal measured for **1p** in MeOH and fit from global analysis.

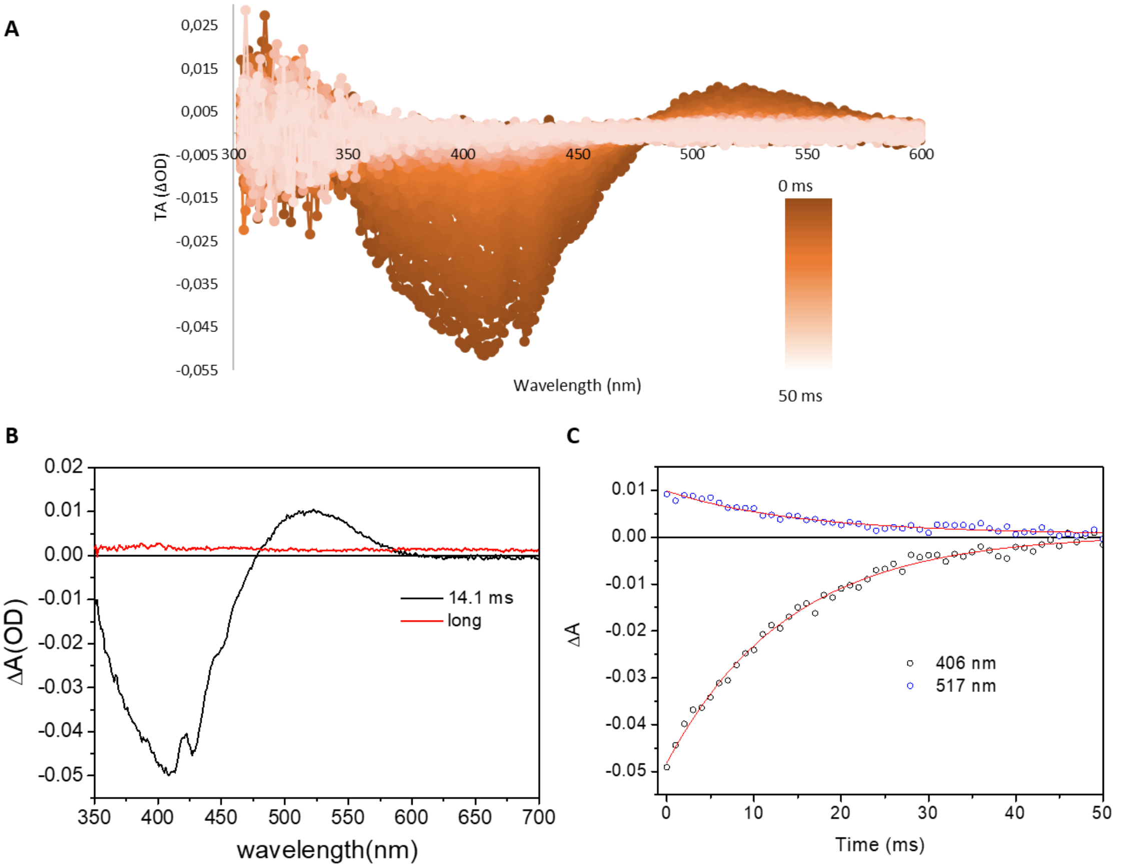

Figure S93 A) Transient absorption of **1q** in MeOH at room temperature. The sample was irradiated with a 430 nm light pulse, upon which the spectrum was recorded in steps of 1 ms increasing delay. **B**) EADS obtained by global analysis of the transient absorption data reported in **Figure S93a**. The exponential decay time extracted from global analysis is 14.1 ms. C**)** Kinetic traces recorded at the maximum of the bleaching and excited state absorption signal measured for **1q** in MeOH and fit from global analysis.

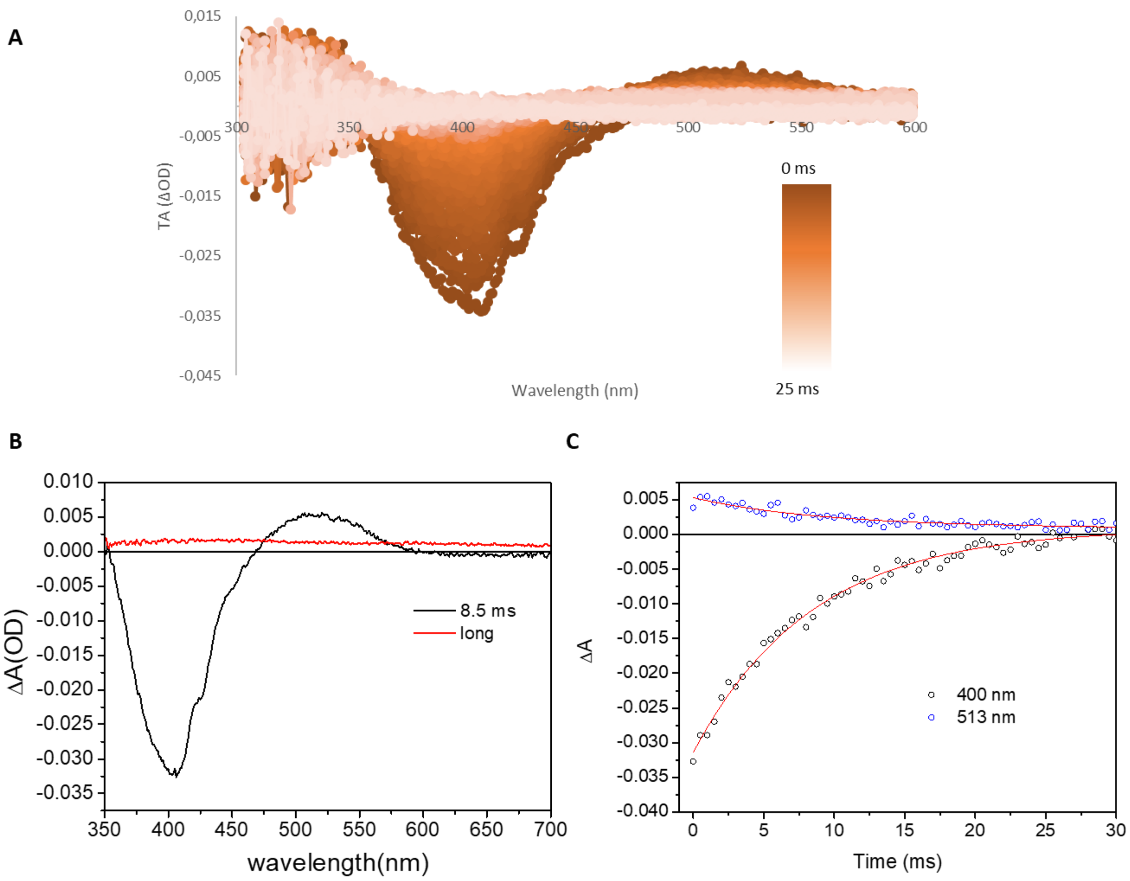

Figure S94 A) Transient absorption of **1r** in MeOH at room temperature. The sample was irradiated with a 430 nm light pulse, upon which the spectrum was recorded in steps of 0.5 ms increasing delay. **B**) EADS obtained by global analysis of the transient absorption data reported in **Figure S94a**. The exponential decay time extracted from global analysis is 8.5 ms. **C**) Kinetic traces recorded at the maximum of the bleaching and excited state absorption signal measured for **1r** in MeOH and fit from global analysis.

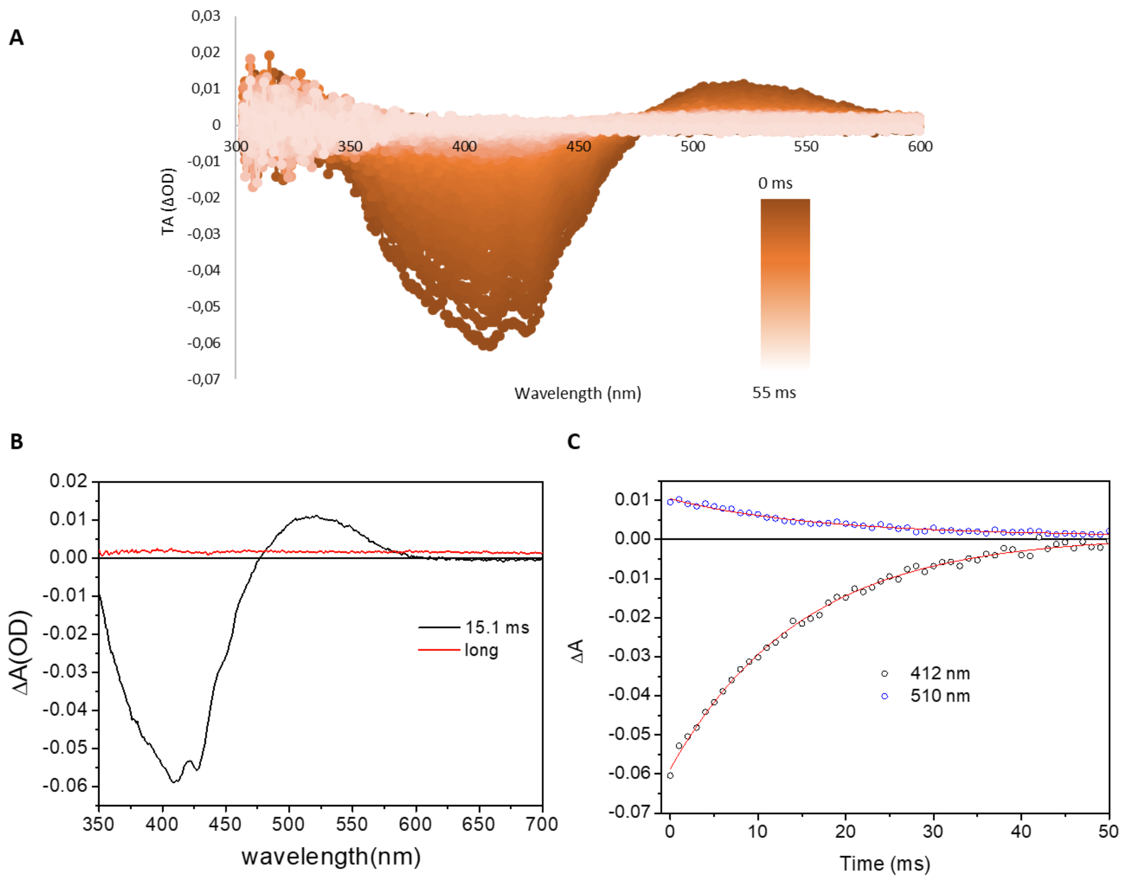

Figure S95 A) Transient absorption of **1s** in MeOH at room temperature. The sample was irradiated with a 430 nm light pulse, upon which the spectrum was recorded in steps of 1 ms increasing delay. **B**) EADS obtained by global analysis of the transient absorption data reported in **Figure S95a**. The exponential decay time extracted from global analysis is 15.1 ms. **C**) Kinetic traces recorded at the maximum of the bleaching and excited state absorption signal measured for **1s** in MeOH and fit from global analysis.

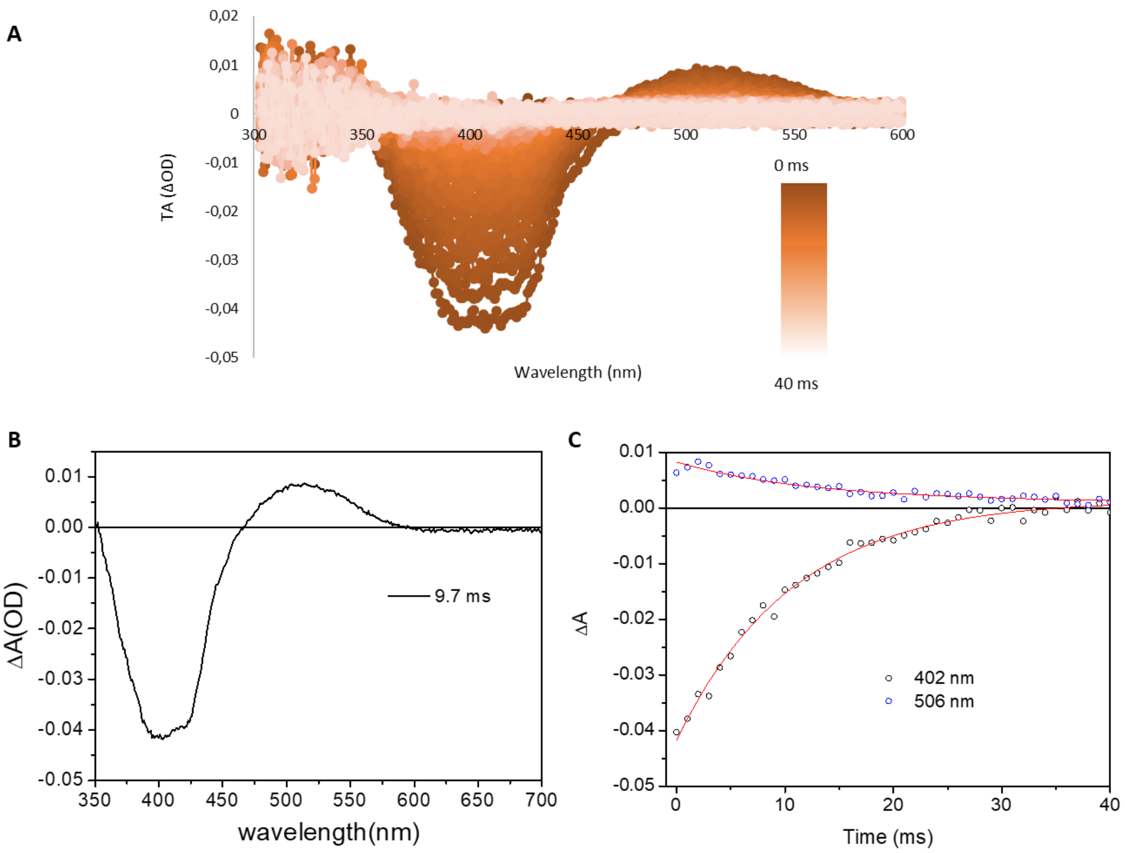

Figure S96 A) Transient absorption of **1t** in MeOH at room temperature. The sample was irradiated with a 430 nm light pulse, upon which the spectrum was recorded in steps of 1 ms increasing delay. **B**) EADS obtained by global analysis of the transient absorption data reported in **Figure S96a**. The exponential decay time extracted from global analysis is 9.7 ms. **C**) Kinetic traces recorded at the maximum of the bleaching and excited state absorption signal measured for **1t** in MeOH and fit from global analysis.

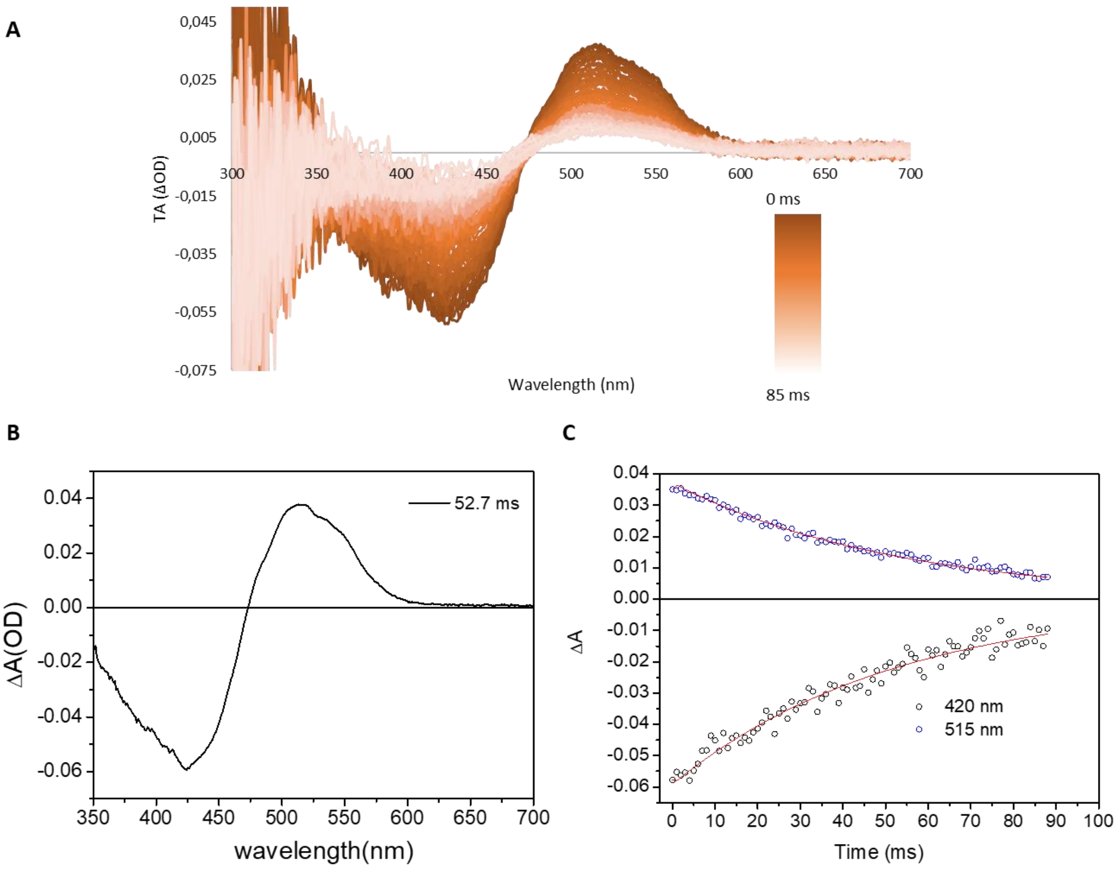

Figure S97 A) Transient absorption of **1u** in MeOH at room temperature. The sample was irradiated with a 430 nm light pulse, upon which the spectrum was recorded in steps of 1 ms increasing delay. **B**) EADS obtained by global analysis of the transient absorption data reported in **Figure S97a**. The exponential decay time extracted from global analysis is 52.7 ms. **C**) Kinetic traces recorded at the maximum of the bleaching and excited state absorption signal measured for **1u** in MeOH and fit from global analysis.

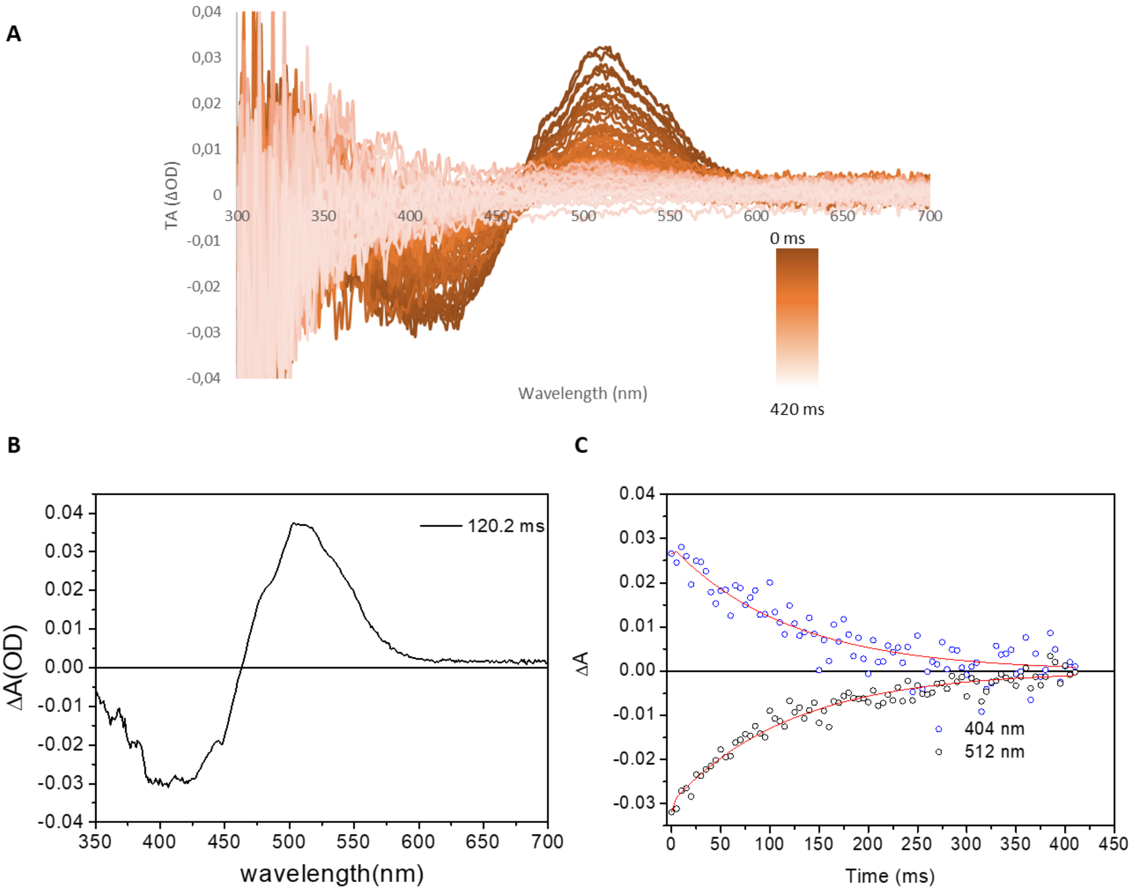

Figure S98 A) Transient absorption **1v** in MeOH at room temperature. The sample was irradiated with a 420 nm light pulse, upon which the spectrum was recorded in steps of 5 ms increasing delay. **B**) EADS obtained by global analysis of the transient absorption data reported in **Figure S98a**. The exponential decay time extracted from global analysis is 120.2 ms. **C**) Kinetic traces recorded at the maximum of the bleaching and excited state absorption signal measured for **1v** in MeOH and fit from global analysis.

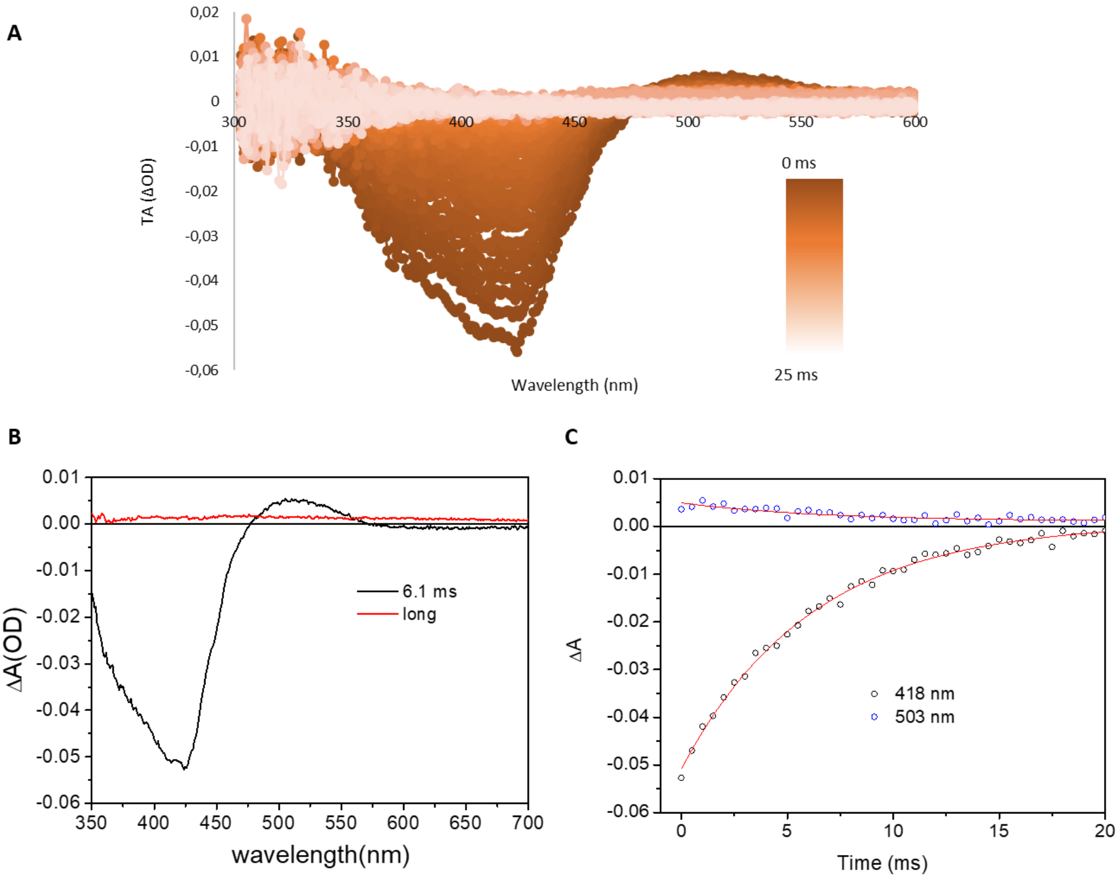

Figure S99 A) Transient absorption of **1w** in MeOH at room temperature. The sample was irradiated with a 430 nm light pulse, upon which the spectrum was recorded in steps of 0.5 ms increasing delay. **B**) EADS obtained by global analysis of the transient absorption data reported in **Figure S99a**. The exponential decay time extracted from global analysis is 6.1 ms. **C**) Kinetic traces recorded at the maximum of the bleaching and excited state absorption signal measured for **1w** in MeOH and fit from global analysis.

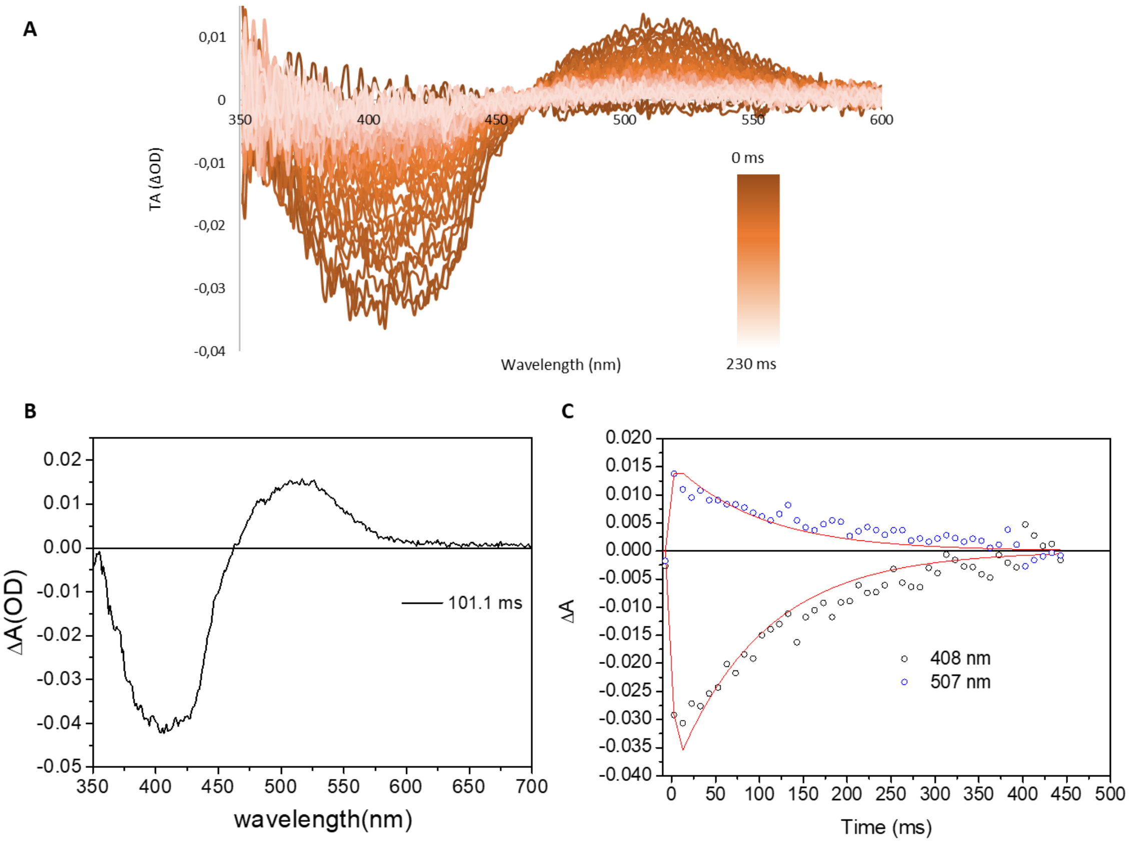

Figure S100 A) Transient absorption of **1x** in MeOH at room temperature. The sample was irradiated with a 420 nm light pulse, upon which the spectrum was recorded in steps of 10 ms increasing delay. **B**) EADS obtained by global analysis of the transient absorption data reported in **Figure S100a**. The exponential decay time extracted from global analysis is 101.1 ms. **C**) Kinetic traces recorded at the maximum of the bleaching and excited state absorption signal measured for **1x** in MeOH and fit from global analysis.

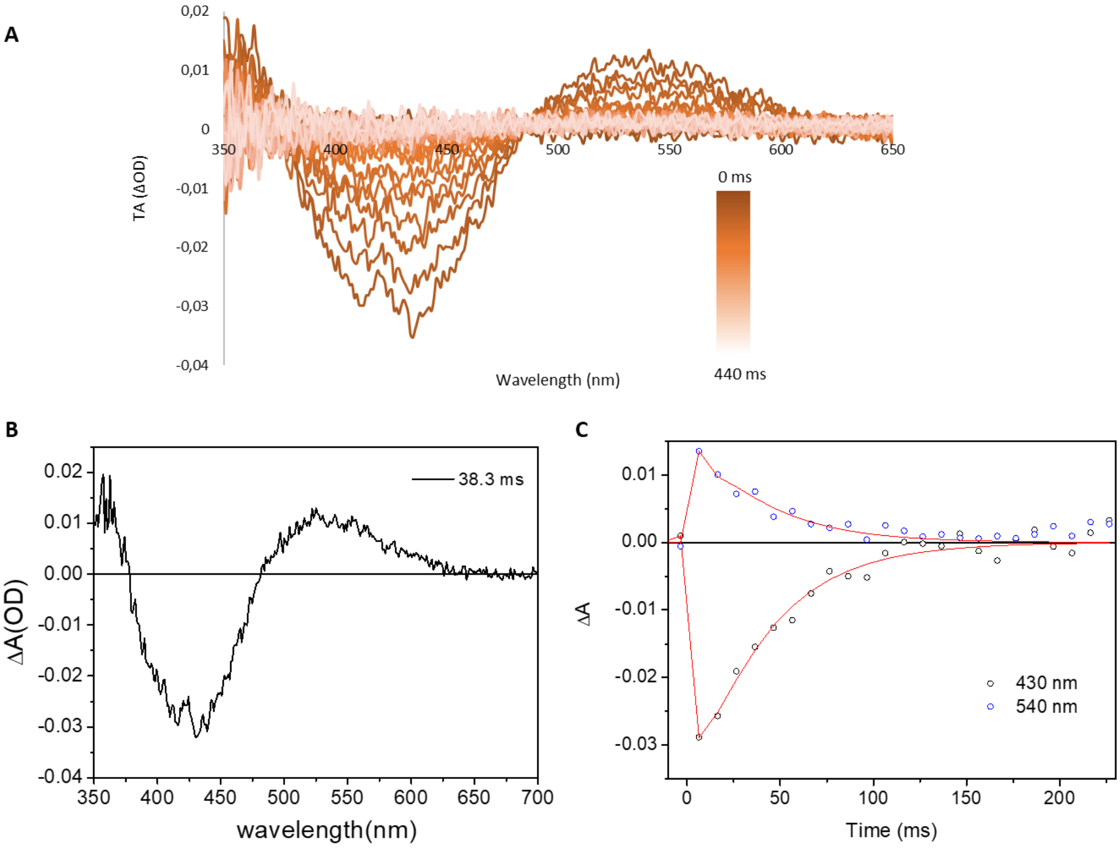

Figure S101 A) Transient absorption of **1y** in MeOH at room temperature. The sample was irradiated with a 420 nm light pulse, upon which the spectrum was recorded in steps of 10 ms increasing delay. **B**) EADS obtained by global analysis of the transient absorption data reported in **Figure S101a**. The exponential decay time extracted from global analysis is 38.3 ms. **C**) Kinetic traces recorded at the maximum of the bleaching and excited state absorption signal measured for **1y** in MeOH and fit from global analysis.

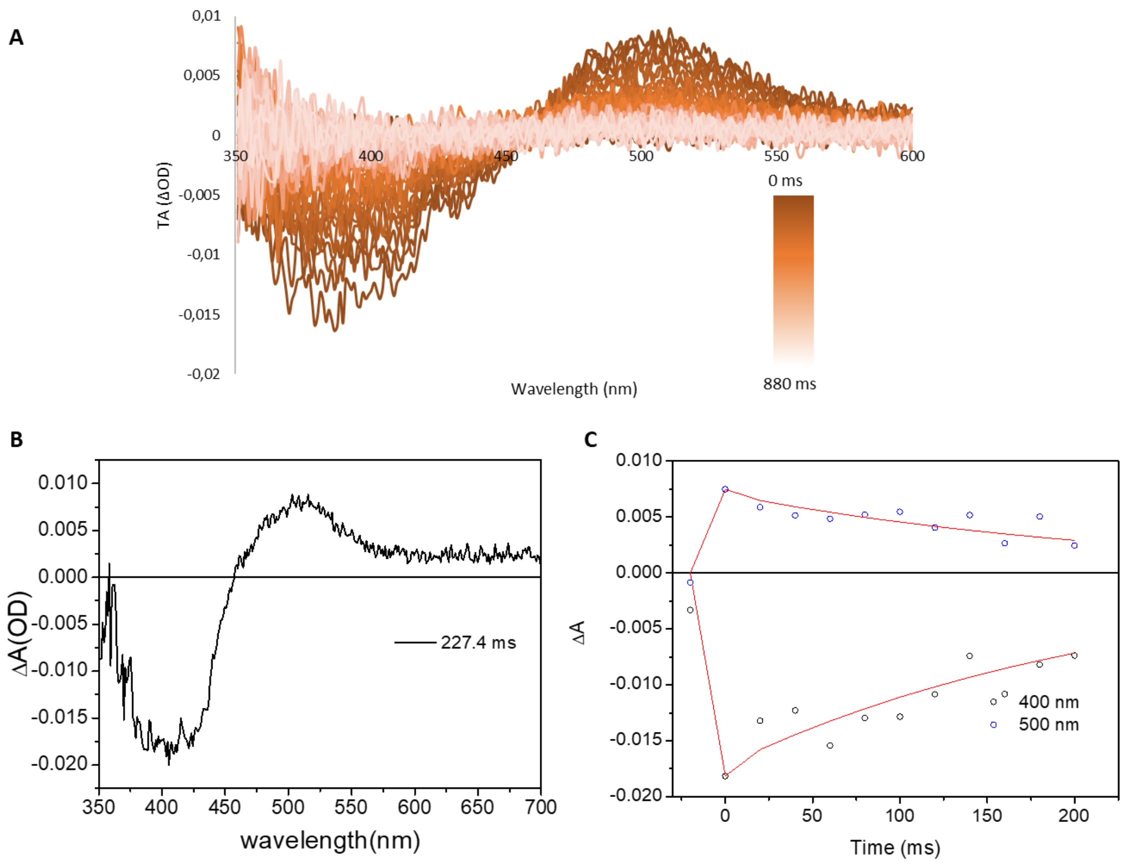

Figure S102 A) Transient absorption of **1z** in MeOH at room temperature. The sample was irradiated with a 420 nm light pulse, upon which the spectrum was recorded in steps of 20 ms increasing delay. **B**) EADS obtained by global analysis of the transient absorption data reported in **Figure S102a**. The exponential decay time extracted from global analysis is 227.4 ms. **C**) Kinetic traces recorded at the maximum of the bleaching and excited state absorption signal measured for **1z** in MeOH and fit from global analysis.

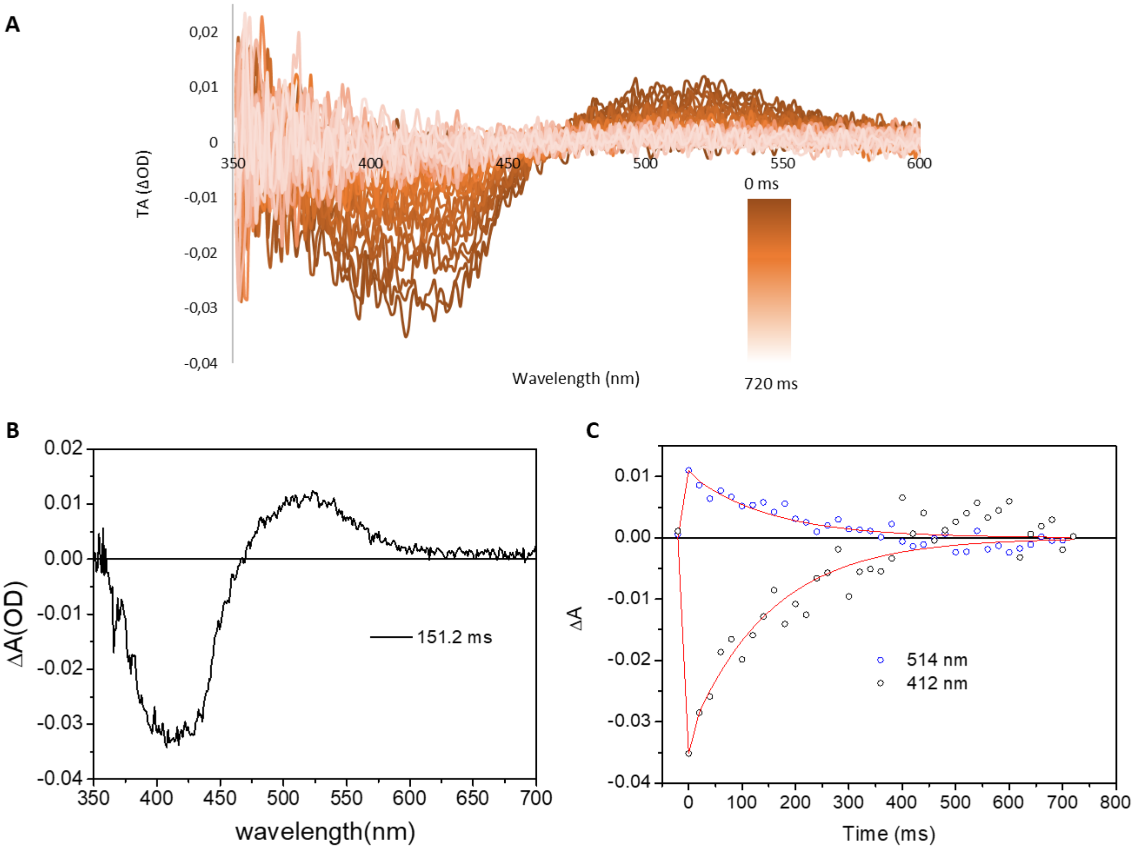

Figure S103 A). Transient absorption of **1α** in MeOH at room temperature. The sample was irradiated with a 420 nm light pulse, upon which the spectrum was recorded in steps of 20 ms increasing delay. **B**) EADS obtained by global analysis of the transient absorption data reported in **Figure S103a**. The exponential decay time extracted from global analysis is 151.2 ms. **C**) Kinetic traces recorded at the maximum of the bleaching and excited state absorption signal measured for **1α** in MeOH and fit from global analysis.

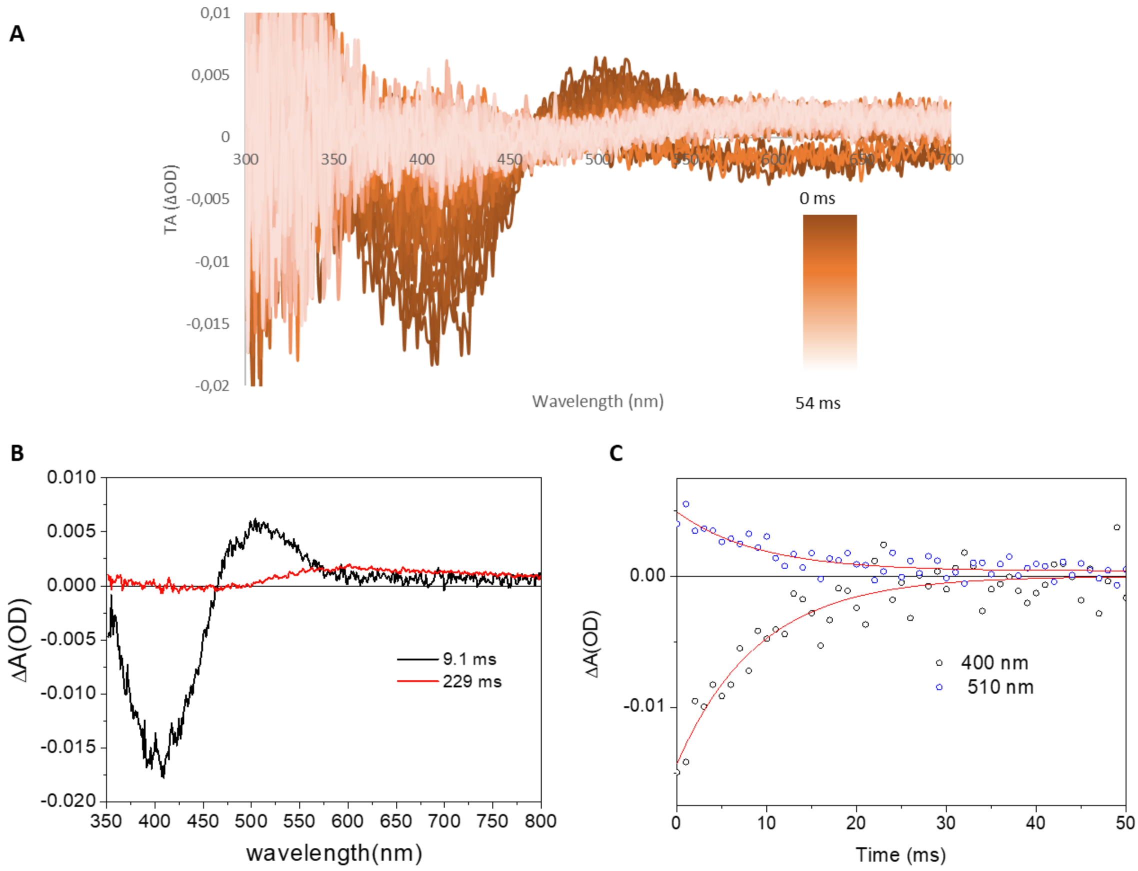

Figure S104 A) Transient absorption of **1β** in MeOH at room temperature. The sample was irradiated with a 420 nm light pulse, upon which the spectrum was recorded in steps of 1 ms increasing delay. B**)** EADS obtained by global analysis of the transient absorption data reported in **Figure S104a**. The exponential decay time extracted from global analysis is 9.1 ms. **C**) Kinetic traces recorded at the maximum of the bleaching and excited state absorption signal measured for **1β** in MeOH and fit from global analysis.

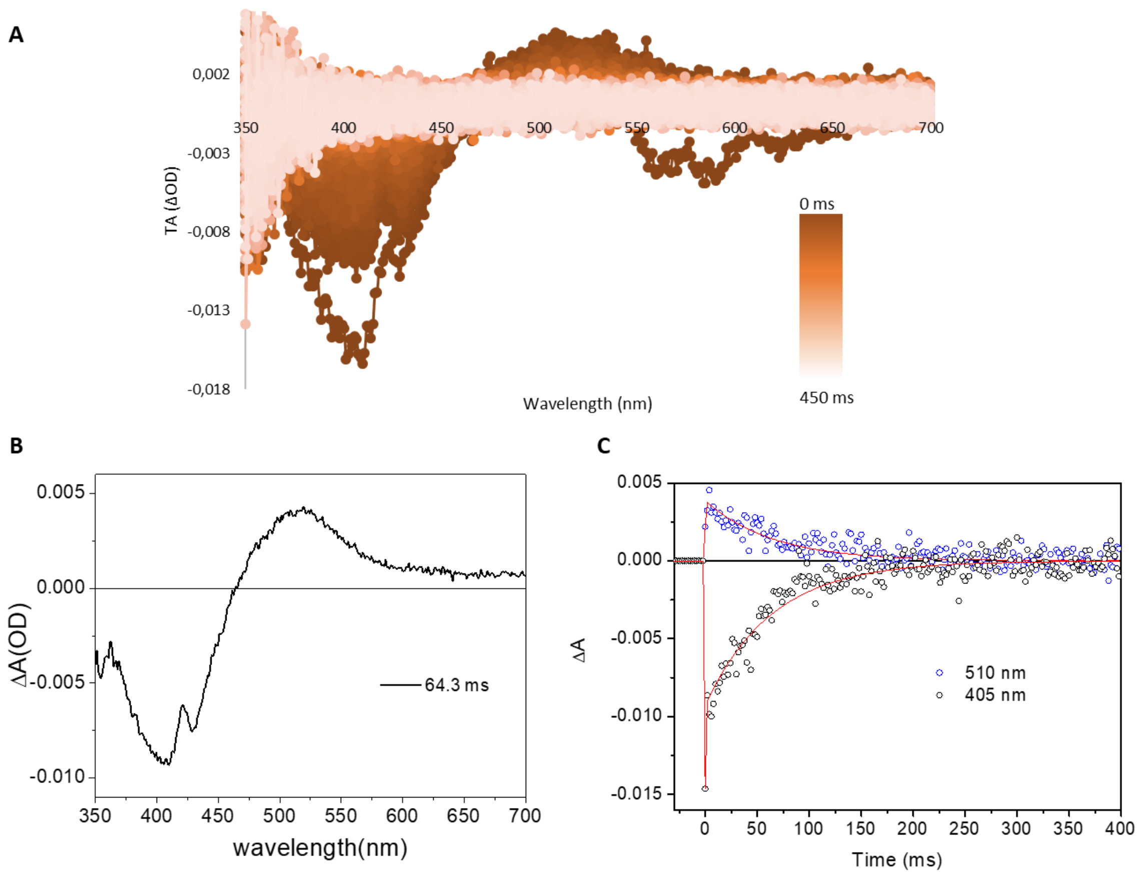

Figure S105 Transient absorption of **1γ** in MeOH at room temperature. The sample was irradiated with a 420 nm light pulse, upon which the spectrum was recorded in steps of 2 ms increasing delay. **B**) EADS obtained by global analysis of the transient absorption data reported in **Figure S105a**. The exponential decay time extracted from global analysis is 64.3 ms. **C**) Kinetic traces recorded at the maximum of the bleaching and excited state absorption signal measured for **1γ** in MeOH and fit from global analysis.

S4. Computational analysis

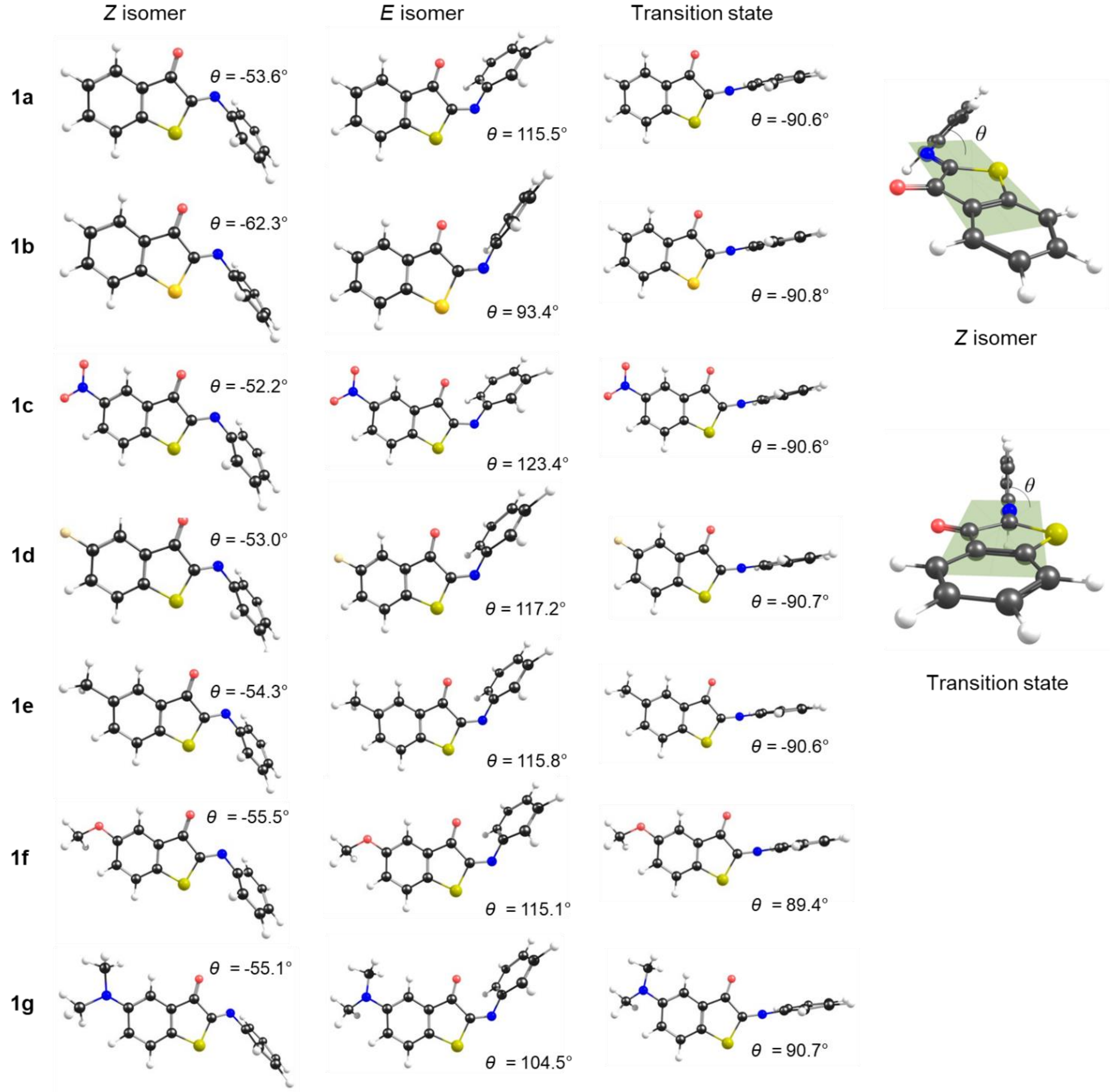

Figure S106 Structures of the *Z*-isomer, *E*-isomer and TS of ITI derivatives (**1a-1g**) in methanol optimized at the M06-2X/6-31+G(d)/SMD level of theory along with the definitions of dihedral angle ϑ in the *Z* isomer and TS.

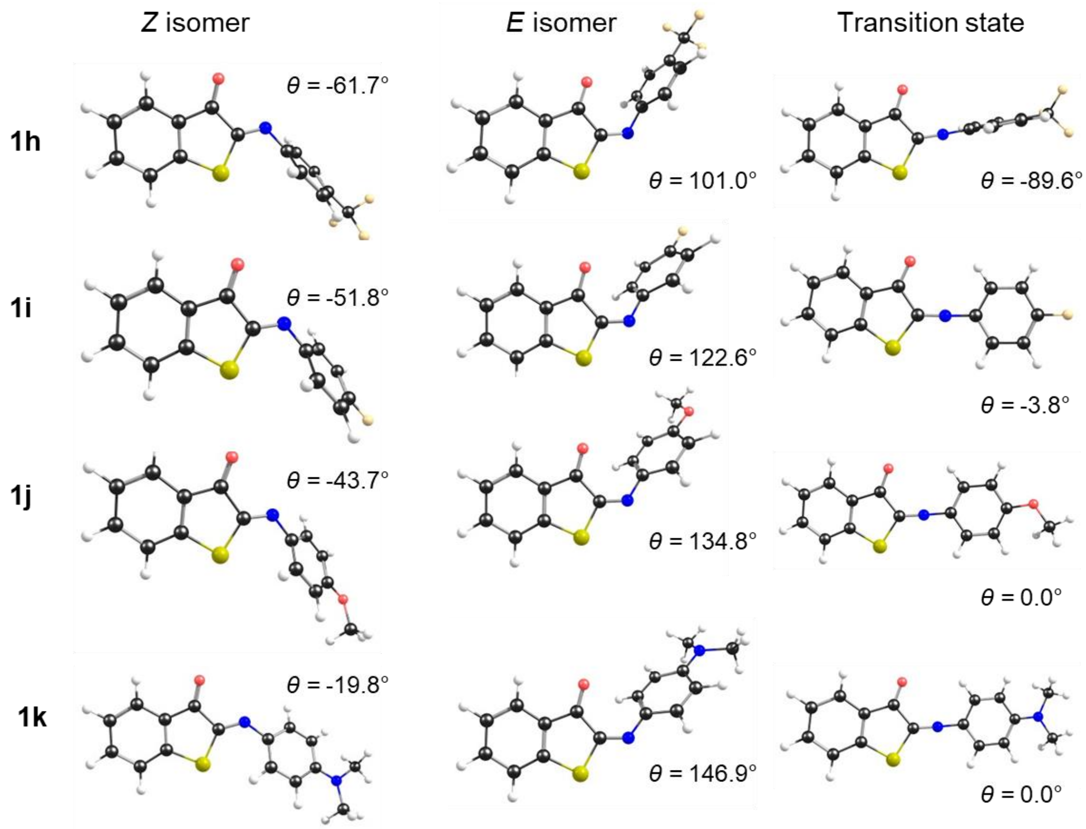

Figure S107 Structures of the *Z*-isomer, *E*-isomer and TS of ITI derivatives (**1h-1k**) in methanol optimized at the M06-2X/6-31+G(d)/SMD level of theory. See Figure S4.1 for definition of dihedral angle ϑ.

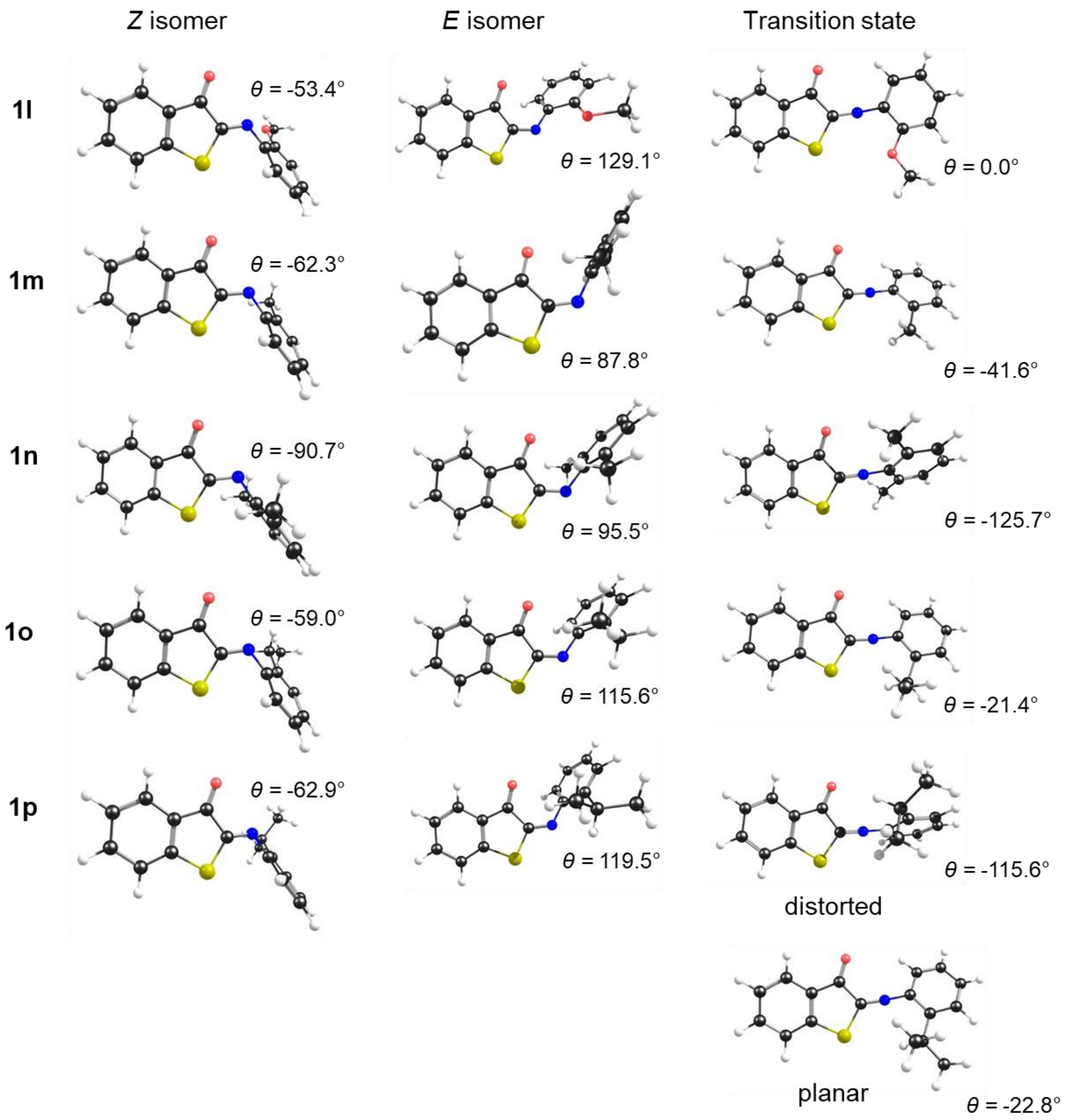

Figure S108 Structures of the *Z*-isomer, *E*-isomer and TS of ITI derivatives (**1l-1p**) in methanol optimized at the M06-2X/6-31+G(d)/SMD level of theory. See Figure S4.1 for definition of dihedral angle ϑ.

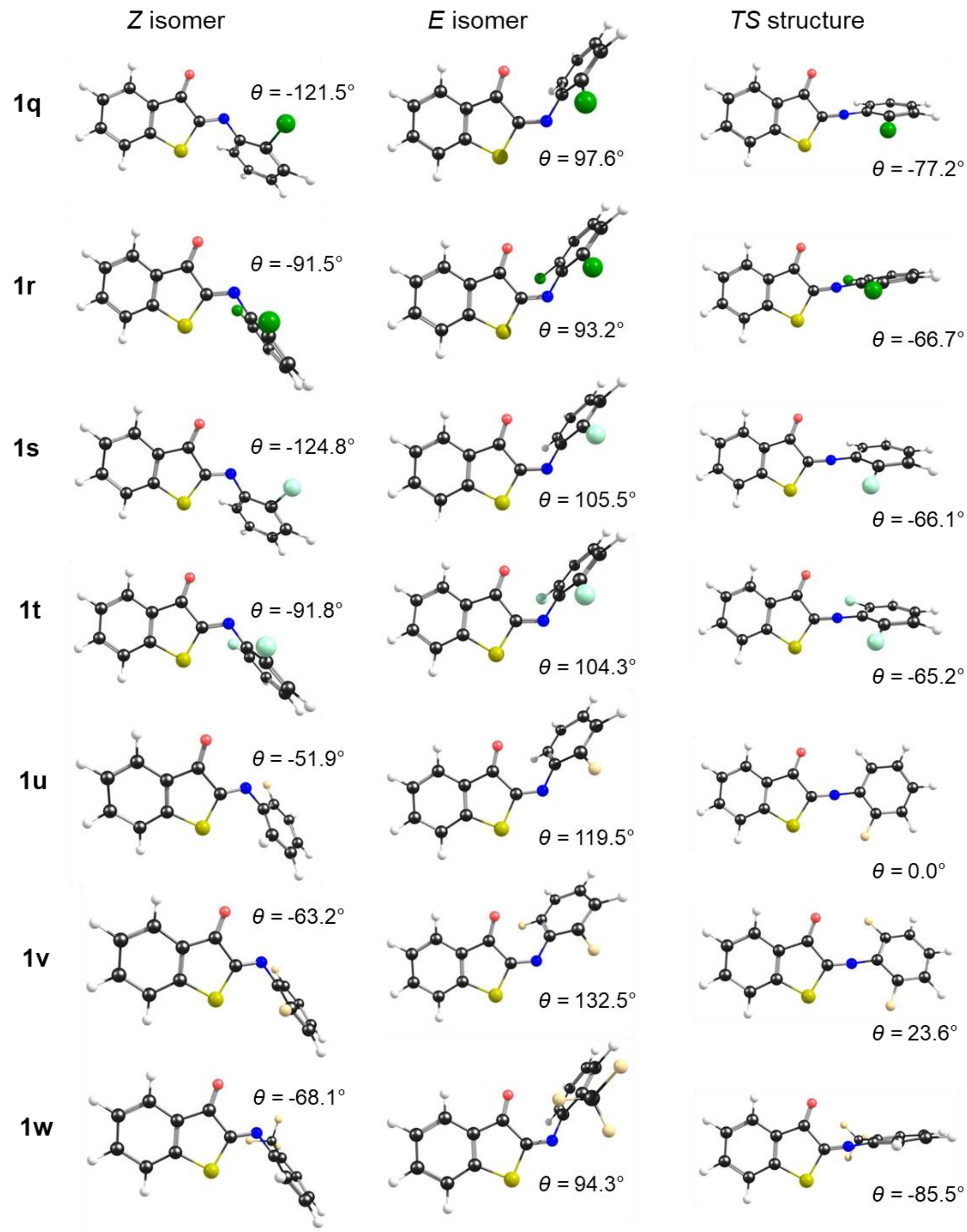

Figure S109 Structures of the *Z*-isomer, *E*-isomer and TS of ITI derivatives (**1q-1w**) in methanol optimized at the M06-2X/6-31+G(d)/SMD level of theory. For compounds **1q** and **1r**, the cc-pVTZ(-PP) basis set was used. See Figure S4.1 for definition of dihedral angle ϑ.

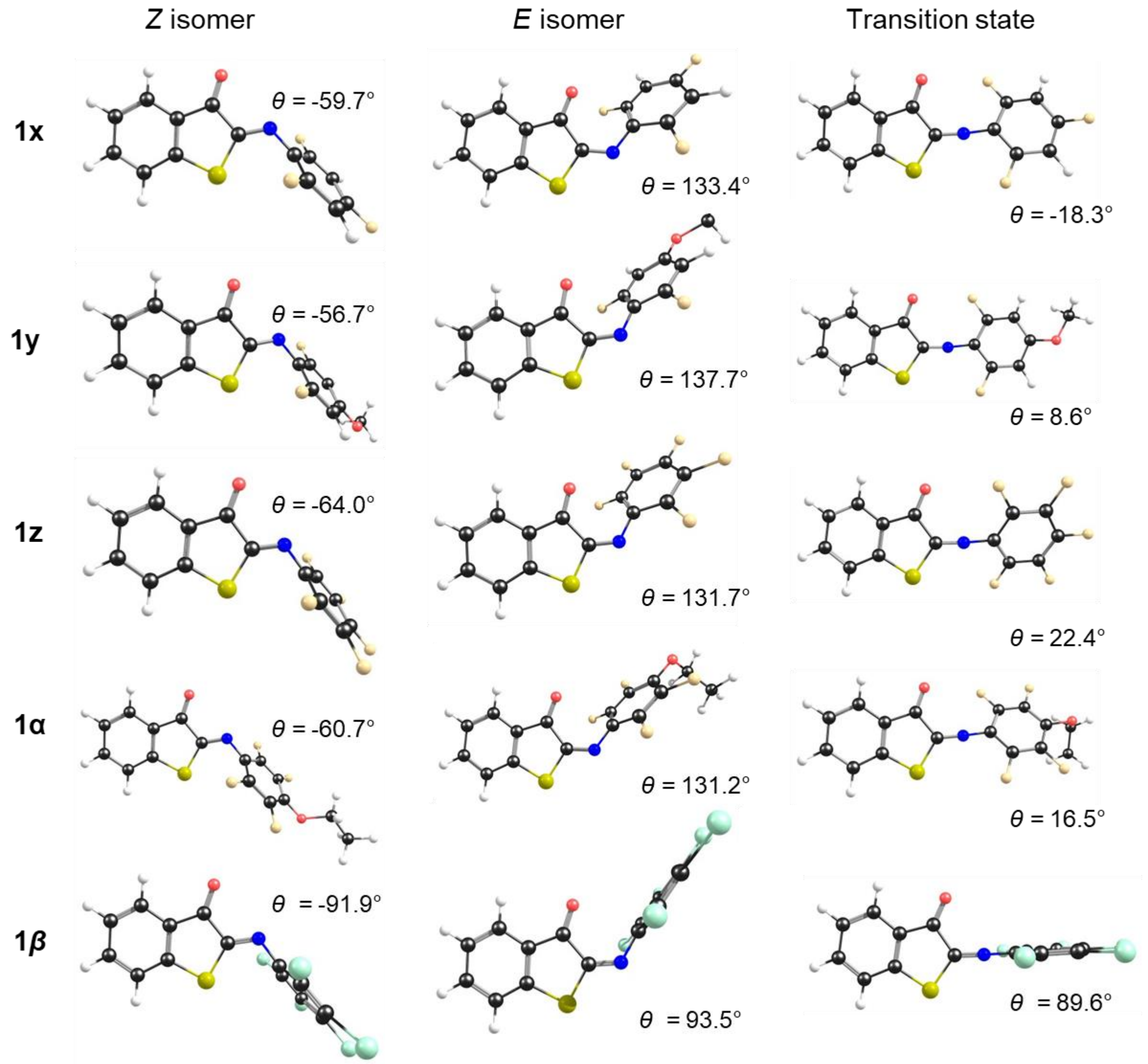

Figure S110 Structures of the *Z*-isomer, *E*-isomer and TS of ITI derivatives (**1x-1β**) in methanol optimized at the M06-2X/6-31+G(d)/SMD level of theory. See Figure S4.1 for definition of dihedral angle ϑ.

Table S1 Vertical excitation energies (VEEs) and oscillator strengths (*f*) for the first three excited states of *E/Z* isomers of **1a-1g** in methanol obtained at the TD-M06-2X/6-311++G(2df,2p)/SMD level, using the non-equilibrium cLR^2 approach.

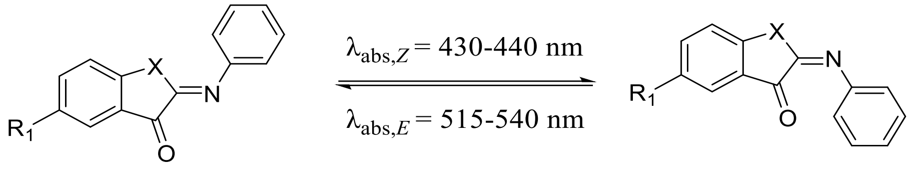

***Z*-1** ***E*-1**

1a: X = S, R_1 = H
1b: X = Se, R_1 = H
1c: X = S, R_1 = NO_2
1d: X = S, R_1 = F
1e: X = S, R_1 = Me
1f: X = S, R_1 = OMe
1g: X = S, R_1 = NMe_2

Cmpd.	R_1	Isomer	Transition	MO weight	Type	VEE (eV)	λ_{max} (nm)	*f*
**1a**	H	*Z*	$S_0 \rightarrow S_1$	58 → 63 (0.34)	(n,π*)	3.09	401	0.098
				62 → 63 (0.58)	(π,π*)			
			$S_0 \rightarrow S_2$	58 → 63 (0.44)	(n,π*)	3.24	383	0.022
				61 → 63 (0.37)	(π,π*)			
				62 → 63 (0.35)	(π,π*)			
			$S_0 \rightarrow S_3$	56 → 63 (-0.27)	mixed	3.61	343	0.054
				58 → 63 (-0.28)	(n,π*)			
				61 → 63 (0.51)	(π,π*)			
		E	$S_0 \rightarrow S_1$	62 → 63 (0.55)	(π,π*)	2.45	506	0.015
				58 → 63 (-0.30)	(n,π*)			
				61 → 63 (0.28)	(π,π*)			
			$S_0 \rightarrow S_2$	61 → 63 (0.60)	(π,π*)	3.11	399	0.023
				62 → 63 (0.35)	(π,π*)			
			$S_0 \rightarrow S_3$	56 → 63 (0.24)	(n,π*)	3.98	312	0.068
				58 → 63 (0.48)	(n,π*)			
**1b**	H	*Z*	$S_0 \rightarrow S_1$	71 → 72 (0.68)	(π,π*)	2.94	422	0.067
			$S_0 \rightarrow S_2$	67 → 72 (0.52)	(n,π*)	3.17	391	0.001
				70 → 72 (-0.39)	(π,π*)			
			$S_0 \rightarrow S_3$	65 → 72 (0.29)	Mixed	3.57	347	0.047
				67 → 72 (0.29)	(n,π*)			
				70 → 72 (0.50)	(π,π*)			
		E	$S_0 \rightarrow S_1$	67 → 72 (0.29)	(n,π*)	2.43	510	0.000
				70 → 72 (0.62)	Mixed			
			$S_0 \rightarrow S_2$	71 → 72 (0.70)	(π,π*)	2.91	426	0.017
			$S_0 \rightarrow S_3$	63 → 72 (0.30)	(n,π*)	4.02	308	0.001
				67 → 72 (0.49)	(n,π*)			
				70 → 72 (-0.25)	Mixed			
**1c**	NO_2	*Z*	$S_0 \rightarrow S_1$	69 → 74 (0.41)	(n,π*)	3.05	406	0.101
				73 → 74 (0.51)	(π,π*)			
			$S_0 \rightarrow S_2$	69 → 74 (0.35)	(n,π*)	3.29	377	0.110
				72 → 74 (0.35)	(π,π*)			
				73 → 74 (0.42)	(π,π*)			
			$S_0 \rightarrow S_3$	67 → 74 (-0.22)	(n,π*)	3.61	343	0.010
				69 → 74 (0.24)	(n,π*)			

				72 → 74 (0.53)	(π,π*)			
		E	$S_0 \rightarrow S_1$	69 → 74 (0.29)	(n,π*)	2.38	521	0.029
				73 → 74 (0.57)	(π,π*)			
			$S_0 \rightarrow S_2$	72 → 74 (0.63)	Mixed	3.23	384	0.066
				73 → 74 (-0.28)	(π,π*)			
			$S_0 \rightarrow S_3$	64 → 74 (-0.21)	(n,π*)	3.88	320	0.095
				69 → 74 (0.44)	(n,π*)			
				72 → 74 (-0.21)	Mixed			
				73 → 74 (-0.21)	(π,π*)			
**1d**	F	*Z*	$S_0 \rightarrow S_1$	62 → 67 (0.24)	(n,π*)	3.02	411	0.106
				66 → 67 (0.64)	(π,π*)			
			$S_0 \rightarrow S_2$	62 → 67 (0.48)	(n,π*)	3.20	387	0.005
				65 → 67 (-0.40)	(π,π*)			
				66 → 67 (-0.23)	(π,π*)			
			$S_0 \rightarrow S_3$	60 → 67 (-0.28)	(n,π*)	3.56	348	0.061
				62 → 67 (0.28)	(n,π*)			
				65 → 67 (0.50)	(π,π*)			
		E	$S_0 \rightarrow S_1$	62 → 67 (0.29)	(n,π*)	2.42	512	0.017
				65 → 67 (0.32)	(π,π*)			
				66 → 67 (0.52)	(π,π*)			
			$S_0 \rightarrow S_2$	65 → 67 (0.57)	(π,π*)	3.02	411	0.020
				66 → 67 (-0.40)	(π,π*)			
			$S_0 \rightarrow S_3$	59 → 67 (-0.22)	(n,π*)	3.93	315	0.073
				62 → 67 (0.47)	(n,π*)			
				65 → 67 (-0.21)	(π,π*)			
**1e**	CH_3	*Z*	$S_0 \rightarrow S_1$	66 → 67 (0.66)	(π,π*)	3.03	409	0.103
			$S_0 \rightarrow S_2$	62 → 67 (0.51)	(n,π*)	3.21	386	0.002
				65 → 67 (0.39)	(π,π*)			
			$S_0 \rightarrow S_3$	60 → 67 (0.28)	(n,π*)	3.59	345	0.071
				62 → 67 (-0.28)	(n,π*)			
				65 → 67 (0.50)	(π,π*)			
		E	$S_0 \rightarrow S_1$	62 → 67 (-0.27)	(n,π*)	2.46	504	0.015
				65 → 67 (0.41)	(π,π*)			
				66 → 67 (0.46)	(π,π*)			
			$S_0 \rightarrow S_2$	65 → 67 (-0.49)	(π,π*)	3.01	412	0.021
				66 → 67 (0.49)	(π,π*)			
			$S_0 \rightarrow S_3$	60 → 67 (0.22)	(n,π*)	3.98	312	0.078
				61 → 67 (-0.20)	Mixed			
				62 → 67 (0.45)	(n,π*)			
				65 → 67 (0.23)	(π,π*)			
**1f**	OMe	*Z*	$S_0 \rightarrow S_1$	70 → 71 (0.69)	(π,π*)	2.85	435	0.078
			$S_0 \rightarrow S_2$	65 → 71 (-0.32)	mixed	3.18	390	0.009
				66 → 71 (0.45)	mixed			
				69 → 71 (-0.39)	(π,π*)			
			$S_0 \rightarrow S_3$	64 → 71 (0.28)	(n,π*)	3.54	350	0.075

				65 → 71 (-0.26)	mixed			
				69 → 71 (0.50)	(π,π*)			
		E	$S_0 \rightarrow S_1$	65 → 71 (-0.26)	(n,π*)	2.44	508	0.074
				69 → 71 (0.54)	mixed			
				70 → 71 (0.30)	(π,π*)			
			$S_0 \rightarrow S_2$	69 → 71 (-0.29)	mixed	2.82	440	0.017
				70 → 71 (0.63)	(π,π*)			
			$S_0 \rightarrow S_3$	65 → 71 (0.42)	(n,π*)	3.96	313	0.015
				66 → 71 (-0.26)	(π,π*)			
				69 → 71 (0.28)	mixed			
**1g**	NMe_2	*Z*	$S_0 \rightarrow S_1$	74 → 75 (0.69)	(π,π*)	2.43	510	0.042
			$S_0 \rightarrow S_2$	69 → 75 (0.52)	(n,π*)	3.17	391	0.027
				73 → 75 (-0.39)	(π,π*)			
			$S_0 \rightarrow S_3$	67 → 75 (-0.25)	Mixed	3.54	350	0.116
				69 → 75 (0.34	(n,π*)			
				73 → 75 (0.48)	(π,π*)			
		E	$S_0 \rightarrow S_1$	69 → 75 (0.20)	(n,π*)	2.46	504	0.010
				73 → 75 (0.40)	(π,π*)			
				74 → 75 (0.53)	(π,π*)			
			$S_0 \rightarrow S_2$	69 → 75 (-0.22)	(n,π*)	2.53	490	0.009
				73 → 75 (-0.46)	(π,π*)			
				74 → 75 (0.45)	(π,π*)			
			$S_0 \rightarrow S_3$	67 → 75 (0.21)	Mixed	4.02	308	0.060
				69 → 75 (-0.43)	(n,π*)			
				71 → 75 (0.30)	(π,π*)			
				73 → 75 (0.26)	(π,π*)			

Table S2 Vertical excitation energies and band separation of the isomers for the $S_0 \rightarrow S_1$ transitions of *E/Z* isomers of **1a**-**1g** in methanol obtained at the TD-M06-2X/6-311++G(2df,2p)/SMD level, using the non-equilibrium cLR^2 approach.

Structure	Isomer	Transition	VEE (eV)	λ_{max} (nm)	$\Delta\lambda_{max}$ (nm)	*f*
**1a**	*Z*	$S_0 \rightarrow S_1$	3.09	401	105	0.098
	E	$S_0 \rightarrow S_1$	2.45	506		0.015
**1b**	*Z*	$S_0 \rightarrow S_1$	2.94	422	88	0.067
	E	$S_0 \rightarrow S_1$	2.43	510		0.000
**1c**	*Z*	$S_0 \rightarrow S_1$	3.05	406	115	0.101
	E	$S_0 \rightarrow S_1$	2.38	521		0.029
**1d**	*Z*	$S_0 \rightarrow S_1$	3.02	411	101	0.106
	E	$S_0 \rightarrow S_1$	2.42	512		0.017
**1e**	*Z*	$S_0 \rightarrow S_1$	3.03	409	105	0.103
	E	$S_0 \rightarrow S_1$	2.46	504		0.015
**1f**	*Z*	$S_0 \rightarrow S_1$	2.85	435	73	0.078
	E	$S_0 \rightarrow S_1$	2.44	508		0.074
**1g**	*Z*	$S_0 \rightarrow S_1$	2.43	510	6	0.042
	E	$S_0 \rightarrow S_1$	2.46	504		0.010

Table S3 Electronic and Gibbs reaction energies for $Z \rightarrow E$ transformation and activation barriers $E_{a,E\rightarrow Z}$ for back ($E \rightarrow Z$) isomerization (in kcal/mol, T = 298.15 K) of ITI derivatives **1a**-**1g** in methanol obtained at the M06-2X/6-31+G(d)//6-311++G(2df,2p)/SMD level of theory. Thermal corrections and entropic contributions were obtained with the smaller basis set.

Structure	ΔE				$\Delta G°$			
	6-31+G(d)		6-311++G(2df,2p)		6-31+G(d)		6-311++G(2df,2p)	
	$Z \rightarrow E$	$E_{a,E\rightarrow Z}$	$Z \rightarrow E$	$E_{a,E\rightarrow Z}$	$Z \rightarrow E$	$E_{a,E\rightarrow Z}$	$Z \rightarrow E$	$E_{a,E\rightarrow Z}$
**1a**	4.3	14.5	4.0	14.1	3.6	13.6	3.3	13.3
**1b**	5.4	14.7	4.9	13.0	4.8	14.6	4.3	12.8
**1c**	4.1	14.4	3.9	13.8	3.6	13.2	3.5	12.7
**1d**	4.2	14.4	4.0	14.0	3.7	13.5	3.5	13.1
**1e**	4.3	14.3	4.0	13.9	3.9	14.0	3.7	13.6
**1f**	4.3	14.5	4.1	14.0	3.7	13.3	3.4	14.0
**1g**	4.3	14.5	4.0	14.2	3.8	13.9	3.5	13.6

Table S4 Orbital character**,** vertical excitation energies (VEEs) and oscillator strengths (*f*) for the first three excited states of *E*/*Z* isomers of **1h-1k** obtained at the M06-2X/6-311++G(2df,2p)/SMD level, using the cLR^2 approach.

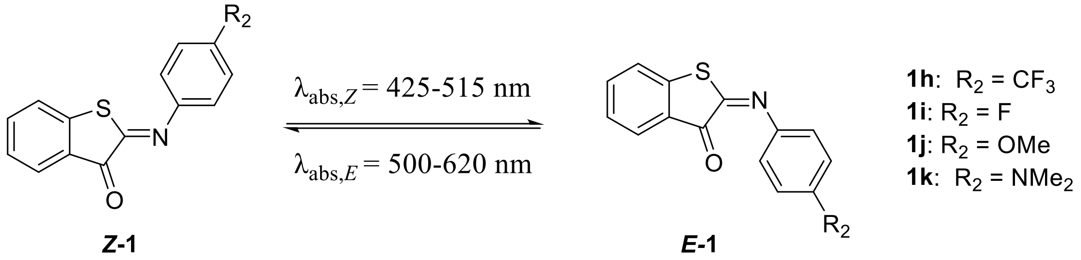

Cmpd.	-R$_2$	Isomer	Transition	MOs weight	Type	VEE (eV)	λ_{max} (nm)	*f*
**1h**	CF$_3$	*Z*	$S_0 \rightarrow S_1$	74 → 79 (0.36)	(n,π*)	3.15	394	0.066
				78 → 79 (0.55)	(π,π*)			
			$S_0 \rightarrow S_2$	74 → 79 (-0.43)	(n,π*)	3.25	381	0.022
				77 → 79 (0.31)	(π,π*)			
				78 → 79 (0.42)	(π,π*)			
			$S_0 \rightarrow S_3$	72 → 79 (-0.27)	(n,π*)	3.68	337	0.047
				74 → 79 (0.27)	(n,π*)			
				77 → 79 (0.52)	(π,π*)			
		E	$S_0 \rightarrow S_1$	74 → 79 (-0.29)	(n,π*)	2.57	482	0.002
				77 → 79 (0.54)	(π,π*)			
				78 → 79 (-0.29)	(π,π*)			
			$S_0 \rightarrow S_2$	77 → 79 (0.32)	(π,π*)	3.13	396	0.021
				78 → 79 (0.62)	(π,π*)			
			$S_0 \rightarrow S_3$	70 → 79 (0.25)	Mixed	4.14	299	0.013
				72 → 79 (-0.23)	(n,π*)			
				74 → 79 (0.48)	(n,π*)			
				77 → 79 (0.23)	(π,π*)			
**1i**	F	*Z*	$S_0 \rightarrow S_1$	62 → 67 (-0.33)	(n,π*)	3.08	403	0.110
				66 → 67 (0.60)	(π,π*)			
			$S_0 \rightarrow S_2$	62 → 67 (0.45)	(n,π*)	3.25	381	0.020
				65 → 67 (0.38)	(π,π*)			
				66 → 67 (0.32)	(π,π*)			
			$S_0 \rightarrow S_3$	60 → 67 (0.28)	(n,π*)	3.60	344	0.059
				62 → 67 (-0.27)	(n,π*)			
				65 → 67 (0.52)	(π,π*)			
		E	$S_0 \rightarrow S_1$	62 → 67 (0.30)	(n,π*)	2.43	510	0.026
				65 → 67 (-0.26)	(π,π*)			
				66 → 67 (0.55)	(π,π*)			
			$S_0 \rightarrow S_2$	65 → 67 (0.60)	(π,π*)	3.10	400	0.024
				66 → 67 (0.35)	(π,π*)			
			$S_0 \rightarrow S_3$	58 → 67 (-0.21)	Mixed	3.95	314	0.108
				60 → 67 (-0.23)	(n,π*)			
				62 → 67 (0.48)	(n,π*)			

				65 → 67 (0.22)	(π,π*)			
**1j**	OMe	*Z*	$S_0 \rightarrow S_1$	66 → 71 (-0.28)	(n,π*)	2.93	423	0.232
				70 → 71 (0.62)	(π,π*)			
			$S_0 \rightarrow S_2$	66 → 71 (0.48)	(n,π*)	3.25	381	0.020
				69 → 71 (-0.43)	(π,π*)			
			$S_0 \rightarrow S_3$	64 → 71 (-0.30)	(n,π*)	3.52	352	0.067
				66 → 71 (0.28)	(n,π*)			
				69 → 71 (0.52)	(π,π*)			
		E	$S_0 \rightarrow S_1$	66 → 71 (0.33)	(n,π*)	2.29	541	0.074
				70 → 71 (0.59)	(π,π*)			
			$S_0 \rightarrow S_2$	69 → 71 (0.63)	(π,π*)	3.07	404	0.031
				70 → 71 (0.24)	(π,π*)			
			$S_0 \rightarrow S_3$	64 → 71 (0.24)	(n,π*)	3.71	334	0.204
				66 → 71 (0.47)	(n,π*)			
				69 → 71 (0.27)	(π,π*)			
				70 → 71 (-0.25)	(π,π*)			
**1k**	NMe_2	*Z*	$S_0 \rightarrow S_1$	74 → 75 (0.67)	(π,π*)	2.62	473	0.747
			$S_0 \rightarrow S_2$	70 → 75 (0.36)	(n,π*)	3.25	381	0.017
				71 → 75 (0.45)	Mixed			
				73 → 75 (0.26)	(π,π*)			
			$S_0 \rightarrow S_3$	73 → 75 (0.62)	(π,π*)	3.45	359	0.074
		E	$S_0 \rightarrow S_1$	71 → 75 (0.25)	(π,π*)	2.09	593	0.238
				74 → 75 (0.61)	(π,π*)			
			$S_0 \rightarrow S_2$	71 → 75 (0.30)	(π,π*)	3.06	405	0.081
				72 → 75 (-0.24)	(π,π*)			
				73 → 75 (0.51)	(π,π*)			
				74 → 75 (-0.21)	(π,π*)			
			$S_0 \rightarrow S_3$	69 → 75 (-0.22)	Mixed	3.37	368	0.250
				71 → 75 (-0.22)	(π,π*)			
				72 → 75 (0.26)	(π,π*)			
				73 → 75 (0.47)	(π,π*)			
				74 → 75 (0.24)	(π,π*)			

Table S5 Vertical excitation energies and band separation of the isomers for the $S_0 \rightarrow S_1$ transition of *E*/*Z* isomers of **1a**,**1h-1k** obtained at the M06-2X/6-311++G(2df,2p)/SMD level, using the cLR^2 approach.

Structure	Isomer	Transition	VEE (eV)	λ_{max} (nm)	$\Delta\lambda_{max}$ (nm)	*f*
**1a**	*Z*	$S_0 \rightarrow S_1$	3.09	401	105	0.098
	E	$S_0 \rightarrow S_1$	2.45	506		0.015
**1h**	*Z*	$S_0 \rightarrow S_1$	3.15	394	88	0.066
	E	$S_0 \rightarrow S_1$	2.57	482		0.002
**1i**	*Z*	$S_0 \rightarrow S_1$	3.08	403	107	0.110
	E	$S_0 \rightarrow S_1$	2.43	510		0.026
**1j**	*Z*	$S_0 \rightarrow S_1$	2.93	423	118	0.232
	E	$S_0 \rightarrow S_1$	2.29	541		0.074
**1k**	*Z*	$S_0 \rightarrow S_1$	2.62	473	120	0.748
	E	$S_0 \rightarrow S_1$	2.09	593		0.238

Table S6 Electronic and Gibbs reaction energies for $Z \rightarrow E$ transformation and activation barriers $E_{a,E\rightarrow Z}$ for back ($E \rightarrow Z$) isomerization (in kcal/mol, T = 298.15 K) of ITI derivatives **1a**,**1h-1k** in methanol obtained at the M06-2X/6-31+G(d)//6-311++G(2df,2p)/SMD level of theory. Thermal corrections and entropic contributions were obtained with the smaller basis set.

Structure	ΔE				$\Delta G°$			
	6-31+G(d)		6-311++G(2df,2p)		6-31+G(d)		6-311++G(2df,2p)	
	$Z \rightarrow E$	$E_{a,E\rightarrow Z}$	$Z \rightarrow E$	$E_{a,E\rightarrow Z}$	$Z \rightarrow E$	$E_{a,E\rightarrow Z}$	$Z \rightarrow E$	$E_{a,E\rightarrow Z}$
**1a**	4.3	14.5	4.0	14.1	3.6	13.6	3.3	13.3
**1h**	3.5	13.6	3.3	13.3	2.7	13.3	2.5	13.1
**1i**	4.2	14.4	4.1	14.7	4.1	13.5	4.0	13.8
**1j**	4.8	12.9	4.7	13.2	4.4	12.9	4.3	13.2
**1k**	5.3	11.0	5.4	11.2	5.3	10.1	5.4	10.3

Table S7 Vertical excitation energies (VEEs) and oscillator strengths (*f*) for the first three excited states of *E*/*Z* isomers of **1l-1p** in methanol obtained at the TD-M06-2X/6-311++G(2df,2p)/SMD level, using the non-equilibrium cLR^2 approach.

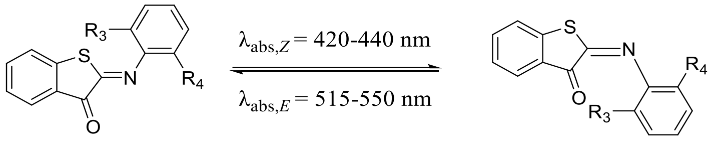

***Z*-1** ***E*-1**

1l: R_3=OMe R_4=H
1m: R_3=Me R_4=H
1n: R_3=Me R_4=Me
1o: R_3=Et R_4=H
1p: R_3=*i*Pr R_4=H

Cmpd.	-R_3	-R_4	Isomer	Transition	MOs weight	Type	VEE (eV)	λ_{max} (nm)	*f*
**1l**	OMe	H	*Z*	$S_0 \rightarrow S_1$	66 → 71 (-0.27)	(n,π*)	2.96	419	0.139
					70 → 71 (0.60)	(π,π*)			
				$S_0 \rightarrow S_2$	66 → 71 (0.44)	(n,π*)	3.25	381	0.022
					68 → 71 (-0.24)	(π,π*)			
					69 → 71 (0.42)	(π,π*)			
				$S_0 \rightarrow S_3$	64 → 71 (0.21)	Mixed	3.47	357	0.039
					66 → 71 (-0.34)	(n,π*)			
					69 → 71 (0.46)	(π,π*)			
			E	$S_0 \rightarrow S_1$	66 → 71 (0.26)	(n,π*)	2.36	525	0.046
					68 → 71 (-0.28)	(π,π*)			
					70 → 71 (0.56)	(π,π*)			
				$S_0 \rightarrow S_2$	68 → 71 (-0.27)	Mixed	3.08	403	0.030
					69 → 71 (0.58)	(π,π*)			
					70 → 71 (-0.27)	(π,π*)			
				$S_0 \rightarrow S_3$	66 → 71 (-0.31)	(n,π*)	3.49	355	0.097
					68 → 71 (0.33)	Mixed			
					69 → 71 (0.37)	(π,π*)			
					70 → 71 (0.30)	(π,π*)			
**1m**	Me	H	*Z*	$S_0 \rightarrow S_1$	62 → 67 (-0.35)	(n,π*)	3.06	405	0.075
					66 → 67 (0.58)	(π,π*)			
				$S_0 \rightarrow S_2$	62 → 67 (0.41)	(n,π*)	3.25	381	0.028
					65 → 67 (-0.40)	(π,π*)			
					66 → 67 (0.34)	(π,π*)			
				$S_0 \rightarrow S_3$	60 → 67 (-0.25)	(n,π*)	3.52	352	0.026
					62 → 67 (0.33)	(n,π*)			
					65 → 67 (0.48)	(π,π*)			
			E	$S_0 \rightarrow S_1$	62 → 67 (0.29)	(n,π*)	2.38	521	0.001
					66 → 67 (0.61)	(π,π*)			
				$S_0 \rightarrow S_2$	65 → 67 (0.70)	(π,π*)	3.13	396	0.021
				$S_0 \rightarrow S_3$	60 → 67 (-0.24)	(n,π*)	3.90	318	0.001
					62 → 67 (0.50)	(n,π*)			
					66 → 67 (-0.27)	(π,π*)			
**1n**	Me	Me	*Z*	$S_0 \rightarrow S_1$	66 → 71 (-0.42)	(n,π*)	3.04	408	0.000
					70 → 71 (0.53)	(π,π*)			
				$S_0 \rightarrow S_2$	69 → 71 (0.70)	(π,π*)	3.23	384	0.048

				$S_0 \rightarrow S_3$	64 → 71 (-0.27)	(n,π*)	3.38	367	0.000
					66 → 71 (0.48)	(n,π*)			
					70 → 71 (0.38)	(π,π*)			
			E	$S_0 \rightarrow S_1$	66 → 71 (-0.28)	(n,π*)	2.33	532	0.001
					70 → 71 (0.62)	(π,π*)			
				$S_0 \rightarrow S_2$	69 → 71 (0.70)	(π,π*)	3.13	396	0.020
				$S_0 \rightarrow S_3$	64 → 71 (-0.24)	(n,π*)	3.88	320	0.001
					66 → 71 (0.53)	(n,π*)			
					70 → 71 (0.26)	(π,π*)			
**1o**	Et	H	*Z*	$S_0 \rightarrow S_1$	70 → 75 (0.26)	(n,π*)	2.95	420	0.141
					74 → 75 (0.60)	(π,π*)			
				$S_0 \rightarrow S_2$	70 → 75 (0.43)	(n,π*)	3.24	383	0.023
					72 → 75 (-0.25)	(π,π*)			
					73 → 75 (0.42)	(n,π*)			
				$S_0 \rightarrow S_3$	70 → 75 (-0.35)	(n,π*)	3.46	358	0.036
					73 → 75 (0.46)	(n,π*)			
					74 → 75 (0.20)	(π,π*)			
			E	$S_0 \rightarrow S_1$	70 → 75 (-0.26)	(n,π*)	2.35	528	0.034
					72 → 75 (-0.27)	(π,π*)			
					74 → 75 (0.57)	(π,π*)			
				$S_0 \rightarrow S_2$	72 → 75 (0.24)	(π,π*)	3.11	399	0.026
					73 → 75 (0.61)	(n,π*)			
					74 → 75 (0.22)	(π,π*)			
				$S_0 \rightarrow S_3$	70 → 75 (0.33)	(n,π*)	3.50	354	0.072
					72 → 75 (0.35)	(π,π*)			
					73 → 75 (-0.32)	(n,π*)			
					74 → 75 (0.31)	(π,π*)			
**1p**	*i*Pr	H	*Z*	$S_0 \rightarrow S_1$	70 → 75 (0.36)	(n,π*)	3.04	408	0.063
					74 → 75 (0.56)	(π,π*)			
				$S_0 \rightarrow S_2$	70 → 75 (-0.36)	(n,π*)	3.24	383	0.034
					73 → 75 (0.42)	(π,π*)			
				$S_0 \rightarrow S_3$	70 → 75 (0.35)	(n,π*)	3.51	353	0.019
					73 → 75 (0.46)	(π,π*)			
			E	$S_0 \rightarrow S_1$	70 → 75 (-0.28)	(n,π*)	2.41	514	0.020
					73 → 75 (-0.22)	Mixed			
					74 → 75 (0.57)	(π,π*)			
				$S_0 \rightarrow S_2$	73 → 75 (0.62)	Mixed	3.11	399	0.024
					74 → 75 (0.30)	(π,π*)			
				$S_0 \rightarrow S_3$	70 → 75 (0.41)	(n,π*)	3.85	322	0.081
					72 → 75 (0.30)	(π,π*)			
					73 → 75 (-0.21)	Mixed			
					74 → 75 (0.23)	(π,π*)			

Table S8 Vertical excitation energies and band separation for the $S_0 \rightarrow S_1$ transition of the *Z*/*E* isomers of **1a**, **1l-1p** in methanol obtained at the TD-M06-2X/6-311++G(2df,2p)/SMD level, using the non-equilibrium cLR^2 approach in methanol.

Structure	Isomer	Transition	VEE (eV)	λ_{max} (nm)	$\Delta\lambda_{max}$ (nm)	*f*
**1a**	*Z*	$S_0 \rightarrow S_1$	3.09	401	105	0.098
	E	$S_0 \rightarrow S_1$	2.45	506		0.015
**1l**	*Z*	$S_0 \rightarrow S_1$	2.96	419	106	0.140
	E	$S_0 \rightarrow S_1$	2.36	525		0.046
**1m**	*Z*	$S_0 \rightarrow S_1$	3.06	405	116	0.075
	E	$S_0 \rightarrow S_1$	2.38	521		0.009
**1n**	*Z*	$S_0 \rightarrow S_1$	3.04	408	124	0.000
	E	$S_0 \rightarrow S_1$	2.33	532		0.001
**1o**	*Z*	$S_0 \rightarrow S_1$	2.95	420	108	0.141
	E	$S_0 \rightarrow S_1$	2.35	528		0.034
**1p**	*Z*	$S_0 \rightarrow S_1$	3.04	408	106	0.063
	E	$S_0 \rightarrow S_1$	2.41	514		0.020

Table S4.9 Electronic and Gibbs reaction energies for $Z \rightarrow E$ transformation and activation barriers $E_{a,E\rightarrow Z}$ for back ($E \rightarrow Z$) isomerization (in kcal/mol, T = 298.15 K) of ITI derivatives **1a**, **1l-1p** in methanol obtained at the M06-2X/6-31+G(d)//6-311++G(2df,2p)/SMD level of theory. Thermal corrections and entropic contributions were obtained with the smaller basis set.

Structure	ΔE				$\Delta G°$			
	6-31+G(d)		6-311++G(2df,2p)		6-31+G(d)		6-311++G(2df,2p)	
	$Z \rightarrow E$	$E_{a,E\rightarrow Z}$	$Z \rightarrow E$	$E_{a,E\rightarrow Z}$	$Z \rightarrow E$	$E_{a,E\rightarrow Z}$	$Z \rightarrow E$	$E_{a,E\rightarrow Z}$
**1a**	4.3	14.5	4.0	14.1	3.6	13.6	3.3	13.3
**1l**	4.2	12.9	4.1	13.2	3.8	13.0	3.6	13.3
**1m**	4.0	14.2	3.6	14.3	4.1	13.1	3.7	13.2
**1n**	3.3	13.3	3.1	13.3	2.9	13.3	2.7	13.4
**1o**	3.0	14.3	2.9	14.5	3.2	13.7	3.0	13.8
**1p**	3.0	14.3 (14.2)^a	2.8	13.9	3.0	13.9 (14.4)^a	2.8	13.4

^{a} The activation barriers obtained for the planar structure (see Figure S4.3)

Table S10 Vertical excitation energies (VEEs) and oscillator strengths (*f*) for the first three excited states of *E*/*Z* isomers of **1q-1w** in methanol obtained at the TD-M06-2X/6-311++G(2df,2p)/SMD and TD-M06-2X/cc-pVTZ-PP/SMD (marked with *) levels, using the non-equilibrium cLR^2 approach.

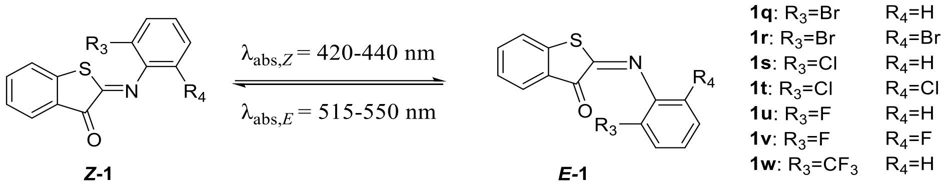

Cmpd.	-R_3	-R_4	Isomer	Transition	MOs weight	Type	VEE (eV)	λ_{max} (nm)	*f*
**1q***	Br	H	*Z*	$S_0 \rightarrow S_1$	69 → 75 (0.23)	Mixed	3.18	390	0.048
					70 → 75 (-0.37)	(n,π*)			
					74 → 75 (0.48)	(π,π*)			
				$S_0 \rightarrow S_2$	69 → 75 (-0.21)	Mixed	3.32	373	0.044
					70 → 75 (0.28)	(n,π*)			
					73 → 75 (0.31)	(π,π*)			
					74 → 75 (0.48)	(π,π*)			
				$S_0 \rightarrow S_3$	66 → 75 (-0.23)	(n,π*)	3.64	341	0.029
					69 → 75 (0.21)	Mixed			
					70 → 75 (-0.24)	(n,π*)			
					72 →75 (-0.20)	Mixed			
					73 → 75 (0.48)	(π,π*)			
			E	$S_0 \rightarrow S_1$	69 → 75 (0.27)	(n,π*)	2.52	492	0.001
					74 → 75 (0.59)	(π,π*)			
				$S_0 \rightarrow S_2$	73 → 75 (0.69)	(π,π*)	3.21	386	0.022
				$S_0 \rightarrow S_3$	69 → 75 (0.40)	(n,π*)	4.48	277	0.004
					72 → 75 (-0.32)	Mixed			
					74 → 75 (-0.31)	(π,π*)			
**1r***	Br	Br	*Z*	$S_0 \rightarrow S_1$	82 → 87 (0.53)	(n,π*)	3.22	385	0.000
					85 → 87 (-0.37)	(π,π*)			
				$S_0 \rightarrow S_2$	86 → 87 (0.70)	(π,π*)	3.30	376	0.046
				$S_0 \rightarrow S_3$	77 → 87 (0.21)	(π,π*)	3.55	349	0.000
					82 → 87 (0.28)	(n,π*)			
					85 → 87 (0.53)	(π,π*)			
			E	$S_0 \rightarrow S_1$	81 → 87 (-0.25)	Mixed	2.54	488	0.000
					86 → 87 (0.61)	(π,π*)			
				$S_0 \rightarrow S_2$	85 → 87 (0.70)	(π,π*)	3.19	389	0.021
				$S_0 \rightarrow S_3$	84 → 87 (0.70)	Mixed	4.09	303	0.003
**1s**	Cl	*H*	*Z*	$S_0 \rightarrow S_1$	66 → 71 (0.34)	(n,π*)	3.13	396	0.076
					70 → 71 (0.58)	(π,π*)			
				$S_0 \rightarrow S_2$	66 → 71 (0.44)	(n,π*)	3.26	380	0.021
					69 →71 (0.32)	(π,π*)			
					70 → 71 (-0.36)	(π,π*)			

				$S_0 \rightarrow S_3$	63 → 71 (-0.22)	(n,π*)	3.59	345	0.042
					66 → 71 (-0.28)	(n,π*)			
					69 → 71 (0.50)	(π,π*)			
			E	$S_0 \rightarrow S_1$	66 → 71 (0.29)	(n,π*)	2.50	496	0.004
					69 → 71 (0.24)	(π,π*)			
					70 → 71 (0.55)	(π,π*)			
				$S_0 \rightarrow S_2$	69 → 71 (0.63)	(π,π*)	3.10	400	0.020
					70 → 71 (-0.31)	(π,π*)			
				$S_0 \rightarrow S_3$	66 → 71 (0.45)	(n,π*)	3.95	314	0.027
					68 → 71 (0.25)	(π,π*)			
					70 → 71 (-0.25)	(π,π*)			
**1t**	Cl	Cl	*Z*	$S_0 \rightarrow S_1$	66 → 71 (0.34)	(n,π*)	3.35	370	0.000
					70 → 71 (0.58)	(π,π*)			
				$S_0 \rightarrow S_2$	66 → 71 (0.44)	(n,π*)	3.44	360	0.044
					69 → 71 (0.32)	(π,π*)			
					70 → 71 (-0.36)	(π,π*)			
				$S_0 \rightarrow S_3$	63 → 71 (-0.22)	Mixed	3.65	340	0.000
					66 → 71 (-0.28)	(n,π*)			
					69 →71 (0.50)	(π,π*)			
			E	$S_0 \rightarrow S_1$	74 → 79 (0.29)	(n,π*)	2.51	494	0.003
					77 → 79 (-0.33)	(π,π*)			
					78 →79 (0.52)	(π,π*)			
				$S_0 \rightarrow S_2$	77 → 79 (0.58)	(π,π*)	3.11	399	0.019
					78 → 79 (0.39)	(π,π*)			
				$S_0 \rightarrow S_3$	68 → 79 (-0.23)	(n,π*)	4.02	308	0.017
					74 → 79 (0.51)	(n,π*)			
					78 → 79 (-0.21)	(π,π*)			
**1u**	F	H	*Z*	$S_0 \rightarrow S_1$	62 → 67(-0.32)	(n,π*)	3.09	401	0.108
					66 → 67 (0.60)	(π,π*)			
				$S_0 \rightarrow S_2$	62 → 67 (0.47)	(n,π*)	3.24	383	0.016
					65 → 67 (0.34)	(π,π*)			
					66 → 67 (0.32)	(π,π*)			
				$S_0 \rightarrow S_3$	60 → 67 (0.26)	(n,π*)	3.62	342	0.073
					62 → 67 (-0.26)	(n,π*)			
					65 → 67 (0.52)	(π,π*)			
			E	$S_0 \rightarrow S_1$	62 → 67 (-0.29)	(n,π*)	2.48	500	0.026
					65 → 67 (-0.32)	(π,π*)			
					66 → 67 (0.51)	(π,π*)			
				$S_0 \rightarrow S_2$	65 → 67 (0.55)	(π,π*)	3.08	403	0.024
					66 → 67 (0.42)	(π,π*)			
				$S_0 \rightarrow S_3$	60 → 67 (-0.20)	(n,π*)	3.92	316	0.116
					62 → 67 (0.42)	(n,π*)			
					64 → 67 (-0.28)	(π,π*)			
					65 → 67 (-0.26)	(π,π*)			
**1v**	F	F	*Z*	$S_0 \rightarrow S_1$	66 → 71 (0.40)	(n,π*)	3.15	394	0.062

					70 → 71 (0.53)	(π,π*)			
				$S_0 \rightarrow S_2$	66 → 71 (-0.40)	(n,π*)	3.23	384	0.029
					69 → 71 (0.33)	(π,π*)			
					70 → 71 (0.45)	(π,π*)			
				$S_0 \rightarrow S_3$	64 → 71 (0.24)	(n,π*)	3.55	349	0.059
					66 → 71 (0.30)	(n,π*)			
					69 → 71 (0.53)	(π,π*)			
			E	$S_0 \rightarrow S_1$	66 → 71 (-0.29)	(n,π*)	2.52	492	0.042
					69 → 71 (0.37)	(π,π*)			
					70 → 71 (0.49)	(π,π*)			
				$S_0 \rightarrow S_2$	69 → 71 (0.51)	(π,π*)	3.06	405	0.025
					70 → 71 (-0.47)	(π,π*)			
				$S_0 \rightarrow S_3$	63 → 71 (-0.21)	Mixed	3.99	311	0.192
					66 → 71 (0.45)	(n,π*)			
					69 → 71 (0.26)	(π,π*)			
**1w**	CF_3	H	*Z*	$S_0 \rightarrow S_1$	74 → 79 (-0.40)	(n,π*)	3.17	391	0.048
					78 → 79 (0.52)	(π,π*)			
				$S_0 \rightarrow S_2$	74 → 79 (0.41)	(n,π*)	3.24	383	0.026
					77 → 79 (0.29)	(π,π*)			
					78 → 79 (0.46)	(π,π*)			
				$S_0 \rightarrow S_3$	72 → 79 (-0.26)	(n,π*)	3.62	342	0.027
					74 → 79 (-0.27)	(n,π*)			
					77 → 79 (0.54)	(π,π*)			
			E	$S_0 \rightarrow S_1$	74 → 79 (-0.31)	(n,π*)	2.55	486	0.000
					77 → 79 (0.56)	(π,π*)			
					78 → 79 (0.24)	(π,π*)			
				$S_0 \rightarrow S_2$	77 → 79 (-0.27)	(π,π*)	3.12	397	0.020
					78 → 79 (0.65)	(π,π*)			
				$S_0 \rightarrow S_3$	70 → 79 (-0.21)	(n,π*)	4.09	303	0.000
					72 → 79 (-0.24)	(n,π*)			
					74 → 79 (0.49)	(n,π*)			
					77 → 79 (0.25)	(π,π*)			

Table S11 Vertical excitation energies and band separation for the $S_0 \rightarrow S_1$ transition of *E*/*Z* isomers of **1q-1w** in methanol obtained at the TD-M06-2X/6-311++G(2df,2p)/SMD and TD-M06-2X/cc-pVTZ-PP/SMD (marked with *) levels, using the non-equilibrium cLR^2 approach.

Structure	Isomer	Transition	VEE (eV)	λ_{max} (nm)	$\Delta\lambda_{max}$ (nm)	*f*
**1q***	Z	$S_0 \rightarrow S_1$	3.18	390	98	0.048
	E	$S_0 \rightarrow S_1$	2.52	492		0.001
**1r***	Z	$S_0 \rightarrow S_1$	3.22	385	103	0.000
	E	$S_0 \rightarrow S_1$	2.54	488		0.000
**1s**	Z	$S_0 \rightarrow S_1$	3.13	396	100	0.076
	E	$S_0 \rightarrow S_1$	2.50	496		0.004

**1t**	Z	$S_0 \rightarrow S_1$	3.35	370	124	0.000
	E	$S_0 \rightarrow S_1$	2.51	494		0.003
**1u**	Z	$S_0 \rightarrow S_1$	3.09	401	99	0.108
	E	$S_0 \rightarrow S_1$	2.48	500		0.026
**1v**	Z	$S_0 \rightarrow S_1$	3.15	394	98	0.062
	E	$S_0 \rightarrow S_1$	2.52	492		0.042
**1w**	Z	$S_0 \rightarrow S_1$	3.17	391	95	0.048
	E	$S_0 \rightarrow S_1$	2.55	486		0.000

Table S12 Electronic and Gibbs reaction energies for $Z \rightarrow E$ transformation and activation barriers $E_{a,E\rightarrow Z}$ for back ($E \rightarrow Z$) isomerization (in kcal/mol, T = 298.15 K) of ITI derivatives **1q-1w** in methanol obtained at the M06-2X/6-31+G(d)//6-311++G(2df,2p)/SMD level of theory. Thermal corrections and entropic contributions were obtained with the smaller basis set. Quantities for compounds denoted with * have been calculated using the cc-pVTZ-PP basis set.

Structure	ΔE				ΔG°			
	6-31+G(d)		6-311++G(2df,2p) (cc-pVTZ-PP)		6-31+G(d)		6-311++G(2df,2p) (cc-pVTZ-PP)	
	$Z \rightarrow E$	$E_{a,E\rightarrow Z}$	$Z \rightarrow E$	$E_{a,E\rightarrow Z}$	$Z \rightarrow E$	$E_{a,E\rightarrow Z}$	$Z \rightarrow E$	$E_{a,E\rightarrow Z}$
**1q***	-	-	3.0	14.2	-	-	2.8	13.7
**1r***	-	-	2.3 (2.1)[a]	14.0 (14.2)[a]	-	-	2.3	13.4
**1s**	3.5	14.3	3.2	14.2	2.9	13.8	2.6	13.6
**1t**	2.5	14.2	2.2	14.1	2.5	14.3	2.3	14.2
**1u**	3.9	14.2	3.7	14.4	3.2	13.5	3.1	13.8
**1v**	2.1	15.1	2.1	15.2	2.8	14.7	2.8	14.8
**1w**	2.9	13.7	2.7	13.3	3.2	13.5	3.0	13.1

[a] The values obtained at the DHK2-M06-2X/cc-pVTZ/SMD level.

Table S13 Vertical excitation energies (VEEs) and oscillator strengths (*f*) for the first three excited states of *E*/*Z* isomers of **1v**,**1x-1β** in methanol obtained at the TD-M06-2X/6-311++G(2df,2p)/SMD level, using the non-equilibrium cLR[2] approach.

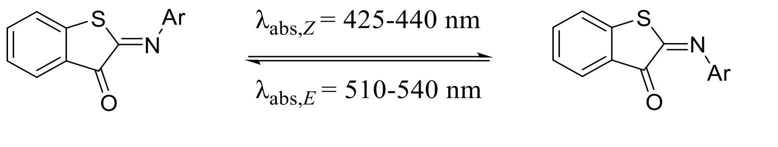

***Z*-1** ***E*-1**

1v: Ar = 2,6-F_2-Bz
1x: Ar = 2,4,6-F_3-Bz
1y: Ar = 2,6-F_2-4-OMe-Bz
1z: Ar = 2,3,4,5,6-F_5-Bz
1α: Ar = 2,3,5,6-F_4-4-OEt-Bz
1β: Ar = 2,3,4,5,6-Cl_5-Bz

Cmpd.	Isomer	Transition	MOs weight	Type	VEE (eV)	λ_{max} (nm)	*f*
**1v**	*Z*	$S_0 \rightarrow S_1$	66 → 71 (0.40)	(n,π*)	3.15	394	0.066
			70 → 71 (0.53)	(π,π*)			
		$S_0 \rightarrow S_2$	66 → 71 (-0.40)	(n,π*)	3.23	384	0.022
			69 → 71 (0.33)	(π,π*)			
			70 → 71 (0.45)	(π,π*)			
		$S_0 \rightarrow S_3$	64 → 71 (0.24)	(n,π*)	3.55	349	0.051
			66 → 71 (0.30)	(n,π*)			
			69 → 71 (0.53)	(π,π*)			
	E	$S_0 \rightarrow S_1$	66 → 71 (-0.29)	(n,π*)	2.52	492	0.044
			69 → 71 (0.37)	(π,π*)			
			70 → 71 (0.49)	(π,π*)			
		$S_0 \rightarrow S_2$	69 → 71 (0.51)	(π,π*)	3.06	405	0.025
			70 → 71 (-0.47)	(π,π*)			
		$S_0 \rightarrow S_3$	63 → 71 (-0.21)	Mixed	3.99	311	0.194
			66 → 71 (0.45)	(n,π*)			
			69 → 71 (0.26)	(n,π*)			
**1x**	*Z*	$S_0 \rightarrow S_1$	70 → 75 (0.37)	(n,π*)	3.14	395	0.083
			74 → 75 (0.57)	(π,π*)			
		$S_0 \rightarrow S_2$	70 → 75 (0.44)	(n,π*)	3.22	385	0.017
			73 → 75 (-0.36)	(π,π*)			
			74 → 75 (-0.39)	(π,π*)			
		$S_0 \rightarrow S_3$	68 → 75 (0.25)	(n,π*)	3.56	348	0.063
			70 → 75 (0.30)	(n,π*)			
			73 → 75 (0.53)	(π,π*)			
	E	$S_0 \rightarrow S_1$	70 → 75 (-0.30)	(n,π*)	2.52	492	0.049
			73 → 75 (0.34)	(π,π*)			
			74 → 75 (0.50)	(π,π*)			
		$S_0 \rightarrow S_2$	73 → 75 (0.52)	(π,π*)	3.06	405	0.024
			74 → 75 (-0.45)	(π,π*)			
		$S_0 \rightarrow S_3$	67 → 75 (-0.22)	Mixed	3.98	312	0.207
			70 → 75 (0.47)	(n,π*)			
			73 → 75 (0.27)	(π,π*)			
**1y**	*Z*	$S_0 \rightarrow S_1$	74 → 79 (0.30)	(n,π*)	3.00	413	0.162
			78 → 79 (0.61)	(π,π*)			

		$S_0 \rightarrow S_2$	74 → 79 (-0.43)	(n,π*)	3.23	384	0.018
			77 → 79 (0.50)	(π,π*)			
		$S_0 \rightarrow S_3$	72 → 79 (-0.28)	Mixed	3.45	359	0.045
			74 → 79 (0.36)	(n,π*)			
			77 → 79 (0.46)	(π,π*)			
	E	$S_0 \rightarrow S_1$	74 → 79 (-0.33)	(n,π*)	2.42	512	0.095
			78 → 79 (0.58)	(π,π*)			
		$S_0 \rightarrow S_2$	77 → 79 (0.60)	(π,π*)	3.07	404	0.030
			78 → 79 (-0.29)	(π,π*)			
		$S_0 \rightarrow S_3$	72 → 79 (0.25)	Mixed	3.76	330	0.255
			74 → 79 (0.45)	(n,π*)			
			77 → 79 (0.29)	(π,π*)			
			78 → 79 (0.23)	(π,π*)			
**1z**	*Z*	$S_0 \rightarrow S_1$	78 → 83 (-0.34)	(n,π*)	3.17	391	0.062
			82 → 83 (0.59)	(π,π*)			
		$S_0 \rightarrow S_2$	78 → 83 (0.49)	(n,π*)	3.23	384	0.011
			81 → 83 (0.29)	(π,π*)			
			82 → 83 (0.37)	(π,π*)			
		$S_0 \rightarrow S_3$	76 → 83 (-0.26)	Mixed	3.64	341	0.061
			78 → 83 (-0.23)	(n,π*)			
			81 → 83 (0.56)	(π,π*)			
	E	$S_0 \rightarrow S_1$	78 → 83 (-0.31)	(n,π*)	2.59	479	0.039
			81 → 83 (-0.42)	(π,π*)			
			82 → 83 (0.44)	(π,π*)			
		$S_0 \rightarrow S_2$	81 → 83 (0.44)	(π,π*)	3.03	409	0.022
			82 → 83 (0.53)	(π,π*)			
		$S_0 \rightarrow S_3$	75 → 83 (-0.24)	(n,π*)	4.06	305	0.209
			78 → 83 (0.43)	(n,π*)			
			81 → 83 (-0.28)	(π,π*)			
**1α**	*Z*	$S_0 \rightarrow S_1$	86 → 91 (0.33)	(n,π*)	3.14	395	0.088
			90 → 91 (0.60)	(π,π*)			
		$S_0 \rightarrow S_2$	86 → 91 (0.50)	(n,π*)	3.23	384	0.011
			89 → 91 (-0.33)	(π,π*)			
			90 → 91 (-0.33)	(π,π*)			
		$S_0 \rightarrow S_3$	83 → 91 (-0.28)	(n,π*)	3.59	345	0.084
			86 → 91 (0.24)	(n,π*)			
			89 → 91 (0.55)	(π,π*)			
	E	$S_0 \rightarrow S_1$	86 → 91 (-0.34)	(n,π*)	2.55	486	0.051
			89 → 91 (0.33)	(π,π*)			
			90 → 91 (0.49)	(π,π*)			
		$S_0 \rightarrow S_2$	89 → 91 (0.52)	(π,π*)	3.04	408	0.025
			90 → 91 (-0.45)	(π,π*)			
		$S_0 \rightarrow S_3$	86 → 91 (0.42)	(n,π*)	3.96	313	0.222
			88 → 91 (0.23)	Mixed			
			89 → 91 (0.28)	(π,π*)			

**1β**	*Z*	$S_0 \rightarrow S_1$	98→103 (0.62)	(n,π*)	3.21	386	0.000
			101→103(-0.26)	(π,π*)			
		$S_0 \rightarrow S_2$	102→103 (0.70)	(π,π*)	3.20	387	0.050
		$S_0 \rightarrow S_3$	95→103 (0.24)	(π,π*)	3.57	347	0.000
			101→103 (0.57)	(π,π*)			
	E	$S_0 \rightarrow S_1$	98 →103 (0.35)	(n,π*)	2.60	477	0.000
			101 →103 (0.59)	(π,π*)			
		$S_0 \rightarrow S_2$	102 →103 (0.70)	(π,π*)	3.08	403	0.020
		$S_0 \rightarrow S_3$	95 →103 (0.28)	(π,π*)	4.07	305	0.000
			98 →103 (0.43)	(n,π*)			
			101→103(-0.31)	(π,π*)			

Table S14 Vertical excitation energies and band separation of the isomers for the $S_0 \rightarrow S_1$ transition of *E*/*Z* isomers of **1v**,**1x-1β** in methanol obtained at the TD-M06-2X/6-311++G(2df,2p)/SMD level, using the non-equilibrium cLR^2 approach.

Structure	Isomer	Transition	VEE (eV)	λ_{max} (nm)	$\Delta\lambda_{max}$ (nm)	*f*
**1v**	Z	$S_0 \rightarrow S_1$	3.15	394	98	0.066
	E	$S_0 \rightarrow S_1$	2.52	492		0.044
**1x**	Z	$S_0 \rightarrow S_1$	3.14	395	97	0.083
	E	$S_0 \rightarrow S_1$	2.52	492		0.049
**1y**	Z	$S_0 \rightarrow S_1$	3.00	413	99	0.162
	E	$S_0 \rightarrow S_1$	2.42	512		0.095
**1z**	Z	$S_0 \rightarrow S_1$	3.17	391	88	0.062
	E	$S_0 \rightarrow S_1$	2.59	479		0.039
**1α**	Z	$S_0 \rightarrow S_1$	3.14	395	91	0.088
	E	$S_0 \rightarrow S_1$	2.55	486		0.051
**1β**	Z	$S_0 \rightarrow S_1$	3.21	386	91	0.000
	E	$S_0 \rightarrow S_1$	2.60	477		0.000

Table S15 Electronic and Gibbs reaction energies for $Z \rightarrow E$ transformation and activation barriers $E_{a,E \rightarrow Z}$ for back ($E \rightarrow Z$) isomerization (in kcal/mol, T = 298.15 K) of ITI derivatives **1v**, **1x-1β** in methanol obtained at the M06-2X/6-31+G(d)//6-311++G(2df,2p)/SMD level of theory. Thermal corrections and entropic contributions were obtained with the smaller basis set.

Structure	ΔE				$\Delta G°$			
	6-31+G(d)		6-311++G(2df,2p)		6-31+G(d)		6-311++G(2df,2p)	
	$Z \rightarrow E$	$E_{a,E \rightarrow Z}$	$Z \rightarrow E$	$E_{a,E \rightarrow Z}$	$Z \rightarrow E$	$E_{a,E \rightarrow Z}$	$Z \rightarrow E$	$E_{a,E \rightarrow Z}$
**1v**	2.1	15.1	2.1	15.2	2.8	14.7	2.8	14.8
**1x**	2.0	14.8	2.0	14.9	2.3	14.4	2.3	14.5

**1y**	2.1	13.6	2.2	13.7	2.2	13.2	2.2	13.2
**1z**	1.5	15.9	1.6	15.8	2.2	15.4	2.3	15.4
**1α**	1.8	15.6	1.7	15.5	2.4	14.8	2.4	14.6
**1β**	1.7	13.9	1.5	13.7	1.0	15.0	0.9	14.9

Table S16 Bond lengths of central C=N and N–C bonds (Å), angle between these two bonds α_{CNC} (°), twisting angle between the thioindoxyl and phenyl moieties (°), orbital energies ε of six higher MOs (eV),[a] and differences between (summed) energies (kcal/mol) of selected orbitals of TS and *E*-isomer of **1m**, **1u**, and **1v** in methanol obtained at the M06-2X/6–31+G(d)//6-311++G(2df,2p) level. The selected MOs are displayed in Figure S4.17.

Cmpd.	R_3/R_4	Form	*d*(CN)/*d*(NC)	α_{CNC}	ϑ	Orbital energies ε (eV)								
						π-HOMO	π-1	π-2	π-3	**n**	n-1/π-4	Δ_{TS-E} ε(n) (kcal/mol)	Δ_{TS-E} ε(HOMO) +ε(n) (kcal/mol)	Δ_{TS-E} $\Sigma\ \varepsilon$(1-6) (kcal/mol)
**1m**	H/CH_3	*E*	1.261/1.421	122.6	88.3	-7.56	-7.84	-8.28	-9.02	**-9.49**	-9.87			
		TS	1.224/1.360	177.3	51.4	-7.04	-8.34	-8.96	-9.15	**-7.76**	-10.01	39.9	52.0	18.8
					Δ (eV) =	0.52	-0.50	-0.68	-0.13	**1.73**	-0.13			
**1u**	H/F	*E*	1.264/1.410	123.1	-64.5	-7.65	-8.03	-8.52	-9.03	**-9.55**	-10.05			
		TS	1.228/1.354	177.3	0.0	-7.50	-8.52	-8.75	-8.96	**-7.92**	-10.20	37.7	41.3	22.5
					Δ (eV) =	0.15	-0.50	-0.23	0.07	**1.64**	-0.15			
**1v**	F/F	*E*	1.267/1.402	123.6	-56.8	-7.69	-8.17	-8.53	-9.03	**-9.59**	-10.17			
		TS	1.230/1.345	176.0	-25.5	-7.48	-8.63	-8.90	-9.02	**-7.92**	-10.20	38.4	43.4	24.1
					Δ (eV) =	0.22	-0.46	-0.37	0.01	**1.67**	-0.03			

[a] Note that the n orbital (in bold) is the HOMO-4 and HOMO-1 in the *E*-form and the TS, respectively.

Table S17 Dipole moments (in D) for the ground (S_0) and excited (S_1) states of structures **1a**, **1k** in methanol calculated at the (TD-)M06-2X/6-311++G(2df,2p)/SMD level of theory.

	Isomer	S_0				S_1			
**Structure**		*x*	*y*	*z*	***Tot.***	*x*	*y*	*z*	***Tot.***
**1a**	Z	2.53	-6.02	0.86	**6.58**	0.70	-8.03	-1.01	**8.12**
	E	-5.40	-0.96	0.43	**5.50**	1.08	-0.29	0.62	**1.28**
**1k**	Z	4.36	-7.01	-0.68	**8.29**	16.38	-9.14	-0.90	**18.78**
	E	-0.42	1.99	1.03	**2.28**	-9.01	0.47	1.29	**9.12**

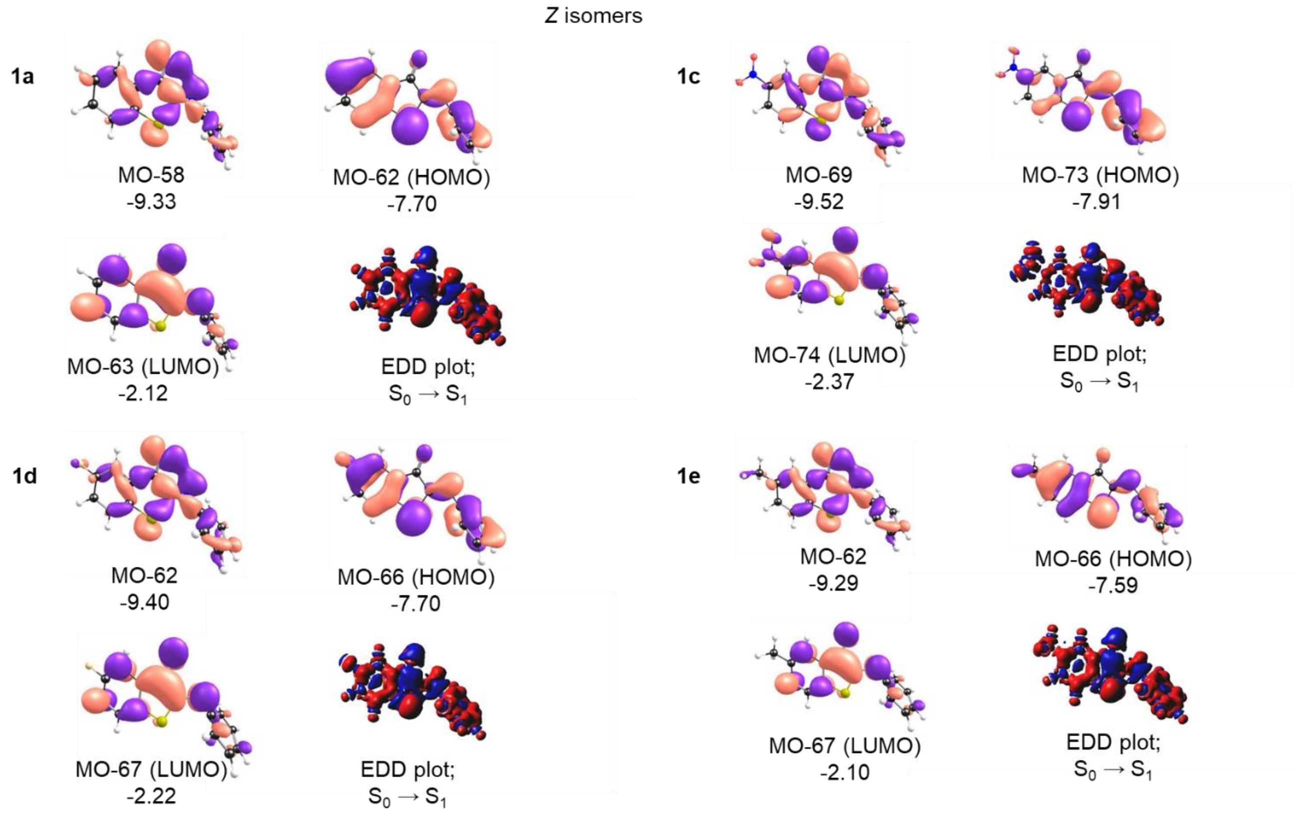

Figure S111 MOs involved in the $S_0 \rightarrow S_1$ transition of *Z* isomers for **1a, 1c-1e** (orbital energies are given in eV, contour value = 0.025) and the corresponding electron density difference (EDD) plot (red = decrease, blue = increase, isovalue = 0.0015 a.u.) obtained at the (TD)-M06-2X/6-31++G(2df,2p)/SMD (methanol) level of theory.

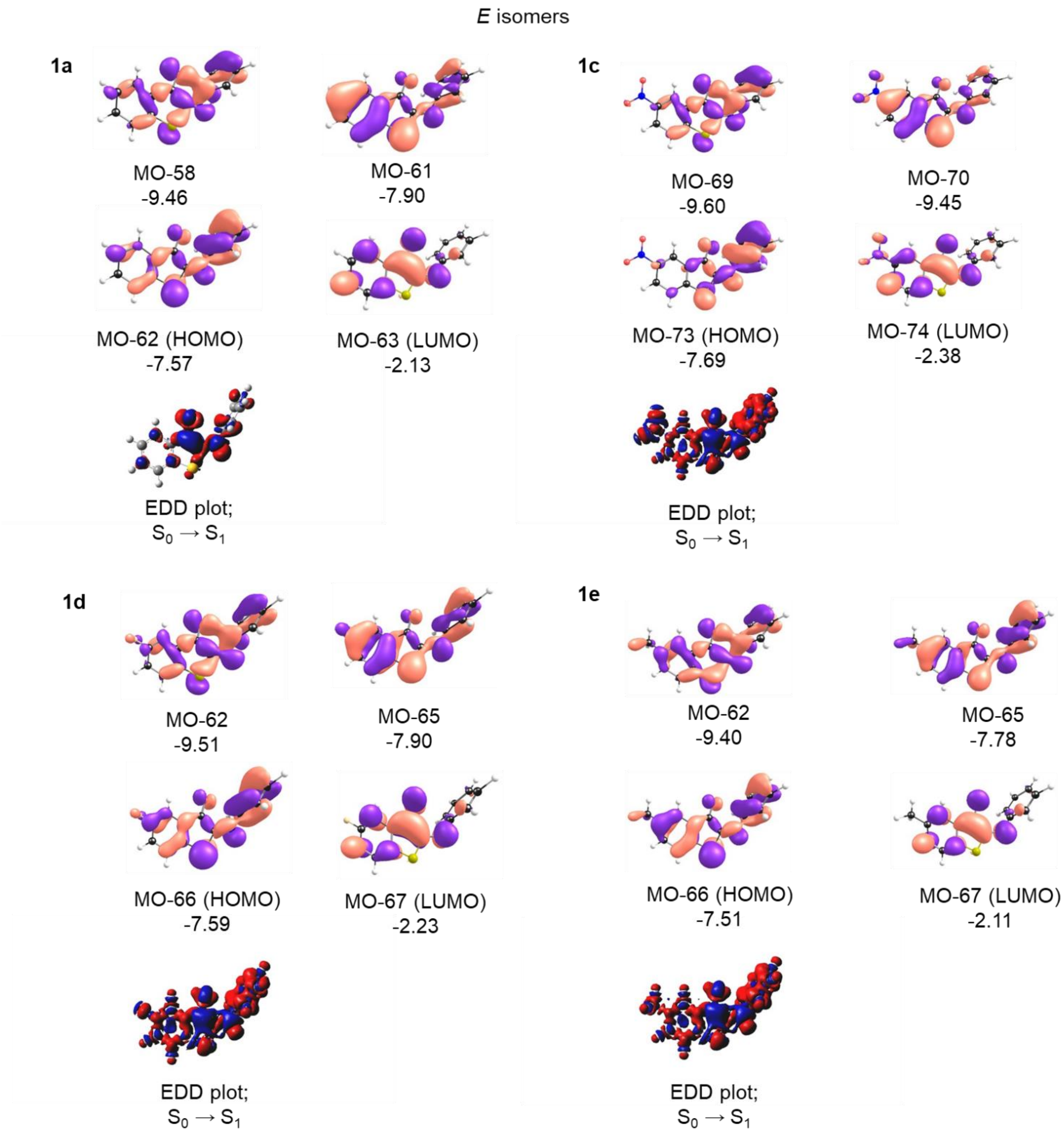

Figure S112 MOs involved in the $S_0 \rightarrow S_1$ transition of *E* isomers of **1a, 1c-1e** (orbital energies are given in eV, contour value = 0.025) and the corresponding EDD plot (red = decrease, blue = increase, isovalue = 0.0015 a.u.) obtained at the (TD)-M06-2X/6-31++G(2df,2p)/SMD (methanol) level of theory.

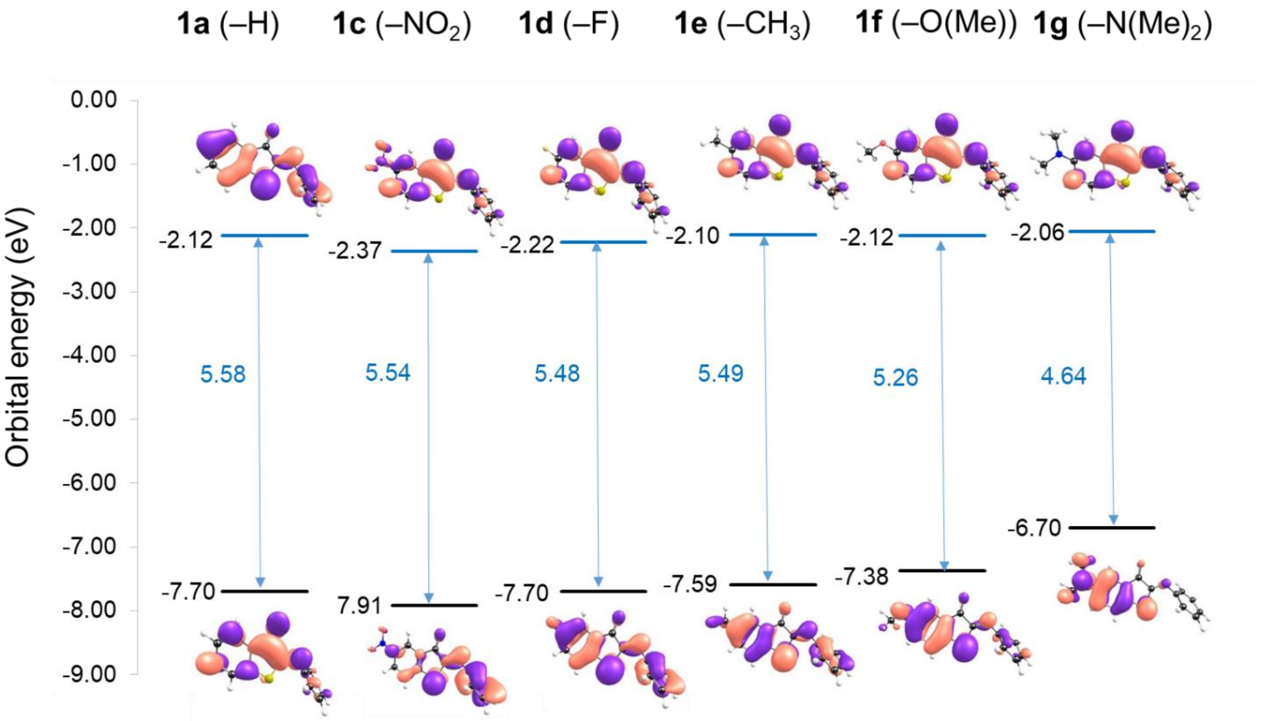

Figure S113 Frontier MO energy diagram of the *Z* isomer for compounds **1a**, **1c**-**1g**.

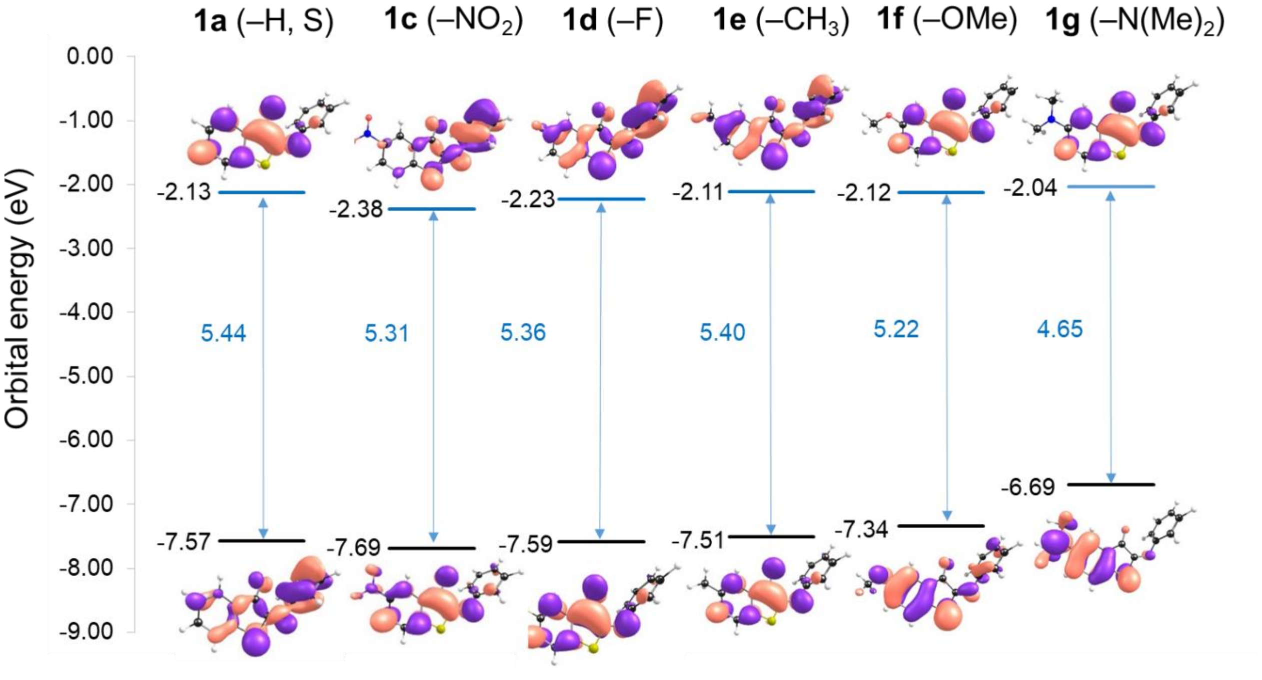

Figure S114 Frontier MO energy diagram of the *E* isomer for compounds **1a**, **1c**-**1g**.

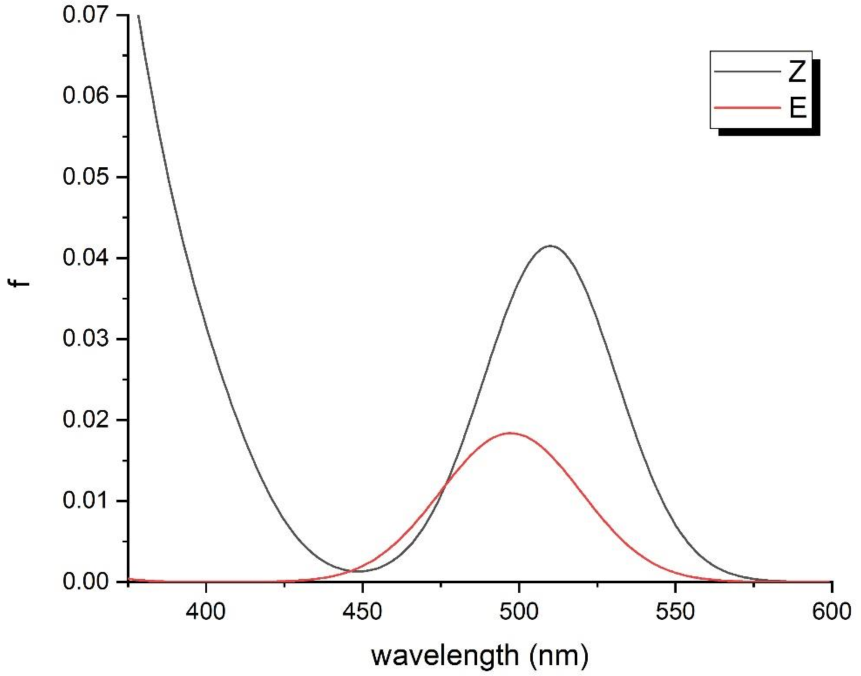

Figure S115 Predicted absorption maxima of *Z*-**1g** and *E*-**1g** obtained at the TD-M06-2X/6-31++G(2df,2p)/cLR^2-SMD (methanol) level of theory.

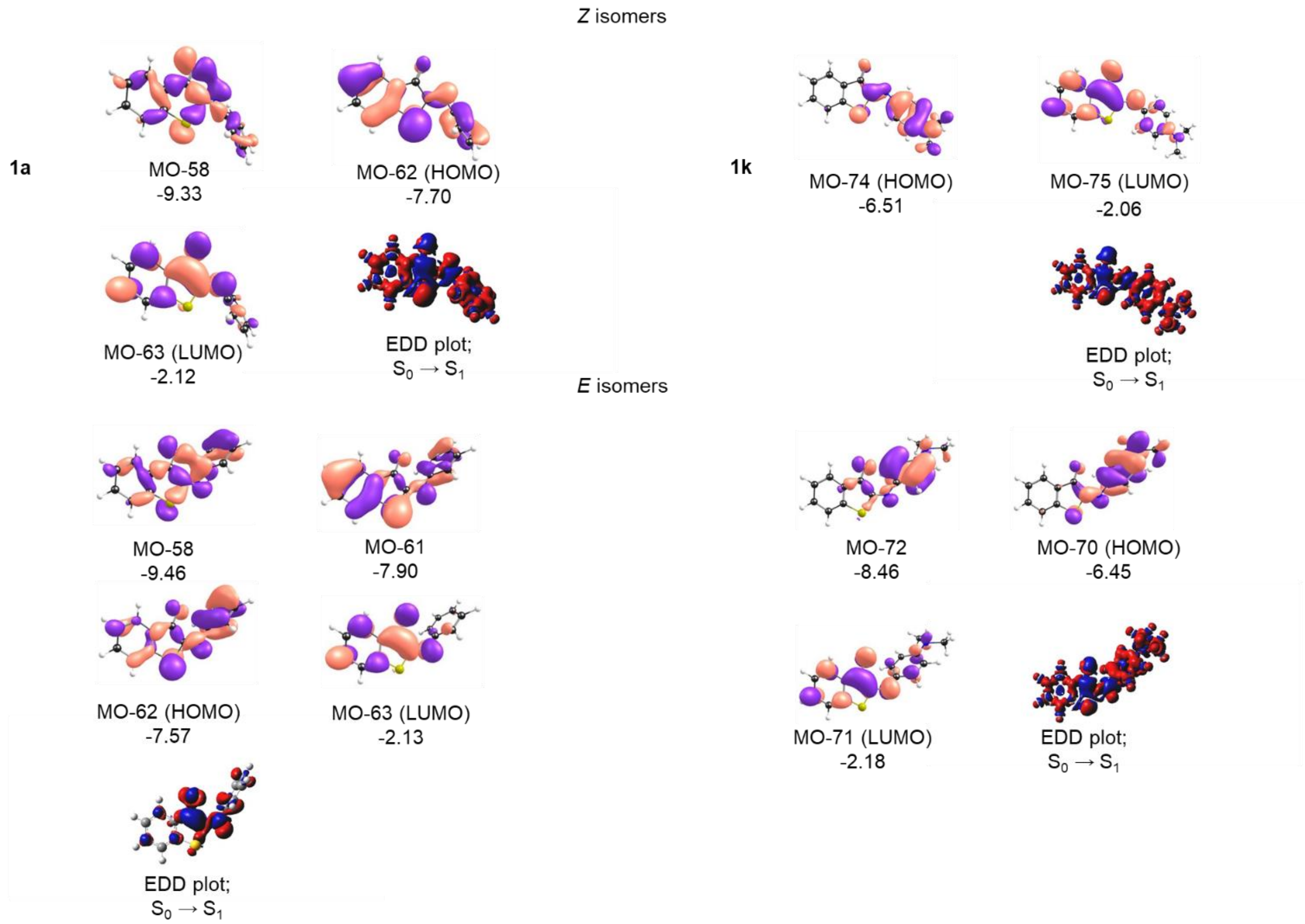

Figure S116 MOs involved in the $S_0 \rightarrow S_1$ transition of *Z* and *E* isomers of **1a** and **1k** (orbital energies are given in eV, contour value = 0.025) and the corresponding EDD plot (red = decrease, blue = increase, isovalue = 0.0015 a.u.) obtained at the (TD)-M06-2X/6-31++G(2df,2p)/SMD (methanol) level of theory.

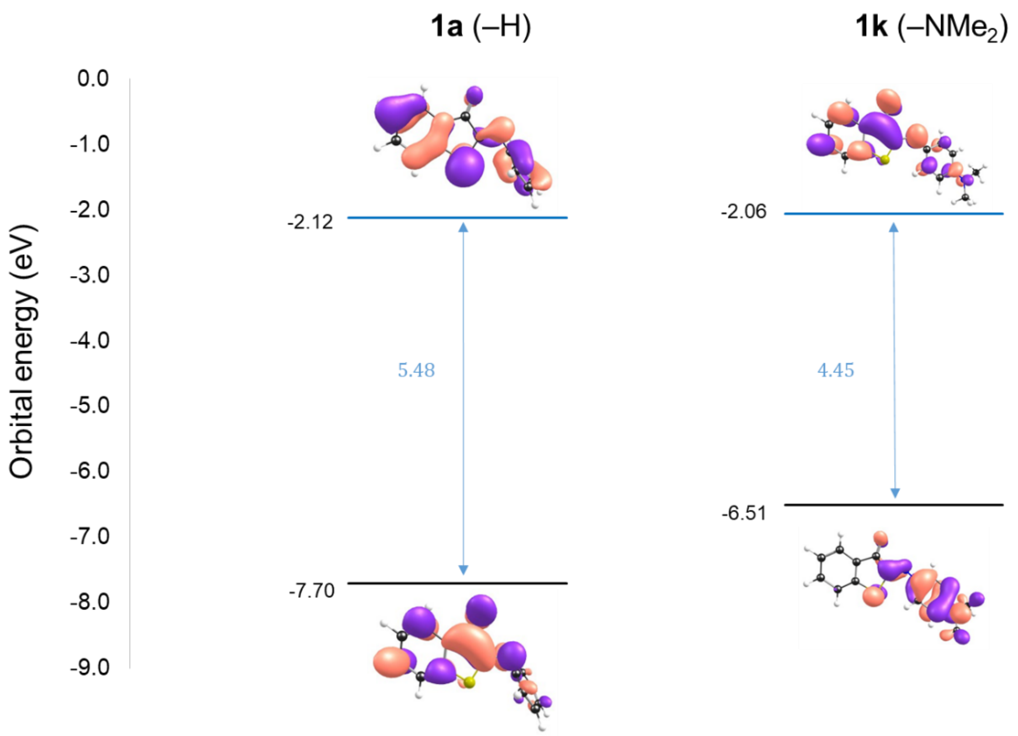

Figure S117 Frontier MO energy diagram of the *Z* isomer for compounds **1a** and **1k**.

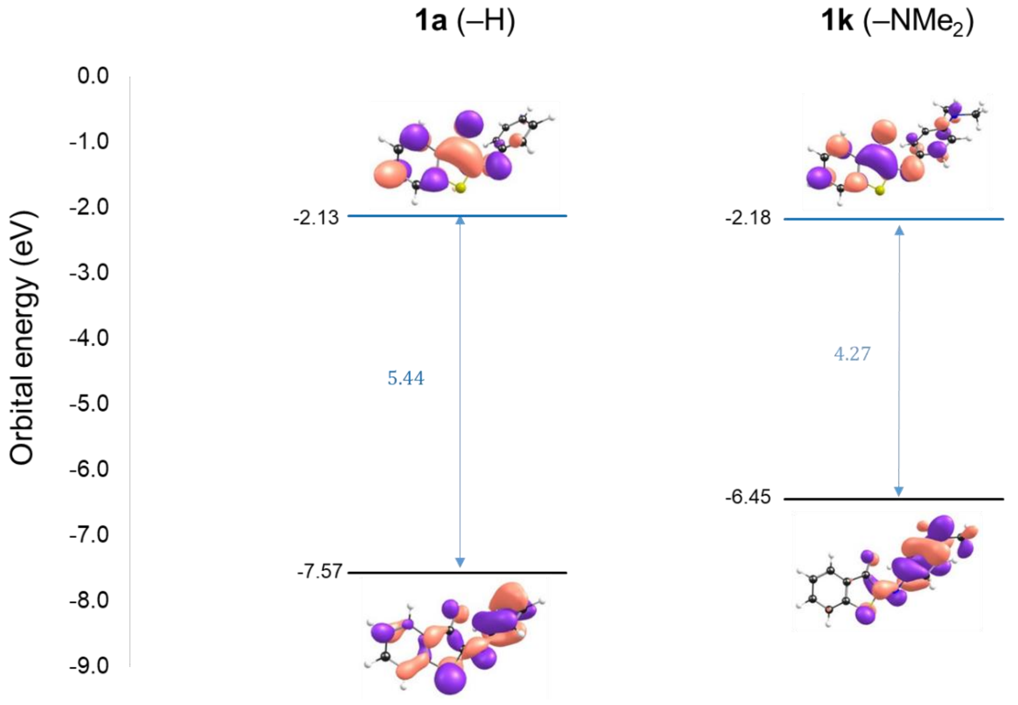

Figure S118 Frontier MO energy diagram of the *E* isomer for compounds **1a** and **1k**.

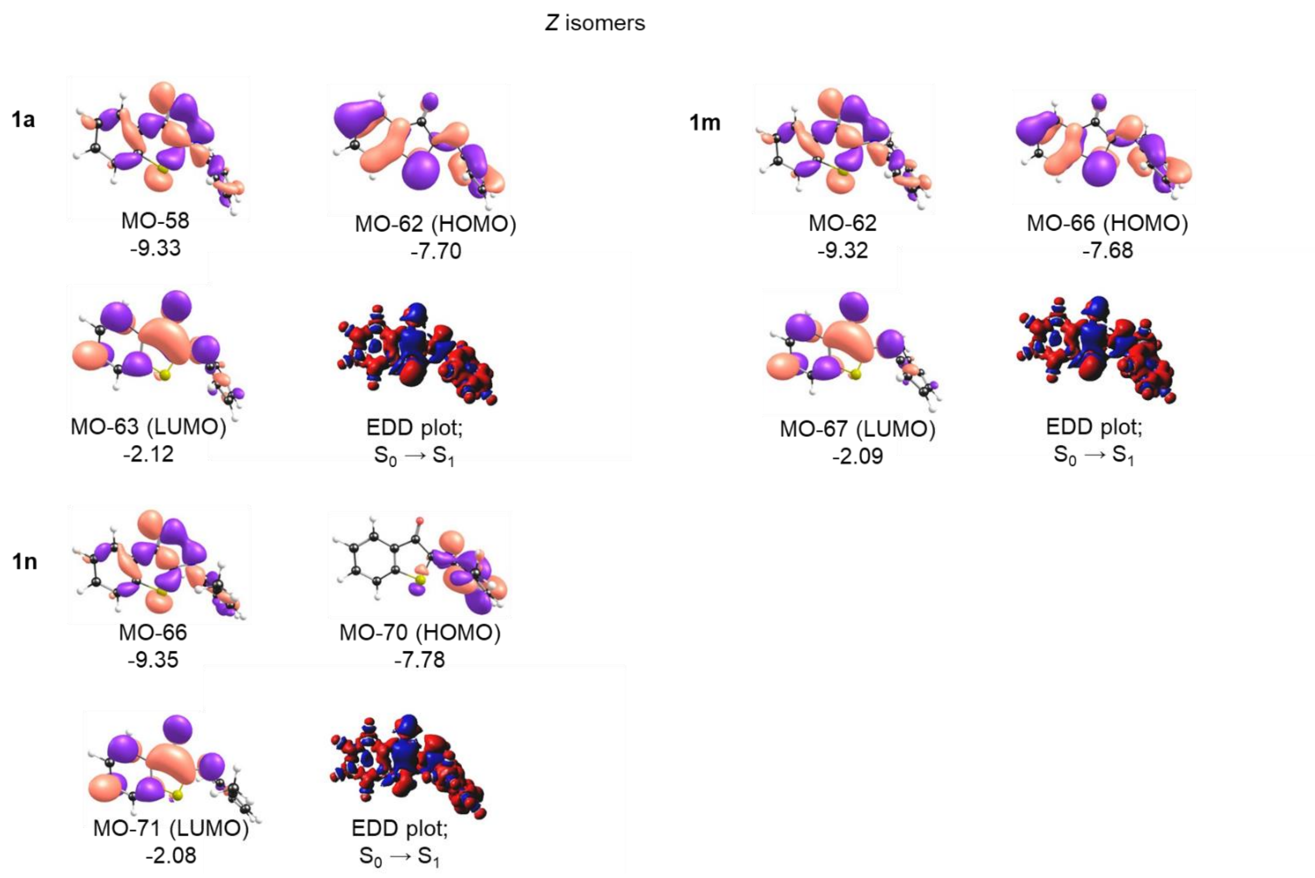

Figure S119 MOs involved in the $S_0 \rightarrow S_1$ transition of *Z* isomers of **1a, 1m** and **1n** (orbital energies are given in eV, contour value = 0.025) and the corresponding EDD plot (red = decrease, blue = increase, isovalue = 0.0015 a.u.) obtained at the (TD)-M06-2X/6-31++G(2df,2p)/SMD (methanol) level of theory.

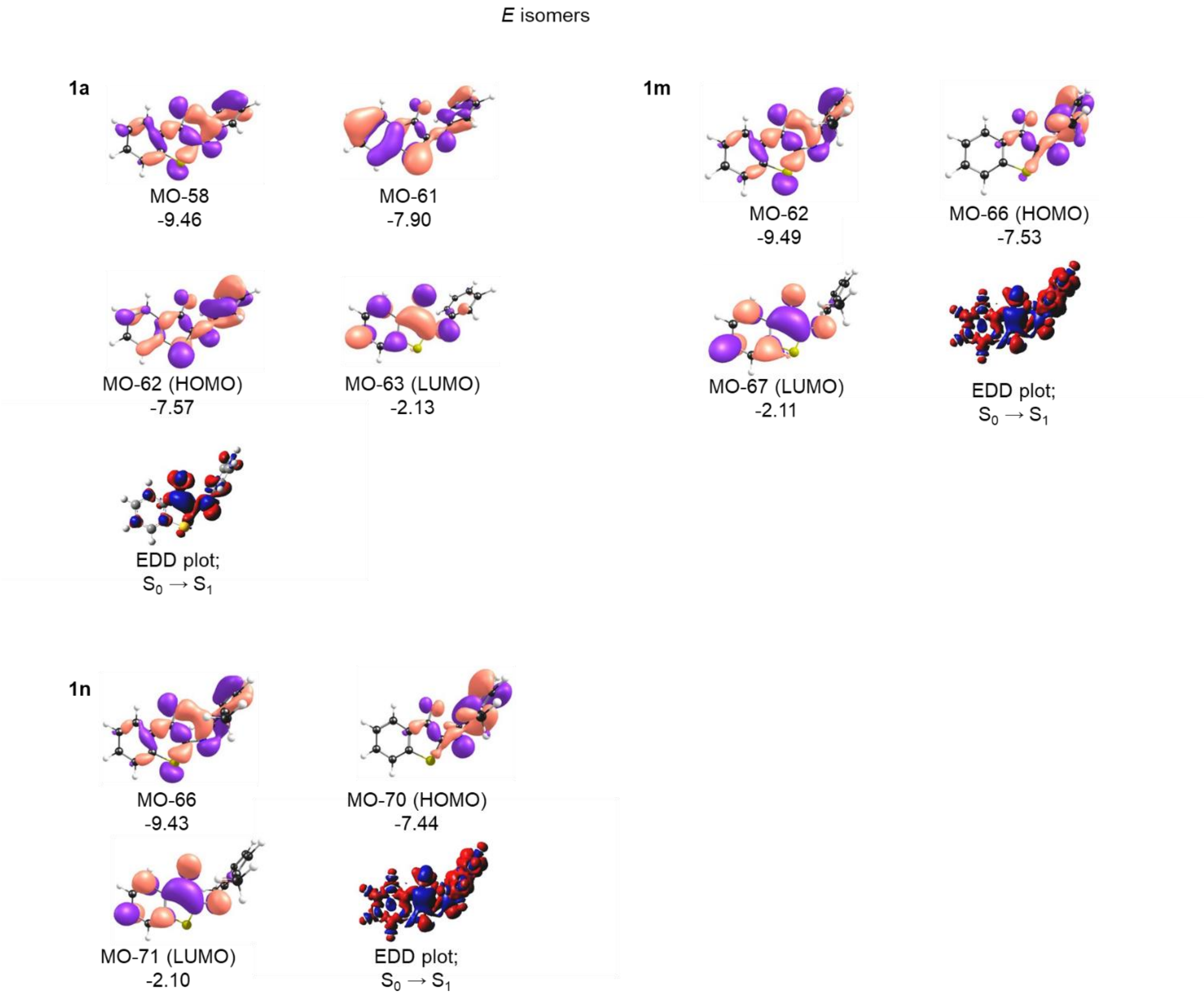

Figure S120 MOs involved in the $S_0 \rightarrow S_1$ transition of *E* isomers of **1a, 1m** and **1n** (orbital energies are given in eV, contour value = 0.025) and the corresponding EDD plot (red = decrease, blue = increase, isovalue = 0.0015 a.u.) obtained at the (TD)-M06-2X/6-31++G(2df,2p)/SMD (methanol) level of theory.

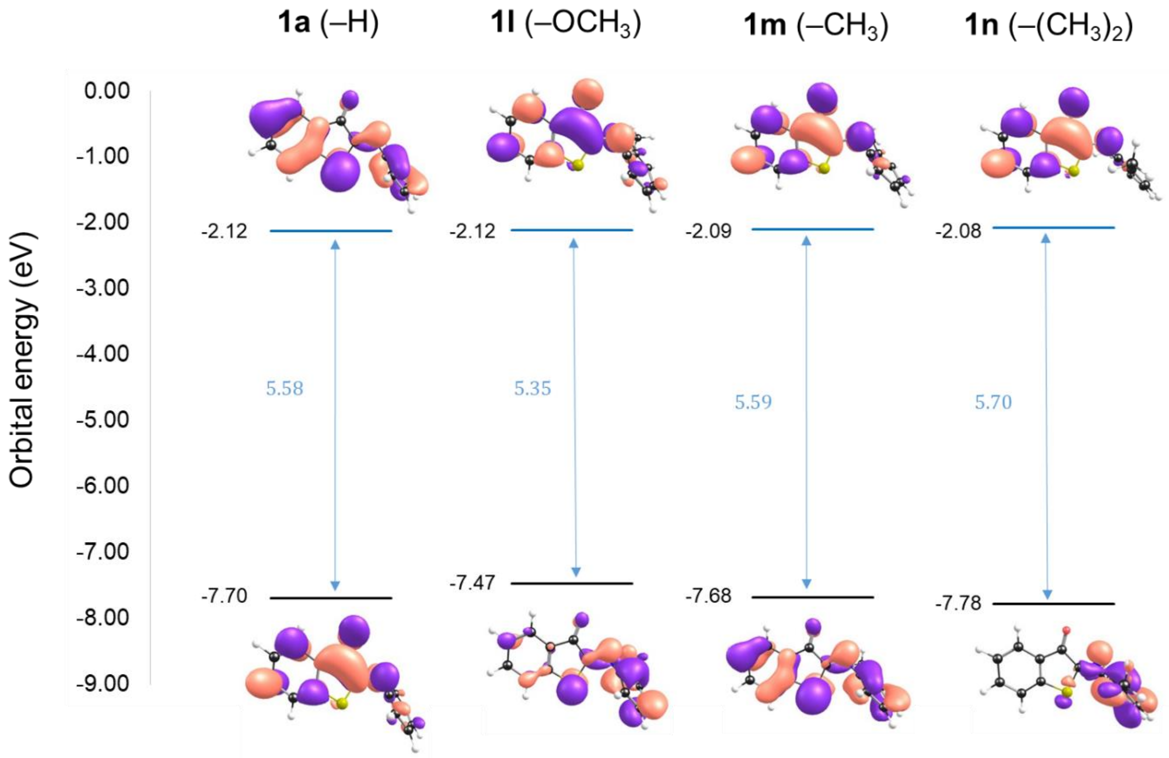

Figure S121 Frontier MO energy diagram of the *Z* isomer for compounds **1a**, **1l**, **1m** and **1n**.

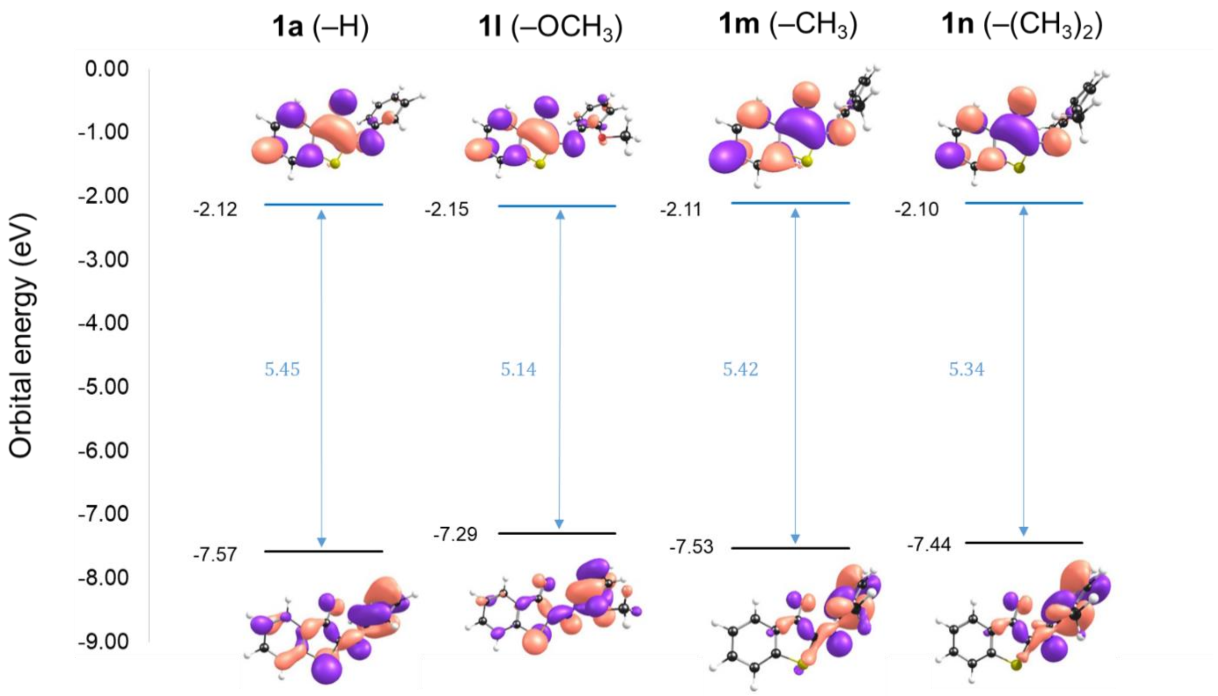

Figure S122 Frontier MO energy diagram of the *E* isomer for compounds **1a**, **1l**, **1m** and **1n**.

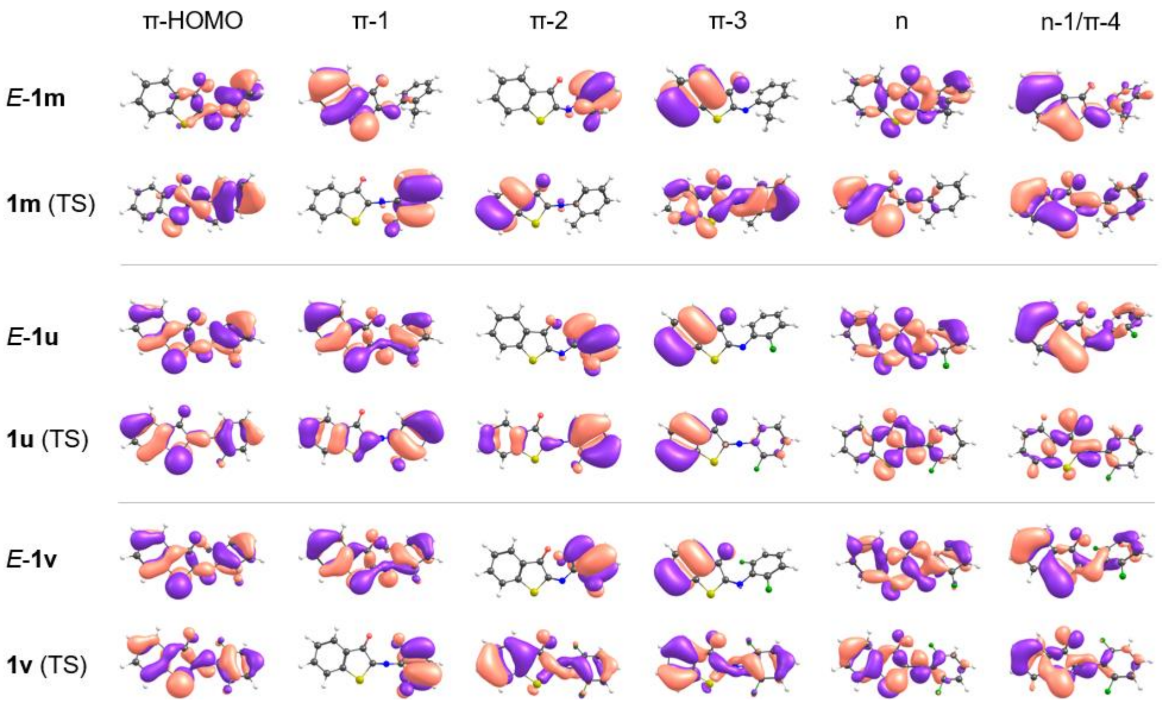

Figure S123 Six higher occupied MOs (HOMO – HOMO-5) of TS and E-isomer of **1m**, **1u**, and **1v** in methanol obtained at the M06-2X/6–31+G(d)//6-311++G(2df,2p)/SMD level.

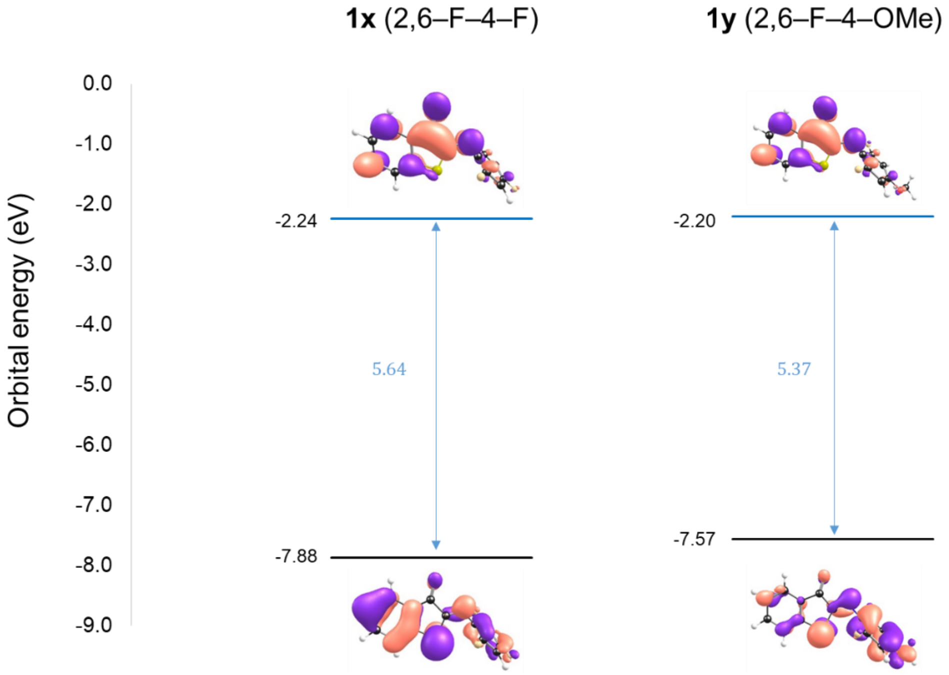

Figure S124 Frontier MO energy diagram of the *Z* isomer for compounds **1x** and **1y**.

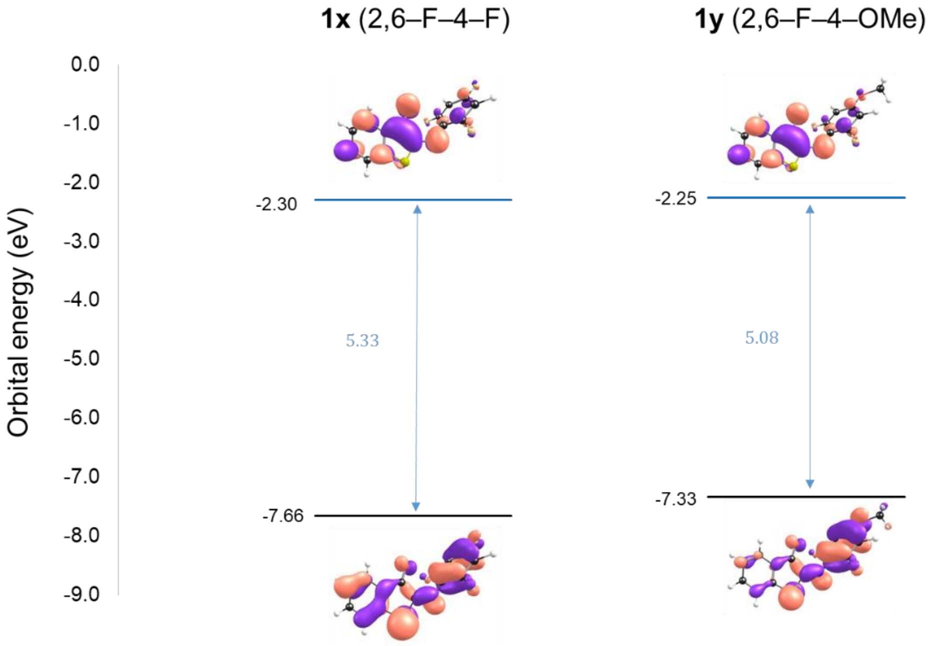

Figure S125 Frontier MO energy diagram of the *E* isomer for compounds **1x** and **1y**.

S5. Quantum yield calculations

Table S18 Quantum yield calculations

ITI	ε_Z (M^{-1} cm^{-1}) (420 or 430 nm)	OD in 1 cm cell @420 or 430 nm	Pulse energy (mJ)	Excitation wavelength (nm)	$\phi_{Z\text{->}E}$ (%)
**1b**	1800	1.51	1.60	455	16
**1c**	2900	1.65	1.80	430	11
**1d**	4600	1.60	1.80	430	6.8
**1e**	4200	1.36	1.60	430	6.5
**1f**	1420	1.37	4.00	460	3.9
**1g**	1000	1.35	1.00	430	N.A.
**1i**	4000	1.46	3.20	430	0.40
**1k**	27000	1.49	4.60	430	0.0062
**1l**	3570	1.59	3.80	430	6.1
**1m**	1600	1.54	2.80	430	12
**1n**	2500	1.38	2.40	420	2.1
**1o**	3030	1.71	3.80	430	6.2
**1p**	3010	1.76	3.80	430	5.7
**1q**	2120	1.32	3.80	430	4.6
**1r**	1480	1.27	3.80	430	3.6
**1s**	1410	1.38	3.80	430	8.4
**1t**	2740	1.78	3.80	430	2.6
**1u**	2000	1.41	2.80	430	7.5
**1v**	2100	1.11	2.40	420	4.8
**1w**	1400	1.50	3.80	430	7.9
**1x**	2380	1.37	2.20	420	4.6
**1y**	4000	1.09	2.20	420	0.047
**1z**	2020	1.34	2.20	420	1.1
**1α**	1760	1.37	2.20	420	5.7
**1β**	598	1.67	2.20	420	7.6

S6. References

1. Y. Zhao and D. G. Truhlar, *Theoretical Chemistry Accounts*, 2007, **120**, 215-241.
2. R. Ditchfield, W. J. Hehre and J. A. Pople, *The Journal of Chemical Physics*, 1971, **54**, 724-728.
3. T. H. Dunning, *The Journal of Chemical Physics*, 1989, **90**, 1007-1023.
4. K. A. Peterson, D. Figgen, E. Goll, H. Stoll and M. Dolg, *The Journal of chemical physics*, 2003, **119**, 11113-11123.
5. B. A. Hess, *Phys Rev A Gen Phys*, 1986, **33**, 3742-3748.
6. B. A. Hess, *Phys Rev A Gen Phys*, 1985, **32**, 756-763.
7. G. Jansen and B. A. Hess, *Phys Rev A Gen Phys*, 1989, **39**, 6016-6017.
8. M. Douglas and N. M. Kroll, *Annals of Physics*, 1974, **82**, 89-155.
9. B. P. Pritchard, D. Altarawy, B. Didier, T. D. Gibson and T. L. Windus, *J Chem Inf Model*, 2019, **59**, 4814-4820.
10. A. K. Wilson, D. E. Woon, K. A. Peterson and T. H. Dunning, *The Journal of Chemical Physics*, 1999, **110**, 7667-7676.
11. W. A. De Jong, R. J. Harrison and D. A. Dixon, *The Journal of Chemical Physics*, 2001, **114**, 48-53.
12. A. V. Marenich, C. J. Cramer and D. G. Truhlar, *The Journal of Physical Chemistry B*, 2009, **113**, 6378-6396.
13. C. A. Guido, A. Chrayteh, G. Scalmani, B. Mennucci and D. Jacquemin, *J Chem Theory Comput*, 2021, **17**, 5155-5164.
14. M. Caricato, B. Mennucci, J. Tomasi, F. Ingrosso, R. Cammi, S. Corni and G. Scalmani, *J Chem Phys*, 2006, **124**, 124520.
15. M. e. Frisch, G. Trucks, H. Schlegel, G. Scuseria, M. Robb, J. Cheeseman, G. Scalmani, V. Barone, B. Mennucci and G. Petersson, *Wallingford CT*, 2009.
16. M. e. Frisch, G. Trucks, H. B. Schlegel, G. Scuseria, M. Robb, J. Cheeseman, G. Scalmani, V. Barone, G. Petersson and H. Nakatsuji, *Journal*, 2016.
17. J. J. Snellenburg, S. Laptenok, R. Seger, K. M. Mullen and I. H. van Stokkum, *Journal of Statistical Software*, 2012, **49**, 1-22.
18. M. Jang, T. Lim, B. Y. Park and M. S. Han, *J Org Chem*, 2022, **87**, 910-919.
19. F. Kink, M. P. Collado, S. Wiedbrauk, P. Mayer and H. Dube, *Chemistry*, 2017, **23**, 6237-6243.
20. K. Naiman, H. Dračínská, M. t. Martínková, M. Sulc, M. Dracinsky, L. Kejikova, P. Hodek, J. Hudecek, J. Liberda and H. H. Schmeiser, *Chemical research in toxicology*, 2008, **21**, 1610-1621.
21. A. Purkait, S. K. Roy, H. K. Srivastava and C. K. Jana, *Organic letters*, 2017, **19**, 2540-2543.
22. S. Fountoulaki, P. L. Gkizis, T. S. Symeonidis, E. Kaminioti, A. Karina, I. Tamiolakis, G. S. Armatas and I. N. Lykakis, *Advanced Synthesis & Catalysis*, 2016, **358**, 1500-1508.
23. R.-Q. Ran, S.-D. Xiu and C.-Y. Li, *Organic letters*, 2014, **16**, 6394-6396.
24. H.-L. Liu, X.-T. Li, H.-Z. Tian and X.-W. Sun, *Organic Letters*, 2021, **23**, 4579-4583.
25. F. Tibiletti, M. Simonetti, K. M. Nicholas, G. Palmisano, M. Parravicini, F. Imbesi, S. Tollari and A. Penoni, *Tetrahedron*, 2010, **66**, 1280-1288.
26. X. Yang, G. Ma, S. Zheng, X. Qin, X. Li, L. Du, Y. Wang, Y. Zhou and M. Li, *Journal of the American Chemical Society*, 2020, **142**, 9460-9470.

27. T. Soeta, S. Shitaya, T. Okuno, S. Fujinami and Y. Ukaji, *Tetrahedron*, 2016, **72**, 7901-7905.
28. X. Huang, J. Xue and Y. Yang, *Synlett*, 2007, **2007**, 1533-1536.
29. M. W. H. Hoorens, M. Medved, A. D. Laurent, M. Di Donato, S. Fanetti, L. Slappendel, M. Hilbers, B. L. Feringa, W. Jan Buma and W. Szymanski, *Nat Commun*, 2019, **10**, 2390.
